# GATOR2-dependent mTORC1 activity is a therapeutic vulnerability in *FOXO1* fusion–positive rhabdomyosarcoma

**DOI:** 10.1172/jci.insight.162207

**Published:** 2022-12-08

**Authors:** Jacqueline Morales, David V. Allegakoen, José A. Garcia, Kristen Kwong, Pushpendra K. Sahu, Drew A. Fajardo, Yue Pan, Max A. Horlbeck, Jonathan S. Weissman, W. Clay Gustafson, Trever G. Bivona, Amit J. Sabnis

**Affiliations:** 1 Division of Pediatric Oncology, Department of Pediatrics, and; 2 Division of Hematology-Oncology, Department of Medicine, UCSF, San Francisco, California, USA.; 3 College of Osteopathic Medicine, Kansas City University, Kansas City, Missouri, USA.; 4 School of Medicine, University of Nevada, Reno, Nevada, USA.; 5 Department of Cellular and Molecular Pharmacology, UCSF, San Francisco, California, USA.; 6 Howard Hughes Medical Institute, Chevy Chase, Maryland, USA.; 7 Boston Children’s Hospital, Boston, Massachusetts, USA.; 8 Whitehead Institute, Boston, Massachusetts, USA.; 9 Revolution Medicines, Redwood City, California, USA.; 10 Chan Zuckerberg Biohub, San Francisco, California, USA.

**Keywords:** Genetics, Oncology, Cancer, Drug therapy, Signal transduction

## Abstract

Oncogenic *FOXO1* gene fusions drive a subset of rhabdomyosarcoma (RMS) with poor survival; to date, these cancer drivers are therapeutically intractable. To identify new therapies for this disease, we undertook an isogenic CRISPR-interference screen to define *PAX3-FOXO1*–specific genetic dependencies and identified genes in the GATOR2 complex. GATOR2 loss in RMS abrogated aa-induced lysosomal localization of mTORC1 and consequent downstream signaling, slowing G1-S cell cycle transition. In vivo suppression of GATOR2 impaired the growth of tumor xenografts and favored the outgrowth of cells lacking *PAX3-FOXO1*. Loss of a subset of GATOR2 members can be compensated by direct genetic activation of mTORC1. *RAS* mutations are also sufficient to decouple mTORC1 activation from GATOR2, and indeed, fusion-negative RMS harboring such mutations exhibit aa-independent mTORC1 activity. A bisteric, mTORC1-selective small molecule induced tumor regressions in fusion-positive patient-derived tumor xenografts. These findings highlight a vulnerability in *FOXO1* fusion–positive RMS and provide rationale for the clinical evaluation of bisteric mTORC1 inhibitors, currently in phase I testing, to treat this disease. Isogenic genetic screens can, thus, identify potentially exploitable vulnerabilities in fusion-driven pediatric cancers that otherwise remain mostly undruggable.

## Introduction

Rhabdomyosarcoma (RMS) is the most common soft tissue sarcoma of childhood and is comprises 2 genetically defined subtypes: *FOXO1* fusion positive (FP) and fusion negative (FN), which are characterized by activating mutations in receptor tyrosine kinase (RTK)/RAS pathways (1, 2). Despite genomic, histologic, and clinical factors that distinguish FP from FN disease, patients with either diagnosis are treated with the same backbone of chemoradiotherapy. Only 60% of patients are cured, and relapse is frequently lethal. Patients with *FOXO1* FP disease have worse overall and relapse-free survival than do patients with FN disease (3).

Recurrent translocations between chromosomes 2 and 13 in alveolar RMS were mapped to rearrangements of *PAX3* over 20 years ago (4). The expressed product of the *PAX3-FOXO1* translocation retains the N-terminal DNA binding elements of PAX3 and the C-terminal transactivation domain of FOXO1. A related but less frequent t(1;13) rearrangement results in the *PAX7-FOXO1* fusion, associated with similar biological and clinical outcomes as *PAX3-FOXO1* (5). PAX3-FOXO1 binds at super-enhancers distal from target genes and enables active chromatin states through recruitment of histone acetylation machinery (1). Targeting FP RMS through either BET/bromodomain inhibitors or targeted degradation of the fusion protein are new and promising directions for therapy. To date, however, these approaches have not shown conclusive benefit in patients, and thus ongoing efforts to identify or refine therapies for *FOXO1* FP RMS remain vital.

The mTOR kinase controls the growth of normal and cancerous cells. As part of the mTORC1 complex, mTOR phosphorylates p70S6K to promote ribosome assembly, and 4EBP1 to permit m^7^-GTP cap-dependent mRNA translation. The activity of mTORC1 is controlled both by growth-factor signaling that inhibits the TSC1/TSC2 complex, and aa sufficiency signals. In response to aa, including leucine and arginine, the pentameric GATOR2 complex inhibits the GATOR1 complex, which, in turn, prevents mTORC1 localization to its site of activity at the lysosome by accelerating GTP hydrolysis by RAGA and RAGB (6). Thus, GATOR2 is an aa-responsive, positive regulator of mTORC1.

The allosteric mTOR inhibitor temsirolimus delays disease progression but does not improve the survival of patients with relapsed RMS (7). These allosteric inhibitors inhibit phosphorylation of p70S6K, but not 4EBP1, which limits their efficacy in certain cancer models (8, 9). Active-site kinase inhibitors suppress phosphorylation of both p70S6K and 4EBP1 but also inhibit mTORC2, resulting in dose-limiting hyperglycemia (10, 11) and removal of AKT-dependent feedback inhibition of RTK signaling (12). Newer bisteric inhibitors with combined active-site and allosteric binding suppress p70S6K and 4EBP1 phosphorylation, have been optimized for mTORC1 selectivity, and might improve upon the modest clinical success of rapalogs in RMS (13). However, a cellular mechanistic understanding of how RMS-initiating genetic events (i.e., *FOXO1* fusions or mutations in the RAS pathway) create mTOR dependence is lacking, which limits the precise use and further development of these agents in RMS.

We reasoned that genetic screens could uncover targetable dependencies downstream of *PAX3-FOXO1*. Prior efforts to do so have used shRNA libraries and compared FP and FN cell lines (14). However, because FN RMSs harbor genetic changes besides lack of *PAX3-FOXO1*, such as large-scale copy number alterations and *RAS* mutations (15, 16), an isogenic system might provide a better means to identify *PAX3-FOXO1* dependencies. By engineering and deploying such a system coupled with a more precise CRISPR/Cas9 genetic screening platform, we report here that *PAX3-FOXO1* FP RMS depends on GATOR2 for cellular proliferation, and we identify a clinically translatable therapeutic approach to target this potential vulnerability in patients with RMS.

## Results

### A CRISPR-interference screen identifies genes required for the growth of FP RMS cells.

**Figure 1:**
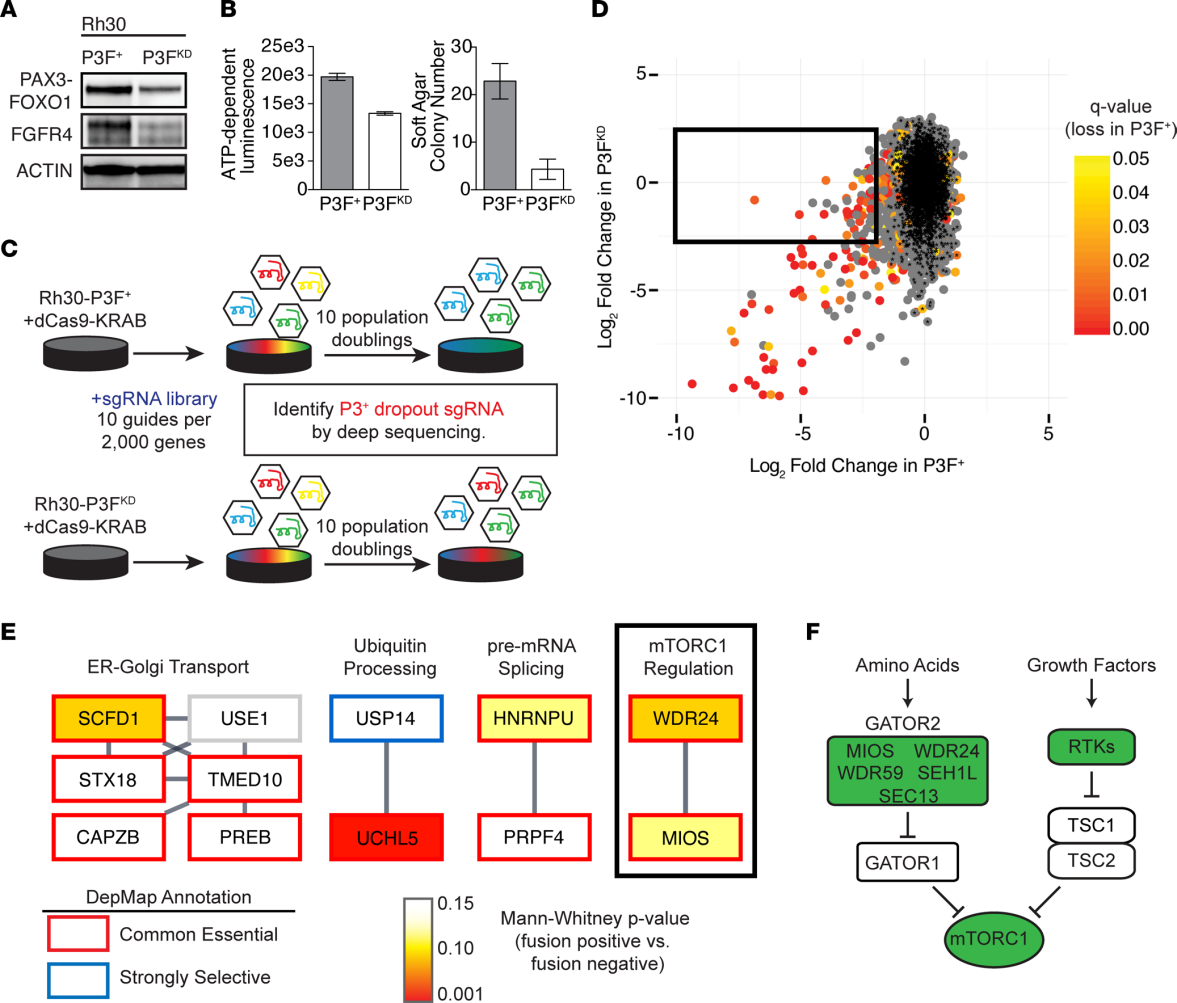
An isogenic screen identifies *PAX3-FOXO1* dependencies. (**A**) Immunoblot of lysates from P3F^+^ and P3F^KD^ cells demonstrating decreased abundance of both PAX3-FOXO1 and its target FGFR4. (**B**) Differential growth of P3F^+^ and P3F^KD^ cells in 2D (left: CellTiterGlo after 72 hours of tissue culture; *n* = 10 wells per condition) or 3D (right: crystal violet quantitation of soft agar colony formation in 0.4% agarose over 4 weeks; *n* = 3 wells per condition) growth. (**C**) Schematic of CRISPR-interference screen to identify *PAX3-FOXO1* genetic dependencies. Each condition was carried out in experimental duplicate. (**D**) Screen results. Gene-level scores are plotted on the basis of log2 fold-change in P3F^+^ (*x*-axis) or P3F^KD^ (*y*-axis) conditions. Color indicates FDR-adjusted *P* value by Mann-Whitney test for significance of fold-change in the P3F^+^ condition. Black asterisks represent negative control sgRNA. The black box indicates genes selected for further study. (**E**) Results from the box in **D** arranged by physical interactions identified in the STRING database (20). Outlines correspond to selectivity calls from the DepMap database (19). Coloring indicates the *P* value, by Mann-Whitney test, for stronger CERES gene effect score in FP (*n* = 6) rather than FN (*n* = 5) cell lines in the DepMap. (**F**) Schematic of the role of the GATOR2 complex, including *MIOS* and *WDR24*, in regulation of mTORC1. Positive regulators of mTORC1 are shown in green.

We developed a system to identify genes that are essential to the growth and survival of *PAX3-FOXO1–*positive cells, given the poor outcomes for FP RMS. We transduced FP Rh30 cells with either a lentiviral shRNA construct targeting the *PAX3-FOXO1* fusion boundary or a nontargeting control shRNA. The former pool was clonally derived through limiting dilution to ensure the purity of cells harboring the *PAX3-FOXO1* shRNA. The resultant cells (herein referred to as P3F^KD^) or Rh30 cells carrying a nontargeting control shRNA (referred to here as P3F^+^) were then transduced with the dCas9-KRAB transcriptional repressor. We confirmed P3F^KD^ cells had decreased levels of the fusion protein, as well as downstream transcriptional targets of *PAX3-FOXO1* such as *FGFR4*, when compared with P3F^+^ controls (Figure 1A; see complete unedited blots in the supplemental material). P3F^KD^ cells grow in 2D culture but with a doubling time almost twice that of P3F^+^ cells. Furthermore, P3F^KD^ cells showed near-complete loss of colony formation in soft agar assays, demonstrating a loss of oncogenic potential (Figure 1B).

We then transduced P3F^+^ and P3F^KD^ cells with a pooled library of approximately 20,000 lentiviral sgRNA constructs targeting 2944 genes involved in protein homeostasis, on the basis of our prior work identifying targets in this cellular network as therapeutic opportunities for RMS (17). After growing cells for 10 population doublings, we collected and deep sequenced genomic DNA to identify the abundance of each sgRNA, and we calculated gene-level scores for enrichment or depletion in the P3F^+^ and P3F^KD^ conditions (Figure 1C and Supplemental Table 1; supplemental material available online with this article; https://doi.org/10.1172/jci.insight.162207DS1) (18). This analysis was corrected for the observed number of population doublings in each condition, thus accounting for the slower growth of P3F^KD^ cells. We focused on genes whose knockdown depleted P3F^+^ cells but was tolerated in P3F^KD^ cells, because these should be selectively required in *PAX3-FOXO1*–positive RMS (Figure 1D).

Of the 2944 genes in the screening library, 36 met the following criteria for future study: fold-depletion of greater than 2^–2^ in P3F^+^ cells with an FDR-adjusted Mann-Whitney *q* value of less than 0.20, and a “neutral” fold-change between 2^2^ and 2^–2^ in P3F^KD^ cells (Figure 1E and Supplemental Figure 1). Comparing these results with those of the DepMap project (http://www.depmap.org) (19), more than half of our screen hits (*n* = 20 of 36) were reported as “common essential”; 5 (*USP14, RAB35, PRDX1, FBXO28,* and *KCTD10*) of the 36 screen hits were reported as “strongly selective” but did not meet criteria for lineage enrichment in RMS.

We next asked whether our screen hits showed differential effects when knocked down in 6 FP vs. 5 FN RMS cell lines included in the DepMap. Only 2 of the 36 hits (*UCHL5* and *EIF2B5*) had significantly more potent effects in FP RMS, by a Mann-Whitney test. Although possibly reflective of dependencies unique to the Rh30 cell line, this comparison spurred us to test whether the isogenic *PAX3-FOXO1* screen identified fusion-specific dependencies that did not emerge from a larger, less focused effort.

We next prioritized genes for further validation by using STRING analysis (20) to identify structurally interacting gene products among screen hits (Figure 1E). Four clusters of interacting genes were identified, including a 6-gene cluster encompassing ER to Golgi transport, 2 genes involved in ubiquitin processing at the proteasome, 2 genes involved in pre-mRNA splicing, and 2 members of the GATOR2 complex, *MIOS* and *WDR24*. GATOR2 activates mTORC1 in response to aa (Figure 1F; ref. 6), making it of specific interest given clinical trials supporting the use of mTOR inhibitors in RMS (7). To our knowledge, the GATOR2 complex has not previously been implicated in sarcoma cell signaling or growth. The other 3 GATOR2 complex members had varying results in our screen: *SEH1L* was not included in the library; *WDR59* was depleted in both P3F^KD^ and P3F^+^ conditions, but without meeting statistical significance in the latter; and *SEC13* was strongly and significantly depleted in both conditions, consistent with its GATOR2-independent role in ER vesicular biogenesis (21) (Supplemental Table 1).

### GATOR2 is necessary for proliferation in PAX3-FOXO1–positive RMS cells.

**Figure 2:**
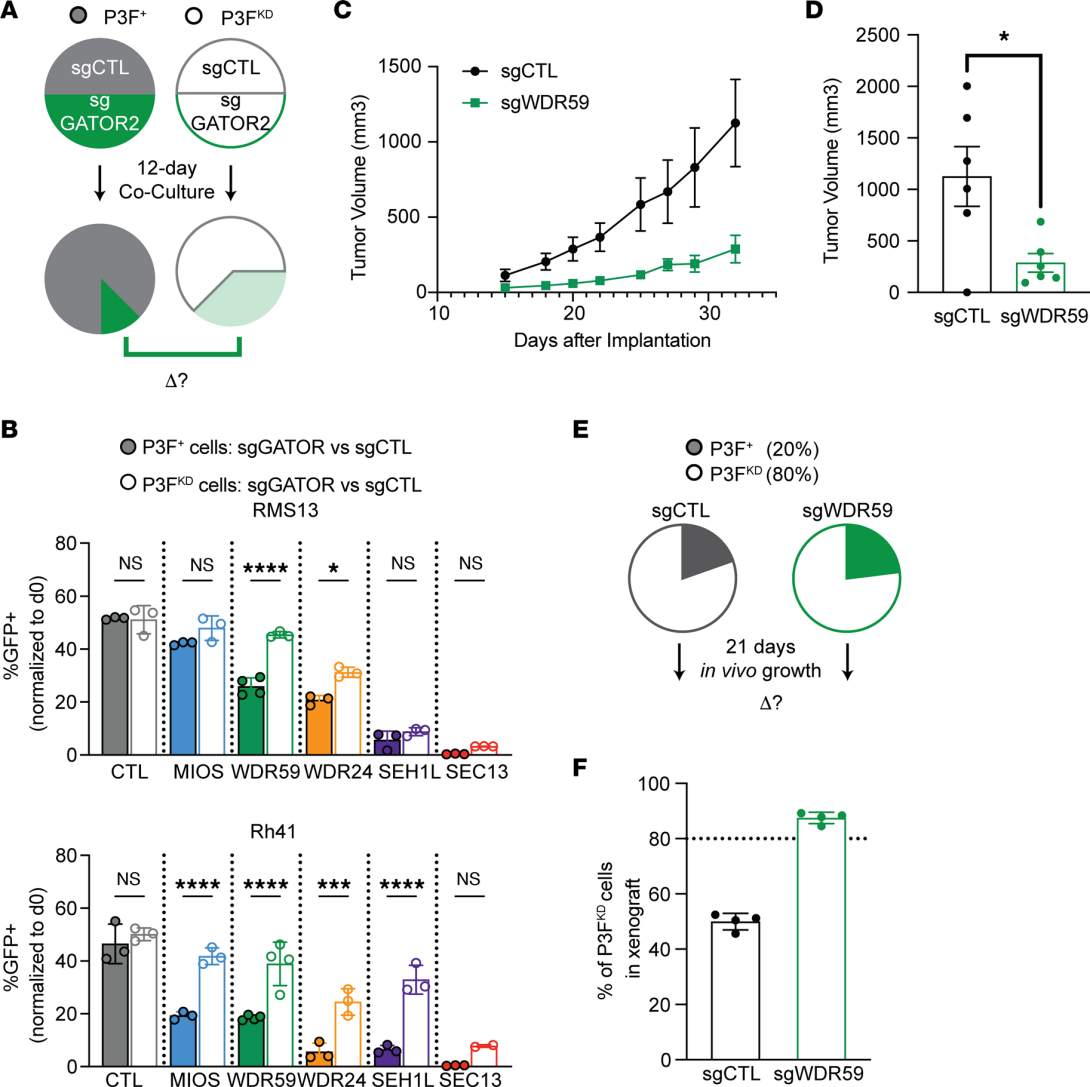
Reducing *PAX3-FOXO1* dosage decreases GATOR2 dependence in RMS. (**A**) Schematic of in vitro competitive fitness assays. Equal numbers of differentially labeled cells were plated after puromycin selection, then maintained in puromycin for 12 days of growth. Separate competitions were carried out to assess the effects of GATOR2 loss in P3F^+^ cells (filled circles) or P3F^KD^ cells (open circles). (**B**) FP cell lines RMS13 and Rh41 show suppressed growth after GATOR2 knockdown, compared with the control (filled bars), assessed by 1-way ANOVA and post hoc Dunnett’s test. The experiment was repeated with cells that harbored combined knockdown of GATOR2 or control by sgRNA and knockdown of *PAX3-FOXO1* by shRNA (open bars). Loss of PAX3-FOXO1 partially rescued cells from GATOR2 knockdown, assessed by 2-way ANOVA and post hoc Sidak’s test comparing the effects of GATOR2 knockdown between P3F^+^ and P3F^KD^ cells. (**C**) NSG mice were implanted with 2 × 10^6^ Rh30 cells transduced with sgRNA targeting *WDR59* (green) or the nontargeting control (gray) after puromycin selection for 7 days and monitored for tumor development. Rates of tumor growth are shown (*n* = 6 mice per condition). (**D**) Tumor volumes after 32 days of growth at time of euthanasia compared by a 2-tailed *t* test**.** (**E**) Schematic of in vivo competitions. A 4:1 excess of P3F^KD^ cells to P3F^+^ cells, transduced with either nontargeting sgRNA (gray) or sgWDR59 (green), was implanted in NSG mice. (**F**) Relative compositions of sgCTL (gray) or sgWDR59 (green) tumors after 21 days of in vivo growth. Δ, change.*P < 0.05; ***P < 0.001; ****P < 0.0001.

To extend these findings, we evaluated the role of each of the 5 members of the GATOR2 complex (*MIOS, WDR59, WDR24, SEH1L,* and *SEC13*) in 2 additional FP RMS cell lines: Rh41 and RMS13. To test the effect of *PAX3-FOXO1* dosage on GATOR2 dependence, we conducted independent competition assays (22–24) in either parental (P3F^+^) or knockdown (P3F^KD^) cells. Cells expressing dCas9-KRAB were transduced with a blue fluorescent protein–tagged (BFP-tagged) control sgRNA and GFP-tagged sgRNAs targeting GATOR2 or nontargeting control (Figure 2, A and B; P3F^+^ cells are indicated by filled circles). In parallel, cells were transduced with bicistronic vectors, including shRNA targeting the *PAX3-FOXO1* fusion and either BFP-tagged control sgRNA, GFP-tagged GATOR2, or control sgRNA (Figure 2, A and B; P3F^KD^ cells are indicated by open circles). Equal numbers of BFP- and GFP-positive cells were plated, and the relative abundance of GFP-positive cells after 12 days in culture was quantified. Competing P3F^+^ cells against P3F^KD^ cells confirmed that knockdown of *PAX3-FOXO1* impaired the growth of all FP cell lines (Supplemental Figure 2A).

Knockdown of GATOR2 decreased the competitive fitness of all cells, regardless of *PAX3-FOXO1* level. However, *PAX3-FOXO1* knockdown had a protective effect against GATOR2 loss in each of the cell lines tested (Figure 2B and Supplemental Figure 2, B–D; filled circles vs. open circles; 2-way ANOVA *P* < 0.0001 for effects of shPAX3-FOXO1, GATOR2, and interaction; adjusted *P* values are indicated for pairwise comparisons by Sidak’s multiple comparisons test). Direct cell-counting assays confirmed that loss of *PAX3-FOXO1* diminished the effects of GATOR2 loss (Supplemental Figure 2E). We conclude that GATOR2 loss more effectively impedes the growth of *PAX3-FOXO1*–positive cells than those in which the fusion protein, and resultant cellular suppression, have been suppressed.

Tissue culture provides an overabundance of aa and thus might incorrectly measure the effects of the aa-responsive GATOR2 complex on cellular growth. Therefore, we turned to murine xenograft models. Rh30 cells were transduced with either sgRNA targeting *WDR59* or a nontargeting control and implanted in the flanks of NSG mice. Tumor formation and growth were monitored over time and confirmed that cells transduced with sgWDR59 had reduced growth compared with cells transduced with a non-targeting control sgRNA (sgCTL; Figure 2, C and D). Thus, GATOR2 promotes both in vivo and in vitro growth of FP RMS. We next used an in vivo competition assay (25) to assess the contribution of *PAX3-FOXO1* to GATOR2 dependence. Knockdown of *PAX3-FOXO1* was selected against tumor cells in the absence of puromycin selection that could not be maintained in vivo, so we used this experimental design to benchmark the fitness of cells that may have slowly increasing *PAX3-FOXO1* expression over time against the parental cell line. First, we confirmed that *PAX3-FOXO1* is required for the fitness of Rh30 cells in vivo. A 4-fold excess of GFP tagged P3F^KD^ cells was mixed with BFP-tagged P3F^+^ cells and implanted in the flanks of NSG mice. After 21 days of growth, animals were euthanized and the resultant tumors were analyzed by flow cytometry and immunoblot. Consistent with our in vitro findings and a myoblast-derived in vivo model (26), the 4-fold excess of P3F^KD^ cells was overtaken by P3F^+^ cells during that time (Figure 2, E and F).

Finally, we asked whether suppression of GATOR2 might favor the slowly proliferating P3F^KD^ cells by depriving P3F^+^ cells of mTORC1 signaling. We repeated the experiment using a 4-fold excess of GFP-tagged P3F^KD^/sgWDR59 cells that competed against BFP-tagged P3F^+^/sgWDR59 cells. In contrast to the *WDR59*-sufficient tumors, the majority of cells in the *WDR59* knockdown tumors were derived from the P3F^KD^ xenograft (Figure 2, E and F). These data support the hypothesis that loss of GATOR2 diminishes the growth of *PAX3-FOXO1*–positive RMS in vivo.

### GATOR2 regulates mTORC1 activation and cellular proliferation in FP RMS.

**Figure 3:**
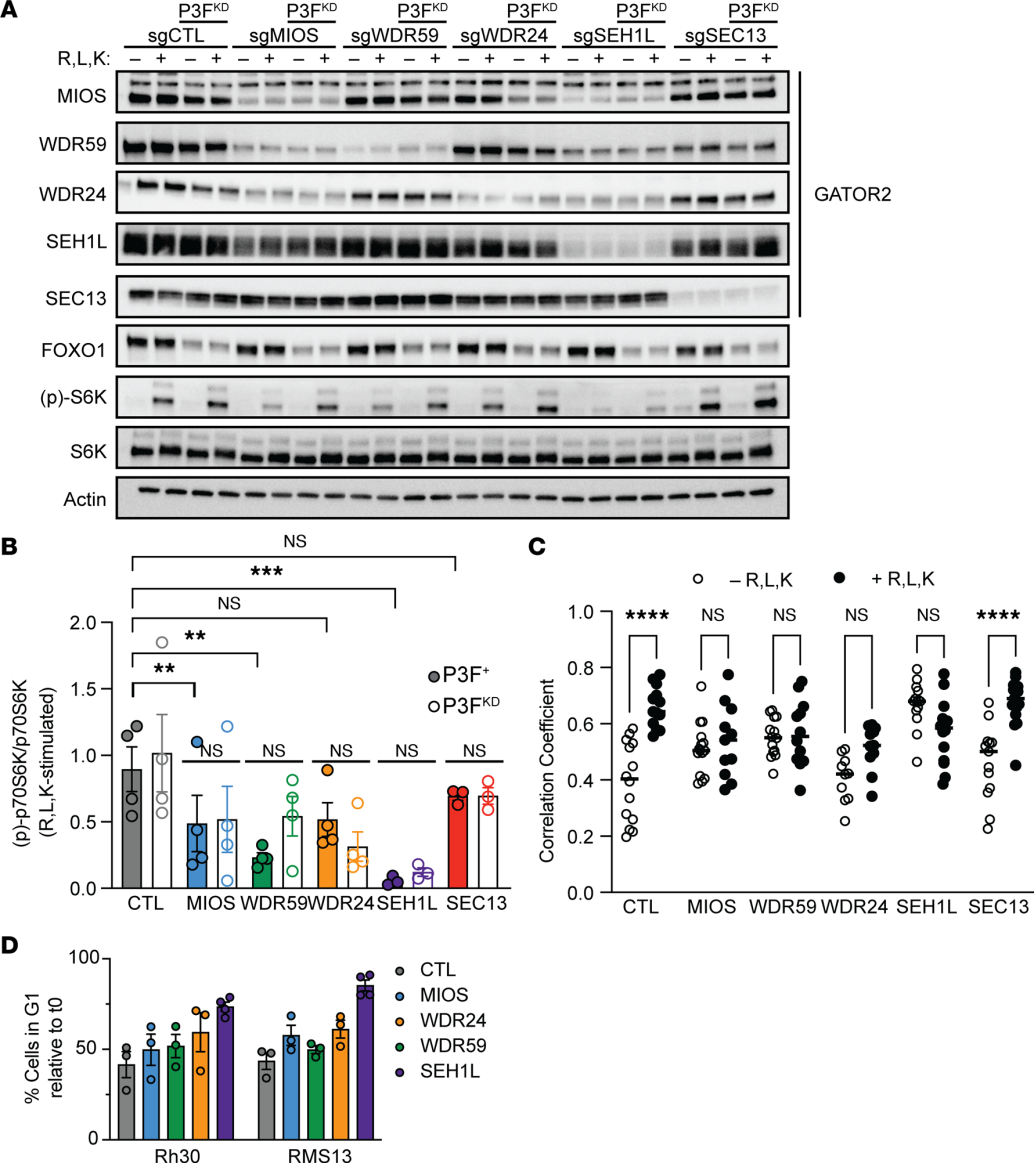
Loss of GATOR2 abrogates mTORC1 activation and slows cell cycle progression in RMS. (**A**) RMS13 cells transduced with the indicated sgRNA or shRNA plus sgRNA combination were seeded and then grown in serum-free RPMI-1640 lacking arginine, leucine, and lysine for 3 hours. Cells were either lysed directly (–) or after 10 minutes of stimulation with aa-replete medium (+). Representative immunoblot demonstrates the effects of GATOR2 knockdown and/or *PAX3-FOXO1* knockdown on mTORC1 signaling. (**B**) Quantitation of p70S6K phosphorylation in response to aa stimulation from replicates of immunoblot, as in **A**. Significance was assessed by 1-way ANOVA and Dunnett’s multiple comparisons test (comparing GATOR2 knockdown to control) or Sidak’s multiple comparisons test (comparing P3F^+^ to P3F^KD^ cells within each genotype). (**C**) Quantification of correlation coefficients for mTOR and LAMP2 colocalization by structured illumination microscopy of aa-starved cells fixed immediately or after 10 minutes of stimulation with full RPMI-1640 (*n* = 10–15 cells imaged per condition). (**D**) GATOR2 knockdown cells were subjected to a single thymidine block for 18 hours, then washed and plated in full medium for 8 hours prior to fixation and permeabilization. Percentage of cells in G1 was calculated by propidium iodide staining and analysis on a flow cytometer. CTL, control.**P < 0.01; ***P < 0.001; ****P < 0.0001.

The GATOR2 complex inhibits the GATOR1 GTPase activating protein complex, thus enabling RAG-GTP/GDP heterodimers to recruit mTORC1 to the lysosomal membrane in response to aa abundance (6). We examined whether this pathway operates as previously described here in the context of FP RMS and whether loss of *PAX3-FOXO1* alters this signaling cascade. Rh30 cells were deprived of leucine, lysine, and arginine for 3 hours and either lysed immediately or after 10 minutes of stimulation with aa-replete medium. As expected, cells transduced with control sgRNA had robust phosphorylation of p70S6K after aa stimulation, indicative of mTORC1 activation. By contrast, knockdown of most GATOR2 components attenuated p70S6K phosphorylation, indicating impaired transmission of aa sufficiency signals to mTORC1 (Figure 3, A and B; adjusted *P* values by 2-way ANOVA with Dunnett’s multiple comparisons test). Similarly, knockdown of most GATOR2 complex members prevented the localization of mTOR to the lysosome by widefield deconvolution microscopy, confirming that GATOR2 regulates mTORC1 in RMS through this previously described mechanism (Figure 3C and Supplemental Figure 3A). *SEC13* was an exception to both findings: knockdown of *SEC13* did not prevent phosphorylation of p70S6K or localization of mTOR to the lysosome upon aa stimulation. Because *SEC13* loss does not perturb GATOR2 function in RMS and was equally depleted in P3F^+^ and P3F^KD^ cells in our screen, we excluded it from further study of the PAX3-FOXO1 and GATOR2 interaction. We found that knockdown of *PAX3-FOXO1* did not significantly affect aa stimulation of mTORC1 (*P* = 0.13 by 2-way ANOVA), nor did knockdown of GATOR2 alter the expression of *PAX3-FOXO1*. Thus, the protective effect of *PAX3-FOXO1* knockdown seen in competition assays is not due changes in aa control of mTORC1 signaling; instead, it may reflect a decreased requirement for mTORC1 activation in P3F^KD^ cells.

We next investigated why GATOR2-deficient cells are lost over time in competition assays. We did not detect significant apoptosis as measured by annexin V and propidium iodide staining (Supplemental Figure 3B). Because mTORC1 is essential for cell cycle progression (27), we interrogated whether GATOR2 loss slowed cell cycle transit in FP RMS. We assessed G1-S progression by blocking cells in G1 with thymidine and releasing into nocodazole. After thymidine block, cells demonstrated slowed transition from G1 into S phase after GATOR2 knockdown (Figure 3D and Supplemental Figure 3C). Thus, GATOR2 loss, like pharmacological inhibition of mTORC1 (9), results in cytostatic rather than cytotoxic effects through slowed G1-S cell cycle progression.

### Activation of mTORC1 is sufficient to compensate for loss of MIOS and WDR59.

**Figure 4:**
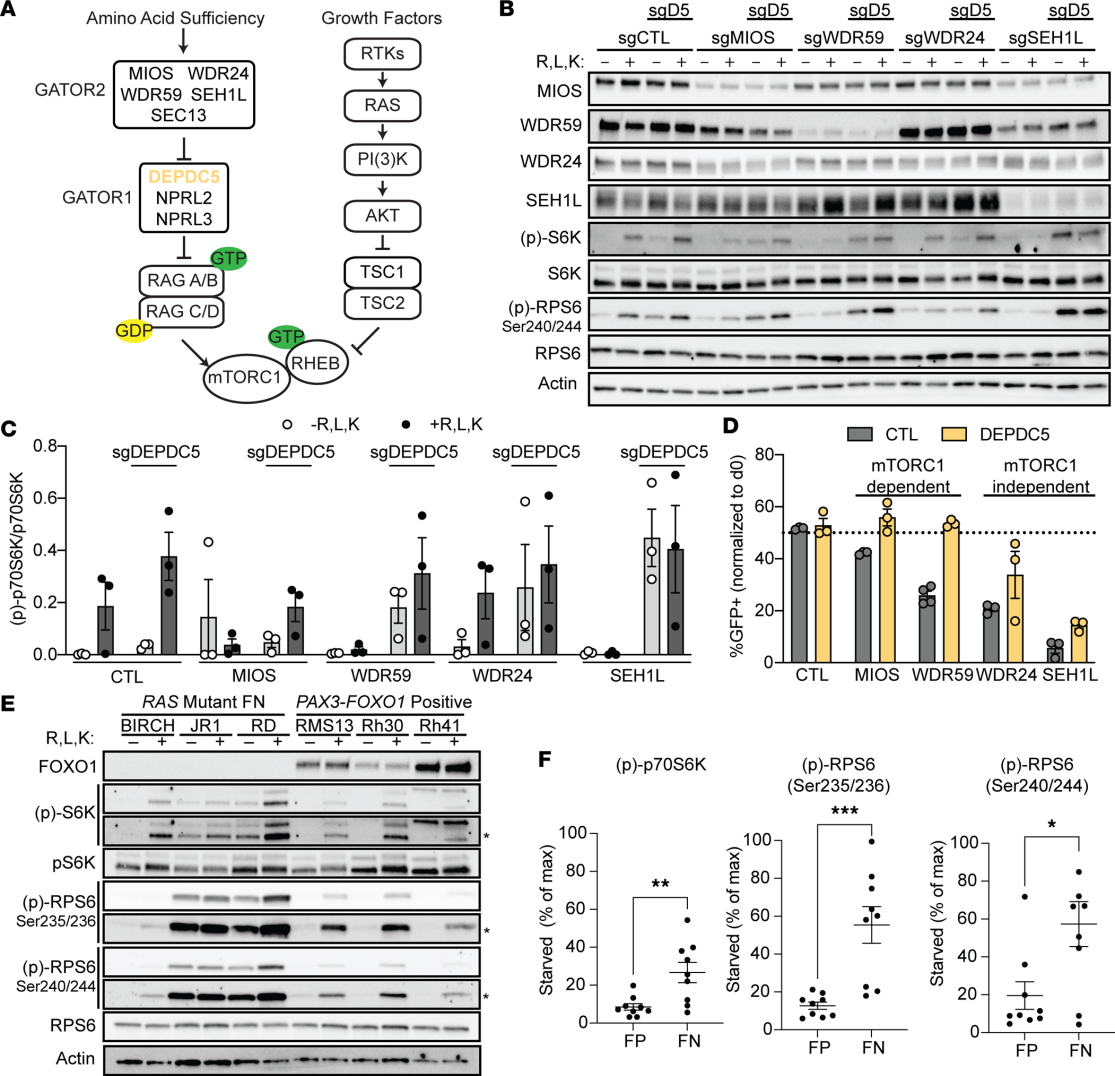
Activation of mTORC1 overcomes GATOR2 dependence. (**A**) Schematic of nutrient-sensing and mitogen pathways that converge on mTORC1 activation. (**B**) RMS13 cells were transduced with the indicated sgRNA, and mTORC1 activity was assessed by immunoblot after aa starvation and stimulation. (**C**) Quantification of levels phosphorylated p70S6K normalized to total p70S6K (see Supplemental Figure 4 for 2 additional replicates and quantification of m7-GTP binding by 4EBP1). Differences in aa-stimulated levels of p70S6K phosphorylation across *sgDEPDC5* conditions were NS by 1-way ANOVA. (**D**) Competition assays confirmed that reactivation of mTORC1 can rescue cell growth after knockdown of *MIOS* or *WDR59*, but not *WDR24*, *SEH1L*, or *SEC13*. One-way ANOVA with post hoc Dunnett’s test was significant for differences between sgDEDPC5 and sgDEPDC5-WDR24 (*P* = 0.0271) and sgDEPDC5-SEH1L (*P* < 0.0001), and was NS for others. (**E**) Immunoblot of aa-starved and -stimulated *RAS-*mutant FN (BIRCH, JR1, RD) or *PAX3-FOXO1* FP (RMS13, Rh30, Rh41) cells shows increased basal mTORC1 activity in the former; representative immunoblot from 3 independent replicates. Asterisks indicate long exposures of the same blots. (**F**) Quantitation of phosphorylation of p70S6K and RPS6 under aa starvation conditions in FP and FN RMS cells. Significance of differences assessed by 2-tailed *t* test. CTL, control; max, maximum. *P < 0.05; **P < 0.01; ***P < 0.001.

Although we have demonstrated that *MIOS, WDR59, WDR24,* and *SEH1L* are each required for mTORC1 activation, these proteins also have distinct, mTOR-independent roles in cell biology (28–30). Therefore, we considered whether our findings of increased GATOR2 dependence represent a requirement for mTORC1 signaling or, instead, highlight distinct, mTOR-independent genetic dependencies in FP RMS. We reasoned that if any given GATOR2 member is specifically required to activate mTORC1, then direct activation of mTORC1 should rescue cells from its loss. To test this hypothesis, we activated mTORC1 downstream of GATOR2 (Figure 4A) through knockdown of *DEPDC5*, a member of the GATOR1 complex that GATOR2 inhibits.

Dual knockdown of GATOR2 and *DEPDC5* rescued p70S6K phosphorylation (Figure 4, B and C) and 4EBP1 release from the m^7^-GTP cap (Supplemental Figure 4). In competition assays, knockdown of *DEDPC5* rescued cells from *MIOS* or *WDR59* loss (Figure 4D) but only partially rescued *WDR24* or *SEH1L* loss (differences assessed by 1-way ANOVA with post hoc Dunnett’s test) when compared with BFP-tagged sgCTL cells. Thus, the GATOR2 complex can be segregated into members with entirely mTOR-dependent (*MIOS, WDR59*) and partially mTOR-independent (*WDR24, SEH1L*, and as shown above, *SEC13*) effects on proliferation in FP RMS. Ongoing efforts to elucidate the molecular function of each component as it acts within the GATOR2 complex may help clarify these differences (31–34).

We next examined mTORC1 regulation in FN RMS as a comparison with the aa dependence exhibited by FP RMS. FN RMS shares histologic features with FP RMS and yet has distinct genetics and superior cure rates. These cells lack *FOXO1* fusions; instead, they harbor oncogenic mutations in the RAS-MAP kinase and PI3-kinase pathways (15, 16) that might activate mTORC1 in parallel to aa signaling (Figure 4A) (35, 36). FN RMS cells maintain higher levels of p70S6K and RPS6 phosphorylation in the absence of aa (Figure 4, E and F). Correspondingly, these FN cell lines have no decreased fitness upon loss of the mTOR-dependent GATOR2 complex members *MIOS* and *WDR59*, but they retain sensitivity to the partially mTOR-independent members *WDR24*, *SEH1L*, and *SEC13* (Supplemental Figure 5). Exogenous expression of *PAX3-FOXO1* was insufficient to enhance dependence on *MIOS* or *WDR59* (Supplemental Figure 5). These data raise the possibility that oncogenic *RAS* mutation drives the decreased sensitivity of mTORC1 to aa deprivation or GATOR2 loss observed in these FN RMS cell lines.

### Oncogenic RAS mutations diminish the role of GATOR2 in mTORC1 activation or growth.

**Figure 5:**
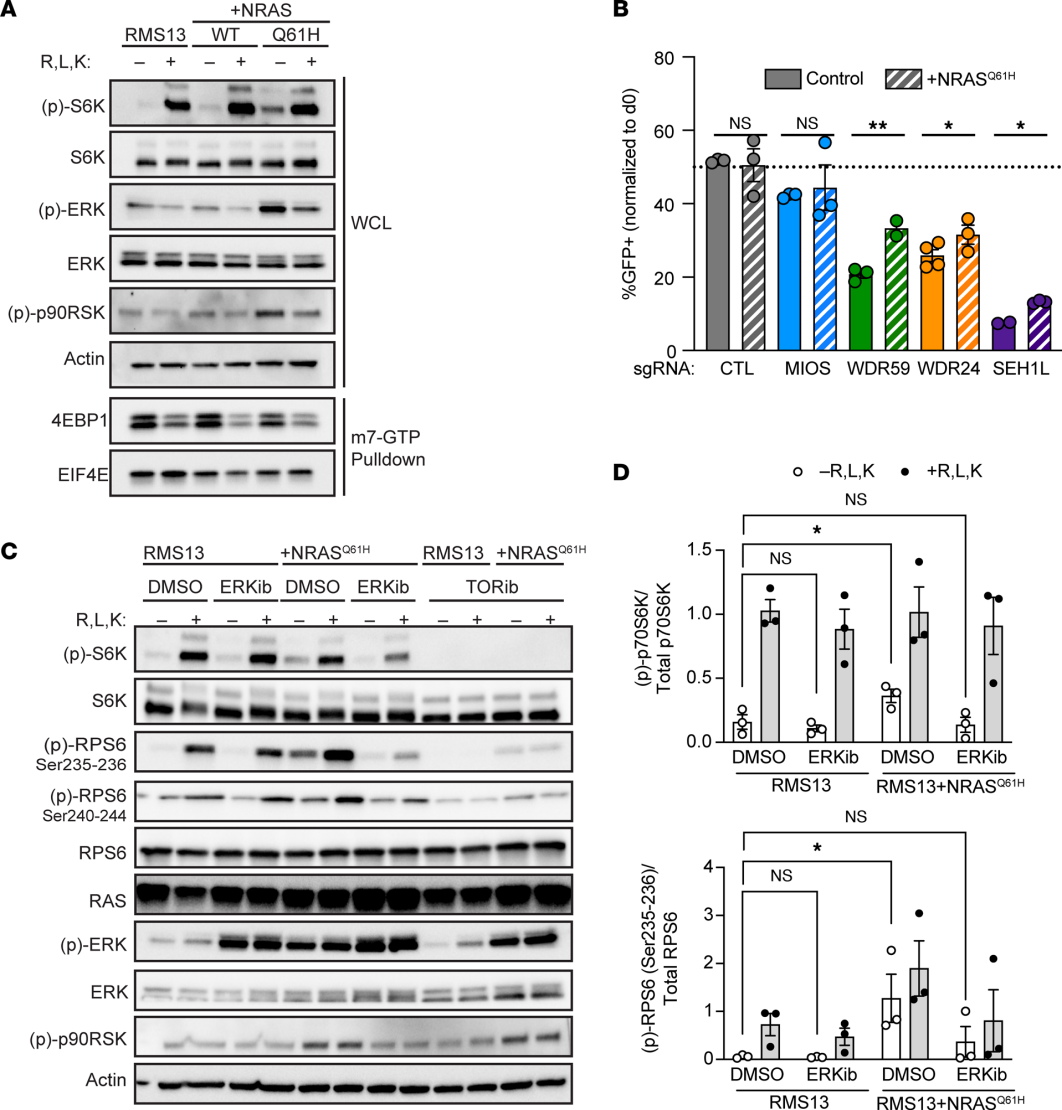
MAP kinase activation downstream of oncogenic *RAS* is necessary and sufficient for aa-independent activation of mTORC1 in RMS. (**A**) *PAX3-FOXO1*–positive RMS13 cells transduced with WT or mutant *NRAS* were subjected to aa starvation and stimulation. Representative immunoblot from 3 replicates of lysates and m7-GTP pulldowns demonstrates that *NRAS^Q61H^* is sufficient to drive aa-dependent phosphorylation of p70S6K and RPS6, but not of 4EBP1. (**B**) Competition assays in *NRAS^Q61H^* expressing RMS13 cells compared with parental cells from Figure 2B; differences between parental and *NRAS^Q61H^* expressing cells assessed by 2-tailed *t* test. (**C**) RMS13 or RMS13 cells expressing *NRAS^Q61H^* were incubated with DMSO, 1 μM ulixertinib (ERKib), or 100 nM sapanisertib (TORib) for the duration of a 3-hour aa starvation with or without 10-minute stimulation. Immunoblots demonstrate that sapanisertib suppresses p70S6K phosphorylation in either condition but incompletely suppresses RPS6 phosphorylation. By contrast, ulixertinib restores aa control of RPS6 phosphorylation. (**D**) Quantification of p70S6K and RPS6 phosphorylation from **C** (*n* = 3 independent replicates). Significance of basal p70S6K or RPS6 phosphorylation assessed by 1-way ANOVA and Dunnett’s multiple comparisons test. CTL, control. *P < 0.05; **P < 0.01.

To determine whether oncogenic *RAS* is sufficient to reduce aa thresholds for mTORC1 activity, we expressed either WT or Q61H mutant alleles of *NRAS* (the most commonly mutated codon and *RAS* isoform in FN RMS; ref. 2), in FP RMS13 cells (Figure 5A). *NRAS^Q61H^*, but not WT *NRAS*, bolstered basal levels of p70S6K and RPS6 phosphorylation (but not 4EBP1 release from eIF4E). Like *DEPDC5* inactivation, expression of oncogenic *NRAS^Q61H^* rescued FP cells from GATOR2 loss in growth assays (Figure 5B), though with more modest results.

We next asked whether MAP kinase activity is required for the observed mTORC1 signaling in *NRAS*-mutant RMS in aa-limiting conditions. In FP RMS13 cells, blocking MAP kinase output with the ERK inhibitor ulixertinib had minimal effects on p70S6K phosphorylation or RPS6 phosphorylation in response to aa. In cells harboring an *NRAS^Q61H^* mutation, however, ERK inhibition blocked both basal and aa--stimulated phosphorylation of p70S6K and RPS6 (Figure 5, C and D, and Supplemental Figure 6). The same effects were seen with the MEK inhibitor trametinib, and in BIRCH cells with endogenous *HRAS* mutation (Supplemental Figure 6). These data suggest that MEK-ERK activity downstream of oncogenic *RAS* mutations is necessary and sufficient for aa-independent phosphorylation of p70S6K. Recent work has shown that oncogenic RAS can confer RAG-independent activation of mTORC1 in response to select aa (37). However, FN RD cells transduced with a dominant negative RAG construct (38) lost aa-responsive p70S6K phosphorylation (Supplemental Figure 7). Thus, we conclude that although maximal mTORC1 activity in *RAS*-mutant RMS requires intact ERK and RAG signaling, hyperactive ERK can sustain mTORC1 in aa-limiting conditions.

### Bisteric mTORC1 inhibition induces regressions in xenografts derived from patients with FP RMS.

Our genetic findings demonstrate that mTORC1 signaling is central for FP RMS growth and that its activity is under tight homeostatic control by nutrient availability. The allosteric mTOR inhibitor temsirolimus can improve event-free survival in patients with RMS who have relapsed disease, but temsirolimus is insufficient to cure the disease even when combined with chemotherapy. On the basis of the data presented here, we hypothesized that mTORC1 is, in fact, a critical vulnerability in FP RMS but that the pharmacological means previously used to exploit this vulnerability may have been insufficient to provide maximal benefit. mTORC1-selective inhibitors with distinct modes of action from rapalogs (13) are now in clinical development. Bisteric inhibitors combine allosteric binding with an ATP-competitive inhibitory mechanism (13) and more durably suppress mTORC1 outputs, including phosphorylation of 4EBP1, than do allosteric inhibitors in models of glioma (9). Therefore, we investigated whether RMC-6272, a preclinical tool compound with properties representative of the clinical-stage bisteric mTORC1 inhibitor RMC-5552, currently in phase I testing, more completely suppresses mTORC1 activity and thus more effectively targets mTORC1 dependence in FP RMS.

**Figure 6:**
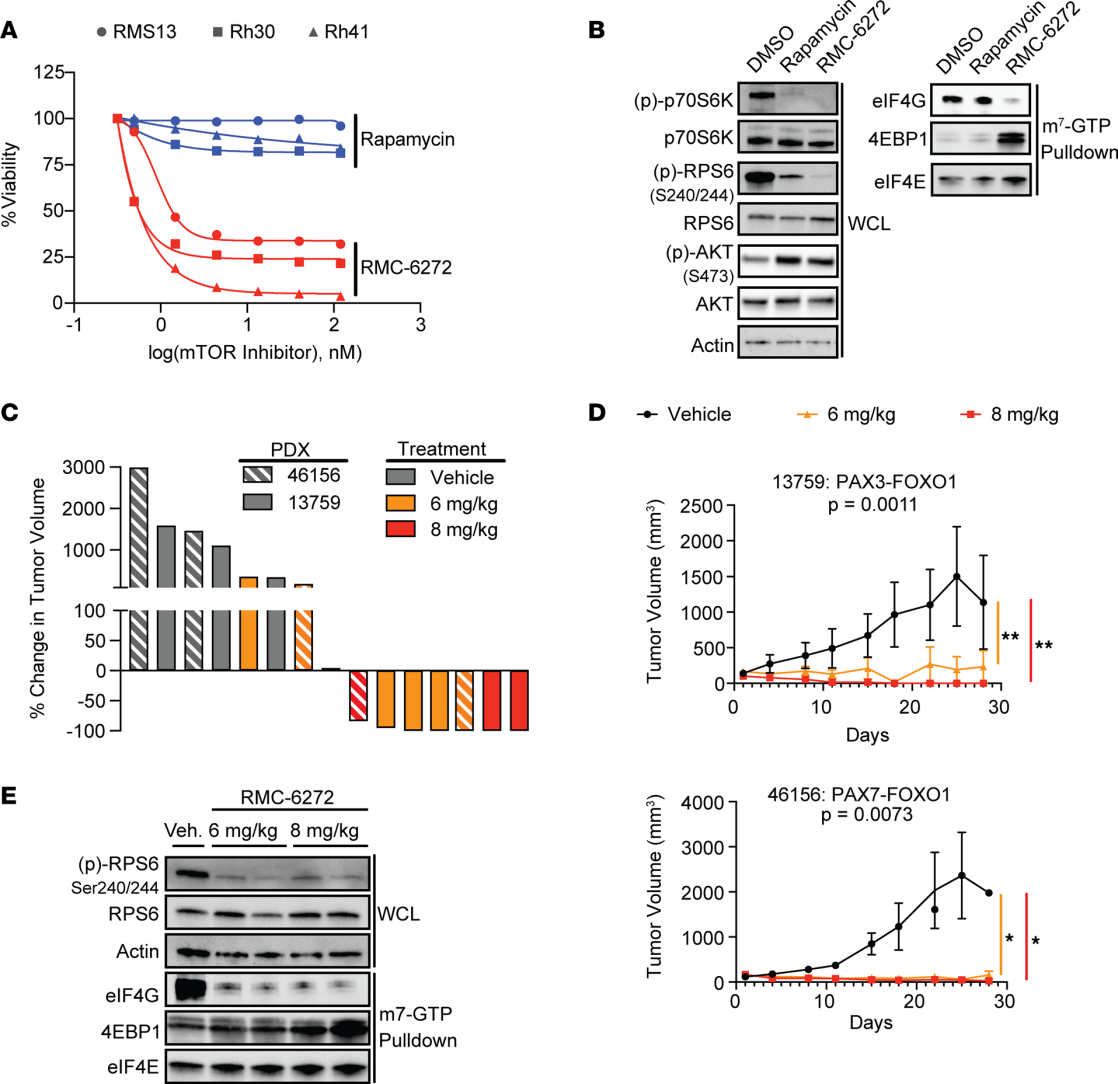
The bisteric mTORC1 inhibitor RMC-6272 induces complete remission in FP RMS PDXs. (**A**) FP RMS cell lines were seeded in 96-well plates and treated across a dose range of the allosteric mTOR inhibitor rapamycin and RMC-6272. After 6 days, viability was measured by alamarBlue assay. (**B**) Representative immunoblot from 3 independent replicates of RMS13 cells treated with 1 nM rapamycin or RMC-6272 shows similar dephosphorylation of p70S6K but poor suppression of cap-dependent translation by rapamycin, as measured by competitive binding of EIF4G and 4EBP1 to EIF4E in an m7-GTP pulldown assay. (**C**) Waterfall plot demonstrating tumor response after 28 days based on PDX and drug treatment. (**D**) Tumor growth curves of 2 FP RMS PDXs treated with RMC-6272 at the indicated doses. Differences between vehicle (Veh) and drug treatment were measured by a linear mixed-effects regression model with Dunnett’s multiple comparisons test. (**E**) Mice harboring the 13759 PDX were euthanized 24 hours after administration of vehicle or the indicated doses of RMC-6272. Immunoblot of whole-cell lysates (WCLs) and m7-GTP pulldowns from excised, flash-frozen PDX demonstrates mTORC1 inhibition with RMC-6272. *P < 0.05; **P < 0.01.

In vitro, RMC-6272 was significantly more potent than the allosteric inhibitor rapamycin (of which temsirolimus is a prodrug) (Figure 6A). Although both agents prevented p70S6K phosphorylation and increased phosphorylation of the mTORC2 target AKT at Ser473 (13), RMC-6272 induced dissociation of 4EBP1 from EIF4E, suppressing cap-dependent translation, whereas rapamycin did not (Figure 6B and Supplemental Figure 8). On the basis of these data, we tested the preclinical activity of RMC-6272 in 2 xenografts derived from patients (PDXs) with FP RMS disease. Flanks of NSG mice were implanted with 1 × 10^6^ viable cells in 50% MatriGel, and animals were randomized to either RMC-6272 or vehicle treatment once tumors reached a volume of 100 mm^3^. RMC-6272 was dosed by weekly i.p. injection, either at 6 or 8 mg/kg. At both doses, RMC-6272 demonstrated significant tumor control in these models (Figure 6, C and D). Of 10 animals treated, we observed 5 complete responses to therapy (67% at 8 mg/kg and 43% at 6 mg/kg), and 7 with a best response of stable disease or better (100% at 8 mg/kg, and 71% at 6 mg/kg). By contrast, although temsirolimus treatment (20 mg/kg i.p. twice a week) (39) led to dephosphorylation of RPS6, it only induced regression in 1 of 8 tumors (–10% volume) and had no effect on tumor growth in 1 of the 2 models tested (Supplemental Figure 9). Treatment with RMC-6272 suppressed both RPS6 phosphorylation and EIF4G binding to EIF4E in treated tumors, confirming effective pharmacodynamic modulation of the target pathway and consistent with in vitro data (Figure 6E). Three of 5 animals treated at 8 mg/kg had weight loss of greater than 10%, compared with none of the animals treated at 6 mg/kg. Indeed, mice treated at 6 mg/kg did not exhibit any significant weight loss compared with vehicle treatment (Supplemental Figure 10; *P* = 0.16 by 2-tailed *t* test), indicating this dose to be the maximum tolerated dose in NSG mice. Together, these data demonstrate that a bisteric mTORC1 inhibitor induces regressions in FP RMS PDX models and highlight 4EBP1 binding to EIF4E as a biomarker of treatment response.

## Discussion

*FOXO1* fusions are a consistent adverse prognostic factor for patients with RMS (3). Unlike kinase fusions present in other sarcomas involving *NTRK3* or *ALK*, however, *FOXO1* fusions lack an enzymatic, readily druggable activity. Novel technologies such as protein degraders may eventually permit direct targeting of nonenzymatic oncoproteins. As a complementary and alternative approach, here we used a functional genomics screen to identify a liability within the aa regulation of protein synthesis. Importantly, our isogenic system identified this vulnerability, alongside others, that could not be specifically linked to the *PAX3-FOXO1* oncogene in important, larger-scale efforts. One limitation of our approach is that although our screen suggested that GATOR2 is completely dispensable in *PAX3-FOXO1* knockdown cells, more detailed mechanistic studies instead unveiled a dosage response of enhanced sensitivity with higher *PAX3-FOXO1* expression. Additionally, different GATOR2 members exhibited differing biochemical, cellular, and genetic interaction phenotypes, perhaps due to distinct contributions to the function of the GATOR2 complex. Although the relationship between *PAX3-FOXO1* and GATOR2 is thus less stark and more nuanced than one of synthetic lethality, the differing sensitivity of *PAX3-FOXO1* intact and knockdown cells to GATOR2 knockdown is shown here to have a potential therapeutic impact. Another limitation of our study was gene-by-gene validation, which has left the relationship between *PAX3-FOXO1* and the other 34 screen hits awaiting additional study. A pooled rescreening strategy in additional FP cell lines may reduce this bottleneck in larger screens. The development and refinement of such isogenic systems may thus further uncover important oncogene dependencies with clinical ramifications.

Although nutrient control of mTORC1 is a conserved regulatory mechanism, we found that 2 RMS oncogenes, *PAX3-FOXO1* and *NRAS^Q61H^*, differently affected the impact of GATOR2 on mTORC1 output. Reduced *PAX3-FOXO1* levels diminish the cell biological effects of GATOR2 loss. It is possible that aa thresholds for mTORC1 activation are higher in PAX3-FOXO1–positive cells relative to knockdown cells, which our aa starvation and stimulation experiments may not reveal. Alternatively, or in addition, it may be that *PAX3-FOXO1*–positive cells have higher demands on protein synthesis than their knockdown counterparts, opening a potential therapeutic window. A third possibility is that there is a subset of PAX3-FOXO1 target transcripts that exhibit mTOR-dependent translation, defining an important level of control on fusion oncoprotein output. Unbiased approaches such as ribosome profiling or quantitative nascent peptide mass spectrometry may help distinguish among these possibilities. Broadly, we propose that cells transformed by oncogenes that redefine epigenetic states (e.g., *PAX3-FOXO1*) rather than those that directly activate signaling cascades (e.g., *NRAS^Q61H^*) retain homeostatic buffers that control protein translation. Thus, *PAX3-FOXO1* cannot overcome a strict requirement for aa activation that oncogenic *RAS* is able to circumvent. How other driver mutations in FN RMS, such as *PIK3CA* or *FGFR4* activation, alter mTORC1 signaling remains to be studied.

Driven by genetic data supporting mTORC1 dependence in FP RMS, we tested the bisteric mTORC1 inhibitor RMC-6272 in PDX models of this disease. This agent is sufficient to induce complete remissions in RMS preclinical testing, supporting the central importance of mTORC1 in FP RMS despite the more modest effects of allosteric inhibitors (39, 40). We believe the preclinical data presented here provide strong rationale to pursue clinical testing of bisteric inhibitors regardless of the effects seen in ongoing studies with allosteric inhibitors such as temsirolimus. In addition to measuring pharmacokinetics and determining a maximally tolerated dose, response biomarkers to determine optimal biological dosing (41) will be essential to make sense of potentially conflicting data between these 2 classes of inhibitors. Prior studies have confirmed the prognostic impact of multiparameter mTOR pathway activation in RMS, including phosphorylation of both RPS6 and 4EBP1 (42). Our data confirm that dephosphorylation of 4EBP1 is a crucial difference between allosteric and bisteric inhibitors (9, 13). Knockdown of 4EBP1 and dual knockdown of 4EBP1 and 4EBP2 enhanced, but did not completely restore, binding of EIF4G to EIF4E in the presence of RMC-6272 (Supplemental Figure 11A). This, in turn, gave rise to only partial RMC-6272 resistance in vitro (Supplemental Figure 11B), suggesting that 4EBP1 and 4EBP2 are not the sole effectors by which mTORC1 inhibition limits the growth of RMS. Studies will be required to define which mTORC1 targets contribute to the antitumor effects of RMC-6272 in RMS and may have a role in predicting mechanisms of drug resistance. Resistance to bisteric mTORC1 inhibitors may also emerge through selection for cells expressing low levels of *PAX3-FOXO1*, as have been identified in human tumors and murine models (26, 43). Combination therapy approaches such as those already tested with temsirolimus may circumvent this possibility.

In conclusion, the data presented here support the development of isogenic systems to probe genetic networks that regulate the survival of oncogene-driven cancer cells. Such screens can identify oncogene-selective vulnerabilities within intact homeostatic mechanisms, for instance, aa control of protein translation, that can be re-examined as new pharmacological tools emerge with which to target them. The general strategy described in this study offers a means of refining precision therapy that could be useful to develop molecular approaches for the treatment of other cancers with driver oncogenes that are challenging to drug by current methods.

## Methods

### Cell culture and lentiviral transduction.

RMS cell lines were obtained from the COG Repository (Rh30, Rh41), purchased from ATCC (RD, RMS13), or were a gift from Marielle E. Yohe (BIRCH, JR1; National Cancer Institute). RD and JR1 cells were grown in DMEM, and all other cell lines were grown in RPMI-1640, supplemented with 10% FBS and 1× penicillin/streptomycin, in a 37°C incubator with 5% CO_2_. Cells were tested quarterly for mycoplasma and tested to confirm identity by short tandem repeat analysis twice a year.

Either 293T (ATCC) or Lenti-X cells (Takara Bio) were plated in DMEM supplemented with 10% FBS and 1× penicillin/streptomycin and transfected with plasmids of interest, pCMVdR8.91, and pMD2.g using TransIT-LT1 transfection reagent (Mirus) at a 3:1 ratio. Six hours later, ViralBoost reagent (Alstem) was added at 1:500. Seventy-two hours after transfection, viral particles were harvested from the supernatant, filtered through a 0.45-micron polyethersulfone syringe, and then added to target cells with 6 μg/mL polybrene. In 24 hours, selection was started with either puromycin (shRNA or sgRNA constructs) or hygromycin (pLV-EF1-Hygro constructs) for 3 or 10 days, respectively. For PAX3-FOXO1 expression, Lenti-X cells were transfected with pBABE-Neo-PAX3-FOXO1, pCMV-VSV-G, and pUMVC, then retroviral particles were produced and collected as described above. Cells were selected with G418 for 14 days.

### Generation of PAX3-FOXO1 knockdown cells.

Rh30 cells were transduced with shP3F-GFP lentiviral particles, then sorted for GFP expression after 72 hours. GFP-positive cells were seeded at a concentration of 1000 cells/15-cm plate, and after 2 weeks, cloning cylinders were used to isolate and expand single-cell clones in 24-well plates. Resultant clones were analyzed by flow cytometry and immunoblot to identify a cell line with high GFP and low PAX3-FOXO1 expression, which was designated P3F^KD^. These cells were then transduced with lentiviral particles, including the dCas9-KRAB construct, sorted twice for BFP expression, and used in the initial screen.

### CRISPR-interference screen.

Lentiviral particles were generated from 293T cells transduced with pooled sgRNA libraries as described (18), then used to infect either Rh30-P3F^KD^ cells, or Rh30 cells serially transduced with pLMN-GFP and dCas9-KRAB BFP, and sorted for GFP and BFP positivity (Rh30-P3F^+^). Flow cytometry was used to confirm an MOI of approximately 1 at 48 hours after infection. Cells were selected in puromycin for 72 hours, an aliquot was frozen for t_0_ analysis, and the remainder were seeded in 500 cm^2^ tissue culture plates at equal density (8 × 10^6^ cells/plate). Every 3 days, cells were trypsinized, pooled, counted, and then replated at the same density to maintain 1000× coverage of each sgRNA construct. After 10 population doublings, cells were viably frozen.

Deep sequencing and data analysis were performed as described (18). Briefly, genomic DNA was extracted from t_0_ and t_end_ cells using a DNEasy Blood & Tissue kit (Qiagen), digested to enrich for lentiviral integration sites, and sgRNA sequences were amplified by PCR for subsequent sequencing on an Illumina HiSeq. Reads were aligned to the sgRNA library, and fold-change from t_0_ to t_end_ in the P3F^+^ or P3F^KD^ conditions was calculated. A gene-level score was then calculated as the mean of the top 3 scoring sgRNAs targeting a given transcript.

### Flow cytometric competitive fitness and cell-fate assays.

After puromycin selection, equal numbers of cells transduced with either GFP- or BFP-control labeled sgRNA constructs were plated in a single well. A sample of this mixed culture was taken at the time of plating, then every 4 days with trypsinization, for flow cytometric analysis. The percentage of GFP^+^ cells was measured until 12 days in culture, normalizing back to the percentage present when mixed cultures were initiated. For apoptotic assays, cells were allowed to recover from puromycin selection for 48 hours prior to staining with annexin V and propidium iodide and analysis per manufacturer’s instructions (CF488A Annexin V and PI Apoptosis Kit, Biotium). Analysis was restricted to BFP^+^ cells to exclude residual nontransduced cells. For cell cycle assays, cells were plated in 2 μM thymidine for 18 hours after puromycin selection, then washed and released into fresh medium. After 8 hours, cells were trypsinized, fixed in ice-cold ethanol, and stained and analyzed per manufacturer’s instructions (FxCycle PI/RNase staining solution, Thermo Fisher Scientific).

### Immunoblots and m^7-^GTP pulldown.

Cells for immunoblots were lysed in ice cold RIPA buffer with protease and phosphatase inhibitors (Roche). Lysates were quantified using a DC protein assay (Bio-Rad), boiled in 1× Laemmli buffer, and then run on a 4% to 16% TGX gel. Gels were transferred onto nitrocellulose membrane and blotted for 3 to 18 hours in primary Ab, washed 3 times, and then incubated with HRP-conjugated secondary Ab for 1 hour. Blots were washed thrice, then imaged using ECL reagent (Amersham) on a GelDoc (Bio-Rad). Blots are representative of at least 3 independent replicates. Quantitation of phosphorylation was carried out by ImageJ (NIH) and normalized to total protein levels for the p70 band of S6K or RPS6, respectively.

For m7-GTP pulldowns, cells were lysed in 10 mM Tris-HCl (pH 7.6), 140 mM KCl, 4 mM MgCl_2_, 1 mM DTT, and 1 mM EDTA (buffer A) supplemented with 1% NP-40. Lysates were frozen at –70°C overnight, clarified by centrifugation (18,000*g* for 10 minutes), then quantified as described above. We incubated 300 μg of protein with 20 μL of γ-aminophenyl–m^7^-GTP agarose beads (Jena Biosciences) that had been washed 3 times in buffer A with 0.5% NP-40, rotating at 4°C for 3 hours. The beads were then washed thrice with buffer A/0.5% NP-40, boiled in 1× Laemmli buffer, and loaded onto gels for blotting, as described above.

### Amino acid starve and stimulation assays.

For immunoblots, cells were washed with PBS, then cultured in RPMI-1640 lacking arginine, leucine, or lysine (Thermo Fisher Scientific) for 3 hours (starve). Medium was aspirated and replaced with full RPMI-1640 (stimulation) for 10 minutes, after which cells were washed with ice-cold PBS and lysed. For microscopy experiments, starvation was limited to 2 hours, and stimulation was achieved by careful addition of RPMI-1640 supplemented to 2× arginine, leucine, and lysine, achieving 1× final concentration. Microscopy experiments were carried out in the presence of 10% dialyzed FBS. After 10 minutes, medium was aspirated and cells were immediately fixed.

### Microscopy.

Cells were plated on chamber slides (BioTek) coated with poly-l-lysine. Cells were fixed for 15 minutes with 4% PFA, washed, permeabilized for 5 minutes with 0.01% Triton-X-100 in PBS, washed, incubated for 1 hour with primary Ab in PBS with 1% BSA, washed, incubated with fluorescent conjugated secondary Abs for 1 hour, washed again, and sealed behind coverslips with ProLong Anti-Fade with Hoechst 33342 (Thermo Fisher Scientific). Slides were imaged on an OMX-SR system using a TIRF 60× objective (Nikon Imaging Center, UCSF Center for Light Microscopy). Colocalization of MTOR and LAMP2 was measured by Pearson’s correlation coefficient using DeltaVision SoftWoRx software.

### Cell line xenograft experiments.

The Rh30 cell line stably expressing dCas9-KRAB was transduced with either guide RNA targeting *WDR59,* nontargeting control, or bicistronic versions of those vectors including an shRNA targeting *PAX3-FOXO1*, and selected in puromycin for 7 days. Once *WDR59* and/or *PAX3-FOXO1* knockdown was confirmed by immunoblot, cells were trypsinized, washed in PBS, and suspended in 50% Matri-Gel (Corning), then injected s.c. into the flanks of NSG mice (UCSF LARC colony, initiated from Jackson Laboratory strain 005557). Animals were monitored twice weekly and diameters of s.c. tumors were measured by calipers. At the end of the experiment, mice were humanely euthanized following IACUC protocols, and tumors were dissected out and digested following the PDX protocol described below, then filtered and analyzed on a flow cytometer.

### PDX experiments.

FP PDX were obtained through the Childhood Solid Tumor Network (St. Jude’s) (44). Viably frozen PDX were thawed, washed with PBS, resuspended in 50% MatriGel (Corning), and implanted in the flanks of NSG mice. Animals were euthanized when tumors reached 2 cm in maximum dimension, following IACUC protocols, and tumors were surgically extracted, macerated with a scalpel, and digested for 1 hour in buffer containing 0.1% collagenase (Sigma), 0.1% BSA, 20 mM HEPES, 1 μM CaCl_2_, 1.25% Kolliphor-P188 (Sigma), and DNAse. Cells were strained, washed thrice, and 1 million viable cells in 50% MatriGel were implanted s.c. into the flanks of recipient mice for therapeutic studies. Animals were monitored daily, and tumor diameters were measured twice a week to calculate volumes.

RMC-6272 (Revolution Medicines) was resuspended in a 1:1 mix of Transcutol and Kolliphor HS15 (Sigma-Aldrich), then diluted 10-fold with sterile water. Once tumor volumes reached 100 mm^3^, animals were randomized to either vehicle or RMC-6272 administered at a volume of 10 μL/g once a week by i.p. injection. Animals received 0.5 mL of normal saline by s.c. injection on dosing days. Body-condition scoring was monitored daily with weights measured twice a week. After 28 days, animals were euthanized. Temsirolimus (SelleckChem) was dissolved in DMSO at a concentration of 100 mg/mL, then diluted into a total of 30% PEG-300 and 2% Tween-80 (both Fisher Scientific) and administered at a volume of 10 μL per gram of body weight twice a week by i.p. injection. Monitoring and endpoints were the same as with RMC-6272. A cohort of mice were euthanized 24 hours for pharmacodynamic analysis after receiving single doses of RMC-6272, temsirolimus, or vehicle.

### Abs, sgRNA, and drugs.

Abs from the following sources were used for immunoblotting. From Cell Signaling Technologies, we used: 4EBP1 (9644); (p)-4EBP1 (2855); 4EBP2 (2845); AKT (4691); (p)-AKT Ser473 (4060); EIF4E (2067); EIF4G (2469); EIF4E (2067); ERK (4695); (p)-ERK (4370); FGFR4 (8562); FOXO1 (2880); HA (3724); P70S6K (2708); (p)-p70S6K (9205); (p)-p90RSK Thr359/Ser363 (9344); MTOR (2983); RAS (67648); RPS6 (2217); (p)-RPS6 Ser240/244 (2215); (p)-RPS6 Ser235/236 (2211); WDR59 (53385). ProteinTech: GAPDH (60004-1-Ig); MIOS (20826-1), SEC13 (15397-1-AP); WDR24 (20778-1). From Abcam, we used SEH1L (ab218531). From Santa Cruz Biotechnology, we used LAMP2 (sc-18822). And from Sigma, we used Actin (A2228) and FLAG (F1804). Small molecules, including ulixertinib, sapanisertib, trametinib, and temsirolimus, were purchased from SelleckChem, and on-target effects were validated by immunoblot. RMC-6272 was provided by Revolution Medicines. Sequences used for sgRNA and shRNA are listed in Supplemental Table 2.

### Plasmids and cloning.

The shP3F vector was made by cloning the shP3F sequence into the pCDH-LMN vector with a T2A-GFP expression cassette (45).

The dCas9-KRAB-BFP, mU6 sgRNA-Puro-T2A-GFP, mU6 sgRNA-Puro-T2A-BFP, and hU6 sgRNA-Puro-T2A-BFP plasmids were gifts from Dr. Jonathan Weissman (Whitehead Institute). To generate dual sgRNA constructs, the mU6 sgRNA-Puro-T2A GFP vector was digested with XbaI and KpnI, and the 3.9 kb fragment was ligated into the 5.5 kb backbone of the hU6 sgRNA-Puro-T2A GFP vector digested with AvrII and KpnI.

To generate dual sgRNA and shRNA constructs, the shP3F construct was cloned into the pLKO vector (Addgene). The mU6 sgRNA-Puro-T2A-GFP or -BFP cassette was then digested with XbaI and KpnI, and the 3.9 kb fragment was ligated into the 6.1 kb backbone of pLKO-shP3F digested with SpeI and KpnI.

WT or mutant cDNAs of *NRAS* were synthesized as gBlocks (IDT), then cloned into pLV1-Hygro with a Gibson Assembly MasterMix (NEB). The *PAX3-FOXO1* cDNA was synthesized using a BioXP system (Synthetic Biosystems, Inc., at the Center for Advanced Technologies, UCSF) and cloned into pBABE-Neo with a Gibson Assembly MasterMix (NEB).

### Statistics.

Statistical analysis was completed using GraphPad Prism or R. All experiments were carried out in at least triplicate, and in the figures, exact numbers of replicates are stated and plotted. Analysis of CRISPR-interference screen data was completed using open source code provided by author MAH, available at https://github.com/mhorlbeck/ScreenProcessing Differences in flow cytometric composition of cultures was analyzed by 2-way ANOVA to distinguish between effects of *PAX3-FOXO1* and/or GATOR2 knockdown, with pairwise comparisons tested by a post hoc Sidak’s multiple comparisons test. Differences between mean tumor volumes or percent GFP composition in 2-variable xenograft experiments were compared using a 2-tailed Student’s *t* test. To quantify phosphorylation of target proteins, band intensities of phosphorylated and total protein were quantified in ImageJ. Phosphorylated levels were normalized to total protein levels. Comparisons were by2-tailed *t* test for 2-sample comparisons or by 2-way ANOVA to compare the effects of genotype and treatment. Post hoc comparisons to a single control were made with Dunnett’s multiple comparisons test, and pairwise comparisons were made using Sidak’s multiple comparisons test. Analysis of mTOR localization was made by Pearson’s correlation coefficient using SoftWoRx software (DeltaVision). Comparison of PDX growth curves was performed by a linear mixed-effects regression model, with post hoc comparisons to the control by Dunnett’s multiple comparisons test. In all experiments, an adjusted *P* value of less than 0.05 was considered significant.

### Study approval.

All procedures were carried out in accordance with IACUC policies under an approved protocol.

## Author contributions

AJS led the project, conducted experiments, analyzed data, coordinated the contributions of other authors, and wrote the manuscript. TGB, JSW, and WCG provided project oversight and analyzed data, and TGB edited the manuscript. MAH provided experimental guidance in the sgRNA screen and analyzed data that it produced. JM and DVA conducted experiments and analyzed data, with support from KK, PKS, YP, JAG, and DAF. All authors reviewed the final manuscript.

## Supplementary Material

Supplemental data

Supplemental table 1

## Version 1 10/25/2022

In-Press Preview

## Version 2 12/08/2022

Electronic publication
