# Supplementary material for: GATOR2-dependent mTORC1 activity is a therapeutic vulnerability in FOXO1 fusion–positive rhabdomyosarcoma
Source: JCI Insight. 2022 Dec 8;7(23):e162207. doi: 10.1172/jci.insight.162207 (PMC9746907; doi:10.1172/jci.insight.162207)
Supplement: Supplemental data [file jciinsight-7-162207-s197.pdf]

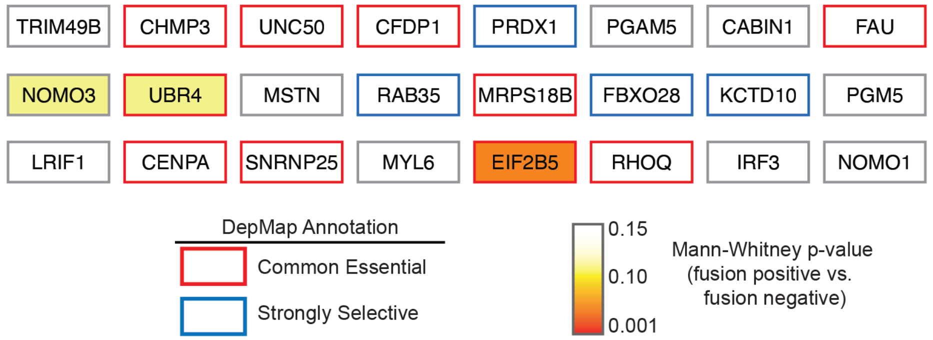

Figure S1: extended screen results. Genes meeting statistical criteria for enhanced dependence in *PAX3-FOXO1*$^{+}$ cells compared to *PAX3-FOXO1*^{KD} cells, as bounded in the blue box in Figure 1D, that lacked structural interactions with other screen hits. As in Figure 1, gene outlines indicated DepMap annotations (common essential, red; strongly selective, blue) and shading indicates Mann-Whitney significance for enrichment in FP over FN cell lines in the DepMap.

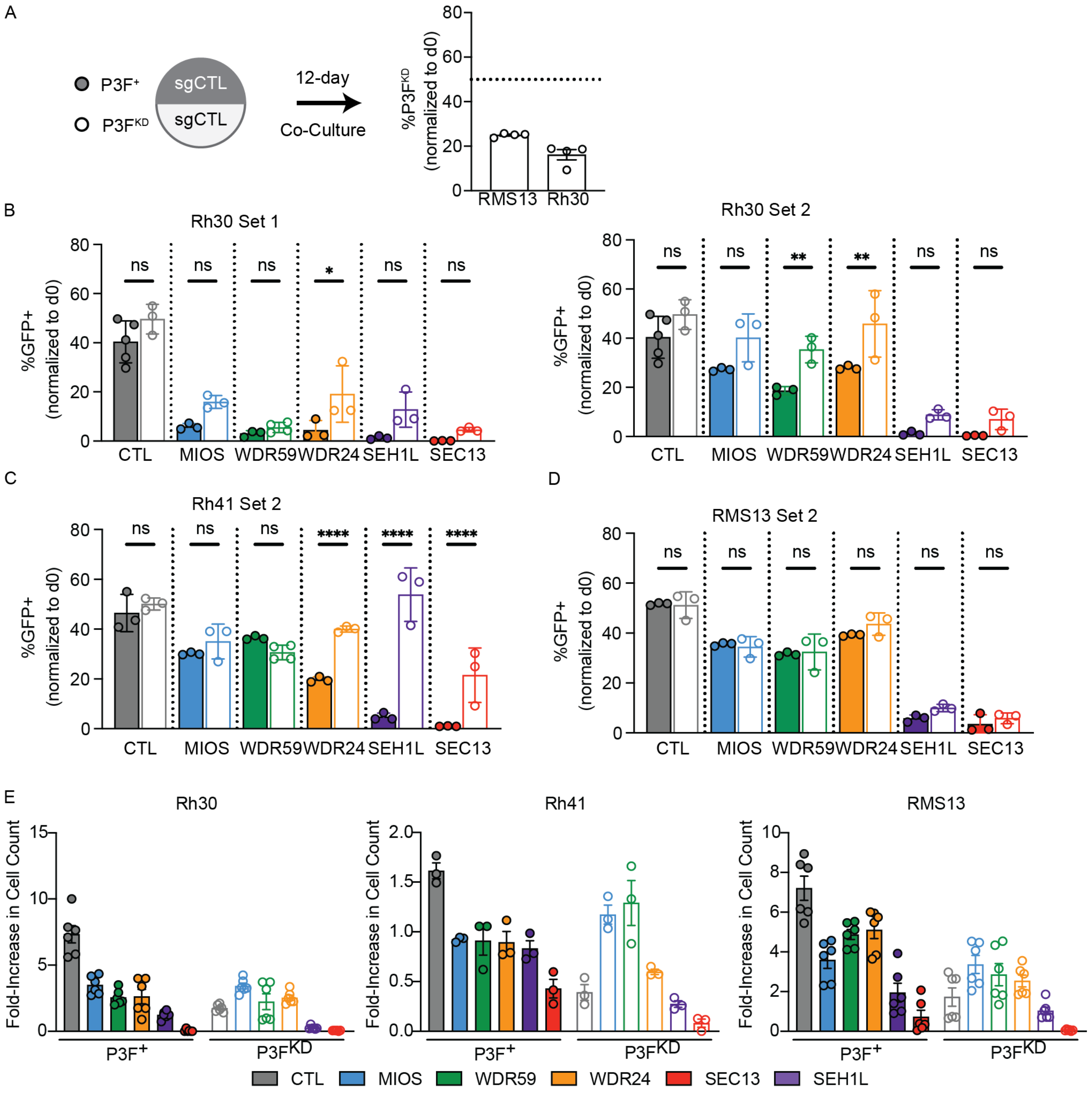

Figure S2. Knockdown of *PAX3-FOXO1* diminishes GATOR2 dependence. A, flow cytometric competition assay of P3F^{KD} cells against parental P3F^{+} cells demonstrates loss of cells after fusion knockdown in both RMS13 and Rh30 cell lines. Competition assays as in Figure 2 with additional sgRNA in *PAX3-FOXO1* positive Rh30 (B), Rh41 (C), and RMS13 (D) cells. Significance assessed by 2-way ANOVA and post-hoc Sidak's test comparing the effects of GATOR2 knockdown between P3F^{+} and P3F^{KD} cells. E, Cell counting assay reproduces the fusion-dependent effects of GATOR2 loss. Cells were transduced with the indicated sgRNA or shPAX3-FOXO1/sgRNA viral particles, seeded for 4 days in puromycin, counted, and seeded in 12-well plates. After 5 days, cells were trypsinized and counted again. Fold-change in the number of viable cells by trypan blue exclusion is quantified. Error bars show standard error of the mean.

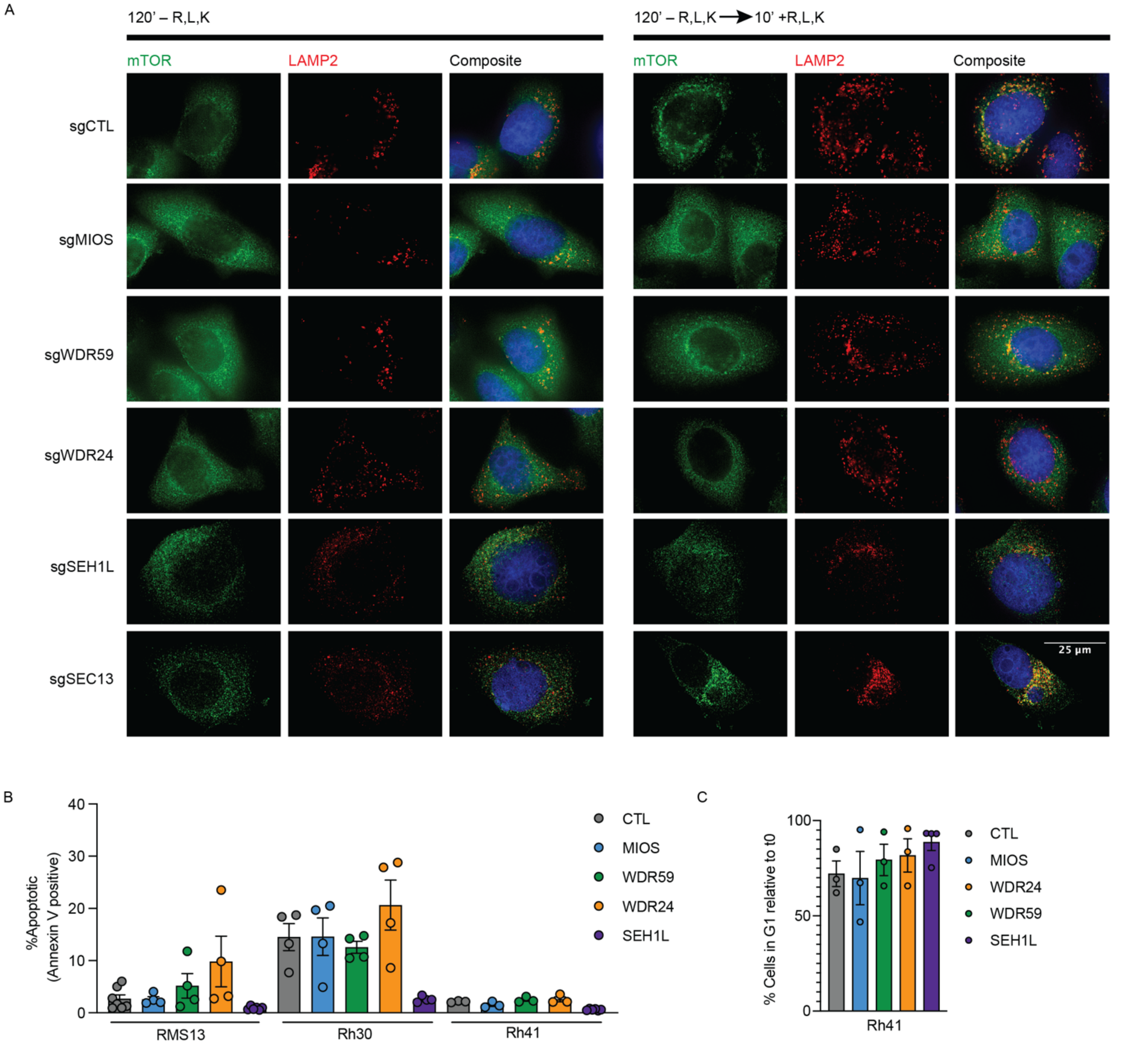

Figure S3. **GATOR2 controls localization of mTOR to the lysosome in FP RMS.** A, Representative single-channel images taken of cells fixed and stained for mTOR and LAMP2 following amino acid starvation or stimulation, as quantified in Figure 3A (n = 10-15 cells per condition). B, Indicated cell lines were transduced with GATOR2-targeting sgRNA or control, selected in puromycin for two days, then removed from selection for two additional days prior to staining for apoptosis with Annexin V and propidium iodide. Percentages of cells that were positive for Annexin V are plotted. C, Rh41 cells transduced with the indicated sgRNA underwent single thymidine block and release. Percentage of cells remaining in G1 after release are indicated, as in Figure 3D.

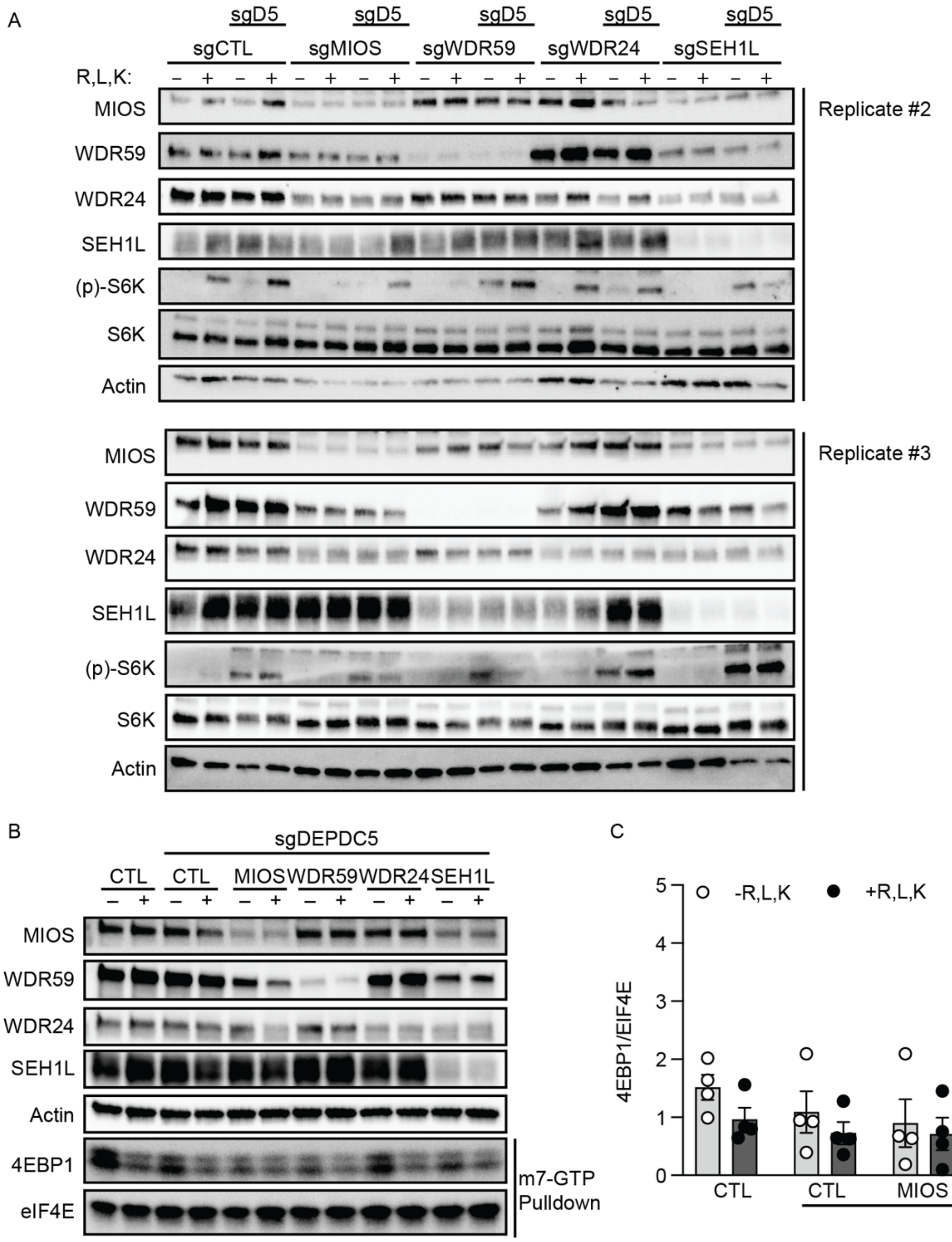

Figure S4. Genetic reactivation of mTORC1 in GATOR2 deficient cells. A, independent replicates of the experiment shown and quantified in Figure 4B-C. B, Representative m7-GTP pulldown of RMS13 cells harboring combined knockdown of GATOR2 and *DEPDC5* . C, Quantitation of 4EBP1 binding to EIF4E across independent replicates. Bars show SEM.

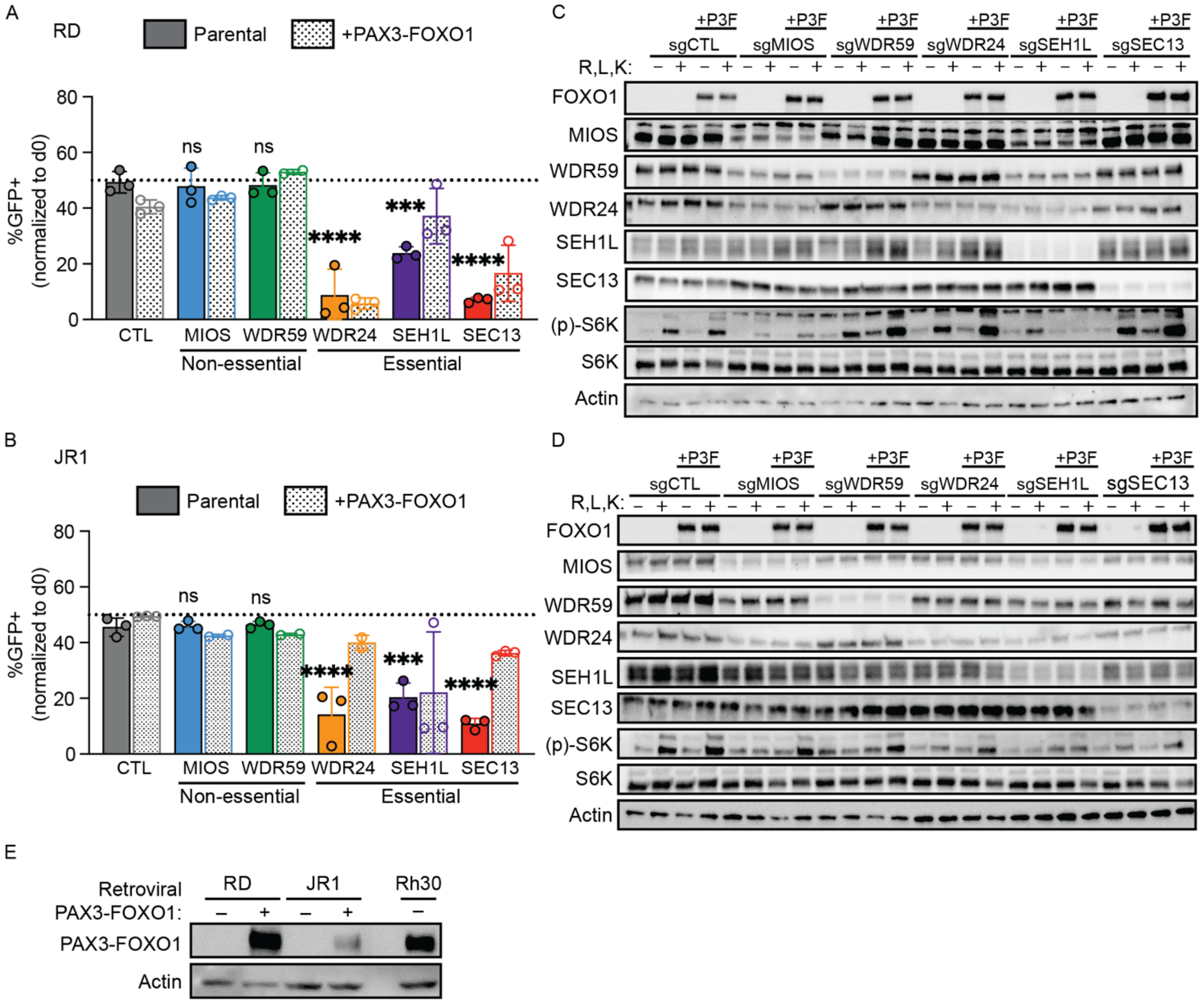

Figure S5. *RAS* mutant FN RMS cell lines do not require *MIOS* or *WDR59* for cell viability. Competition assays as in Figure 2 conducted in *RAS* mutant FN cell lines RD (A) and JR1 (B). Values for statistical significance (one-way ANOVA with post-hoc Dunnett's test) compare the indicated condition to sgCTL cells (dark gray solid bar). Genes with exclusively mTOR-dependent effects in FP RMS (*MIOS* and *WDR59*) are non-essential, and ectopic expression of *PAX3-FOXO1* does not create dependence. C-D, effects of GATOR2 loss on amino acid-induced S6K phosphorylation in RD and JR1 cells seen in representative immunoblots from three independent experiments. E, immunoblot demonstrates levels of lentiviral *PAX3-FOXO1* expression relative to fusion positive Rh30 cells.

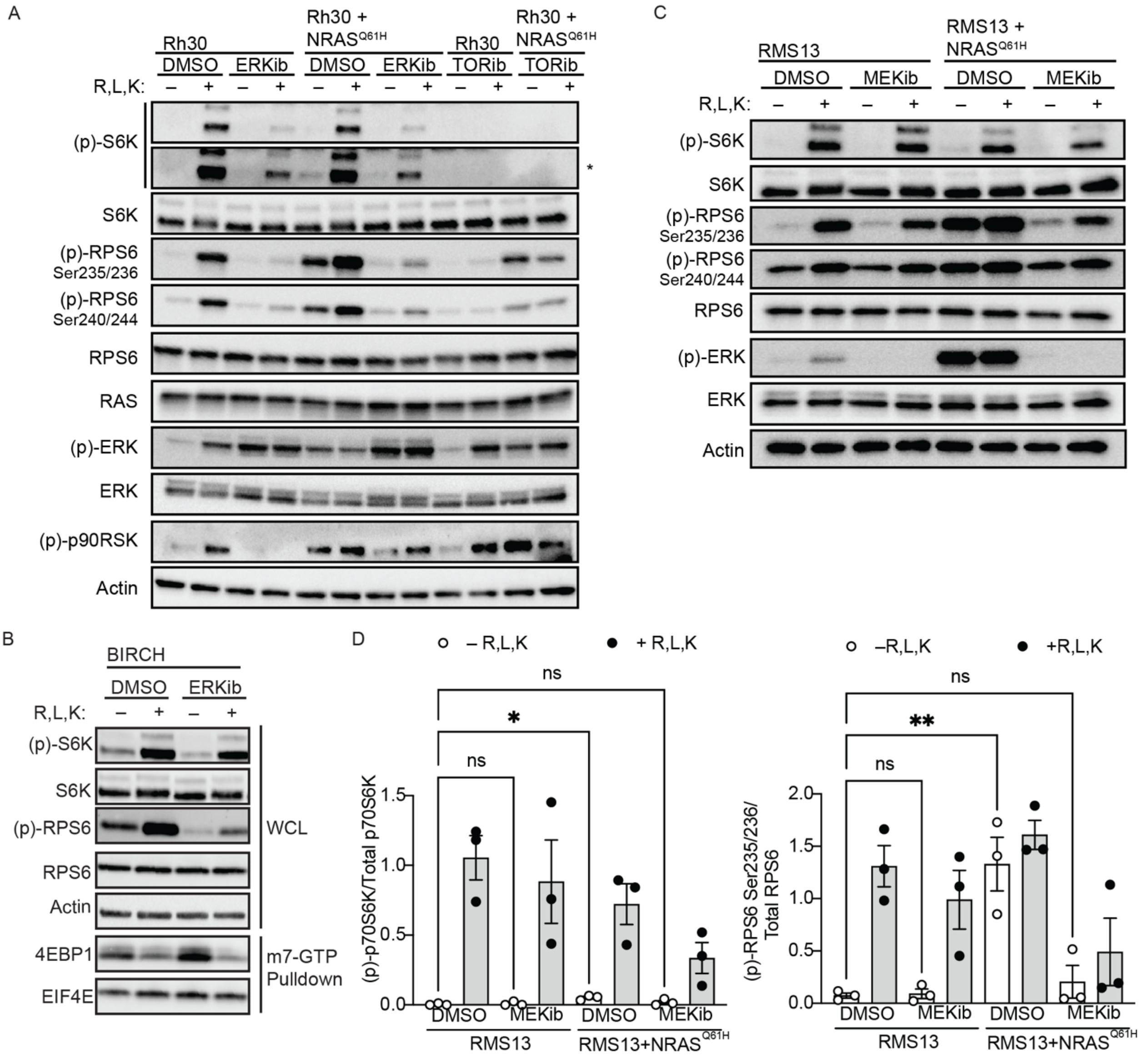

Figure S6. **Mutant *RAS* elevates basal p70S6K and RPS6 phosphorylation in an ERK-dependent manner.** Representative immunoblots or m7-GTP pulldown of FP Rh30 cells with or without exogenous $NRAS^{Q61H}$ expression (A) and FN BIRCH cells that harbor an $HRAS^{Q61K}$ mutation (B); each experiment repeated in triplicate. Cells were transduced with GATOR2-targeting sgRNA, then starved and stimulated with amino acid with or without 1 μM ulixertinib as in Figure 5D. Asterisks indicate longer exposures of the same blot. C, Immunoblot of RMS13 cells with or without exogenous $NRAS^{Q61H}$ expression with amino acid starvation and stimulation treated with or without 100 nM trametinib (MEKib). D, quantitation of p70S6K and RPS6 phosphorylation from panel C and two additional independent replicates. Error bars show SEM; significance of basal p70S6K or RPS6 phosphorylation assessed by one-way ANOVA and Dunnett's multiple comparisons test.

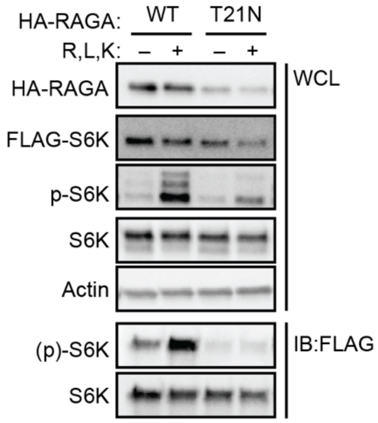

Figure S7. Phosphorylation of S6K in *NRAS* mutant FN RMS is RAG dependent. FN RD cells with an $NRAS^{Q61H}$ mutation were transfected with FLAG-tagged S6K, wildtype RAGC, and either HA-tagged wildtype RAGA or a dominant negative T21N RAGA. Cells were starved and stimulated of amino acid, then phosphorylation of S6K in transfected cells assessed by FLAG-pulldown and immunoblot. Both basal and amino acid-stimulated phosphorylation of S6K is ablated by dominant negative RAGA. Immunoblot representative of three independent replicates.

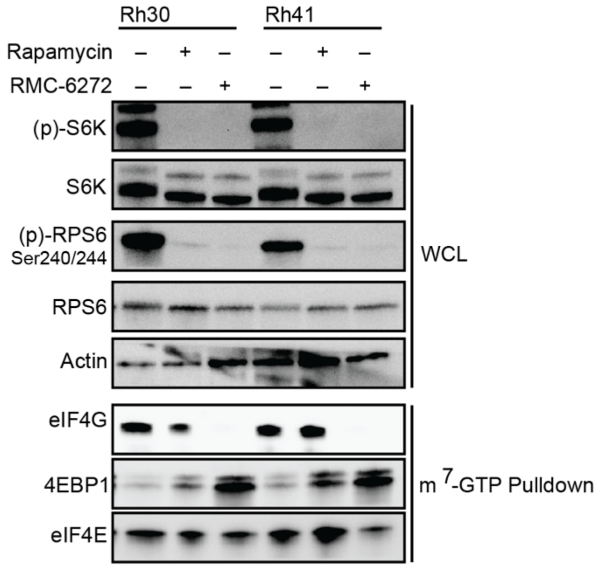

Figure S8. Comparison of rapamycin and RMC-6272 in FP RMS. Rh30 and Rh41 cells were treated with DMSO, 1 nM of rapamycin or RMC-6272 as in Figure 6B. Representative immunoblots and m7-GTP assays from three independent replicates demonstrate incomplete mTORC1 suppression by rapamycin.

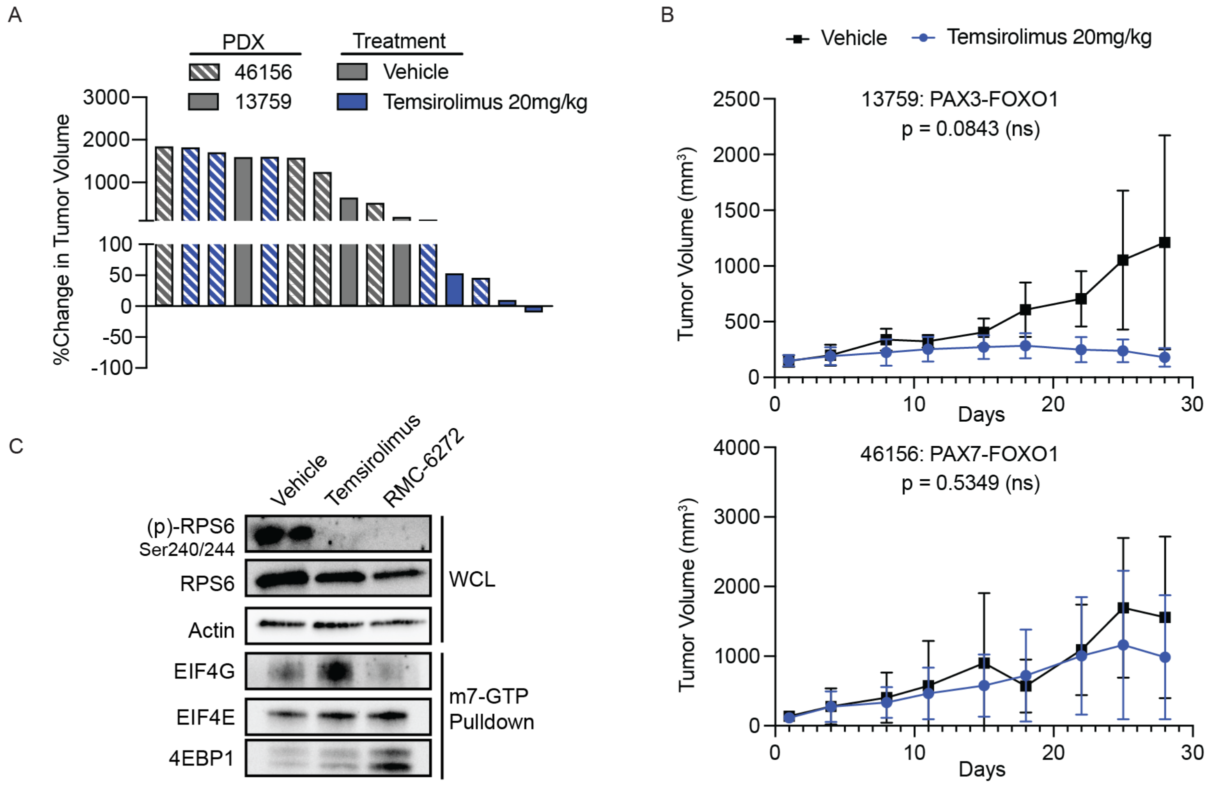

Figure S9. Effects of temsirolimus in RMS PDX. A, waterfall plot demonstrating tumor response after 28 days based on PDX and treatment (temsirolimus was dosed at 20 mg/kg by IP injection twice weekly). B, tumor growth curves of two FP RMS PDX treated with temsirolimus. Differences between vehicle and drug treatment were measured by a linear mixed-effects regression model with Dunnett's multiple comparisons test. C, mice harboring the 46156 PDX were euthanized 24 hours after administration of vehicle or the indicated doses of RMC-6272 or temsirolimus. Immunoblot of whole cell lysates from excised, flash-frozen PDX demonstrates inhibition of RPS6 phosphorylation with both agents.

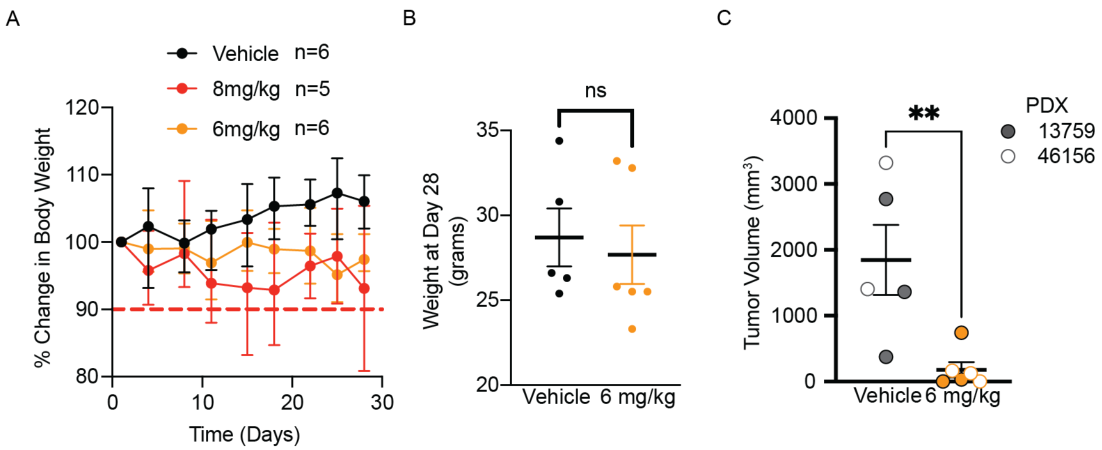

Figure S10. Tolerability and efficacy of RMC-6272. A, Weights of mice treated during dose-determining preclinical studies with RMC-6272. Error bars denote range. B, weights of mice treated with either vehicle or 6 mg/kg RMC-6272 after 4 weeks of treatment during dose-determining studies. No significant difference in weights was found by a student's t-test. C, tumor volumes after 4 weeks of either vehicle or 6 mg/kg RMC-6272. Difference in volumes significant by a student's t-test.

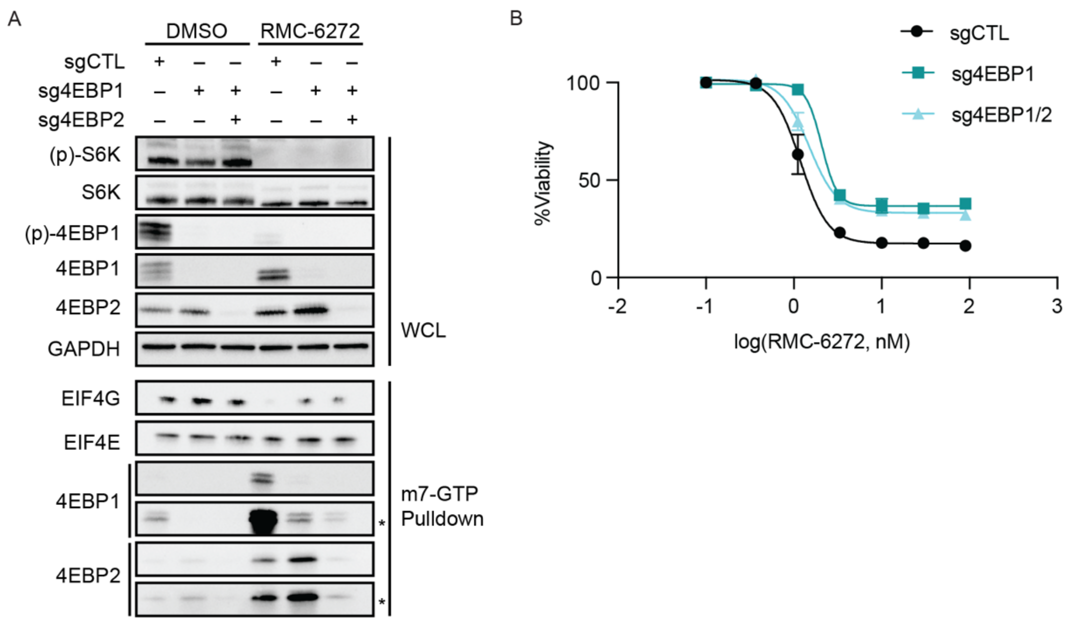

Figure S11. Anti-tumor effects of RMC-6272 are not exclusively mediated by 4EBP1 and 4EBP2. A, RMS13 cells were transduced with sgRNA targeting 4EBP1 and/or 4EBP2 and assessed by immunoblot and m7-GTP pulldown after treatment with DMSO or RMC-6272 (1 nM) for 8 hours. Blot is representative of three independent replicates. B, viability of RMS13 cells transduced with the indicated sgRNA after 6 days of treatment with RMC-6272 assessed by Alamar blue staining (n = three replicates per dose).

Table S2. sgRNA and shRNA Sequences

sgRNA Target	Protospacer Motif
CTL (*GAL4*)	GAACGACTAGTTAGGCGTGTA
MIOS	GTCGCGCTCACTGACCTGAGG
	GACGCCCGGGCCTTTCAACT
WDR24	GGTTCCTGGGAGCGGCGCAG
	GAGACGCGGGAAGGGCCCAG
WDR59	GGCACGGTCCCGGGATACTG
	GCCGCCGTCCCCAGTATCCC
SEH1L	GGCTCCCGGGCTGCGAGGTC
	GCCGAAGAGGACAGTGGCGG
SEC13	GAGCTGCCACGTCCGAGACC
	GCTGCTCCAGGTCTCGGACG
DEPDC5	GGCGTAGGCGGGGTATCTGG
EIF4EBP1	GCACAGGAGACCATGTCCGG
EIF4EBP2	GCTGAGGCCGGAGGATCGAG
**shRNA Target**	**Seed Sequence (*PAX3* in capitals, *FOXO1* lower case)**
PAX3-FOXO1	CCTCAGaattcaattcgtcat
