# Supplementary material for: GATOR2-dependent mTORC1 activity is a therapeutic vulnerability in FOXO1 fusion–positive rhabdomyosarcoma
Source: JCI Insight. 2022 Dec 8;7(23):e162207. doi: 10.1172/jci.insight.162207 (PMC9746907; doi:10.1172/jci.insight.162207)
Supplement: Supplemental table 1 [file jciinsight-7-162207-s198.pdf]

gene	P3F-KD_MW	P3F-KD_Log2FC	P3F+_MW	P3F+_Log2FC
A1BG	0.334192779	-0.472438268	0.076987969	-0.648882353
A1CF	0.3836854	1.041532947	0.121430145	-0.652072785
AADAC	0.33693863	-3.349555151	0.925582049	0.015786938
AAMP	0.676678945	-1.039559495	0.865006686	-1.773447558
AASDHPPT	0.420098318	-1.114174793	0.836212161	-0.193384059
ABCA12	0.637131428	-1.185902659	0.575987148	-0.351022998
ABCA2	0.987215722	-0.150214846	0.070925424	-0.328536267
ABCB6	0.867466147	-0.202582131	0.222269989	0.543005645
ABCB7	0.098539921	-4.185581086	0.047150282	-1.368698808
ABCB9	0.554756997	-0.546532314	0.312071695	-1.06762478
ABCC5	0.060079541	-1.040716587	0.272292511	-0.224350359
ABCD1	0.779912935	0.394064029	0.976616473	-0.072693437
ABHD1	0.175027451	-0.925467367	0.750689664	-0.107247919
ABHD13	0.026192564	0.748668128	0.982227445	-0.224729399
ABHD14A	0.775118987	0.710182872	0.071169972	-1.387614333
ABHD14B	0.74070691	-0.882840635	0.044888572	-0.158706649
ABHD2	0.081397238	-1.247434054	0.010771097	1.14233558
ABHD4	0.395095651	-0.126042722	0.677822752	0.150038494
ABHD5	0.942362595	-0.491758447	0.185346681	-0.278653055
ABTB2	0.799170825	-0.824932406	0.497236012	0.37169757
ACAA1	0.16212985	1.177025243	0.077118629	0.344778482
ACAA2	0.025361547	-1.055677682	0.736500304	0.24294344
ACAD10	0.373527623	1.321980941	0.739448855	0.126132336
ACAD11	0.508697209	-0.003181805	0.836822674	-0.274183868
ACADL	0.622694118	-1.413281807	0.198383347	-1.893834548
ACADVL	0.44205214	-1.655247762	0.608400445	-0.274728681
ACAP2	0.033468081	-1.355097809	0.119747749	-0.725748582
ACBD7	0.399672858	-0.820005403	0.609221095	-0.26053249
ACER1	0.606176599	-0.691483894	0.615420282	0.171750006
ACER2	0.696223142	0.279531175	0.875468135	0.138924991
ACIN1	0.038923882	0.290764022		

0.084263319 -0.598580359
ACLY 0.957300377 -0.212707495
0.707815699 -1.081641484
ACO1 0.085436911 2.294952953
0.121430145 -0.342213326
ACOT2 0.086723441 -0.817119585
0.021318863 -1.085114768
ACOT4 0.166157552 -1.983009195
0.249110536 -0.457670853
ACOT7 0.08003915 1.4091819 0.373036345 0.223696644
ACOT8 0.34927259 0.265344205
0.639365011 -0.181490606
ACR 0.189795858 0.475153116 0.450920366 0.11074122
ACSF3 0.629339917 0.682942426
0.005194536 -0.994426256
ACSM1 0.017364906 -1.835636264 0.168803523 0.747289585
ACSM2A 0.073360682 -2.849870098
0.143126574 -0.661347166
ACSM2B 0.359761057 -1.480833256 0.299847079 0.369150407
ACSM4 0.648894071 0.733168988
0.146101681 -0.192760563
ACTL6A 0.524361243 -2.145190625
0.730027188 -0.989226429
ACTR1A 0.518268561 -2.606248418
0.180211683 -0.986442697
ACTR5 0.710724579 -0.527205766 0.951696668 -1.44852352
ACTR6 0.371432561 -2.173533023
0.935522562 -0.928194448
ADAD1 0.645524202 -1.550292153
0.270249477 -1.018639412
ADAM10 0.938631109 0.226046283
0.894599755 -0.670040043
ADAM11 0.326812711 -0.88171135 0.700272636 0.272853086
ADAM18 0.918134197 0.553154964 0.118083665 0.692844339
ADAM2 0.18431112 0.307544211
0.545451244 -0.070766922
ADAM20 0.450451123 1.967462823 0.300178945 -0.94303071
ADAM21 0.339659246 -3.839241954 0.603875417 0.13081232
ADAM23 0.790127695 -0.574249033
0.388827099 -0.513732338
ADAM29 0.409809928 -1.420806392 0.092480216 0.297590299
ADAM30 0.833771101 1.251777762 0.817336961 0.188343904
ADAM32 0.511206972 0.745643158 0.812482621 0.12242153
ADAM7 0.406708666 -0.567599992 0.041073395 0.290267266
ADAM8 0.551702599 0.508564999 0.50569404 0.203676193
ADAM9 0.785316139 -0.200456637
0.31424829 -0.605016029
ADAMDEC1 0.700851836 0.807859489
0.889038588 -0.834362796
ADAMTS1 0.457518789 -1.673095281

0.238363177 -0.802490321
ADAMTS10 0.190060004 -0.939293376
0.159563229 -0.329281294
ADAMTS13 0.040592829 -1.747893635 0.335370516 0.33135591
ADAMTS14 0.499219335 -0.262408264 0.451389888 0.517815802
ADAMTS15 0.116984394 -0.568725754
0.717723028 -0.625747935
ADAMTS16 0.152944089 0.772980259
0.003033434 -0.763014827
ADAMTS17 0.00638171 1.298931134 0.521818171 0.225972128
ADAMTS18 0.123892843 -2.178063902 0.057877626 -0.26449001
ADAMTS19 0.34406579 -0.124230282
0.738268972 -0.187608723
ADAMTS2 0.136795338 -0.745456519 0.371850987 -1.45525571
ADAMTS3 0.772725211 -0.651644723 0.18821666 0.761573438
ADAMTS4 0.732967163 -0.589078657 0.370179028 0.136550255
ADAMTS6 0.659612561 -1.419595364 0.309759235 0.216329895
ADAMTS7 0.657915442 -0.264283688 0.693913017 0.202424431
ADAMTS8 0.819159157 -0.211263503 0.471117328 -0.28878262
ADAMTS9 0.729439669 0.113015974 0.242433158 0.395423767
ADRM1 0.580796849 0.743499821
0.011933292 -0.960970792
AEBP1 0.951696668 0.230534394
0.882249041 -0.178208484
AFM 0.10046681 -1.001602513
0.628717904 -0.452596141
AGA 0.167356374 -1.842044439 0.691605734 0.326497605
AGAP3 0.264518066 -0.644020632
0.275376838 -0.299785583
AGAP4 0.361478061 -0.18114929 0.191650565 0.206969786
AGBL1 0.708978743 -0.638738329 0.172216692 0.653392599
AGBL2 0.508697209 -0.493457831
0.437425034 -0.111877003
AGBL4 0.481035002 0.275467145 0.051389698 0.692282276
AGBL5 0.975369738 1.269795304
0.224946513 -0.511522534
AGFG1 0.144589549 2.482302291 0.495256895 0.028031469
AGL 0.466081566 -1.678118082
0.715971166 -0.162126662
AGMO 0.678394935 -0.028291321
0.111960342 -0.536936595
AGPAT1 0.166843568 1.309158085 0.120097383 0.349073782
AGPAT6 0.506193921 -0.14880024 0.779313228 0.546808103
AGTPBP1 0.726504455 -0.369093441
0.120867304 -0.780865974
AHCYL1 0.540268773 -0.195930883 0.586162907 0.509326387
AHSA1 0.256242669 0.718285899
0.315754102 -0.496816754
AHSA2 0.249110538 0.422597811
0.490819312 -0.620462316

AHSG	0.488471278	0.593388138	0.412874669	0.240501723
AHSP	0.212938321	-0.575317882	0.820982329	-0.025345752
AIFM3	0.204728807	-0.742300434	0.104721064	-0.940913448
AIMP2	0.377739638	-0.521635268	0.098221638	0.373638413
AIP	0.413817857	-1.899382673	0.265187965	1.251044476
AIPL1	0.248789681	2.06443155	0.493775324	-0.360701101
AKR7A3	0.472802564	1.049239125	0.196751916	-0.489092277
ALB	0.870542274	0.469551569	0.190589105	-0.018490482
ALDH1L2	0.162835215	-0.999232515	0.004007529	-0.790112674
ALG14	0.033338249	-1.608487455	0.181485414	0.554677653
ALG2	0.895217988	-0.712719063	0.46417107	0.770030432
ALG6	0.039624771	-2.188877435	0.115621619	-1.476163594
ALOX12B	0.050278055	-1.757315676	0.236810715	0.254927002
ALS2CL	0.491311321	-1.180270038	0.25986142	0.442928294
AMACR	0.758408672	-0.484008525	0.893981587	0.013865317
AMDHD1	0.422344012	0.363155415	0.363952612	-0.236996787
AMELX	0.715387545	0.356238649	0.44951346	-0.269495164
AMZ1	0.828893806	-1.455283349	0.574920885	-0.719138755
AMZ2	0.17555661	1.149554516	0.007280709	-0.82920525
ANGPTL3	0.071661143	0.572913662	0.413371388	-0.856777178
ANK1	0.455628014	0.371329836	0.901934239	-0.37433719
ANK3	0.815515752	0.27793185	0.149603404	-0.511003153
ANKIB1	0.98980719	0.755878248	0.1511877	-0.982317483
ANKRD13A	0.028942909	1.859296884	0.178439634	-0.25924873
ANKRD13B	0.237120636	-0.600036131	0.074794228	0.55081396
ANKRD13D	0.354732314	-0.127341019	0.299086377	-0.351442938
ANKRD27	0.888421012	-0.846718425	0.37140335	-0.344986356
ANKRD30A	0.348870299	0.12051876	0.750095102	-0.000616737
ANKRD60	0.398798559	-0.906458108	0.075049635	0.784327448
ANO5	0.046889975	1.83297188	0.452329761	-0.634187536
ANTXR2	0.173448114	-1.509677914	0.761980262	0.193071015
ANXA11	0.261517951	1.599108767	0.59371378	-0.381471617

ANXA6	0.039325387	-3.472998565	0.922477947	-1.053224729
ANXA7	0.468475813	0.670908841	0.5754539	-0.52345158
AOAH	0.399672858	-1.166589146	0.281966247	0.805192003
AP1AR	0.425072022	-0.388422524	0.513723184	0.244635822
AP1B1	0.356216826	-0.732560352	0.382136649	0.452202259
AP1G1	0.102259652	-2.001069973	0.675535882	0.218380399
AP1G2	0.032594273	-2.054715358	0.159995277	0.380229317
AP1M1	0.387108559	-0.752925516	0.292586746	0.367716897
AP1M2	0.113556313	1.418632839	0.130319381	0.415201428
AP1S1	0.480062447	1.132560093	0.861934202	-0.091745494
AP1S3	0.376472971	1.435733798	0.227796626	0.179866532
AP2A1	0.180720378	-0.924962219	0.729439668	0.185252568
AP2A2	0.50120685	0.336396382	0.041384019	-0.704950378
AP2B1	0.168320102	-2.237108321	0.04758683	-0.650465052
AP2M1	0.050924038	-1.900983606	0.3146243	-0.386717675
AP2S1	0.217568183	-1.60562858	0.74890979	-0.85893929
AP3B1	0.115712082	0.96459022	0.53186756	-0.375525345
AP3B2	0.343667329	0.166725292	0.995946292	-0.290958656
AP3D1	0.278311629	-0.877073012	0.25986142	0.273932045
AP3M1	0.149318592	0.598353854	0.077006292	-0.630691915
AP3M2	0.062901505	-1.165388609	0.875468135	-0.13668929
AP3S1	0.399672858	-1.495479929	0.028148819	0.756026539
AP3S2	0.91627333	-0.799251205	0.706072413	-0.127556113
AP4B1	0.144804831	-0.887915388	0.388396997	-0.371458393
AP4E1	0.547010783	0.600991123	0.490327569	-0.268478572
APBA1	0.193786519	0.843392833	0.763171924	-0.04103338
APBA2	0.365191231	-0.362348624	0.099632719	0.282307065
APBA3	0.485913835	0.865865362	0.143303003	0.443698638
APBB3	0.828284604	-0.273686147	0.179450639	0.049411319
APEH	0.601853042	0.467652336	0.265523354	0.362010548
APH1A	0.021362953	0.47580879	0.368511746	-0.226910471
APH1B	0.419197696	0.443034049	0.231281075	-0.194023741
APLNR	0.170991827	2.452912391	0.224349749	0.166708913
APLP1	0.769138604	-0.62003811	0.534082488	-0.188820115
APOA1	0.160959368	2.068726766	0.789525808	0.179586961
APOA1BP	0.696223142	-0.689667654		

0.019374581 -0.740901622
APOA2 0.145884926 -4.061027509 0.525890126 0.0888082
APOA4 0.965398278 -0.907922539
0.310131697 -0.382862661
APOA5 0.403300358 1.805434944 0.232807803 -0.61434858
APOB 0.55693881 0.385607902 0.735911058 0.144720032
APOBEC1 0.331461398 -4.073073678
0.21293832 -1.981416607
APOC1 0.065602809 -2.997525498 0.03138031 0.842269119
APOC2 0.235882677 0.968703743
0.131715272 -0.420675518
APOC3 0.797360001 -1.020183198
0.781112738 -1.238045531
APOC4 0.032000838 -1.307912157 0.741219828 0.686765226
APOF 0.724745249 0.251468461
0.943606709 -0.788568463
APOOL 0.656502522 -0.472918495
0.140335234 -0.688550093
APPBP2 0.290441441 -2.588578084 0.722987494 0.025565789
AQP11 0.572259321 -0.460545805 0.733555628 0.590708968
AQP2 0.836212162 -0.565456876
0.252011272 -0.597312293
AQP6 0.258212172 -3.623683579
0.871773276 -0.189529339
AQPEP 0.304944126 -1.079274426
0.927445058 -0.308051349
ARAP2 0.073527823 -1.670233664
0.81794425 -1.557806503
ARC 0.947028723 -2.205159413 0.37605133 0.400159813
ARCN1 0.134338353 -4.998136379 5.60E-05 -5.032711866
AREL1 0.384112279 -0.954229094 0.693913017 0.785851092
ARF3 0.998440876 1.062771637
0.110731307 -0.363537347
ARFGAP3 0.006914791 -2.083219301 0.133729468 0.186327424
ARFGEF1 0.311811436 -1.245220332
0.200849007 -0.325110581
ARFGEF2 0.048115168 1.269169367
0.041696631 -0.608911899
ARFIP2 0.03237818 -1.287432825 0.811270153 0.305075115
ARFRP1 0.714220799 -0.793302155 0.707234433 -0.49627638
ARHGAP12 0.368095659 1.275126329
0.332630216 -0.141989688
ARHGAP27 0.70433067 -0.309175856
0.345662576 -0.767636842
ARHGAP30 0.138036763 -0.860130107 0.832551169 0.147524367
ARHGAP33 0.235265416 -0.722374633 0.582940511 0.212250463
ARHGAP4 0.693913019 0.526902183
0.718307312 -0.607287602
ARHGAP40 0.669831834 -0.166724483 0.243379285 0.295643393
ARHGAP42 0.82463157 -0.001562826

0.413817855 -0.256519475
ARHGAP6 0.371850989 -0.61548282 0.054750095 0.486479965
ARHGDIA 0.13265351 -1.457184342
0.000244954 -1.541591412
ARHGDIB 0.965398278 -3.216997544
0.007280709 -1.447862542
ARHGDIG 0.082494965 0.53916333
0.250880421 -0.907382132
ARHGEF10 0.744765884 -1.218100512 0.660178656 0.228951513
ARIH1 0.371432561 0.36337479
0.000984362 -0.853627871
ARIH2 0.732967163 -0.945555598 0.771529131 0.061693281
ARL2 0.270249479 -3.078591657
0.008563375 -2.368845387
ARL4A 0.147877677 -0.940810578 0.209007172 0.259769241
ARL4C 0.496245929 1.024773886
0.048916908 -0.602375554
ARL4D 0.262515365 -0.581064127
0.722987494 -0.021143552
ARL6IP1 0.572259321 -1.903066829
0.249431683 -0.296913258
ARL8A 0.737089707 0.416203014
0.356564131 -0.387254293
ARL8B 0.708978743 0.864856983 0.701431207 -0.16995888
ARMC5 0.291870466 -1.476973183
0.64160187 -0.233520842
ARR3 0.318779921 -1.665537253
0.980980476 -0.011860742
ARRDC2 0.460363192 0.241791502 0.377317122 0.836194854
ARSB 0.843544723 -0.809770133
0.464648284 -0.418107015
ARSG 0.318211138 0.391857676
0.312747208 -0.434882436
ASAH1 0.30273861 0.135592777
0.835601747 -0.228347671
ASB1 0.98971006 -0.545450726 0.305681659 0.563391135
ASB10 0.124850347 -0.771010144 0.992204499 0.265319756
ASB11 0.189268378 0.563279141
0.039549744 -0.800838024
ASB12 0.071661143 -1.168899917 0.466559869 0.14328466
ASB13 0.381555367 -1.409826378
0.648331923 -0.254229669
ASB14 0.576573126 -0.401479644
0.061068316 -1.024447759
ASB15 0.071538091 1.306802168 0.091338478 0.105760743
ASB16 0.72415917 -0.015401456 0.1683201 0.030648573
ASB17 0.936765866 -0.180398402
0.331266848 -0.041767903
ASB18 0.822198317 -0.129504268
0.831331641 -0.121640308

ASB2	0.379856576	1.045393088	0.053674762	0.685813665
ASB3	0.093815792	-1.699346896	0.707344934	-0.116042704
ASB4	0.68527551	-0.129083598	0.133729468	0.101849485
ASB5	0.537171155	1.091283192	0.768541313	-0.256873135
ASB6	0.649456418	-0.546319119	0.620485628	0.176861201
ASB7	0.625308989	0.062876886	0.999671191	-0.257539422
ASB8	0.409366031	1.088268403	0.861319954	-0.216360356
ASB9	0.292945331	0.381465066	0.45327074	-0.454878031
ASCC2	0.974123063	-0.260976143	0.252983397	-0.596781092
ASGR1	0.237741334	-1.502436265	0.068044237	0.972317774
ASPHD1	0.656220085	0.747443052	0.338119849	-0.2422021
ASPRV1	0.168803525	-1.788927434	0.026884994	0.653633041
ASTL	0.140967173	-0.145946235	0.942984634	0.132021377
ASZ1	0.164490066	-0.462549254	0.300543725	-0.20346682
ATAD2	0.576520631	-0.589309467	0.647769977	-0.055333539
ATE1	0.579726403	-0.470375879	0.008944729	-1.219070992
ATF4	0.062790977	0.973075402	0.299450271	-0.280153112
ATF6	0.09758754	-0.463167569	0.19947645	0.404832766
ATF6B	0.382530809	0.419054735	0.299999333	0.875483316
ATF7	0.1649652	1.542569105	0.13928685	-0.256650119
ATG13	0.087315035	-7.197238866	0.084151157	-1.79803865
ATG16L1	0.204491016	0.866873702	0.337994402	0.061416694
ATG2A	0.358606465	0.222001259	0.74772507	0.077969996
ATG4B	0.660178657	0.408686479	0.979110112	-0.125510636
ATG4C	0.695067727	-0.529045051	0.563254049	0.158052275
ATG4D	0.520295381	-2.008580774	0.955432226	0.457383172
ATG9A	0.629986081	0.227855093	0.227044114	0.228333046
ATG9B	0.334584158	1.818868017	0.034790357	-0.755636344
ATHL1	0.168320102	2.002421398	0.289372764	-0.413635509
ATL3	0.968513851	-0.540410251	0.889656231	-0.250744559
ATP10B	0.525380247	0.272215948	0.316131295	-0.175466029
ATP1A3	0.618831505	0.138975418	0.830112519	0.049987037
ATP2A2	0.606214453	0.336075701	0.392278089	-0.793277586
ATP2B2	0.234649298	0.719258164		

			0.042248512	-0.668198008
ATP2B3	0.054032784	-1.403411458	0.622976071	0.083430539
ATP5F1	0.283016378	1.15805046	0.917513857	-0.017151822
ATP5G1	0.043128235	0.371849174	0.334584156	0.187586894
ATP5G2	0.068755355	-1.175076863	0.055145554	0.176703492
ATP5G3	0.162835215	-3.156514312	0.526910647	-0.409758979
ATP5H	0.861934203	-0.640021381	0.033794553	-0.613928454
ATP5I	0.483471079	1.215166651	0.799170824	-0.925778392
ATP5J	0.001587611	2.217694217	0.042967143	-1.056741693
ATP5L	0.520802723	-0.630237117	0.772127102	-0.107861593
ATP5O	0.634344079	-0.353352418	0.794947264	-0.257308199
ATP6AP1	0.042967144	-3.077805774	0.001184488	-4.982670587
ATP6AP1L	0.377739638	-0.330131999	0.575987148	-0.363215289
ATP6V0A2	0.274689382	-0.614040974	0.135767412	0.391021673
ATP6V0B	0.014852823	0.748228521	0.078170425	0.882750809
ATP6V0C	0.138660722	-0.40300082	0.346863262	-0.224840995
ATP6V0D1	0.472320731	-0.445714281	0.106259362	0.247139479
ATP6V0D2	0.927445058	-0.073660102	0.043614574	0.612962699
ATP6V0E1	0.343269164	1.498184476	0.468955476	-2.230180294
ATP6V0E2	0.73002719	1.162647028	0.965398278	-0.841241495
ATP6V1B1	0.885334157	-0.635331428	0.907595511	-0.244667266
ATP6V1C2	0.720646096	0.804970032	0.470396101	-0.324418513
ATP6V1E2	0.889038588	-0.302994356	0.323354121	0.210114955
ATP6V1F	0.447641465	-0.5104268	0.442980909	-0.806235609
ATP6V1H	0.48542475	0.279677658	0.652271141	0.267993694
ATP7A	0.173695185	0.79143772	0.951696668	-0.480215039
ATP9B	0.713637677	1.563041176	0.92806615	-0.091736354
ATPAF2	0.882865923	0.863761302	0.445773906	-1.146041519
ATXN3L	0.547010783	-0.277698728	0.019374581	-1.812226725
AUP1	0.050924038	1.730738363	0.531515468	-0.260587363

AVIL	0.930550952	0.481424473	0.068399035	0.81385455
AVL9	0.70491108	0.172794275	0.773921829	0.258215059
AVP	0.592091994	0.154791919	0.020752871	0.238270265
AWAT1	0.860091711	0.430978132	0.581332417	0.186958475
AWAT2	0.486403187	-2.288656501	0.682405389	0.175783453
AZU1	0.145633343	-0.558766252	0.128450223	0.316245382
B3GALT5	0.125411814	0.978941603	0.172664472	0.241328966
B3GALT6	0.906976076	-0.275896524	0.324504323	0.297424203
B3GALTL	0.333020415	-0.369270678	0.322206579	0.901562039
B3GNT1	0.332630218	-1.447395761	0.171971195	0.706273169
B4GALNT1	0.012012873	1.888523376	0.919995512	0.05668139
B4GALNT2	0.38796725	0.450240029	0.368511746	-0.505752749
BACE2	0.872081075	1.008204847	0.243379285	-0.580207451
BAG2	0.205008007	2.080022362	0.151601067	-0.387739474
BAG3	0.299450273	-0.337817883	0.017145216	-0.971569459
BAG4	0.016784483	-5.452295299	0.174934487	-0.687260418
BAG5	0.056852911	1.563690161	0.426434431	0.33713973
BAG6	0.02648203	1.410922617	0.661609399	-0.410787826
BAHD1	0.168309922	-0.629372953	0.177811562	-0.369345324
BAP1	0.064443574	-1.275670896	0.527549385	-0.289714031
BATF3	0.502700233	-0.58678867	0.712471933	-0.137980598
BAZ1A	0.21932291	-0.73513508	0.382193612	0.52816307
BAZ1B	0.563781875	-0.597130447	0.265523354	-0.763206496
BBS10	0.674964633	-0.2633382	0.51120697	0.466200665
BBS12	0.860091711	-0.739191051	0.668124306	0.135865744
BCAP31	0.215440558	1.283614065	0.137419471	-0.985258194
BCS1L	0.350481227	-3.950521593	0.015242378	-2.988734467
BDKRB1	0.627675622	-0.327972368	0.036712981	-0.679055327
BECN1	0.586175987	1.326339438	0.031691187	-0.828209763
BET1	0.749502373	-0.280128617	0.191650565	-0.462658741
BET1L	0.036575804	-0.590145392	0.227344906	-0.594544788
BFAR	0.340094456	-1.094432611	0.222566249	0.625127244
BGN	0.299814463	-0.74757703	0.92434027	-0.288381911

BHLHA15 0.132920951 1.701299933
0.386679646 -1.109628657
BIRC2 0.708978743 0.31323029 0.130120909 0.266749086
BIRC7 0.425688113 0.448644554
0.461472059 -0.131346845
BIRC8 0.454979244 -0.525117566
0.054032783 -0.834859781
BLMH 0.313872578 0.646531181
0.500709577 -0.152260461
BLOC1S6 0.747132935 0.424823515
0.546490691 -0.239081844
BLZF1 0.553723637 -0.351619713
0.98520443 -0.258867232
BMI1 0.088025567 0.450597209 0.636015868 0.097647065
BMP1 0.29402285 1.682729152
0.393576985 -0.555165796
BMP6 0.994075379 -1.419267212 0.190854064 -0.12843602
BPIFA2 0.376894902 -0.053476634 0.277446253 0.526708855
BPIFA3 0.857636242 -0.09959109 0.452329761 0.088579804
BPIFB3 0.055543392 -1.802244603 0.737089705 0.107268811
BPIFB4 0.222862794 -2.906956576 0.851503642 0.342496474
BPIFB6 0.598048456 -1.978367882 0.216694613 0.478650675
BPIFC 0.379856576 -1.322759239
0.143303003 -0.667422353
BRAP 0.3836854 -0.295342253 0.985968598 0.365433342
BRIX1 0.051389699 -0.617972832
0.213513131 -0.764625654
BROX 0.397925412 0.328939179 0.236501082 0.209254596
BRPF1 0.198383349 1.105597207
0.361478059 -0.328309931
BRPF3 0.898310098 -2.56535947
0.310504455 -0.817774988
BSCL2 0.515740788 0.374677 0.497731448 -0.162766446
BTBD1 0.959791609 -0.788941753
0.565366777 -0.315374187
BTBD10 0.262848419 -0.501067088 0.510202289 0.279218308
BTBD2 0.429623277 -1.904420578
0.031196176 -1.059502308
BTBD7 0.488853935 1.168784996 0.112490354 0.707482958
BTBD8 0.592998825 -0.452180369
0.894107394 -0.315984603
BTBD9 0.00724674 -1.674432363
0.032000837 -1.035535561
BTC 0.668693293 -1.014389445
0.479090962 -0.343244808
BTLA 0.109340698 -1.469788465 0.409107276 0.25627326
BTN2A1 0.94080765 0.585938966
0.385822695 -0.481171644
BTN3A1 0.218444281 1.389255964
0.173695183 -0.627226403

BTNL3 0.783513907 -0.158673447
0.203892866 -0.126137205
BTRC 0.919375025 0.15440111
0.374788163 -0.716011363
C10orf111 0.898310098 1.085591106
0.239610309 -0.18022624
C10orf32 0.452329763 0.140930387
0.961660291 -0.025258559
C11orf54 0.126782657 -2.120948567 0.761384643 0.112166093
C11orf71 0.955432226 -0.354646606
0.510202289 -0.220838536
C12orf4 0.700272637 -0.506070066
0.863162948 -0.682630764
C14orf1 0.996569936 -1.202673218
0.484447379 -0.599718246
C19orf52 0.735731737 -1.717517708
0.119001281 -0.786804217
C19orf54 0.512212684 0.745105534
0.702590473 -0.136583678
C1D 0.136177865 -4.534075942
0.791934103 -1.242175037
C1GALT1C1 0.664146763 -0.474177223
0.872388894 -0.156806225
C1RL 0.718891763 -2.41827906 0.426434431 0.270613216
C1orf85 0.262515365 0.649997585
0.600764902 -0.284451746
C2 0.727091179 0.143246143 0.908215002 0.109011961
C2orf42 0.093545259 1.490064251
0.581332417 -0.412720571
C2orf76 0.814302165 -0.850173928 0.787119521 -0.30770597
C3orf38 0.779912935 0.379933571
0.005219707 -0.912552467
C3orf43 0.581647753 0.73026174 0.791331842 -0.29732448
C3orf58 0.196480971 1.693055278 0.75306096 0.326254778
C5orf15 0.656502522 -2.927258113 0.688150178 0.524251601
C6orf58 0.283016378 0.226600972
0.157027462 -0.504620517
C7orf25 0.107400696 -0.489102183 0.232536812 0.247310792
C7orf55 0.641601871 0.452841996
0.19892935 -0.133735121
C9orf3 0.580796849 0.335774114 0.880398825 0.344158271
CABIN1 0.225993578 0.655038072 4.77E-05 -2.255593397
CABYR 0.270658738 2.1299371
0.295463669 -0.237573118
CACHD1 0.10242388 -0.967025496
0.939252931 -0.669662244
CALCOCO2 0.440197946 -1.23945488
0.663579309 -0.852772028
CALHM1 0.637689516 0.483240829 0.305312744 0.29365964
CALR3 0.008140018 1.82987255

			0.908215002	−0.079795009
CAMSAP1	0.263515403	−1.968578205	0.978174972	0.345180114
CAND1	0.30308456	0.214832222	0.860967223	0.242670219
CAND2	0.498227149	0.70800924	0.346863262	−0.990580859
CAPN10	0.84538001	−2.273970138	0.493281994	−0.277853012
CAPN11	0.735321968	0.381174884	0.602942066	−0.581917621
CAPN12	0.083187871	1.773102243	0.064240435	−1.589007338
CAPN13	0.045419133	−4.230283456	0.39112285	−0.50711031
CAPN15	0.873004589	−2.117821282	0.0864362	−0.554395635
CAPN2	0.166157552	−0.655534603	0.089638414	0.044553568
CAPN3	0.954186927	−0.55177504	0.875468135	0.019204732
CAPN5	0.794947265	0.598944123	0.988462877	−0.354681229
CAPN6	0.494700441	1.218533923	0.868745142	0.295905395
CAPN7	0.769736031	−0.349793832	0.785917138	−0.313605404
CAPN8	0.727091179	−0.317583197	0.030110734	0.720147345
CAPN9	0.13051809	−1.156502994	0.48493593	−0.283704966
CAPRIN1	0.215533789	−3.428233822	0.016082953	−2.050045608
CAPZB	0.405825169	−1.344816851	0.001591873	−3.135023194
CASC3	0.453741645	−0.796617622	0.359425452	−0.360858179
CASP10	0.307260847	0.575124114	0.460727679	−0.79692216
CASP14	0.099339229	−1.993207059	0.756624802	−0.292672686
CASP2	0.297633754	−0.735708489	0.093239961	−1.482846026
CASP6	0.753654572	−1.805964825	0.247189728	−0.261682311
CASP9	0.749502373	−0.205585489	0.570665194	−0.431198621
CATSPER2	0.222269991	−1.486576434	0.781712835	−0.351348002
CAV2	0.155429772	−1.933234021	0.380705388	−0.2103879
CBLB	0.947962171	0.653440448	0.965398278	0.178725581
CBLC	0.322971313	1.284703032	0.222269989	−0.382407098
CBLL1	0.135357918	−0.653626058	0.620485628	−0.056378726
CBX1	0.124083884	−2.058681406	0.334584156	0.236128761
CBX3	0.376051332	−0.052488179	0.064578819	0.705772326
CBX5	0.791934104	−1.256850295	0.665282244	0.35193721

CBX6	0.140756285	1.288915217	0.742992174	-0.37488759
CBX8	0.493281996	0.993006899	0.289372764	-0.031316368
CCDC115	0.238363179	-3.46843821	0.001562257	-2.96274211
CCDC116	0.531002817	-0.314674919	0.39836184	0.698277576
CCDC117	0.685850081	-0.425157694	0.216985522	0.758593486
CCDC134	0.738268973	-0.575665139	0.182765814	-0.040805226
CCDC22	0.376472971	-0.126300842	0.050094754	-1.081194663
CCDC34	0.890273941	-2.50963581	0.21179205	-0.782629842
CCDC47	0.024603288	-1.062233151	0.219322908	0.266090649
CCDC79	0.945473137	-0.029253469	0.145236129	0.278536977
CCDC80	0.273317984	-1.081712786	0.193786517	0.226990414
CCDC88B	0.338410122	-0.526145543	0.803112258	0.260092309
CCIN	0.975993098	0.48840907	0.357788649	0.215989182
CCL3L3	0.013351707	-1.670167006	0.450685708	0.274919242
CCS	0.038732378	-1.909702575	0.032951337	0.644027324
CCT2	0.02505585	-7.566828565	0.208097488	-6.498098933
CCT4	0.125620497	-4.971850138	0.044435432	-4.037874129
CCT5	0.297996467	1.227665151	0.142663033	-0.482399712
CCT6A	0.073402153	-4.63653698	0.236810715	-3.387201249
CCT6B	0.553794178	-0.789172856	0.281267631	0.245521369
CCT7	0.348468303	-0.390119746	0.69333593	-1.003558382
CCT8	0.548572525	-0.805782702	0.055344174	-0.747314704
CCT8L2	0.386251028	-2.873293242	0.064127966	-1.085871654
CD164	0.84599196	0.725573928	0.882865922	-0.366678308
CD207	0.135767413	0.353452093	0.037283006	0.747134658
CD2BP2	0.31518887	-0.770497673	0.094465962	-1.082020432
CD300LG	0.686424834	0.302807108	0.749502372	-0.433513131
CD63	0.192717308	-1.028103571	0.073151338	-1.507549054
CD68	0.246551772	-1.125959514	0.820982329	-0.061486007
CD74	0.694490284	-0.288586904	0.987839296	-0.617166087
CD81	0.374788165	0.4985182	0.31424829	-0.373526616
CD93	0.292945331	0.556289047		

0.571993482	-0.154980979
CDK2AP2	0.186908087	-1.511436224
0.809149425	-0.345090298
CDK5RAP3	0.901281627	-0.517116902
0.514703007	-0.286972767
CDRT1	0.763171925	0.559279397	0.212078199	0.675132151
CDS1	0.671541069	-0.415580127
0.676678944	-0.359764658
CDY1B	0.869311586	0.144669052	0.855795542	-0.22140826
CDY2B	0.476182966	0.113617984	0.292945329	0.447489916
CDYL2	0.764364149	0.920535304	0.199202762	-0.59489122
CECR2	0.093851358	0.905460626
0.029470774	-1.044120969
CECR5	0.292586748	-1.081861714
0.336545478	-0.807034832
CELA2B	0.004037321	2.246325484
0.008404117	-0.612386963
CELA3A	0.348267415	0.551598058
0.657915441	-0.598408824
CELA3B	0.804609745	0.031924336
0.665850273	-0.158774397
CENPA	0.017364906	-1.476552625
0.000912954	-4.055713015
CENPB	0.538202702	0.546630743
0.002628861	-0.727307059
CENPC	0.001240929	-9.670667355	1.01E-06	-4.889598289
CENPO	0.480231945	-0.990089214
0.048151852	-1.410277219
CENPP	0.869926891	-2.169592553
0.004369017	-2.839967238
CEP170	0.399672858	0.39503394
0.080579688	-1.091132721
CEPT1	0.239610311	-0.269274623
0.166876066	-0.102285028
CERS1	0.102095634	-0.404158511	0.40274197	0.063553604
CERS2	0.361066953	-1.864232104
0.738217952	-0.241111902
CERS3	0.03315909	-2.36062814
0.237378761	-0.429442495
CERS6	0.523785705	0.265734921
0.086088491	-1.388026224
CETP	0.002221465	1.995370038
0.831331641	-0.204714525
CFDP1	0.249271075	-1.22121839
0.174190115	-3.635109232
CFH	0.025810177	1.384608378
0.371844556	-0.222889513
CFHR4	0.769375002	-1.415902558
0.775099683	-0.291468754
CFI	0.704072565	-0.022956668

0.133710105 -0.964629888
CFL2 0.417120215 0.871569239
0.346863262 -0.337347085
CFLAR 0.239922811 0.522089531
0.000912954 -0.648038883
CHD2 0.134338353 1.22464963
0.017163709 -1.401145582
CHD3 0.259701427 0.539745852
0.111581853 -0.320062585
CHD4 0.310504457 -0.533726371
0.002130485 -1.021810381
CHD5 0.186848199 0.614062783
0.269227348 -0.415181106
CHERP 0.740039027 -3.131545427 0.001171557 -3.17218379
CHGA 0.241175686 -1.601356938
0.238363177 -0.852893383
CHID1 0.41931516 1.15521353 0.143409878 0.675024228
CHMP2A 0.002280121 -7.823398887
0.000354732 -4.523420571
CHMP3 0.437425036 0.09782896
0.013439603 -3.982721667
CHMP4A 0.277100619 -2.514572966
0.264518064 -0.229264648
CHMP4B 0.157946003 0.386162968
0.818551649 -0.073782117
CHMP5 0.04654478 1.323434319
0.005871856 -1.138140647
CHMP6 0.026034979 -2.148444508
0.08346635 -2.051557017
CHN2 0.119005904 -0.637018769 0.022968082 0.540096058
CHORDC1 0.966644447 -0.459491887
0.333020413 -0.251700583
CHP1 0.266734942 0.252841577 0.695430457 0.152016947
CISH 0.409366031 -0.287719844 0.312372677 0.599740521
CKAP4 0.257554508 0.412200249 0.452329761 0.592446963
CLCC1 0.839265705 -0.275194462
0.172216692 -1.795269114
CLCN3 0.256405893 -1.015814768 0.104885017 0.473011725
CLCN7 0.052903827 -3.979033978
0.591011931 -0.331765761
CLDN14 0.208606117 -0.935296259
0.458939754 -0.472828161
CLEC10A 0.333020415 0.795026494 0.206408157 0.784887135
CLEC4D 0.885334157 -0.547488178
0.209796823 -0.439565102
CLEC4F 0.429623277 1.455741804
0.236501082 -1.027393543
CLEC4M 0.743583261 -2.020497358
0.430536911 -0.207945534
CLIC4 0.311998443 -1.343851346 0.929929687 -0.20357386

CLN3	0.253484214	−0.59098322	0.135691865	0.515775824
CLN5	0.152271451	1.919589479	0.386679646	−0.818242352
CLN6	0.157256718	1.851933679	0.081396051	−0.557515625
CLN8	0.85089087	0.20824882	0.063123044	−0.481925116
CLPTM1	0.977863266	−3.31871412	0.134135152	−2.064825513
CLTA	0.005637716	−2.798697109	0.009731148	−0.799051235
CLTB	0.521818173	−0.4226588	0.587777197	−0.058491516
CLUAP1	0.573323245	−0.420179547	0.059866884	0.132434862
CLUH	0.061182218	−1.084079749	0.016116807	0.795548668
CNDP1	0.040534555	−0.314299649	0.087444909	−1.457462778
CNDP2	0.973499749	−0.948859367	0.827066508	0.231839834
CNN3	0.063903517	−0.987683044	0.107962092	−0.252906805
CNOT1	0.005428402	−7.598348931	0.348909655	−2.175498202
CNOT10	0.578122472	0.608553196	0.819766773	0.230037534
CNOT4	0.468955478	−1.996863675	0.005557111	−1.011006564
CNPY1	0.052999686	2.190367778	0.409809926	0.259821353
CNPY2	0.568542992	0.72120475	0.661878111	0.203129822
CNPY3	0.248789681	−0.846546957	0.878549262	−0.377741441
CNTD1	0.794947265	1.162444602	0.163306742	0.321428553
CNTNAP2	0.192716368	−1.015817298	0.06224074	0.508845681
COA3	0.148502305	0.345265541	0.298722778	−0.979392542
COG2	0.481035002	0.440814068	0.657915441	−0.256623353
COG3	0.923719449	0.917759795	0.001324846	−1.997017755
COG4	0.532541527	0.054403538	0.267541819	−1.155021151
COG5	0.491458292	0.740069106	0.045954968	0.280661165
COL26A1	0.871157736	0.71947079	0.118451885	0.211882641
COL4A3BP	0.149162221	−1.122456577	0.126006956	0.626956812
COL5A2	0.344863595	0.310631356	0.848440682	0.261349573
COLGALT2	0.647208233	−1.258330214	0.282666039	−1.128104356
COMMD9	0.265523356	−0.992846646	0.046889974	0.759550471
COPA	0.589932779	−3.819511231	0.007972295	−4.909640909
COPB1	0.529466367	−5.469064148	0.015160487	−3.412902096

COPB2 0.007289224 -9.418041576
0.000173108 -6.827101177
COPE 0.148063602 -3.406899988
0.302372056 -1.178988155
COPG1 0.012555539 -5.14588471
0.000597988 -5.267828202
COPG2 0.093698208 -1.156754758 0.391413605 -0.4033653
COPS6 0.564309938 -0.451608103
0.023478783 -1.821236289
COPS8 0.279526237 1.349233984
0.717138908 -0.169278445
COPZ1 0.00439054 -6.890960499
0.028657119 -7.797543155
COQ9 0.971629906 0.359680438
0.813089023 -0.224790105
CORIN 0.036435744 -0.61922195 0.506694058 -0.46779381
CORO1A 0.168574688 -0.740908935
0.140485215 -0.596153697
COX15 0.833771101 0.474213129
0.166396797 -0.712476047
COX6A1 0.472320731 -0.401609138 0.609494757 0.166646406
CPA2 0.596421269 -1.756246579
0.65396236 -0.674009174
CPA3 0.056954699 0.42725292
0.045860802 -1.002309291
CPA4 0.058937356 -1.064190299 0.910424068 0.247370407
CPA5 0.141178304 -1.772786947
0.44951346 -0.496271287
CPA6 0.316886568 2.091090835
0.203059413 -0.355728662
CPB2 0.367264359 2.318157005 0.911313287 0.328136266
CPLX1 0.382832515 -1.271771916 0.958545942 0.092959366
CPLX2 0.105584263 -1.354712372 0.01194453 0.922009694
CPLX4 0.40011044 0.505576692
0.187692421 -0.398910783
CPM 0.295971166 -1.468369948 0.37844494 0.214376522
CPN1 0.091575094 -0.483806296
0.608400445 -0.681023586
CPN2 0.898928707 0.378609898 0.112490354 0.090089707
CPNE7 0.156341221 -1.231975358 0.448109047 0.040125302
CPNE9 0.478605625 0.309626619 0.614429951 -0.14967788
CPQ 0.19459199 -0.823719555 0.984097978 0.530365419
CPT1C 0.653962362 0.197482333
0.509198643 -0.014573211
CPVL 0.004794745 -1.351943667 0.508196031 0.216473251
CPXM1 0.901403753 0.394630039
0.286535927 -0.216929322
CPXM2 0.19191661 0.9100128
0.15520254 -0.664817212
CPZ 0.26485287 -0.454219656 0.180720376 0.277441044

CR1L	0.322015582	−1.168809321	0.540785908	0.409264257
CRABP1	0.350078054	−0.639266462	0.410698577	−0.261263136
CRABP2	0.995946292	−0.538327395	0.119376374	−0.636709113
CRADD	0.168320102	1.130702748	0.237741332	0.221596976
CRBN	0.666418496	0.550547581	0.128934997	−1.04720806
CRCP	0.49229613	−0.329797906	0.76973603	0.292636166
CREB3	0.192716368	−1.011056688	0.025670465	−1.113741998
CREB3L2	0.132920951	1.489060888	0.605941449	0.01166807
CREB3L4	0.075305756	1.111720998	0.061343132	0.709153059
CRELD2	0.971006659	−0.224509559	0.950451714	−0.154391619
CRIM1	0.457518789	−0.208668221	0.004912849	−1.603211942
CRIP2	0.145020358	−0.468160443	0.999688175	−0.336072446
CRISPLD2	0.040229346	1.192842413	0.203059413	0.182601211
CRX	0.509198644	−0.061264901	0.776316677	−0.167275166
CRYAA	0.349675175	−1.232671367	0.037784945	0.758118474
CRYAB	0.107675743	−0.852311558	0.311460951	0.245306438
CRYBA1	0.425525876	0.473859937	0.451859687	−0.218308423
CRYBA2	0.697957588	−1.333532377	0.445307711	−0.346861791
CRYBA4	0.905118113	0.521586535	0.159099891	−0.175604087
CRYBB2	0.002904987	1.507159531	0.249431683	−0.202634297
CRYBB3	0.930550952	−0.129455014	0.836822674	−0.108323378
CRYGA	0.297996467	−0.193761098	0.348468301	0.276696835
CRYGB	0.295824614	−1.081617299	0.917513857	0.250625797
CRYGC	0.656785008	−0.969846575	0.129329364	−0.77338769
CRYGD	0.478184013	0.558975511	0.065763482	−1.400781907
CRYGN	0.225544412	−1.676244866	0.131315269	−0.715221742
CRYGS	0.046803475	−6.020330873	0.004656414	−0.355329287
CSE1L	0.084306322	−5.248571764	0.101604833	−2.955238522
CSPG4	0.327584537	−2.559472285	0.886568693	−1.132896083
CSPG5	0.982227445	0.196597455	0.973499749	0.106922654
CSRP1	0.159995279	−0.624450091	0.008415563	0.703167466
CTBS	0.699693613	0.255659918	0.582404249	−0.204816889

CTRB2	0.949206881	−0.618028218	0.25722611	0.073971265
CTRC	0.871773276	−2.320782294	0.874852134	−0.152928893
CTRL	0.978486682	0.2663549	0.031318829	0.676754463
CTSA	0.022723076	1.519782964	0.139496043	−0.396522774
CTSC	0.231281077	−0.520105048	0.916273329	−0.240163033
CTSE	0.984097978	−0.945998336	0.572791164	0.099154492
CTSO	0.006173644	1.41298508	0.860967223	0.102410911
CTSW	0.887186065	−0.213410451	0.542338801	−0.162833722
CTSZ	0.921857267	−0.553293453	0.022909993	−0.916921718
CTTN	0.839876705	−0.863308009	0.478120554	0.007051748
CUEDC1	0.147652448	−0.738274768	0.03058908	−1.350183471
CUEDC2	0.643281654	−0.709394297	0.41739986	−0.667445439
CUL9	0.373947512	−2.607179386	0.078302716	−0.855078085
CWC27	0.887803504	0.315649546	0.37436769	−0.35244657
CYB561	0.047368134	0.366709018	0.412479308	0.208442469
CYBB	0.115712082	1.646631046	0.229155625	0.256430388
CYBRD1	0.364777485	−0.00544558	0.230064824	−1.002719476
CYP11B2	0.301274174	−0.019026016	0.885334157	0.141341109
CYP26A1	0.693913019	−1.09271537	0.938631108	0.254749861
CYP2C19	0.851503643	0.522801674	0.878549262	0.194672383
CYP2E1	0.094158259	0.796384906	0.370596579	−0.602894518
CYP2F1	0.723573251	−0.654058173	0.367679861	−0.806204823
CYP2R1	0.137208186	1.298627581	0.457992168	−0.479386038
CYP2S1	0.150932936	0.487301409	0.726504454	−0.070377906
CYP2U1	0.100305217	−1.623302831	0.727091178	−0.018913176
CYP3A7	0.456971536	1.272078162	0.889038588	−0.401447846
CYP46A1	0.773323453	0.056653076	0.726504454	0.169905323
CYP4A22	0.234474178	1.109421264	0.707957899	−0.399718437
CYP4B1	0.171971197	−1.866643739	0.236191735	0.746274091
CYP4V2	0.621589448	−0.397666736	0.647769977	−0.357406703
CYP4X1	0.674393569	−0.734893134	0.485913833	0.502011405
CYP4Z1	0.87198974	0.399154517	0.525934692	−0.238181124

CYP8B1	0.334192779	1.149288053	0.735911058	0.231586963
DAAM2	0.177181847	2.446153849	0.800378642	0.490536327
DACT2	0.807577901	0.480760849	0.07839344	-0.872355271
DAD1	0.587238873	-1.00205574	0.009089007	-1.331116668
DAG1	0.569073189	0.332758261	0.046201725	-0.89283423
DAGLB	0.975993098	-0.913439031	0.09648574	0.154520013
DAK	0.023192875	-1.629889213	0.860091711	0.164824698
DALRD3	0.747132935	0.35780843	0.175183138	-0.602146891
DAZL	0.046630877	1.313437804	0.108994053	-0.283145326
DBNL	0.232196258	1.246748712	0.297996465	0.624516618
DCAF13	0.506193921	0.508513988	0.835601747	0.306950391
DCST1	0.394444362	0.140223644	0.952319189	0.262974544
DCUN1D1	0.06687249	-2.134259028	0.088171214	-1.817953596
DCUN1D2	0.153168802	1.476340957	0.135562544	0.701147774
DCUN1D3	0.043128235	0.017223911	0.064918673	0.690143053
DCUN1D4	0.311624503	0.68005238	0.532028371	-0.735310328
DCUN1D5	0.867466147	-0.524628137	0.665282244	-0.149302086
DDHD2	0.298359476	0.127260166	0.034389068	-0.81754944
DDI1	0.54493189	0.40954448	0.656220084	0.400844225
DDI2	0.02118707	-1.746515722	0.466081564	0.031959872
DDN	0.97662845	-0.435843222	0.739982493	0.301843158
DDOST	0.296547384	0.340973318	0.585625267	-1.676960985
DDX31	0.784681667	0.324136319	0.038531007	-0.816582303
DDX39A	0.738268973	0.370975748	0.130717031	-0.297603062
DDX3X	0.316131297	1.645124763	0.142025259	0.327635809
DDX42	0.13192257	1.367684393	0.914412921	-0.256133572
DDX49	0.366019604	-1.16857702	0.183022695	-1.801292556
DDX5	0.852729457	0.640875841	0.649456416	-0.10796677
DDX52	0.648331924	-0.888592923	0.173695183	-1.196432554
DECR2	0.971629906	-0.009628909	0.598048454	-0.43618585
DEF8	0.289728696	-3.848334716	0.061694499	-0.427846161
DEGS1	0.642161595	0.323284196	0.850278187	-0.148827031
DEGS2	0.314624302	0.576916752	0.965398278	-0.088291774
DERL1	0.045330341	-1.639697415		

0.056144628 -1.561600753
DERL3 0.261850131 -0.408067566
0.344065788 -0.220043172
DGAT2L6 0.318400659 -0.824106046
0.842321668 -0.219023382
DHDDS 0.004358292 -3.479981378 2.09E-06 -5.392198377
DHH 0.300383839 -0.183636166
0.05926966 -0.655361247
DHRS1 0.677822753 0.040217676
0.395747589 -0.476054134
DHRS7B 0.893981587 0.478684676
0.285476972 -0.040292296
DHRS9 0.081433011 0.792436753 0.404656279 0.263934652
DHRSX 0.055244791 -0.991068708
0.260192144 -0.953751367
DHX8 0.804609745 2.051090323
0.306790179 -1.692050468
DHX9 2.52E-05 -2.500503372 0.006321489 -1.344651122
DIO1 0.803097973 -0.174081689
0.095549283 -0.571651545
DIO3 0.387108559 0.028750799
0.303105455 -0.515094711
DIP2A 0.316508785 0.045875706
0.687574882 -0.412951594
DLG3 0.775717766 0.519766622 0.174066281 0.522235018
DLX1 0.312747211 1.507470401
0.759003581 -0.239052457
DLX2 0.044352764 -1.595043793 0.427799385 0.060397971
DLX3 0.76675027 0.177887966
0.94858451 -0.676500158
DLX5 0.680685517 0.909537141 0.803400268 0.293521828
DMD 0.928088402 -3.402585901
0.370081682 -0.943221075
DMXL1 0.051483257 -2.157556732
0.157256716 -0.072837273
DMXL2 0.569603622 -1.462328921
0.039474838 -0.995089194
DNAH11 0.10242388 -1.586628772 0.38968811 0.152513819
DNAH2 0.030051396 2.060780785
0.559040051 -0.585371606
DNAH5 0.53837823 -0.746234924 0.248041289 0.690973095
DNAH9 0.14545215 1.151804454
0.937387575 -0.007468816
DNAJA3 0.22375412 -5.920164837
0.069833469 -3.890160957
DNAJB1 0.681831915 -0.388310894
0.361066951 -0.583155199
DNAJB11 0.143945174 0.513509618
0.086292869 -0.381621365
DNAJB12 0.093087614 -1.997775965 0.006276661 0.706364467

DNAJB13	0.399672858	0.03574333	0.114769814	0.202224597
DNAJB14	0.816122712	-0.453053082	0.648331923	-0.12614275
DNAJB2	0.330683662	-0.204699687	0.602152111	-0.233543919
DNAJB3	0.135357918	-1.23340614	0.656785007	-0.043406234
DNAJB4	0.862262629	0.420290123	0.551518382	0.285136127
DNAJB5	0.271269678	-1.070797476	0.441124483	0.221624751
DNAJB6	0.540785909	-0.098565933	0.984721509	-0.084925905
DNAJB7	0.085937852	-3.904797144	0.286748376	-0.63415286
DNAJB8	0.797336501	0.21669283	0.708325269	0.085274113
DNAJB9	0.884099899	-0.215945762	0.504695062	0.234682798
DNAJC1	0.97225317	-0.375808482	0.439272522	-0.097875566
DNAJC10	0.368511748	0.2458165	0.047850386	0.758424261
DNAJC11	0.350078054	0.301852442	0.893981587	0.198038888
DNAJC12	0.836212162	-0.017431788	0.952319189	0.195816639
DNAJC13	0.507695116	0.482474745	0.051764788	-0.518720859
DNAJC14	0.403181568	0.793534696	0.656220084	-0.544805015
DNAJC16	0.854568844	0.408717514	0.860091711	-0.312837689
DNAJC17	0.001278762	-5.203863199	0.227044114	-6.978408922
DNAJC18	0.898928707	-1.035206568	0.905118112	-0.360694102
DNAJC19	0.555365407	-0.183911313	0.58831575	-0.037129355
DNAJC21	0.084728866	-1.553461061	0.784715266	-0.113694059
DNAJC22	0.596421269	-1.789078878	0.11020785	-1.865009469
DNAJC24	0.425980013	-0.360708298	0.018076957	-0.355359554
DNAJC25	0.985968598	-1.138105627	0.8386548	0.461945269
DNAJC27	0.449513462	0.985827904	0.645524201	-0.092166629
DNAJC28	0.372627262	-1.809113622	0.587487036	0.135962494
DNAJC3	0.379008927	0.225510032	0.232654806	0.324624763
DNAJC30	0.530490416	0.977778499	0.653398423	-0.146751074
DNAJC4	0.734144253	-1.174422689	0.308272343	0.349738909
DNAJC5	0.354529147	0.075432098	0.144697157	-0.600161791
DNAJC5B	0.122561976	-1.169883704	0.483959094	0.276082717
DNAJC5G	0.250719163	-1.27649152	0.912552981	-0.0017462
DNAJC7	0.338908808	1.084951912	0.773921829	-0.123190171

DNAJC8	0.508697209	-0.693751225	0.110556604	-0.59794836
DNAJC9	0.013736293	0.90283293	0.890891717	0.257191742
DNAL4	0.481035002	-0.507691796	0.133526983	-0.683202858
DNASE1L1	0.074161081	-1.593859064	0.121995034	-0.90943138
DNASE2	0.367679863	-1.615662012	0.525380245	-0.511643005
DNASE2B	0.714220799	-0.867754145	0.86070579	0.134614807
DNHD1	0.095238754	-2.022000828	0.519281459	-1.018768956
DNM1L	0.805214659	1.690090383	0.971629905	0.222889396
DNMBP	0.68240539	0.30109607	0.206689018	0.045227288
DOCK2	0.385394657	-2.16293537	0.276410225	1.008395551
DOLK	0.956054916	0.423816104	0.961037373	0.177853056
DOPEY1	0.346462741	-1.398366992	0.607853618	-0.634678958
DPEP2	0.331195555	-0.312502132	0.143516815	0.47079631
DPEP3	0.987215722	-0.642199094	0.032823223	0.615969491
DPF1	0.747132935	0.205560787	0.517256682	-0.626846321
DPF2	0.857022589	1.01353456	0.085011511	0.132530897
DPF3	0.727091179	1.552716263	0.153618982	-0.960276852
DPH3	0.264518066	-1.096578984	0.361478059	0.087389381
DPP3	0.939874792	-0.342768631	0.491311319	0.604971664
DPP7	0.119933777	0.656884688	0.108477102	-1.051965416
DPP8	0.438576973	0.540890925	0.034947388	0.901505057
DPPA2	0.423712157	-0.637342865	0.221382903	0.242481372
DPT	0.814302165	-1.55741987	0.407150842	0.144110322
DPYS	0.184569608	1.577445735	0.827066508	-0.021925735
DPYSL3	0.715328142	-1.030963006	0.653294282	-0.368512096
DR1	0.902022666	0.271924702	0.073527822	-1.317901709
DRAM1	0.335760065	-0.19484605	0.291512766	-0.181488856
DRG1	0.090528129	-0.80499248	0.970383429	-0.21132713
DRG2	0.738839936	-0.868134575	0.586175985	-0.211494593
DSCR3	0.254682626	-0.946046294	0.301903967	0.118913715
DSE	0.145190283	-1.754124456	0.431411005	-0.196099139
DTX2	0.10341366	-0.212360736	0.415606578	0.097422762
DTX3	0.145861612	-0.560424728	0.959573771	0.090062112
DUOX2	0.029992158	-3.392292548	0.336152623	0.188739116
DUS3L	0.161426793	-1.316090715	0.098380676	-1.554396685
DVL1	0.901403753	0.481348421	0.207251574	0.31686626

DYM	0.535625701	0.603654342	0.779013423	0.484211279
DYNC1I1	0.558514381	0.290079157	0.528443322	-0.238320701
DYNC1LI2	0.553794178	-2.54397075	0.054063734	-1.327257021
DYNLL2	0.964152194	0.513671203	0.563254049	-0.00460166
DYX1C1	0.554317679	-0.215126052	0.288661787	0.27628279
DZIP3	0.33693863	-0.445526669	0.135357916	0.684480772
EAF2	0.606760622	-1.995233477	0.054553255	-1.117796719
EBP	0.198929352	-0.982859418	0.013005184	-0.548501448
ECE2	0.809452302	0.690132741	0.683552888	-0.129181548
ECEL1	0.680112595	-0.921663157	0.070499122	-1.082073119
ECH1	0.290084921	-0.493135226	0.565366777	-0.320349345
ECI2	0.561475856	0.636366012	0.370081682	-0.329976238
ECT2L	0.322588799	-0.659182886	0.502700232	0.668127755
EDC4	0.17518314	1.552773187	0.270929317	-0.231105097
EDEM1	0.316131297	-0.170627956	0.198383347	-0.997002146
EDEM2	0.234034324	0.009324411	0.129526899	0.454757255
EDEM3	0.983474457	0.884939288	0.971006658	-1.051300398
EEF1G	0.924961137	-0.590030534	0.787119521	-2.541552344
EFNA2	0.97225317	-1.332564894	0.246233223	1.318588996
EFNB1	0.910073809	-0.358571557	0.713637676	-0.081721157
EHBP1	0.405604474	-0.301643497	0.784715266	-0.284407083
EHD1	0.341282759	0.529011745	0.86992689	0.349177902
EHD2	0.33693863	-0.142832097	0.668977855	0.119903409
EHD3	0.474249688	-2.368438488	0.787119521	0.212312266
EHD4	0.862548534	0.419749886	0.840487802	-0.149318903
EIF1	0.837433286	-2.184149424	0.939252931	-1.50899264
EIF2A	0.240235599	0.541814641	0.853955626	0.18097395
EIF2B4	0.050646342	-3.445826332	5.53E-05	-3.687565712
EIF2B5	0.014629657	-1.812933083	0.004455686	-2.61689049
EIF3A	0.051109877	-5.635365081	0.003331082	-6.964587884
EIF3B	0.035195639	-2.849180735	0.34606251	-2.656445849
EIF3F	0.016118813	-2.286681853	0.07307603	-1.885828658

EIF3K 0.15204774 -1.268198544 0.036927963 -0.5511564
EIF4A1 0.559040053 -0.690223956
0.002389289 -1.847932684
EIF4B 0.213181406 1.377627289
0.033421568 -0.718852516
EIF4E1B 0.329907108 -0.46414126 0.042886788 0.370904735
EIF4G1 0.129329366 -1.653738943
0.001081638 -0.920568952
EIF5 0.177432875 -1.298165844 0.34726408 0.176565761
EIF5A2 0.322206581 0.688182383 0.638806307 0.264440118
ELAVL1 0.634344079 0.911520394 0.08515312 -0.99860772
ELP2 0.986592156 -1.673916232
0.875468135 -0.207051115
ELP3 0.3090152 -0.415048124 0.746540947 0.162310427
EMID1 0.753654572 -0.405268662
0.179704054 -0.516724621
EML2 0.545943955 -1.191826344
0.076868684 -0.680286531
ENC1 0.770632428 -0.731866476 0.080174007 1.042075902
ENDOU 0.340490262 1.532029365 0.946095346 0.173646519
ENOSF1 0.367009175 0.584498167
0.259701425 -1.286680157
ENPP6 0.750095103 0.685335239 0.022399156 0.645409554
ENTPD4 0.861319955 0.076145997
0.811270153 -0.031047942
ENTPD7 0.652271143 -0.18196858
0.683552888 -0.485866801
ENY2 0.885951391 0.4523998 0.118821005 0.813886191
EPHX1 0.606214453 -0.591657391
0.039926087 -0.828352089
EPHX4 0.436502971 0.271965709
0.854262224 -0.901170168
EPM2AIP1 0.592091994 1.528173595 0.668124306 0.196867605
EPN3 0.806424836 -0.768552429
0.524870616 -0.112743302
EPS8 0.294202694 -1.001517337
0.62324677 -0.379646804
ERAP1 0.047938511 -1.358329367
0.735911058 -0.540745583
ERAP2 0.280221914 -0.456488936
0.331850707 -0.643846482
ERF 0.849053093 0.635892878
0.044105537 -0.868996392
ERGIC1 0.291155364 -0.165173631
0.926203007 -0.322154227
ERH 0.179450641 0.951500842 0.575987148 0.400171699
ERLEC1 0.560618507 0.32826689 0.512212682 0.184478335
ERLIN1 0.033663639 1.121454843
0.073653668 -0.484804019
ERLIN2 0.214377448 0.373839799 0.076207795 0.269600994

ERMP1	0.805214659	-0.552924484	0.784715266	-0.035014704
ERO1L	0.321443032	0.571227241	0.426434431	-0.596638902
ERP29	0.260192146	-0.490101732	0.467038447	0.324607937
ERP44	0.061368665	-0.70888757	0.265523354	0.193409769
ESYT1	0.18431112	1.279566697	0.626013218	-0.339326112
ETF1	0.421451381	-1.326331299	0.80098273	-0.918362637
ETFB	0.028771142	1.5873735	0.304575799	-0.208505934
EVA1A	0.186908087	-0.931251847	0.0288283	-0.273019677
EXOC3	0.915033006	-0.37993449	9.71E-06	-1.800268367
EXOC3L1	0.213800958	-2.944028848	0.931172259	0.049943607
EXOC5	0.475121086	-1.466145018	0.019979957	-0.484735412
EXOC6B	0.979733555	-1.085870474	0.616078918	0.704915867
EXOC7	0.373947512	0.829115645	0.052999685	-0.84832708
EXOC8	0.119005904	0.979884863	0.167596915	0.16032622
EXTL1	0.721231202	0.836759014	0.851503642	-0.255418325
EXTL3	0.828893806	0.243615248	0.59587932	-0.304826803
EYA1	0.106462311	1.461401402	0.02207926	0.952218978
EZH1	0.175681232	0.680095312	0.832551169	0.42495125
F12	0.159795282	0.483242591	0.731202704	-0.135867481
FAAH2	0.213513133	-0.564464584	0.409809926	0.539334529
FABP1	0.725917893	-0.338270375	0.991580881	0.183210608
FABP2	0.633503673	-1.382900899	0.389262797	-0.270591551
FABP3	0.612234359	0.233932553	0.856409021	-0.070464411
FABP4	0.533054931	-0.751964765	0.554317678	0.109504095
FABP5	0.812482622	0.720066786	0.476666958	-0.142269041
FABP7	0.749439496	-1.512043316	0.444109504	-0.786400952
FADS3	0.989086465	0.383733491	0.596421267	-0.552274471
FAF1	0.201399958	0.533652897	0.391845703	0.130446496
FAF2	0.155657255	-0.317318172	0.176931082	-0.280482894
FAM102A	0.678394935	0.169775663	0.410698577	-0.494233041
FAM120A	0.627121278	0.16096586	0.027703538	-0.840847568
FAM129A	0.578656884	0.401001495	0.16615755	-1.02578854

FAM131C	0.667555512	−0.177116814	0.05524479	0.609760283
FAM132A	0.87423621	−0.435954466	0.210365494	−0.431399723
FAM159A	0.252335026	0.779392291	0.196480969	−0.250305288
FAM192A	0.256898007	0.59445638	0.494804293	−0.35593487
FAM19A1	0.503697127	0.839304378	0.428254934	−1.073698546
FAM210A	0.597505836	−2.079116944	0.462741064	0.048366431
FAM21A	0.160100248	−1.350943066	0.126950493	0.731482402
FAM3C	0.580261511	0.638303212	0.696223141	0.184545594
FAM45A	0.850278187	−0.093084848	0.422806994	0.219413464
FAM63B	0.883482876	−0.456870689	0.252983397	0.198677758
FAM98A	0.598591299	−0.910213381	0.409809926	−0.09592757
FAP	0.145884926	0.460668066	0.014347075	−1.06564171
FAU	0.393143732	0.120623655	0.01081865	−3.15579299
FBXL12	0.916893568	−0.269331841	0.81794425	−0.550395115
FBXL13	0.452445723	−0.606648366	0.146725421	−0.659329222
FBXL14	0.341282759	−0.540927785	0.824023097	0.208744104
FBXL15	0.900166109	−0.231966257	0.86992689	−0.369204084
FBXL16	0.155202542	−1.797104867	0.169045625	0.288045554
FBXL17	0.554841422	0.406201037	0.961660291	−0.566632249
FBXL19	0.269231913	1.115997408	0.203892866	−0.718480014
FBXL2	0.397053419	−0.914343288	0.893363483	−0.266873692
FBXL21	0.581332419	−0.486767372	0.485913833	0.224964923
FBXL22	0.857636242	0.44464179	0.093698207	0.209434706
FBXL3	0.919995513	−0.389116299	0.430994152	−0.563264901
FBXL4	0.337332075	1.071268071	0.077249469	−0.751966631
FBXL5	0.19032442	−3.200097242	0.567483305	0.20596252
FBXL6	0.902641637	0.557562492	0.303840036	−0.155215764
FBXL7	0.94174059	−0.142953399	0.485913833	−0.689901191
FBXL8	0.322206581	−2.780822649	0.03346808	−0.90826857
FBXO10	0.175930674	−0.33867396	0.89150956	−0.019146307
FBXO11	0.370521915	1.352667748	0.162129848	−1.64418068
FBXO15	0.651144656	−1.931325324	0.147844621	0.792959242
FBXO16	0.721231202	−0.497281341	0.327970891	0.543586867
FBXO17	0.143409879	2.462664595	0.038292622	−0.799017219
FBXO18	0.236501084	1.45381171	0.847216136	−0.27236786

FBXO2	0.847216137	0.321622767	0.194323225	0.485656883
FBXO21	0.019172885	-4.020356926	0.434202714	0.565269424
FBXO22	0.53024615	-1.515904932	0.542740776	-0.13525187
FBXO25	0.341282759	-3.133735315	0.626567142	-0.254741998
FBXO27	0.549614908	0.385825862	0.420549053	-0.355968328
FBXO28	0.278138407	-1.936590962	0.036505716	-2.060776799
FBXO3	0.856409022	0.052177142	0.051389698	0.754410352
FBXO30	0.086436202	-3.763621048	0.969137025	-1.316225185
FBXO31	0.450451123	-1.348185216	0.580261509	0.480213271
FBXO32	0.478120556	-1.289375243	0.473284667	-1.065103894
FBXO33	0.97139972	0.219004529	0.95891701	0.13394979
FBXO34	0.240235599	0.365577979	0.354123029	-0.708812118
FBXO36	0.63702957	0.613285997	0.135562544	-0.454349913
FBXO38	0.212364628	1.799077876	0.799170824	-0.228716382
FBXO39	0.794947265	-0.034314358	0.108477102	-0.704447792
FBXO4	0.089343413	-1.143106526	0.955432226	-0.104810581
FBXO40	0.643841988	1.706740951	0.553270918	-0.251434991
FBXO41	0.359015813	-0.883527549	0.655655357	-0.210052038
FBXO42	0.316131297	-0.582615338	0.011014152	-1.678518053
FBXO43	0.664714408	-2.141998083	0.048381179	-1.528692693
FBXO44	0.19191661	0.884405203	0.078038315	0.810864636
FBXO45	0.913792889	-0.777736717	0.002957263	-0.786775213
FBXO46	0.637131428	0.084470426	0.825240147	0.057188516
FBXO47	0.970383429	-1.761766056	0.019496487	-1.269774891
FBXO48	0.81066409	-1.954532513	0.8386548	-1.068361288
FBXO6	0.59317296	-1.41745802	0.652271141	-0.099510638
FBXO7	0.16807878	1.261221978	0.232807803	-0.536280649
FBXO8	0.084587828	-0.791907715	0.000710157	-1.778688762
FBXO9	0.841710285	0.462347483	0.555365405	0.084541999
FBXW10	0.025515601	1.165132995	0.051670801	1.120904533

FBXW12	0.437425036	0.117387063	0.218444279	0.568078682
FBXW4	0.435582027	-0.738020234	0.050924037	-1.245402265
FBXW5	0.280221914	-1.247251958	0.754248327	-0.60252546
FBXW8	0.031565376	-1.578884406	0.745357421	0.401679226
FBXW9	0.82584883	0.260661705	0.809452301	0.232417853
FCGRT	0.01465168	1.240552134	0.141571123	0.669221422
FCHO1	0.203183632	-1.741484988	0.024562545	0.545012953
FERMT2	0.918134197	0.469325329	0.270929317	-0.918005601
FETUB	0.075049637	-0.502783761	0.379432604	0.525813256
FFAR2	0.404502077	1.10116171	0.537686802	-0.997256564
FFAR3	0.530490416	-0.518165782	0.023192874	-0.218259625
FFAR4	0.868081213	0.531965744	0.938009323	-0.445440033
FGF10	0.21991007	-3.34704508	0.481035	0.259862834
FGF3	0.130120911	0.480413894	0.556938808	0.170547881
FGF8	0.170260044	-0.28153604	0.022630114	-0.918125153
FGFBP3	0.241803848	0.931572601	0.952319189	-0.112419567
FGL1	0.182042511	0.84349345	0.217600084	0.344671429
FIG4	0.587777199	-0.485124446	0.041150865	-0.531479137
FIGNL2	0.263515403	0.355552498	0.822198316	-0.22564869
FITM2	0.530490416	-0.435351806	0.808846578	0.204358767
FKBP10	0.194054737	-1.082488611	0.946717589	-0.193376046
FKBP11	0.491311321	0.471540888	0.614429951	-0.059786695
FKBP14	0.164015965	0.347731654	0.588854532	-0.08330562
FKBP15	0.043533198	0.352268298	0.555365405	-0.504693191
FKBP1B	0.14545215	0.628034878	0.144159719	-0.308310509
FKBP2	0.306420379	0.993937766	0.518268559	-0.159623947
FKBP3	0.256898007	-2.163119615	0.026723815	-0.843416825
FKBP4	0.226147852	-1.119625747	0.565092855	0.125614995
FKBP5	0.037929409	-1.397641133	0.564309937	-0.177453699
FKBP6	0.679963147	0.315396296	0.161102885	0.385740191
FKBP7	0.31991948	-0.25542507	0.933657903	-0.68948355
FKBPL	0.129329366	-0.7344085	0.76555693	-0.084036152
FLG	0.340094456	0.482526135	0.906356698	-0.164830889

FLOT1 0.472802564 0.534438797
0.467756823 -0.911823959
FLOT2 0.632916829 0.602816429
0.217419386 -0.342140008
FLRT2 0.059443447 -1.511673258 0.703460376 0.008650783
FMNL2 0.070925425 -1.925406213
0.953564319 -0.642766491
FMO1 0.496245929 0.292658176
0.056144628 -0.565528588
FMO2 0.13741497 -0.486192345
0.218444279 -0.411489218
FMO3 0.709560519 1.860738818 0.842933148 0.227481711
FMO4 0.570134291 -0.462161444
0.60130886 -0.317997398
FMO5 0.858249981 0.35914113 0.968513851 -0.16648286
FNDC3A 0.13051809 -1.480086856 0.201399956 0.725061705
FOLR1 0.075562587 -1.312470267 0.043451948 0.792002441
FOXA1 0.232807805 1.030380046
0.107790853 -1.041230238
FOXA2 0.562031588 0.542386801
0.181230135 -0.281369693
FOXA3 0.688150179 -1.333969827 0.134135152 -0.26821219
FOXF2 0.626567143 -0.083141426 0.899547377 0.03812064
FOXRED1 0.590472242 -0.238051577 0.532541525 0.200524673
FRMD6 0.345106225 -1.508428651 0.618280557 0.004225506
FSIP1 0.811270154 -0.738526817
0.434202714 -0.339690899
FTSJ1 0.415158972 0.099734049 0.191650565 0.483382424
FUCA1 0.827675504 -1.339027654
0.504195964 -0.291252126
FUNDC1 0.268335375 0.261399485
0.892803901 -1.492138446
FUT10 0.994075379 -1.347009971
0.000438701 -0.611390233
FXN 0.345662578 -0.451564791
0.978486682 -0.165870216
G2E3 0.134338353 -0.952558531 0.052903826 0.206301052
G3BP1 0.933657903 0.361198941
0.008822694 -0.624739872
GABARAP 0.302372058 -0.623345961 0.177432873 0.156024952
GABARAPL1 0.769736031 -2.66573733
0.275032962 -1.24592142
GABPB1 0.401424912 -1.011112341
0.942362595 -0.268266337
GAL 0.750095103 -1.46611678
0.214955056 -0.465618879
GALC 0.840487802 0.134513341 0.059654853 0.295508022
GALNT15 0.980980477 -1.834921414
0.560355279 -0.195588039
GALNT2 0.283367009 -1.235049561

0.563254049 -0.362873908
GAN 0.005045798 -1.234803693
0.002846276 -0.955175823
GANC 0.532541527 -0.581218465
0.111081368 -0.794186445
GAPVD1 0.251091535 1.014921053
0.605122774 -0.078882873
GATAD2A 0.278138407 1.184678573 0.197838441 0.181108992
GC 0.600221168 -1.140753681
0.252983397 -0.801386562
GCN1L1 0.405825169 0.336557105
0.927445058 -0.209823251
GDAP2 0.263849332 0.516468926 0.566953817 0.442764711
GDI2 0.313122038 -2.081915867
0.263181763 -0.269053567
GDNF 0.658952025 0.277860455
0.374498611 -0.223754878
GDPGP1 0.484935932 -0.551515629
0.292945329 -0.641276232
GFPT1 0.464171071 -0.390505481 0.022909993 0.616435046
GGA1 0.666986909 0.263679855
0.509198643 -0.218767145
GGA3 0.060079541 0.577726443
0.149382687 -1.027211174
GGH 0.209796825 0.198886082
0.397528194 -0.325044972
GGN 0.252011274 0.231812617
0.59371378 -0.007346819
GGT2 0.111767427 -0.71650027
0.121162384 -0.656150004
GGT6 0.502700233 0.401634392 0.563254049 0.183240159
GHSR 0.163773961 -0.477362131
0.731387525 -0.132845335
GID4 0.606760622 0.111662739 0.460838218 0.146046488
GIMAP1 0.389262799 0.012330526
0.672128879 -0.470889321
GIPC1 0.68355289 0.535657573
0.790729706 -0.546722392
GIPC3 0.365191231 1.761791781 0.86992689 0.081097176
GJA3 0.713054721 0.776698267 0.488363252 0.204225936
GJB1 0.699693613 -0.325170573 0.285476972 0.787542685
GLB1 0.999688175 0.044363611
0.354529145 -0.111249642
GLB1L 0.280918766 0.672077886
0.881015491 -0.231227536
GLCE 0.471839167 -0.880415299
0.002601941 -0.744378467
GLE1 0.772127103 -0.860054609 0.995946292 0.201155924
GLI2 0.171480988 -3.240674532
0.114002801 -0.860255005

GLRA4	0.102752966	-3.658875562	0.291512766	0.818783137
GLTPD2	0.759598634	-0.184545492	0.291155362	0.095113109
GLYCTK	0.14762589	-1.174568176	0.856409021	0.49567027
GMCL1	0.001562257	-1.595977953	0.974123063	-0.674016167
GMPPB	0.633230587	0.159113141	0.716554954	0.35465241
GNA11	0.377317124	-1.199879365	0.013886796	-1.366570216
GNA13	0.94858451	-1.442108128	0.132618729	-0.280748826
GNAI2	0.199202764	0.149737745	0.359015811	-0.049047399
GNAI3	0.35371721	0.322638659	0.244961924	0.591582545
GNAO1	0.031196177	-0.968688563	0.767347146	0.231177225
GNAQ	0.834381217	-0.453497039	0.063345227	-1.292499287
GNAZ	0.872388894	-0.147563433	0.092328865	0.211582188
GNB2L1	0.445773908	-1.063244715	0.895836284	-0.210063522
GNL3L	0.070438396	-3.821476933	0.003143081	-2.109831153
GNPNAT1	0.642721523	-1.859335171	0.163306742	0.0666067
GNPTAB	0.969760218	0.583030156	0.027869802	-0.887040468
GNPTG	0.691029361	0.905092562	0.98971006	-0.476642032
GNS	0.221382905	0.475207434	0.254283622	-0.238010604
GOLGA1	0.816729781	0.194731961	0.016048563	-1.005455779
GOLGA2	0.333020415	0.805102631	0.26384933	-0.726104121
GOLGA3	0.423775309	1.719027785	0.269227348	0.132717981
GOLGA4	0.228550917	0.552137693	0.98877467	0.197674796
GOLGB1	0.695067727	0.282695714	0.728852306	-0.327560604
GOLIM4	0.87916571	-0.160260568	0.063456561	0.423272665
GOLPH3	0.817336961	0.478903231	0.245279316	0.69323957
GOLT1A	0.168803525	0.901436559	0.131515153	0.088942204
GORASP2	0.828284604	0.564957701	0.400548307	0.168365164
GPC2	0.14202526	1.351670967	0.682979047	-0.317965439
GPD1L	0.644402524	-0.522488188	0.388827099	-0.421620479
GPIHBP1	0.262515365	1.78936238	0.492788929	-0.45862625
GPNMB	0.417848894	-4.042819653	0.044187817	-0.925224421
GPR108	0.654018711	-0.701968798	0.602526529	-0.081900931
GPR119	0.84599196	0.039268784	0.835601747	0.374269139

GPR26	0.839876705	-0.513725802	0.375629983	0.387457551
GPR37	0.482495845	0.148118365	0.5382027	-0.464845727
GPR50	0.354450314	-1.973410276	0.451752227	0.758893307
GPR89B	0.57598715	-1.805635069	0.339698943	-0.369093904
GPRC6A	0.681171517	-2.1261286	0.867447919	0.580346895
GPSM1	0.92558205	0.353197183	0.817336961	-0.238686813
GPSM2	0.893363483	0.16587209	0.063903516	-0.6286889
GRB14	0.814908903	0.583614363	0.019455778	-0.483377811
GRIP1	0.663579311	-0.207407152	0.285829662	-1.206627603
GRN	0.048648425	1.490827519	0.831331641	0.216665518
GRPEL1	0.151378108	-0.930724457	0.750095102	-0.076974153
GRPEL2	0.631561906	0.345211844	0.627675621	-0.154389922
GSAP	0.70491108	0.422444935	0.143730871	-0.50154662
GTF2E1	0.214089063	-2.611835606	0.799170824	-0.132305174
GTF2F2	0.385822697	-0.449600341	0.023145518	-0.40699834
GTPBP10	0.501704383	-0.492840638	0.911933107	-0.113608794
GYS1	0.371850989	0.696768093	0.300543725	-0.514234109
GYS2	0.809452302	0.403151589	0.519281459	-0.198800502
GZMA	0.962283233	-0.006337591	0.036857301	-1.206340212
GZMH	0.352312749	-2.184850772	0.97139972	-0.21299148
GZMK	0.688663224	-1.141182846	0.347071031	0.172380421
GZMM	0.270589253	-0.717088223	0.853342497	-0.183307094
H2AFZ	0.000202822	1.733085746	0.1746861	1.165654043
H2BFM	0.061296296	1.088014286	0.144043112	0.26864348
HAAO	0.766153531	0.29843615	0.935522562	0.204271231
HABP2	0.089196206	-1.547893889	0.598591298	-0.123051206
HACE1	0.002075372	-1.49860567	0.249753118	-0.277479985
HAND1	0.389257462	-2.593902733	0.555365405	0.294431889
HARBI1	0.306420379	-1.170336325	0.149603404	0.775738445
HAS2	0.449982154	0.667235717	0.592632362	0.203744458
HAUS3	4.53E-06	-7.328458271	0.000197864	-3.086948927
HAUS7	0.014099945	-3.84239404	0.000266447	-5.241663906
HAX1	0.670971135	0.612723561	0.167596915	-0.183642249

HCCS 0.795550266 -0.332299886 0.893981587 -0.04466146
HDHD3 0.800378643 -0.747701537
0.117167044 -0.547358638
HDLBP 0.782313064 -1.052450138
0.351288456 -0.421435743
HEATR3 0.759598634 0.574831141
0.436042357 -0.213106743
HECTD1 0.24211836 0.925255009
0.088317057 -0.554202332
HECTD2 0.767347147 -1.171303992
0.463217459 -0.755071452
HECTD3 0.897691551 -0.491895937 0.943606709 0.158815143
HECTD4 0.106483865 -0.672638569
0.203317422 -0.298755825
HECW2 0.112844795 -0.371364924
0.252983397 -0.714748188
HERC1 0.385394657 -0.590302704
0.026192563 -1.406917336
HERC3 0.065146062 1.28573147
0.165918563 -0.565427729
HERC4 0.35371721 -0.068568727
0.010250523 -1.026752328
HERC5 0.246551772 -2.287877966 0.631561904 0.416645949
HERC6 0.721231202 -0.498689602 0.146535935 0.625241987
HERPUD1 0.038732378 0.172834183 0.938009323 0.233928981
HERPUD2 0.045185313 -3.439075158 0.298722778 -0.46808427
HES1 0.162778597 1.160946175
0.378501591 -0.664824489
HESX1 0.189795858 -3.176903109 0.243695237 0.237931459
HEXA 0.329907108 0.984383414 0.324120627 0.214579358
HFE 0.916893568 -0.814898676 0.099499708 -1.00665391
HFM1 0.376051332 0.834203474
0.377317122 -0.536136509
HGD 0.417848894 -1.765422328
0.52640026 -0.187432995
HGFAC 0.096016599 0.701932802
0.092935464 -1.296597614
HGSNAT 0.602726301 0.337082981
0.292756117 -0.703983461
HIGD1A 0.075305756 1.228163171
0.209796823 -0.339349689
HILPDA 0.775717766 0.829353814 0.624352715 0.082057767
HIST1H1D 0.127757501 -2.396076577 0.408036058 0.262885002
HIST1H2AA 0.404502077 -1.427147175 0.791331842
0.008208204
HIST1H2AB 0.011593818 -2.169431129
0.638806307 -0.651381439
HIST1H2AC 0.13196425 1.609806953 0.084870093
0.630258816
HIST1H2AD 0.036505717 0.598964507

0.18174096 -0.771296607
HIST1H2AE 0.223456728 0.099714049 0.57652063
0.059207447
HIST1H2AG 0.933441493 -0.075047524
0.346494805 -0.379471087
HIST1H2AH 0.068755355 2.174532662
0.939252931 -0.554356254
HIST1H2AI 0.970383429 0.519993956
0.115712081 -0.364737204
HIST1H2AK 0.430536913 0.752959769 0.816729781
0.159666856
HIST1H2BA 0.804609745 0.307292442 0.720646095
0.202594378
HIST1H2BB 0.125620497 2.14369405
0.04451823 -0.585553201
HIST1H2BC 0.084870094 1.845808304
0.558514379 -0.023634634
HIST1H2BE 0.314060398 -2.131485241
0.329131734 -0.319788028
HIST1H2BF 0.287597534 -0.56732046 0.817336961
0.340679373
HIST1H2BG 0.067105501 -3.82019882 0.539751881
0.171660671
HIST1H2BH 0.787119522 -0.638152744 0.171971195
0.583946299
HIST1H2BJ 0.559255589 0.69536243 0.331651969
0.333387278
HIST1H2BK 0.303676621 0.454226458
0.297316845 -0.200993751
HIST1H2BL 0.738217953 -0.941281719
0.200404915 -0.701702205
HIST1H2BM 0.529978266 0.660071229 0.549093593
0.428894691
HIST1H2BN 0.833161085 -1.923927841
0.950451714 -0.618327289
HIST1H3B 0.921541453 -2.560796873
0.101339444 -0.287084091
HIST1H3C 0.799774674 -0.665067762 0.532541525 0.159721137
HIST1H3D 0.348066602 0.598964507
0.134949375 -0.867991612
HIST1H3F 0.22206474 -3.969770062 0.384780578 0.625948814
HIST1H3G 0.899547378 -2.724824805 0.207815239 0.711484732
HIST1H3H 0.491803593 0.331206632 0.505194421 0.234904668
HIST2H2AB 0.239298097 -2.183424995
0.097587538 -1.191491993
HIST2H2AC 0.932414999 0.799743408
0.307901358 -0.334516591
HIST2H2BE 0.091274973 1.42506276
0.145020356 -1.031698776
HIST2H2BF 0.34927259 -3.307751062 0.36177913

0.732053046
HIST2H3C 0.585625269 -0.984306032
0.69304745 -0.191564604
HIST2H4A 0.240548674 -1.925407326 0.360245614 0.328978825
HLA-C 0.86869636 -0.590447816
0.95294174 -0.319132361
HLA-DMA 0.390119051 0.200578067 0.857022588 -0.00081762
HLA-DMB 0.729439669 -3.201913148 0.531002815 -0.38680671
HLA-DOB 0.367264359 1.055036406
0.603486911 -0.533440464
HLTF 0.128149068 -0.607175983 0.227044114 0.54429997
HM13 0.579191528 0.368295512
0.670971134 -0.017428649
HMBOX1 0.627121278 -0.550831878
0.759598633 -0.017300841
HMGCS2 0.302005801 0.785692356
0.539751881 -0.669574803
HMGN1 0.304575801 -0.584836562
0.04272646 -0.629443116
HMGXB4 0.52793218 0.087444802
0.011762468 -1.194329197
HMHA1 0.233502514 -1.741862126
0.920232687 -0.409200764
HMP19 0.185606245 -1.751125903 0.633230585 0.06117841
HN1L 0.852729457 0.416294625
0.195939895 -0.229709096
HNF1B 0.313872578 1.190200623
0.209229265 -1.202480062
HNRNPC 0.781112739 0.008080941
0.007400729 -1.322063892
HNRNPF 0.259852852 0.632601128
0.148305141 -0.313210539
HNRNPH1 0.021055987 -5.481595363 1.70E-05 -1.812294112
HNRNPH2 0.592632364 0.070650948
0.67610732 -0.232206899
HNRNPK 0.008113468 1.589080081
0.009417007 -0.630767928
HNRNPU 0.598591299 -1.478930378
0.001003701 -3.107239261
HOMER1 0.669831834 0.376326719
0.37394751 -0.127763103
HOOK2 0.751874178 -1.459943996 0.179704054 0.137911059
HOXB1 0.785917139 -1.33549701 0.650018963 -0.20668115
HPR 0.55746376 -0.290414074
0.496740838 -0.149922368
HPS1 0.677822753 0.182765374 0.190324418 0.613376924
HPS4 0.698536088 -1.410390131
0.650581709 -0.625144859
HPS6 0.210081021 1.437966159
0.937387575 -0.254331658

HRC	0.702590474	-0.104476221	0.618831503	-0.25435777
HRG	0.390226856	-0.164824331	0.601308533	0.26032879
HS2ST1	0.725917893	-1.070485285	0.084587827	-0.806691111
HS3ST3B1	0.725917893	-0.251045076	0.379856574	0.272133311
HSBP1	0.632674152	-0.36650086	0.178818262	0.520771337
HSCB	0.0041385	-3.277341159	0.000370952	-1.729896127
HSD17B13	0.945473137	-0.387942254	0.562462756	0.296541469
HSDL1	0.387323123	-0.389258718	0.074539532	-1.180421315
HSF1	0.150045586	-2.515854158	0.267879253	-2.557895169
HSF2	0.997193581	-0.226079628	0.710142463	0.487214488
HSF2BP	0.14202526	1.226967143	0.069053447	0.63205727
HSF4	0.055743205	-1.46866891	0.630450492	-0.153241249
HSPA12A	0.190451073	-1.87775686	0.530246148	0.293896339
HSPA12B	0.884716993	-0.776315844	0.034455674	-0.589450447
HSPA13	0.179450641	-2.17342239	0.463217459	-0.714882311
HSPA14	0.904498906	1.185098489	0.635458398	0.057754307
HSPA1L	0.11625602	0.398104772	0.360656136	0.18892332
HSPA4L	0.735364621	0.227332369	0.069651417	-0.339290202
HSPA6	0.34927259	-2.146254578	0.420549053	-0.674022752
HSPA9	0.245279318	-3.847216536	0.000138061	-4.535204478
HSPB11	0.68240539	-2.358754273	0.325272603	-1.091315857
HSPB2	0.157256718	-1.674183345	0.14437451	-0.556766018
HSPB3	0.897691551	0.018954567	0.124658384	0.589099539
HSPB6	0.262182602	-0.028844667	0.740629351	-0.315274696
HSPB7	0.501704383	-0.602695992	0.871157736	-0.238777684
HSPB9	0.92806615	0.155085136	0.911933107	0.220782372
HSPBP1	0.554317679	-2.018255614	0.24655177	-0.622656598
HSPD1	0.009101286	1.185349527	0.060980393	-0.306560325
HSPE1	0.697957588	0.412422872	0.629895099	0.247067374
HSPH1	0.803400269	0.017083394	0.775717765	-0.132005693
HTRA1	0.697379264	0.498200929	0.301274171	-0.438125469
HTRA2	0.804609745	-1.939829152	0.958545942	0.377746789

HTRA3	0.874852135	-0.368645517	0.1825092	-0.344287196
HTRA4	0.197023139	0.352688732	0.342473715	1.282037428
HTT	0.298359476	-1.5518171	0.426889133	0.30615569
HYAL1	0.125576585	-1.767484761	0.324459912	-0.05583887
HYAL2	0.348468303	-0.911547408	0.402302663	-1.892181534
HYAL3	0.000846372	-1.543069094	0.774520341	0.223639531
HYOU1	0.846604002	-2.515426851	0.025310374	-0.949963513
IAH1	0.994075379	-0.668128516	0.582404249	-0.324958977
IAPP	0.265523356	-0.31975861	0.098062805	-1.020884919
ICA1	0.289366445	-1.03488222	0.178194499	-0.825691136
ICMT	0.046201726	1.424677521	0.895217988	-0.416198257
IDS	0.328744493	-0.73559938	0.688437892	-0.208115186
IFI30	0.633230587	0.460036647	0.59587932	0.102445389
IFI35	0.429166881	1.571797396	0.02905791	-0.653251463
IFI44L	0.94485096	0.923815179	0.01504649	-0.788087133
IFIT1B	0.348066602	0.375745152	0.033925902	-0.560569243
IFIT3	0.004077577	1.292147272	0.615529046	-0.145999591
IFITM3	0.840293912	-0.620309657	0.4266464	-0.471487998
IFNA10	0.484502379	0.470718091	0.526943549	-0.036700462
IFNA13	0.715387545	0.487728372	0.252983397	0.236102525
IFNA14	0.323737228	-2.581547665	0.969137025	-0.78792206
IFNA16	0.801586937	-1.991299487	0.903879758	-0.417834381
IFNA17	0.271269678	1.396248417	0.472320729	-0.305029477
IFNA21	0.29474267	-0.287003894	0.728852306	0.047841191
IFNA5	0.567483307	-1.908409415	0.006442445	-0.651401792
IFNA6	0.696898325	-1.563882473	0.608564858	-0.73700766
IFNA7	0.954809563	-4.344721643	0.76973603	0.21217949
IFNA8	0.535111047	-0.350135024	0.192182922	-0.702470936
IFT122	0.523249163	1.453212593	0.139352265	0.623344037
IFT172	0.889038588	0.417398133	0.130717031	0.731443332
IFT20	0.289728696	0.599745943	0.044023388	-0.196123389

IFT52	0.209512907	−0.56448723	0.657350126	0.721101227
IFT88	0.587777199	0.369672234	0.172708473	−0.253180559
IGFBP4	0.734733032	0.654157644	0.409809926	−0.674713171
IHH	0.208637379	−0.021417958	0.905198285	−0.609502062
IK	0.071916481	−2.450215927	0.067926307	−2.746566966
IKBIP	0.740629352	0.63339898	0.429623275	−0.121680087
IL15RA	0.780883099	0.084597198	0.006906399	−1.511475916
IMMP1L	0.306050873	−0.651249376	0.820374497	−0.707010407
IMMP2L	0.084597377	−8.305448083	0.119709398	−0.801828016
IMP4	0.098380677	2.59899448	0.073527822	−1.129531133
ING2	0.395965049	−0.446861488	0.686999766	0.012428047
INSIG1	0.431451676	1.127042497	0.132518111	−0.221706163
INTS9	0.000479545	−8.396669415	0.007452707	−6.088099653
INVS	0.325272605	−1.19153602	0.021943382	0.224833909
IPO9	0.490327571	−0.89019174	0.874852134	0.156130314
IPP	0.050003315	−1.349851632	0.966021352	0.20289342
IQCB1	0.135210313	0.934227969	0.356381104	0.992412841
IQGAP2	0.453270741	−0.909937491	0.045606503	−1.022775739
IQUB	0.169045627	0.139686986	0.574388104	0.572559084
IRF3	0.217568183	0.186305451	0.159563229	−2.01356761
IRF8	0.266531274	2.008178159	0.621589446	−0.197305269
ISG15	0.722401901	−0.091497672	0.935522562	0.48100672
ISL1	0.538718848	0.506733013	0.808846578	0.107412001
ISL2	0.517762494	−0.458804718	0.214377446	0.618177381
ITCH	0.184828365	1.70622186	0.02391338	−0.249651139
ITGA6	0.786518267	0.328316786	0.957923147	−0.090923738
ITGBL1	0.472320731	0.288349112	0.936765865	−0.291869661
ITIH1	0.897691551	−0.356118237	0.545451244	−0.362080269
ITIH3	0.564814229	0.585382559	0.958260277	0.194312866
ITIH4	0.458465825	0.351662472	0.375629983	0.470910741
ITSN1	0.582940512	−2.697562818	0.030468885	−1.194239476
IVNS1ABP	0.125620497	−1.295138678	0.334192777	0.04344947

JAG1	0.790729707	-0.842206158	0.365605269	-0.413502605
JHDM1D	0.938320211	-0.360459128	0.403621448	-0.659880593
JOSD1	0.071415212	1.684731832	0.033208848	-1.053002858
JTB	0.601308862	-0.50069883	0.962283232	-0.816062462
KANK3	0.299086379	0.198303569	0.307901358	-0.338177071
KBTBD11	0.005557112	1.121170607	0.211220591	-0.332001709
KBTBD12	0.119005904	-1.738703868	0.004771434	0.765717139
KBTBD13	0.455628014	0.723847891	0.108994053	0.158914886
KBTBD2	0.165441365	-2.384207558	0.379432604	0.603120476
KBTBD3	0.405476023	0.219380317	0.294676496	0.272229202
KBTBD4	0.408187556	-0.144996466	0.410058585	0.206271142
KBTBD6	0.10933977	1.435052268	0.097587538	-0.474778895
KBTBD7	0.232501889	-0.555751741	0.943917759	-0.071252159
KBTBD8	0.194054737	-1.402949436	0.183537261	0.429929182
KCNE2	0.423259435	-0.476771545	0.416054472	0.357921141
KCNG3	0.938631109	1.347298148	0.856409021	-0.356744135
KCNIP2	0.198960137	-1.3178416	0.980602164	-0.181766417
KCNIP3	0.87423621	-1.06621314	0.069352606	0.173690988
KCNRG	0.46799642	-0.362542774	0.272634042	-0.666121847
KCTD1	0.860705791	0.298517549	0.800378642	-0.0477724
KCTD10	0.392710767	-1.491677455	0.002221465	-5.051615145
KCTD12	0.130319383	2.147492293	0.149603404	-0.324044173
KCTD13	0.308048674	-0.572668183	0.078947311	0.405091535
KCTD14	0.162282629	0.820931239	0.98980719	-0.172899685
KCTD15	0.168561683	-0.320630187	0.050461916	-0.815874151
KCTD16	0.366019604	-0.796732399	0.048292371	-0.994417472
KCTD17	0.439272524	-0.847986834	0.870542274	-0.891334311
KCTD18	0.322588799	-2.958785037	0.160555719	-0.803922202
KCTD19	0.120373005	1.440931368	0.059654853	-1.382888317
KCTD2	0.716554956	0.295234324	0.255588491	-0.350577705

KCTD20	0.721231202	−0.483587189	0.76555693	−0.250483973
KCTD21	0.895217988	0.376483875	0.586700775	0.529299394
KCTD3	0.65904666	−0.049750412	0.009026921	−1.510784138
KCTD4	0.164252887	0.109801527	0.302738608	−0.98034117
KCTD5	0.738217953	0.598073683	0.53132681	0.098326167
KCTD6	0.053481155	1.741294186	0.367679861	−0.345461183
KCTD7	0.448109049	1.221272705	0.01580964	−0.556302447
KCTD8	0.833771101	−0.483305816	0.086006783	−0.305416419
KCTD9	0.785315264	−0.376667178	0.35146001	0.583975627
KDELC2	0.512715927	0.972307116	0.176680583	−0.241495011
KDELR1	0.876084211	−0.582597672	0.240235597	−0.485362113
KDELR3	0.180211685	1.472249988	0.409809926	−1.139521922
KDM1B	0.127757501	−0.655166692	0.851503642	−1.05302486
KDM2A	0.095393918	1.608967505	0.185346681	−0.350155352
KDM2B	0.533054931	0.53177154	0.628784941	−0.997954805
KDM4C	0.377317124	−0.576562051	0.697379262	−0.260935925
KDM6A	0.375629985	0.119678593	0.664714407	−0.343960412
KDM7A	0.771529132	0.882727468	0.103082891	−1.230082293
KEL	0.997817228	−0.326438406	0.06050673	0.273938802
KHSRP	0.131515154	1.431118909	0.142663033	−0.343605508
KIAA0101	0.334975832	0.642595207	0.377739636	−0.266542649
KIAA0141	0.419647865	−0.608096577	0.819766773	−0.27570964
KIAA1109	0.02505233	2.068823655	0.059232661	0.001551084
KIAA1161	0.190854066	−1.117286792	0.109859966	−0.777799777
KIAA1279	0.990957269	0.833781916	0.730027188	0.218492467
KIF1B	0.523343253	−0.309730774	0.277100616	0.021831727
KIF1C	0.28618265	0.31413169	0.42507202	0.069719429
KIF20A	2.36E−05	−9.856141271	0.00010978	−6.51687318
KIF3A	0.442516386	1.419967382	0.894908864	0.060987318
KIF4A	0.002725096	1.887638802	0.661878111	−0.78180649
KIF5C	0.200573948	−0.517774446	0.100628871	0.681925278
KIF7	0.81855165	−0.088138851	0.53923524	−0.102846987
KIFAP3	0.854568844	0.292061684	0.503198548	0.22814747

KIFC3	0.227097669	-2.349717986	0.860319661	-0.372039175
KLC1	0.012012873	1.87850411	0.870542274	0.235949425
KLC3	0.644402524	-1.123724058	0.344863592	-0.661889413
KLHDC3	0.785316139	-0.229171034	0.724745248	0.44981105
KLHL1	0.524361243	-0.601025538	0.865006686	-0.054999198
KLHL10	0.700562214	-0.718148052	0.839876705	-0.030384014
KLHL11	0.859477716	0.304216679	0.078567847	-0.903814442
KLHL12	0.658480953	0.754898465	0.062460354	0.668613412
KLHL13	0.019619063	-4.512573244	0.00864243	-1.170027808
KLHL14	0.852116505	-0.725854877	0.829503111	-0.093698958
KLHL15	0.873886677	-0.605950432	0.929965495	0.01273071
KLHL17	0.171725962	0.233174437	0.037497424	-0.949449629
KLHL18	0.216403988	1.433811046	0.819766773	-0.566862153
KLHL2	0.226143443	1.493572134	0.471357873	-0.461441714
KLHL20	0.717723029	0.485227342	0.040305464	0.683363468
KLHL21	0.668124307	0.090635139	0.632117923	-0.327806033
KLHL22	0.478363056	0.335462	0.956677633	0.119820516
KLHL23	0.025567133	-2.208228752	0.485424749	0.291769825
KLHL24	0.377739638	1.039659699	0.072279006	-0.246853736
KLHL25	0.06126037	-0.559880991	0.019415142	0.127796406
KLHL26	0.381980793	0.657795498	0.060828772	-0.775550479
KLHL28	0.444376157	0.517796483	0.335760063	-0.43829417
KLHL29	0.416502652	0.045413232	0.817336961	-0.067400889
KLHL3	0.958545942	-0.348139439	0.150710723	0.302351298
KLHL30	0.409366031	1.259129157	0.617454539	-0.068961716
KLHL31	0.390667738	-2.881655998	0.189615132	-0.888669771
KLHL32	0.297996467	1.179037397	0.470396101	0.205424453
KLHL33	0.743583261	-1.719704164	0.347665193	-0.250835826
KLHL34	0.396617854	0.374117529	0.825240147	0.090151979
KLHL36	0.246551772	-1.930581517	0.911313287	0.179934643
KLHL38	0.855795542	0.413258878	0.513723184	0.212692139
KLHL40	0.954186927	-0.410998548	0.094158257	0.847396254
KLHL41	0.920616049	-0.754225109	0.535625699	0.39328938

KLHL42	0.073527823	1.340544424	0.000358991	-1.028096457
KLHL5	0.056632881	-1.303077765	0.074281795	-0.904706491
KLHL6	0.258870999	0.881858502	0.545451244	0.11250016
KLHL7	0.884099899	-0.479373217	0.17705643	-0.479180522
KLHL8	0.163542893	0.706346308	0.30753067	-1.002473367
KLHL9	0.819159157	0.828163332	0.431909478	-0.260532004
KLK11	0.510987006	0.747149538	0.305023497	-0.096633847
KLK12	0.211792052	-1.573243973	0.768541313	0.01700285
KLK13	0.153168802	-0.63164388	0.457518787	-1.322110484
KLK14	0.092631767	-1.995614597	0.532028371	-0.22502639
KLK15	0.878549263	-0.037932427	0.735321966	-0.02533888
KLK2	0.735911059	0.379114038	0.650018963	0.147102398
KLK4	0.770931295	-0.327716658	0.8337711	-0.020868157
KLK5	0.3090152	-0.432714451	0.200024648	-0.159836719
KLK8	0.127366866	2.052226269	0.119933775	0.238980648
KLK9	0.384966907	-0.376185524	0.097271718	-0.55261669
KLKB1	0.047019977	-0.482888743	0.763171924	-0.17584488
KRT1	0.898310098	0.472621372	0.693913017	0.088703343
KRT5	0.211792052	1.352457868	0.189005042	-0.356629862
KRT6A	0.65478125	0.565120845	0.095852633	-0.537266699
KRT73	0.106937883	-1.374038781	0.655090828	-0.08051142
KRT78	0.077511694	1.782132961	0.140125073	-0.604355582
KRTAP10-9	0.457045682	-1.904991489	0.164490065	-0.612841
KRTCAP2	0.229458409	0.585281289	0.027047012	-1.121627934
KTI12	0.68419602	0.293450708	0.98980719	0.239573612
LACTB2	0.801586937	1.610574678	0.001084629	0.951279599
LAMP2	0.900166109	-2.670198961	0.01650071	-0.834685114
LAMP3	0.934279416	0.959383548	0.713637676	-0.396218813
LAMTOR2	0.310504457	0.75770083	0.004067502	-0.896275725
LAPTM4A	0.983474457	0.147839558	0.066062191	0.335753043
LAPTM5	0.521310321	0.170574911	0.538718846	-0.19545198
LBP	0.722401901	-0.820205473	0.334584156	1.06910909
LBR	0.197294633	1.140237733		

0.490819312	-0.120896676
LCAT	0.740629352	0.785980634
0.118913425	-0.955859472
LCE3B	0.802795707	-1.517954086	0.66301205	0.336542778
LCLAT1	0.11427136	-1.387072372
0.628784941	-0.114295704
LCN1	0.428254936	0.167742326	0.076727191	0.290113757
LCN12	0.751281006	0.206124593
0.050738765	-0.816415816
LCN15	0.28866179	1.21699571
0.1825092	-0.503792398
LCN6	0.102752966	-3.118590342	0.019455778	0.634137958
LCN8	0.003627798	-2.452967337	0.280918764	-0.56510183
LDLRAP1	0.667555512	0.124693352	0.234341666	0.598235647
LECT2	0.513108718	0.84140338
0.001272013	-1.289987747
LEFTY2	0.229644412	-1.68422081
0.592998823	-0.632060369
LEP	0.404502077	1.215490759	0.113378101	0.47481351
LGMN	0.350481227	1.193218797
0.221678316	-0.319757213
LHX2	0.089049194	-0.845177552	0.101441651	0.193949371
LHX5	0.635458399	-0.264865678	0.639365011	0.389086335
LIM2	0.210365496	0.646281551
0.284420665	-0.179652412
LIPC	0.026034979	-0.690363816
0.320109663	-0.357333835
LIPE	0.713637677	-1.052532575	0.662444984	0.329941756
LIPH	0.761384644	-0.723244883	0.382406506	0.231821801
LIPI	0.018644966	1.771770383	0.3775696	0.472896594
LIPK	0.40054831	-0.443519059	0.183279845	0.146026228
LITAF	0.06062045	-0.901778911
0.147342941	-1.025097151
LLGL1	0.274003097	0.586865756
0.747132934	-0.372032377
LMAN2	0.967267562	0.504074537	0.923098674	0.177279065
LMAN2L	0.934900969	0.267505801	0.7224019	0.218614415
LMF1	0.597505836	-1.688495403
0.133122723	-0.165834811
LMNA	0.218736876	-0.941096263
0.967890697	-0.363108305
LNPEP	0.41025411	2.133317881
0.850890869	-0.245218064
LNX1	0.729439669	-0.729654618
0.470396101	-0.581540364
LONP1	0.893363483	-6.210125017
0.868696359	-3.632032406
LONP2	0.242748248	-1.325913767	0.929929687	0.10440578
LONRF1	0.384539448	-0.641132584
0.792536488	-0.022062709

LONRF2	0.429623277	−0.448386052	0.7212312	0.015050847
LONRF3	0.059337977	−1.542003178	0.849665594	−0.129203504
LPAR3	0.279723022	0.844425469	0.086844899	0.824623503
LPAR4	0.727091179	−1.622477482	0.19892935	−0.141944094
LPCAT3	0.397053419	−0.208042236	0.228853128	−0.868296187
LPCAT4	0.103082892	1.406487741	0.299450271	−0.62376474
LPHN1	0.537834151	−2.19306339	0.624142058	−0.447109424
LPIN1	0.902022666	−3.568764954	0.890273941	−0.857430208
LPIN3	0.045017758	−0.403398735	0.190060002	−0.335921369
LRIF1	0.421451381	−0.816019804	8.42E−07	−2.178303392
LRIT1	0.424618451	−1.498349437	0.269570807	−0.497234551
LRMP	0.073847754	−2.497931971	0.151517143	−0.371354782
LRP10	0.619934041	−0.702479999	0.193518571	0.432772513
LRP12	0.046717109	0.820262191	0.665282244	0.083676935
LRP3	0.374055459	−0.801492072	0.411934703	−0.303091421
LRP4	0.15407017	1.673049353	0.676678944	0.249106648
LRP8	0.006706415	−2.724624762	1.37E−06	−2.505494355
LRPAP1	0.639365012	−0.262265864	0.439272522	0.522320662
LRRC14	0.397925412	−0.452234997	0.666986907	−0.231415271
LRRC27	0.072776433	−1.353214133	0.725917891	−0.236321846
LRRC29	0.416054474	0.483077703	0.613331721	−0.826126142
LRRC34	0.860705791	0.335212819	0.340886361	−0.676500441
LRRC45	0.471839167	−1.27451323	0.208945904	−1.378906813
LRRC48	0.397053419	0.472871898	0.900784901	−0.046073146
LRRC59	0.40011044	0.377874695	0.077511693	0.019909105
LRRC8A	0.105413877	−4.822745039	0.024748603	−1.838296005
LRSAM1	0.013351707	−2.123355441	0.177432873	0.213717125
LSM14B	0.656220085	0.261104708	0.341282757	0.302431817
LTBP4	0.010296832	−2.335106689	0.045017757	0.45117208
LTN1	0.905118113	−0.603385754	0.436502969	−0.814234857
LUC7L3	0.144374512	−0.535551134	0.319539329	−1.880321081
LY75	0.942984634	0.35401086		

0.137829256 -0.658867966
LYPLA1 0.39401053 0.805575894 0.76973603 0.411562125
LYPLA2 0.119005904 0.390866687
0.248469111 -0.353368086
LYSMD1 0.642161595 -0.66803036 0.152944087 0.604401494
LYSMD3 0.450920368 -0.730119519
0.492788929 -0.188781551
LYST 0.416054474 -0.912175806
0.923098674 -0.235949229
LYZL2 0.231585852 0.587466665
0.692182288 -0.195518901
LZTFL1 0.87916571 0.861182435 0.05524479 0.466748754
MAF1 0.796153389 -0.38130482 0.152944087 0.427015296
MAG 0.395747592 -0.475622182 0.153844449 1.17981456
MAGEA11 0.356768036 -0.411496766
0.062901504 -1.224160676
MAGT1 0.951696668 0.464683406
0.416951112 -0.035599341
MAL 0.053012917 -1.486342779 0.365260612 0.506397611
MAL2 0.457045682 -1.28735474
0.400110438 -1.065789024
MAMDC4 0.721231202 -2.489892063 0.037283006 0.665360518
MAN1A2 0.738268973 -0.060743968
0.965398278 -0.243424024
MAN2B1 0.044850726 0.961599524
0.911933107 -0.165247783
MAN2C1 0.023341793 1.076329618
0.521105815 -0.397026408
MANBA 0.263849332 0.242967575 0.541820923 0.300117691
MANF 0.322206581 0.904232126
0.074921843 -0.758605853
MAP1LC3B 0.173201305 -1.301692074
0.453741643 -0.340799851
MAP1LC3C 0.000145217 2.308084435 0.004725121 -0.94644807
MAP4 0.165447001 0.902677516
0.050590432 -0.442534243
MAPK1IP1L 0.711306862 -0.108495117
0.170991825 -0.351547835
MAPT 0.072403102 -1.455831362 0.083327016 0.734579383
1-Mar 0.433284578 1.214762716
0.072155085 -0.240513394
10-Mar 0.407593308 -1.359474248
0.091338478 -1.476260553
11-Mar 0.391413607 0.487419982
0.045775904 -0.830081767
2-Mar 0.087589784 -0.993318944
0.151824278 -0.145644218
3-Mar 0.682979048 -0.984697232
0.320680662 -0.935035584
4-Mar 0.912552982 0.658756766

			0.342473715	-0.269883764
5-Mar	0.227044116	-0.548723084	0.583208725	0.217447981
6-Mar	0.684701121	-0.395410562	0.328744491	-0.661860646
7-Mar	0.10341366	-0.856264036	0.632117923	-0.075515314
8-Mar	0.027155492	1.798787384	0.141389677	0.425807925
9-Mar	0.06457882	-3.205924564	0.788924047	-0.215216053
MASP1	0.221974013	0.949368691	0.839265704	-0.266948594
MATN2	0.469435415	1.992696604	0.425980011	-0.289743733
MBD6	0.889038588	0.482512011	0.126394342	-1.852669517
MBLAC1	0.055843336	-0.835445615	0.12581361	-0.69893421
MBNL2	0.473284669	-0.358025054	0.060936434	0.850859152
MBOAT2	0.121995035	1.070770289	0.58831575	-0.24709972
MBTPS1	0.910693521	0.403198802	0.450451121	0.248646186
MBTPS2	0.152719625	0.278553436	0.168078778	0.101893015
MCOLN1	0.841098996	-3.220636343	0.94485096	-0.070765634
MDGA1	0.844156391	-2.080170137	0.641042349	-0.008870178
MEFV	0.45799217	-2.771423635	0.559565962	0.265755804
MEI1	0.000928394	-1.403578132	0.910073808	0.095727879
MEIS1	0.483959096	0.283347512	0.82280647	-0.124401165
MEOX1	0.569073189	0.265653534	0.242748246	-0.429049386
MEP1A	0.751281006	-0.39919761	0.462741064	-0.906969295
MEP1B	0.337725816	0.607882992	0.928687285	0.181922186
MESDC2	0.070195912	-1.306392603	0.126782655	-0.721396393
METAP1D	0.006996472	1.539370513	0.529978264	-0.160798933
METRNL	0.46083822	0.074817858	0.966644446	-0.08593316
METTL1	0.660178657	-3.002949546	0.524870616	-0.282924388
METTL21B	0.920616049	-2.209886364	0.506694058	0.355041989
METTL6	0.474491111	-0.065795368	0.483959094	0.454303707
MEX3A	0.904498906	0.457852576	0.982850945	-0.281942111
MEX3B	0.788924048	-0.548533039	0.518774881	-0.569153439
MEX3C	0.632117925	0.54364899	0.65396236	-0.209425544
MEX3D	0.316508785	0.273328183	0.896454644	-0.011899119

MFGE8	0.584013726	0.829611973	0.664714407	-0.075270214
MFSD11	0.647064116	0.321655555	0.174011721	0.321443478
MFSD7	0.17939061	0.840726096	0.190270168	0.153846435
MFSD8	0.847828364	0.352487003	0.753654571	0.156662123
MGAT2	0.791934104	-0.032660268	0.217859933	-0.068044555
MGAT4B	0.643841988	1.091824326	0.043533197	-0.736646185
MGLL	0.812482622	-0.667518812	0.406708664	-0.515910492
MGP	0.36395087	-2.351964228	0.928687285	-0.142483693
MGRN1	0.49229613	0.57483222	0.657350126	-0.223493282
MIB1	0.460363192	-0.639306044	0.178187546	-1.517015733
MIB2	0.599677656	-0.403681055	0.578656882	-0.578523613
MID1	0.066450024	-1.030820487	0.262945005	0.207279376
MID2	0.595879322	-0.84560116	0.528443322	-0.344920236
MIDN	0.175930674	1.407586117	0.413817855	-0.076879046
MIOS	0.498876687	0.382936715	0.04624449	-2.698603842
MIPEP	0.240548674	-2.284041673	0.327198474	0.258781484
MITD1	0.284420667	0.726610551	0.023145518	-0.924038979
MKKS	0.426167101	1.173897529	0.432422486	-0.126126412
MKRN1	0.596421269	-0.161027578	0.412033697	0.136855898
MKRN2	0.702590474	0.276121284	0.979733555	0.227655829
MKRN3	0.699693613	0.093035924	0.162599835	0.111793606
MLEC	0.272095333	-2.230958114	0.248041289	-0.372345034
MLST8	0.161192952	-0.263385833	0.689301311	-0.178823059
MLX	0.374367692	-2.641123077	0.123892841	-0.686998248
MLYCD	0.960414479	0.113035502	0.21179205	-0.719488497
MMD	0.71713891	-0.316231501	0.889038588	-0.258126257
MMEL1	0.931793608	0.027764805	0.825240147	0.036276749
MMP23B	0.22315962	-1.367045405	0.158868604	-0.943994245
MNDA	0.767629791	0.262150144	0.727051909	-0.482210689
MOCS1	0.505194423	0.44062718	0.884716993	-0.30006791

MOGAT1	0.026510208	1.724113313	0.280918764	-0.645325468
MOGAT3	0.731202706	0.398783529	0.263515401	0.371215275
MOGS	0.820070622	-0.402558139	0.825240147	-0.532611322
MON1A	0.647208233	-0.871609834	0.23189091	0.40363538
MON2	0.034389069	-2.817775716	0.715971166	0.069701169
MORF4L1	0.573855558	-0.381164283	0.131115621	-0.793614678
MORF4L2	0.228248989	-3.345673944	0.020932576	-0.685854225
MOSPD2	0.848440683	-0.519765324	0.147298253	0.487818486
MOXD1	0.187954408	-0.507157257	0.917513857	0.178971937
MPDU1	0.452800114	0.704016521	0.782781735	0.262360103
MPLKIP	0.200299162	1.844114424	0.018345667	-0.644502518
MPND	0.715387545	0.690117262	0.923098674	-0.175450028
MPV17	0.124764814	1.111535684	0.133122723	0.777201323
MRC1	0.887186065	0.682383664	0.033729041	-0.578577444
MRM1	0.959168763	-1.085735441	0.744174496	-0.371659789
MROH7	0.000382148	2.134368596	0.589393541	0.199967153
MROH8	0.982850946	0.211669142	0.298541088	-0.452416225
MRPL51	0.97534323	0.268348237	0.063615073	-1.784941939
MRPL54	0.670971135	-0.166396108	0.108477102	-0.123616164
MRPS18B	0.929308465	0.466360051	0.041540076	-2.346779171
MRPS28	0.178691989	-1.383304001	0.183022695	-0.51077767
MRVI1	0.689652881	0.178600672	0.397271306	-0.973701898
MSH4	0.931172259	-2.61872603	0.278831733	-0.373418837
MSL3	0.297633754	0.454399781	0.311250857	-0.774474154
MSR1	0.519639455	0.693346575	0.13492631	-0.339585992
MSRB3	0.008722137	-1.169213432	0.05328813	-0.768566932
MSTN	0.667025594	-1.362454095	0.035397883	-2.535235569
MSTO1	0.78025051	-2.656309585	0.060388592	-0.438016291
MT1E	0.059337977	-1.471511978	0.647208231	-1.01469736
MT3	0.02905791	-2.154663775	0.55484142	-0.028799276

MTCH2	0.728852307	−1.095838691	0.414264608	0.105571255
MUC16	0.828284604	0.375009311	0.991580881	0.258815787
MUC20	0.193518573	−0.35674312	0.319539329	−0.669287186
MUL1	0.331461398	−0.438591379	0.527932178	0.245548668
MVD	0.002227267	−5.901783905	0.003183108	−6.279277656
MYH10	0.974746393	−0.214321594	0.490327569	0.061102968
MYH2	0.01584358	1.165915202	0.285631258	−0.214403343
MYH3	0.48542475	0.375736545	0.543893915	−0.482254357
MYH4	0.525722572	0.532840685	0.162205788	−0.354361099
MYH7B	0.583477004	−1.307191979	0.704330668	0.010070035
MYL12B	0.46799642	−0.591998989	0.185866074	0.626969603
MYL6	0.317643022	0.568864179	2.86E-05	−2.177744202
MYLIP	0.493281996	0.088357039	0.490327569	−0.019643938
MYO10	0.55693881	0.529821673	0.033663638	−1.184855359
MYO18A	0.342076436	−5.128930703	0.052903826	−0.532858035
MYO1A	0.170260044	0.269583085	0.221087772	−0.72247764
MYO1C	0.068755355	−0.782878394	0.421902967	−0.316465218
MYO1E	0.145020358	1.502088052	0.121618213	0.703158229
MYO1F	0.289728696	0.306920451	0.555365405	−0.357838069
MYO5B	0.316886568	−0.906806188	0.033925902	−0.590315915
MYO5C	0.902641637	0.483287277	0.67610732	−0.334911291
MYO7A	0.791331843	−0.142874167	0.781112738	−0.272336292
MYO7B	0.477635755	−0.608270963	0.552620469	−0.289686403
MYO9B	0.461313521	0.942427622	0.000706122	−1.755385133
MYOC	0.035811114	−1.089579155	0.206970157	0.712361248
MYRIP	0.171480988	0.363777223	0.871773276	0.712599126
MYSM1	0.778114213	0.336047214	0.024503686	0.395287127
N6AMT2	0.404502077	−0.710389248	0.665850273	−0.336094004
NAAA	0.470876853	0.469699818	0.382832513	−0.428761518
NAALAD2	0.994699014	−0.204176684	0.071047611	−0.812587771
NAALADL1	0.373170124	0.309996544	0.273176197	−0.519742209

NABP1 0.179704056 -2.09753299
0.059337976 -0.819267632
NACAD 0.70433067 -0.649705624
0.598048454 -0.787368664
NALCN 0.079702812 -3.302507924
0.450920366 -0.443697898
NANOG 0.669831834 -0.633359214
0.923719449 -0.133623366
NANS 0.764364149 0.978546627 0.847216136 -0.19361597
NAPA 0.008065089 -1.474044055
0.941118621 -1.301888336
NAPG 0.847828364 -0.808188038
0.222269989 -2.376041118
NAPSA 0.761384644 0.897169141
0.868696359 -0.565126018
NCCRP1 0.270589253 -0.509688641 0.245120583 0.765887076
NCF2 0.058374255 -1.205727985
0.146954039 -0.578411281
NCF4 0.515740788 0.343908651 0.913172908 0.052568908
NCKAP1 0.713054721 -0.531890334
0.473767041 -0.140295231
NCSTN 0.808846579 -1.267009443 0.640483032 0.33701684
NDP 0.995946292 -2.857008026 0.556414099 0.222161689
NDRG4 0.365605271 0.711971691
0.708978742 -0.160835966
NDUFAF2 0.700272637 -0.610397838
0.332630216 -0.205930156
NECAP1 0.526400262 -0.261543903 0.153618982 0.353210097
NEDD4L 0.339698945 0.708716953
0.586700775 -0.088877777
NEDD8 0.532541527 -2.922161815
0.326042064 -3.210648209
NELFA 0.170016639 -2.718046729
0.982850945 -1.270890889
NEU4 0.077262871 1.176191052 0.173201303 0.302727269
NEURL1B 0.739448856 0.462444434
0.104745175 -0.232836724
NEURL2 0.368511748 0.316751998
0.80098273 -0.140228943
NEUROD1 0.494762776 -0.50045915
0.608400445 -0.164287114
NEUROD2 0.171127948 -1.271007034
0.372727942 -0.222508084
NEUROD4 0.584550677 0.363633422
0.516751127 -0.822896649
NEUROD6 0.560618507 0.440651365 0.450451121 0.744112377
NEUROG1 0.862548534 0.183499538
0.306790179 -1.020734774
NEUROG2 0.204171238 0.611159176
0.584013724 -0.347570372

NEUROG3	0.479090964	1.447137923	0.449045045	0.320605014
NFAM1	0.879782232	-0.511287354	0.014347075	-0.827624855
NFATC2IP	0.440197946	2.216102295	0.212364626	-0.209311042
NFATC3	0.76496047	0.595948046	0.09648574	-0.659378283
NFE2L2	0.258657579	0.844421877	0.529166603	0.209086949
NFU1	0.104577996	-0.064752661	0.573855556	-0.363688962
NFX1	0.887186065	-0.532433751	0.274003095	-0.387703744
NFXL1	0.876700362	0.294516282	0.507194456	-0.652924884
NFYC	0.001282254	-1.73320598	0.267204678	-0.152092744
NGDN	0.071169973	-0.662947893	0.076597072	-1.541460356
NGFR	0.747725071	-1.145795255	0.82280647	-1.955489071
NHLRC1	0.377528344	-0.39840294	0.143089435	0.018116178
NKTR	0.062131174	-2.287348271	0.799774673	-0.419020884
NKX2-2	0.936144194	0.321276675	0.107278426	-0.766290459
NKX6-1	0.303840038	-0.472112685	0.003832394	0.727676629
NLGN1	0.63824781	0.722598877	0.697379262	0.210297074
NLN	0.682979048	0.460072189	0.1100338	-0.699026475
NLRP14	0.760193829	-0.193192568	0.569603621	0.182700604
NMU	0.017736543	1.404935629	0.162599835	1.160891093
NOB1	0.16212985	-1.541748464	0.164727502	-1.964196834
NOL8	0.046717109	-3.810529786	0.074921843	-3.892930221
NOMO1	0.014989441	-0.945583219	0.061368664	-4.511728979
NOMO2	0.898928707	-2.437818565	0.048115167	-0.381916611
NOMO3	0.245596999	-0.964395201	0.153168801	-3.943844696
NOP14	0.001896318	-6.973103768	0.000223206	-2.752308906
NOP58	0.141813156	-2.733448456	0.829503111	-1.698232233
NOSIP	0.934279416	0.452359941	0.888421012	-0.008612754
NOSTRIN	0.722401901	-0.340751926	0.846604001	0.098309459
NOX1	0.747132935	-0.628612937	0.746540947	-0.272105988

NOX4	0.172708475	0.205097638	0.041979833	-0.493411095
NOXA1	0.171236277	0.714689345	0.034322573	-0.802181194
NPAS4	0.005334347	1.92073036	0.734144252	0.252034792
NPC1	0.75484223	-0.23468395	0.841098995	-0.497505474
NPC2	0.973499749	-1.03404345	0.56854299	-0.809448374
NPEPL1	0.950451714	-0.145266884	0.605122774	-0.623564316
NPEPPS	0.35534226	-0.87429477	0.150488761	-1.310052781
NPHP3	0.041540077	-0.540256507	0.902641637	0.11758872
NPHS1	0.91627333	0.081924889	0.7212312	0.390252858
NPLOC4	0.407593308	1.996490731	0.178439634	-0.180428983
NPTX1	0.007400729	2.237720859	0.291155362	-0.190321859
NR1D1	0.145884926	-2.27552956	0.834381216	-0.31602853
NR2C1	0.808846579	-2.39287787	0.093087612	-0.420925796
NR5A2	0.468475813	-0.877505925	0.409809926	0.431037446
NRD1	0.147189167	1.825042826	0.151824278	-0.683380884
NRIP2	0.00594237	-2.165701243	0.556938808	-0.644877885
NRIP3	0.798567097	0.513664526	0.65396236	0.38269484
NRROS	0.399235565	0.32080596	0.373108025	0.292463998
NRSN1	0.601853042	-2.228656087	0.407593306	-0.570148153
NRSN2	0.146971177	1.353141813	0.032378179	-0.661657359
NSFL1C	0.72533149	0.098164429	0.323737226	0.110291752
NTF3	0.072776433	-1.563833401	0.292228458	0.655135382
NTPCR	0.611137865	0.30329932	0.067749728	-1.200700427
NUB1	0.46799642	0.532374254	0.863162948	-0.16136067
NUCB2	0.902022666	0.568473155	0.25986142	0.220247582
NUDCD1	0.145668415	1.57781271	0.814302165	-0.195732007
NUDT16	0.103082892	1.415999135	0.240548672	-0.98299945
NUDT7	0.272804918	-2.709375936	0.483959094	-0.19590481
NUP210	0.822806471	-0.453725336	0.940496688	-0.463725333
NUP35	0.009109787	-5.2410597	0.123130972	-2.330588759
NUS1	0.812049823	-0.286868083	0.17792896	-0.931334482
NUTF2	0.11038212	-3.509297336		

0.046630877 -2.523225864
OAF 0.295463671 0.321595987 0.703170365 0.228517923
OAS2 0.849665595 0.087703843 0.055046467 0.370119761
OASL 0.192182924 -1.169288274 0.642721522 -0.298288
OGN 0.046561604 -4.18927114 0.033860936 0.701349663
OGT 0.341679451 -1.131104811
0.217859933 -1.414102571
OLA1 0.432367565 0.78700111
0.059022496 -0.781236493
OLFM1 0.800378643 -0.9619172 0.757813905 0.237587058
OLR1 0.931355738 -0.284741485
0.280456973 -0.184841942
OMA1 0.875468135 0.047127014
0.737089705 -0.179137816
ONECUT1 0.247828844 -1.246483015 0.361478059 0.095752774
OPA1 0.738268973 -0.229667448
0.261517949 -1.454141663
OPTN 0.198484115 0.897624134
0.288661787 -2.424054943
OR10AG1 0.134921026 0.557162535
0.052687084 -0.477461767
OR13G1 0.926203008 -1.046510963
0.688150178 -0.347305978
OR2A1 0.65537642 -0.342222429 0.264611262 0.545978625
OR2A42 0.629986081 -0.282064235 0.644106004 0.650719672
OR51S1 0.890891718 0.83158034
0.682979047 -0.199404059
OR5J2 0.290798255 -0.260068904
0.41515897 -0.104520496
ORC3 0.763767967 -0.646595296 0.532541525 0.621643168
ORMDL2 0.140967173 -2.979233725
0.358606463 -0.284251042
ORMDL3 0.383258812 -3.776160925
0.004067502 -0.937115293
OS9 0.95231919 1.458572748 0.931793608 0.175616584
OSBP 0.086006785 -1.643008059
0.440197944 -0.407765503
OSBP2 0.452800114 0.152929715
0.016784482 -1.175312652
OSBPL10 0.816122712 0.801742077
0.466559869 -0.342676562
OSBPL11 0.202505159 0.369231144 0.600764902 0.259769604
OSBPL3 0.929308465 0.963210338
0.285124575 -0.568649717
OSBPL5 0.003079991 -3.766163102
0.338119849 -0.212182103
OSBPL6 0.924961137 -0.248506665
0.239298095 -0.320674479
OSBPL7 0.838654801 0.122707492
0.929929687 -0.218456884

OSBPL8 0.007677018 1.966432077
0.083851992 -0.582034177
OSBPL9 0.166636304 -2.575631422
0.029173304 -0.891540774
OSGEP 0.516751129 -0.553526233
0.187430706 -0.525553853
OSTC 0.763171925 0.698030583
0.023574763 -1.874062823
OSTM1 0.126394344 0.556432934
0.319159476 -0.886908617
OTUB1 0.135357918 1.146547901
0.181485414 -0.746013119
OTUB2 0.477151223 1.124747485 0.521310319 0.421567715
OTUD1 0.430994154 0.480843438 0.291870464 0.020486776
OTUD4 0.518268561 -1.893084612
0.106767931 -1.461845883
OTUD5 0.82159027 0.074729143
0.582404249 -0.434427512
OTUD6B 0.643841988 0.208670012
0.445773906 -0.433047337
OTUD7A 0.419197696 1.554783168 0.136589273 0.315945214
OTUD7B 0.22315962 1.620004144
0.558514379 -0.109105961
OTX1 0.926824011 -0.847380163 0.250074843 0.502837878
OTX2 0.164846319 -1.677579856
0.690453164 -0.049268659
OVCH1 0.276410227 -0.822049605 0.300543725 0.531961618
OVCH2 0.639365012 -1.482968272
0.170991825 -1.127421568
OVOL1 0.297271336 -1.280242163
0.903879758 -0.175478131
OXNAD1 0.193786519 0.572113144 0.799170824 0.128422527
P2RX1 0.013380949 -3.572117275
0.428254934 -0.286130727
P2RX4 0.169773495 -1.524044046 0.302005799 -0.28219192
P4HA3 0.260854466 -0.369428921
0.432825929 -0.404639039
PAAF1 0.376894902 0.454836971 0.604031978 0.181698182
PABPC1 0.259861422 -1.082209075
0.927755598 -0.283179963
PACS2 0.303840038 -0.273488259
0.249753118 -0.946112818
PAFAH2 0.804004948 -0.133514815 0.415606578 0.20462173
PAMR1 0.280221914 -1.342809877 0.333410906 0.137877179
PAN2 0.12389299 1.715404294 0.184946013 0.62595874
PAOX 0.035811114 0.767213853
0.520802722 -0.229837267
PAPPA 0.345262939 -0.605765934
0.39314373 -0.419629812
PAPPA2 0.895836285 0.361509007 0.309759235 0.185110889

PAQR3	0.625459507	2.052086578	0.473767041	-0.105551779
PAQR7	0.215823574	-1.393438647	0.82280647	0.323222094
PAQR9	0.586162908	0.011917237	0.906976076	-0.473311036
PARP10	0.648894071	-0.435872682	0.732967162	0.168102663
PATE1	0.170747638	0.370584338	0.152047738	0.440583621
PAX6	0.168511508	1.025280204	0.023303172	-0.711083231
PCDH17	0.994699014	1.530219411	0.293304207	0.108549735
PCDHGC4	0.064240436	0.82899005	0.868081213	0.22469804
PCGF1	0.851910051	0.67049839	0.269584707	-1.191893185
PCGF2	0.062790977	2.253590264	0.019455778	-1.353040049
PCGF3	0.168320102	1.17351526	0.016964007	0.857634395
PCGF6	0.251364639	-0.456934187	0.31802169	0.574863308
PCLO	0.041618292	-1.577171974	0.321443029	0.276262853
PCOLCE	0.957923147	0.110303057	0.164608751	0.502865952
PCSK1N	0.816122712	0.692801862	0.012778611	-0.743346099
PCSK4	0.119005904	-1.41478762	0.617179307	-0.341385428
PCSK5	0.727091179	0.66252493	0.787119521	-0.404470751
PCSK9	0.51928146	-1.126162589	0.869311585	0.068106241
PCYOX1	0.346863264	0.864483283	0.102259651	-0.56552458
PDCD11	5.64E-05	-10.06836572	0.000834548	-6.725213053
PDCD6	0.042248513	1.414439159	0.794947264	0.108834754
PDCD6IP	0.16760581	-1.250049077	0.112234282	-0.884000263
PDCL	0.680112595	0.006124901	0.52640026	-1.058798926
PDIA2	0.544412781	-3.333009098	0.063568057	-1.498791916
PDIA3	0.075691271	-2.290521162	0.853955626	-0.199240367
PDIA4	0.088755755	-1.935075558	0.84538001	0.1230005
PDIA5	0.614429952	0.702995243	0.319919478	0.311316606
PDIA6	0.55222513	-0.93429707	0.012638783	-0.726580481
PDRG1	0.113022344	-0.820217035	0.736500304	-0.902043159
PDZD11	0.525890128	-0.130375795	0.026992912	0.583184148
PDZD2	0.453270741	-0.508267974	0.196210296	-0.922363406
PDZD8	0.897073066	-0.476575989	0.367264357	-0.331086841
PDZRN3	0.726504455	-0.142244213	0.190854064	-0.046485214

PDZRN4 0.311998443 0.272552108
0.934279416 -0.599706062
PEBP1 0.730614868 -0.655830336 0.245279316 0.293815434
PEG10 0.306420379 -0.47168806
0.885334157 -0.095519477
PELI1 0.677822753 -1.839623569
0.055843335 -1.852327673
PELI2 0.384112279 0.263587108
0.440661074 -0.498725983
PELI3 0.362301158 -0.271900264
0.573323243 -0.238313832
PEPD 0.985345049 -0.758831382
0.064127966 -0.669317652
PER2 0.059337977 -1.151655896
0.524870616 -0.632559826
PEX10 0.106428673 1.339968821 0.095704852 0.151685917
PEX11G 0.627675622 0.229409973
0.844768153 -0.381388806
PEX12 0.92558205 0.095319582
0.923719449 -0.063987195
PEX19 0.469533791 -1.410830628
0.630571765 -0.108598907
PEX2 0.738858838 0.060954873
0.206689018 -0.501157678
PEX6 0.017774088 1.802100996
0.017624326 -1.154696415
PFDN2 0.875468135 -3.574201961
0.021673808 -2.456255887
PFDN4 0.577855353 -0.297202845
0.670971134 -0.517251072
PFDN5 0.10677273 -1.298968536
0.03043861 -0.896135646
PFDN6 0.106126906 1.056706189
0.324864791 -1.614853971
PGA4 0.697957588 -2.71471587
0.485913833 -0.236690775
PGA5 0.1840529 -2.71471587
0.040611163 -0.257617713
PGAM5 0.768541314 -1.212454088
0.000464339 -2.682520551
PGAP3 0.928687286 0.105679197
0.986592156 -0.545516526
PGC 0.751874178 -0.86847336
0.935522562 -0.182744552
PGM3 0.394878483 1.747212223 0.605122774 0.264986356
PGM5 0.718406973 -0.17128095
0.013676496 -2.895624765
PGPEP1 0.396182579 -0.092915445
0.991580881 -0.473209509
PHB2 0.000458922 -6.065487367

0.000115547	-7.280155849
PHEX	0.892745444	-0.394596324
0.187692421	-0.444133723
PHF14	0.26720468	-2.72464904
0.774563797	-0.057970102
PHF17	0.578351304	-1.515424671	0.014866094	-0.72613966
PHF21A	0.090230772	1.485494694
0.080715285	-0.163585611
PHF7	0.848440683	0.798408095	0.78051277	0.335731256
PHLDA1	0.784681667	-0.279370612	0.370962469	-0.38795057
PHRF1	0.546490692	-2.189111009
0.7224019	-0.204589722
PHTF1	0.650879843	0.299312871
0.105238659	-0.674227197
PIAS1	0.898310098	-0.244662209
0.842933148	-0.091175682
PIAS2	0.811876331	-3.241433386	0.241803846	0.216914748
PIAS3	0.388827101	-0.510471304	0.664714407	0.556684604
PIAS4	0.908215002	0.295271239	0.837433285	0.140265225
PIGK	0.890849205	-0.46238097	0.81588779	0.113690674
PIGU	0.728265105	-0.904676273	0.682979047	0.329371075
PIGW	0.414711649	0.511070717
0.711306861	-1.778665242
PIK3IP1	0.834991433	-0.748595019	0.994699014	0.039441064
PIN4	0.367264359	0.880446339	0.471839165	-0.78762085
PITPNB	0.31349716	0.629630631
0.161660887	-0.473954435
PITPNC1	0.665282245	-0.835796735	0.519788291	0.243807042
PITRM1	0.116984394	1.781252828
0.714804088	-0.859819272
PJA1	0.291381301	-2.971506959
0.031196176	-1.049566333
PJA2	0.124764814	0.93589389
0.011014152	-0.951575349
PKD1L2	0.6517078	-0.212954932	0.495256895	0.287627083
PLA2G15	0.48054859	0.518170143	0.63239601	0.331608751
PLA2G2C	0.241175686	-0.701473268	0.075305754	0.630705488
PLA2G3	0.57598715	-0.88319363
0.929308464	-0.805030837
PLA2G4D	0.014315977	-1.201263802	0.704330668	0.278153159
PLA2G4E	0.384539448	-0.552914064	0.535111046	0.752648504
PLA2G4F	0.823414731	-1.808056035	0.472320729	0.233226706
PLA2R1	0.895217988	-0.560891281
0.048648424	-0.691418552
PLAA	0.858863806	0.144754484
0.081396051	-1.022684486
PLB1	0.216403988	-0.839232878	0.081944013	0.193081512
PLBD2	0.798567097	-0.167890489
0.618280557	-0.390444011
PLCD3	0.72181647	-0.271354622

			0.16953061	-0.956139419
PLCD4	0.106090269	0.37213677	0.843850545	-0.114943694
PLCH1	0.057330342	-1.220674218	0.07380295	0.685897923
PLCL2	0.260050024	-0.089783647	0.651707798	0.165970822
PLCXD2	0.64043096	-0.502832505	0.367054855	0.23366691
PLCXD3	0.243379287	0.802491768	0.260854464	0.582131552
PLD3	0.040103947	-2.296682672	0.650027812	-0.469629822
PLD4	0.787119522	-0.329699534	0.219910068	-0.887242893
PLEKHF1	0.354935556	0.444465079	0.939252931	-0.350432557
PLEKHF2	0.149896354	1.066224307	0.114955713	-0.987394129
PLIN2	0.451859689	-0.519355697	0.668124306	-0.344970113
PLXNA2	0.468016312	0.324573189	0.853202766	-0.201515041
PLXNB2	0.131115623	-2.720621919	0.5947961	-0.96565252
PM20D1	0.858863806	-0.98194747	0.429623275	-0.139940395
PM20D2	0.864392025	-0.404860745	0.131115621	-0.776306
PMPCA	0.929929687	-0.406216426	0.748317356	0.266241381
PMPCB	0.462741066	-1.346631844	0.455628012	0.116280412
PNKD	0.504195965	-1.382101682	0.119933775	-0.179941385
PNLIPRP3	0.972023225	-0.115392377	0.101023019	0.770018451
PNPLA1	0.189005044	1.915724705	0.082910144	-0.848290482
PNPLA5	0.708397137	-1.066158425	0.758831587	-0.540819442
PNPLA6	0.098221639	0.620859179	0.327198474	0.21918621
PNPLA7	0.066111898	1.669901552	0.513892424	-0.205056032
POC1A	0.943606709	0.374083128	0.189795856	-0.763482134
POC1B	0.543375299	-0.361524532	0.143303003	-0.11323021
PODN	0.115339764	0.924107591	0.082095383	0.76119399
POFUT2	0.005334347	1.487465671	0.988462877	0.242432947
POP1	0.000287434	-8.685603623	1.44E-05	-6.125978507
PPIAL4A	0.660744948	-0.267811198	0.809452301	-0.28618895
PPIAL4B	0.761980263	-0.248263726	0.748317356	-0.189836629
PPIAL4C	0.653398424	-0.371097107	0.888421012	0.097941525
PPIAL4D	0.07309635	0.450508159	0.755696857	-0.145154089
PPIAL4E	0.864392025	-2.354225996	0.114989944	0.128598167
PPIAL4F	0.433284578	-2.419107873	0.905118112	-0.504773409

PPIAL4G	0.264183553	0.790717714	0.822198316	0.351931454
PPID	0.564838239	-0.564077801	0.879782231	-0.163974048
PPIE	0.006336497	-9.539111376	2.36E-05	-7.711331568
PPIL1	0.164727504	-1.099103089	0.679539855	-2.15583898
PPIL2	0.572791165	-2.886552152	0.348870297	-2.038798497
PPIL3	0.689301312	0.788314054	0.053771786	-0.814232072
PPIL4	0.073276658	1.56596782	0.01079443	-1.274462266
PPIL6	0.51624583	-1.078117287	0.319539329	-0.152846954
PPP6R1	0.315377208	0.414089168	0.147625888	0.490538309
PPT2	0.175637941	0.635209659	0.095745959	-0.697782754
PPWD1	0.860705791	0.167276488	0.007112281	-1.400105129
PQLC2	0.20668902	0.336877025	0.002704207	-1.039753297
PRAF2	0.169530612	1.292974437	0.158868604	-0.77267182
PRCP	0.237741334	-1.357912211	0.357380183	-0.912068906
PRDX1	0.203892868	-0.475938883	8.45E-05	-2.204481642
PRDX4	0.461789096	1.272330202	0.827675504	-0.138904212
PREB	0.232196258	-1.880115962	0.137621992	-2.978617489
PREPL	0.180159857	1.252240855	0.035039792	0.577123834
PRG3	0.262848419	1.010633435	0.011338542	-0.891483274
PRICKLE3	0.007735493	-1.627355734	0.593172958	0.159569155
PROCA1	0.840487802	1.435988441	0.598591298	-0.513365444
PRPF3	7.71E-05	-8.37241036	0.000105295	-4.951104925
PRPF31	0.138660722	0.615235271	0.814908902	-0.145662381
PRPF4	0.149728416	-1.068365453	0.00472955	-2.575431437
PRPF8	0.000110486	-9.18601846	1.37E-05	-7.095636247
PRR11	0.350481227	-0.662962086	0.836212161	0.261282179
PRR18	0.177181847	1.279566479	0.039926087	-0.172022638
PRRC1	0.817944251	-0.003806106	0.267879253	0.565792298
PRSS12	0.357788651	0.264995943	0.233420491	-1.281648711
PRSS16	0.026139944	1.363257716	0.480062445	-0.57708691
PRSS21	0.379008927	0.226712831	0.295103021	-0.597972693
PRSS22	0.343667329	-0.592556718		

0.145560247	-0.687655387			
PRSS23	0.302372058	-0.347902614		
0.638806307	-0.554411576			
PRSS27	0.611137865	-1.251667594	0.613331721	0.557498112
PRSS33	0.299450273	1.10784075	0.396182577	0.538332172
PRSS35	0.500212569	-0.404410121	0.616353934	0.203160799
PRSS36	0.005373063	-1.358594112	0.210650248	0.528301276
PRSS37	0.333855273	-0.309468955		
0.379394707	-0.915062301			
PRSS38	0.000788766	-2.099320006		
0.667555511	-0.138410236			
PRSS42	0.801586937	1.609296553	0.137208184	-0.72371003
PRSS45	0.834381217	-1.574008633	0.767944161	0.157159662
PRSS48	0.512212684	-1.334415001		
0.391845703	-0.053494239			
PRSS50	0.064015661	-0.357194284		
0.216694613	-0.336429525			
PRSS53	0.256898007	-2.198911777	0.0419324	0.094162413
PRSS54	0.647769978	0.413125449	0.269231911	0.140647602
PRSS55	0.486403187	0.513431532		
0.703170365	-0.209997053			
PRSS57	0.031875898	2.268598126		
0.013947403	-1.110639788			
PRSS58	0.06180343	0.599018322	0.22554441	-0.23747144
PRTG	0.923719449	-0.415512759		
0.544931888	-0.255789596			
PRTN3	0.600221168	1.416544611	0.699693611	0.114931296
PSEN2	0.498723111	2.128849904	0.612782931	0.281110142
PSMA8	0.643281654	-4.476021816	0.280221912	0.312736066
PSMB10	0.145236131	0.227685779	0.666986907	0.057834391
PSMB11	0.404942821	-1.598601501	0.035060099	0.618523157
PSMB8	0.06733918	-1.735161327		
0.528443322	-0.354884753			
PSMB9	0.474732603	-0.775995984	0.50569404	0.210178645
PSMC3IP	0.696801115	-0.425996313		
0.17568123	-0.436970694			
PSMC4	0.178944607	-9.852791962		
0.022630114	-6.199735565			
PSMC6	0.587238873	-0.383229778		
0.202228444	-2.129690865			
PSMD10	0.624352716	0.22947941	0.350078052	0.12058565
PSMD5	0.364777485	-1.181061603		
0.643841986	-0.683138974			
PSMD6	0.086006785	0.291967064		
0.41515897	-0.864307385			
PSMD9	0.709560519	-0.282327693	0.230368459	0.079534045
PSME1	0.201399958	-0.568187313	0.440661074	0.515442644
PSME2	0.316131297	1.215374404	0.831941354	0.159564719
PSME3	0.156341221	-1.669502499		
0.131915628	-1.852610928			

PSME4	0.560618507	-0.624035993	0.547531118	-0.271482171
PSMF1	0.003485295	0.676453038	0.664714407	0.099348024
PTGES3	0.111081369	-0.550449809	0.564309937	-0.430048087
PTRF	0.889656231	-0.048481753	0.069113195	0.35465712
PWP2	0.000194823	-9.351656899	6.62E-07	-9.371322555
PXMP4	0.116984394	-1.690500568	0.497731448	-0.326388021
PYDC1	0.585087859	-0.099626061	0.377739636	-0.579552456
PYGO1	0.663579311	0.74869431	0.702590473	0.185532295
PYGO2	0.884099899	-0.783568825	0.140125073	-1.025996534
QPCTL	0.726504455	-0.643397841	0.616078918	-0.358735301
QSOX1	0.344264622	-0.363310234	0.487954095	-0.56505579
R3HDM1	0.094465964	1.597163955	0.417805999	0.428861818
RAB10	0.739448856	-0.404466347	0.665282244	-0.355644309
RAB11FIP2	0.74121983	-0.488008394	0.067690953	-0.372428629
RAB11FIP5	0.493281996	1.110348093	0.262515363	-0.995277009
RAB12	0.392710767	1.458574618	0.555889631	-0.217203946
RAB14	0.244328006	0.528896416	0.578122471	-0.11631775
RAB18	0.215823574	-3.746579897	0.075177606	-0.309902683
RAB1A	0.318400659	0.665739758	0.059127501	-0.264724085
RAB23	0.475699242	0.112219007	0.681831914	-0.809948312
RAB24	0.152719625	-0.822138264	0.488363252	-0.461623246
RAB30	0.091876007	0.437466588	0.811410597	0.210436592
RAB32	0.16212985	1.238533118	0.10744902	-0.421321786
RAB35	0.08003915	-0.696188019	0.000736907	-2.031287887
RAB37	0.124275154	1.704476218	0.17481026	0.638620528
RAB3C	0.860705791	0.429613333	0.051858917	0.880764978
RAB3GAP2	0.03064933	-0.960282852	0.145236129	-0.451897983
RAB3IP	0.089049194	0.64132297	0.724745248	-0.205263547
RAB40A	0.088025567	1.805491735	0.06554557	-0.874139516
RAB40AL	0.02505585	-0.373683246	0.160492966	-0.442046416

RAB40B	0.080715286	0.357508132	0.181996773	-0.671200462
RAB40C	0.39047414	-0.545995036	0.004545261	0.909688563
RAB42	0.187954408	-0.112465936	0.048648424	-0.807580412
RAB43	0.363538002	1.62581908	0.0216744	-1.087198734
RABAC1	0.688725656	-0.533962965	0.227947342	-0.285638291
RABGEF1	0.473284669	-1.85640069	0.092480216	-0.232844242
RABIF	0.369344804	1.489471395	0.137311546	-0.222018527
RABL3	0.986592156	0.266277061	0.07950156	0.477199536
RAG1	0.177432875	1.469453198	0.999064525	0.497798441
RAI1	0.356972012	-0.338426719	0.291870464	-0.359960358
RALB	0.472504566	0.416028117	0.72091415	-0.508426256
RALYL	0.167318647	0.261174872	0.042475893	0.723519275
RANBP10	0.158406796	-1.506024803	0.113734742	0.289797424
RANBP2	0.127366866	-1.404486668	0.349272588	1.039320378
RAPGEF4	0.501704383	0.391247055	0.735911058	-0.19955181
RAPSN	0.705491661	-0.165838906	0.440197944	0.200813754
RASA4	0.428566748	-1.246168867	0.957923147	0.597500491
RBBP6	0.68527551	-0.352597744	0.065032285	-0.190971181
RBCK1	0.886568693	-0.639597405	0.276410225	-0.734503794
RBM24	0.976616473	-0.610328576	0.927445058	-0.233525279
RBM34	0.641601871	0.19363831	0.221974011	-1.728807893
RBM7	0.119933777	-0.609784589	0.186777596	0.446337831
RBP7	0.299086379	-0.442591215	0.779912934	0.167343808
RC3H1	0.864392025	-0.160360781	0.291512766	-0.094870345
RC3H2	0.023291581	1.449368794	0.562199115	-0.582516281
RCE1	0.650018964	-1.313014016	0.138244507	-0.660918927
RCL1	0.348468303	-5.67329868	0.001838303	-3.999483732
RDH10	0.968513851	-0.784989054	0.458939754	-0.577315105
REST	0.03756913	2.013813249	0.311624501	-0.319116103
RFFL	0.689877149	1.092439866	0.208945904	-0.623357964
RFPL1	0.985345049	-0.388414266	0.189531981	0.052914423

RFPL2	0.276755276	-2.333939035	0.670926735	0.163808195
RFPL3	0.714220799	0.247779957	0.599134364	-0.395464801
RFPL4A	0.542856928	0.175856211	0.360245614	0.219884733
RFPL4B	0.414711649	-0.697187356	0.353920081	-0.589548861
RFWD2	0.915653142	0.508741894	0.814908902	0.022136718
RFWD3	0.773323453	0.173590079	0.209229265	-0.500238915
RGL4	0.700004248	0.393363435	0.375385892	-0.183112233
RGN	0.157486226	1.252714192	0.294022848	-0.551342615
RGS19	0.341282759	1.36703305	0.004853472	-0.38858704
RHAG	0.963529184	1.22002553	0.716554954	0.131568088
RHOBTB1	0.460363192	1.29041329	0.728265104	-0.38920236
RHOBTB2	0.450451123	1.787793118	0.900784901	0.230916073
RHOBTB3	0.497236013	0.387510533	0.357788649	0.244590286
RHOQ	0.216089186	0.617421217	0.180184715	-2.109911776
RHPN1	0.462264944	0.354185529	0.669262467	-0.107102243
RIC8A	0.793138998	-0.403469331	0.043289836	-0.628753045
RILP	0.638806309	0.26178781	0.679359268	-0.396628001
RIMBP2	0.72415917	0.808200652	0.850278187	-0.807175118
RIMS1	0.519788293	-0.263722986	0.343269162	0.192013036
RING1	0.545451245	0.093404376	0.606214451	-0.090957985
RLIM	0.592998825	-0.5969835	0.661340105	-0.336977281
RNASEK	0.862586538	-0.599134589	0.398908951	-0.89117281
RNF10	0.514731473	-2.775830236	0.433743505	-0.267208886
RNF103	0.962283233	0.170545238	0.01456644	-0.351884709
RNF11	0.36892813	-1.16022142	0.507695114	-0.383789671
RNF111	0.577588293	-0.09206706	0.063791534	0.261950703
RNF112	0.804004948	-0.436745787	0.361066951	-0.194381101
RNF113A	0.982850946	-1.760479646	0.017924962	-0.737037054
RNF113B	0.829503111	0.287690246	0.895217988	0.11999713
RNF114	0.523249163	1.506871528	0.056227861	1.19647394
RNF115	0.473767043	0.687600246	0.852116504	-0.27569065
RNF121	0.708978743	-2.045039826	0.611686002	-0.059576627

RNF122	0.794947265	0.656149657	0.463694128	0.004943478
RNF123	0.75484223	0.188644542	0.379432604	-0.788071437
RNF125	0.95294174	-0.408507338	0.457045681	0.640304306
RNF126	0.05624536	-1.075376397	0.902641637	-0.206251051
RNF128	0.547010783	-0.496876011	0.402302663	-0.289978293
RNF13	0.600221168	-0.861521345	0.861319954	-0.065842261
RNF130	0.026510208	1.920886538	0.624906004	-0.413260935
RNF133	0.883482876	0.09214759	0.836822674	0.786864326
RNF135	0.228248989	0.008443268	0.711889313	0.398231326
RNF138	0.787119522	0.402344576	0.206127572	-0.72660119
RNF139	0.641601871	-0.889139533	0.74890979	-0.559702435
RNF14	0.265355624	0.551772862	0.248469111	-0.78042086
RNF141	0.33654548	0.440416992	0.844156391	-0.241218813
RNF144A	0.304207772	-0.643178826	0.337332073	-0.45729755
RNF144B	0.271269678	-0.578200966	0.192716366	0.51874378
RNF145	0.397053419	-3.064837593	0.936144194	-0.573435756
RNF146	0.13638345	-2.583319862	0.136795337	-1.486876044
RNF148	0.127366866	1.739591878	0.127171896	-1.100918837
RNF149	0.698536088	0.4792027	0.00282454	-1.026599808
RNF150	0.586162908	-0.60395134	0.609494757	-0.438866048
RNF151	0.174765438	1.820110072	0.342166348	1.249380785
RNF152	0.184828365	2.138398352	0.251041754	-0.147537839
RNF157	0.535111047	-0.440000264	0.887186064	0.103313821
RNF165	0.411143333	-3.083307384	0.032568275	-1.436511936
RNF166	0.569073189	-0.281076716	0.949206881	-0.89367411
RNF167	0.29617016	0.486445265	0.166388313	-0.330153926
RNF168	0.41829821	0.837761325	0.436502969	0.112438954
RNF169	0.348870299	0.549372445	0.133729468	-0.211339518
RNF17	0.929308465	-0.47780952	0.343269162	-0.271838046
RNF170	0.308643624	-1.038425443	0.829503111	0.162594208
RNF175	0.342871293	-0.84451048	0.186516824	-0.21524854
RNF180	0.200299162	1.638386333	0.906666379	-0.226192944

RNF181	0.378162443	-0.437319637
0.737089705	-0.232978631
RNF182	0.178691989	-1.821066108
0.010837826	-0.806929369
RNF183	0.211636146	1.74125187
0.159785374	-0.583845696
RNF185	0.10357936	1.405727001	0.786518266	0.434970494
RNF186	0.245596999	0.982199955	0.729439668	0.288178551
RNF187	0.922477947	-2.020428745	0.099660395	0.665174803
RNF19B	0.781712836	0.92641614
0.208380016	-0.145601381
RNF207	0.977239863	-0.057660227
0.236810715	-0.305763799
RNF208	0.084294818	1.557469436
0.203903604	-0.895551387
RNF212	0.452800114	0.196723853
0.021763344	-0.862391424
RNF214	0.511206972	0.554159884
0.059492059	-1.505847871
RNF215	0.833771101	0.518945878	0.367679861	0.285096796
RNF216	0.164015965	-4.851126654
0.222862792	-0.335291859
RNF217	0.288306743	-1.070037103
0.085721464	-0.234588946
RNF219	0.157946003	0.831794751
0.64160187	-0.020033747
RNF220	0.578656884	-1.271495589	0.13928685	0.642460327
RNF222	0.058395714	0.6008811	0.257554506	0.313629829
RNF24	0.271951275	-0.447031761
0.034455674	-0.984363873
RNF25	0.288616788	0.543277204
0.881736247	-0.186580318
RNF26	0.115712082	1.827053384
0.207251574	-0.295065236
RNF31	0.365191231	-0.44604139
0.206408157	-0.318523408
RNF32	0.06180343	-0.357168693
0.193518571	-0.622839146
RNF34	0.358197412	-1.417907131	0.127562066	0.148926142
RNF38	0.399337645	0.248636133
0.021913766	-0.433005092
RNF39	0.605122775	1.082329202
0.67610732	-0.653708508
RNF4	0.132719413	-0.787144194
0.396182577	-0.166574565
RNF40	0.518967	0.804941174	0.569839616	0.280317207
RNF41	0.002853555	-4.41208901	7.75E-06	-1.296118464
RNF43	0.301313605	1.067816203
0.028041032	-0.594147484
RNF44	0.388397031	-0.658333065	0.196751916	0.240981808

RNF5	0.198383349	-1.01970772	0.194323225	0.178577726
RNF6	0.401863645	-2.031610504	0.256570191	-0.651153939
RNF7	0.626567143	0.610608388	0.027925413	0.955705775
RNFT1	0.626567143	1.090634655	0.35331168	-0.107445096
RNPEP	0.759598634	0.246889334	0.093698207	-0.865767881
RNPEPL1	0.287243371	0.832060031	0.718307312	-0.352248014
RP2	0.119376375	-4.079842822	0.95294174	-0.323809583
RPH3A	0.263555506	-0.541186087	0.076467133	-1.392129509
RPL36	0.03756913	-8.681225599	1.74E-06	-6.390967019
RPL36AL	0.674393569	0.435656632	0.369344802	-0.301817127
RPL7	0.534082489	0.736147009	0.070195911	-0.837150267
RPLP1	0.962906197	-0.323488115	0.003207347	-1.546678711
RPP38	0.819159157	0.538112023	0.503198548	-2.07948462
RPRD2	0.287243371	-1.176449742	0.274346091	-0.28176554
RPS25	0.756624803	-0.921194936	0.047674546	0.744061414
RPS4Y2	0.21365701	-3.831212776	0.493281994	-0.304360156
RPSA	0.100791012	-2.449165683	0.017145216	-3.13468892
RRAGA	0.785316139	0.079734815	0.512212682	-0.033495138
RRAGD	0.732967163	-1.394269183	0.323737226	0.038357059
RREB1	0.272975867	-1.55597029	0.354935554	-0.15642498
RRP8	0.256242669	-2.484126552	0.466559869	-0.322112508
RSC1A1	0.209796825	-1.383338326	0.952319189	-0.246167938
RSPH9	0.370596581	-0.346358989	0.211220591	0.351778494
RSPO1	0.386379335	-0.041709539	0.09408161	-0.071593565
RSPRY1	0.123153555	1.728046438	0.63997411	0.021673915
RTL1	0.782313064	0.453918093	0.557988948	0.331588554
RTN3	0.359425454	-1.156765951	0.906356698	0.177291896
RTN4RL1	0.429166881	0.409083149	0.754248327	-0.141194723
RTN4RL2	0.618280558	0.446481346	0.442516384	-0.105553646
RUVBL2	0.590472242	0.507430452	0.803400268	0.056670745
RWDD2A	0.424618451	-0.596637087	0.377317122	-0.483505451
S1PR3	0.184828364	-2.070694385	0.10744902	0.434068833
SAMD8	0.730614868	0.513475578		

0.096956715 -0.562681109
SAMM50 0.214361565 -2.64000677
0.002345875 -4.530122229
SAP130 0.888421012 -1.016784461
0.581868218 -0.309159864
SAR1A 0.006138743 1.611906061 0.610869981 0.112405476
SARS2 0.720061155 -2.214050402
0.130717031 -0.809730512
SBNO1 0.42734412 -0.766770604 0.492296128 0.244972209
SCAF11 0.099821291 1.323876292 0.454684276 0.193438717
SCAMP2 0.769736031 -0.601272285
0.799774673 -0.104560788
SCAMP3 0.291155364 0.269849263
0.997193581 -0.348029292
SCAMP4 0.405825169 -0.395083926
0.171971195 -1.242857268
SCAMP5 0.833771101 -0.398875038
0.29690921 -0.134253716
SCARB2 0.598591299 -0.024322433 0.607853618 0.091229816
SCFD1 0.253958133 -0.814390318
0.008702149 -6.856555487
SCFD2 0.166876068 -1.359410435 0.241803846 0.16484724
SCG3 0.551180311 -0.280809394 0.488988753 0.140305915
SCG5 0.764364149 0.116451608
0.91069352 -0.051308361
SCNM1 0.834991433 -1.315940544 0.720646095 0.210157917
SCRIB 0.371223456 -2.101530116 0.046760274 0.325322226
SCRN1 0.076824184 -1.051894851 0.14549719 0.550409641
SCRN2 0.586162908 -0.346781561
0.567483305 -0.165389029
SCRN3 0.791331843 -0.646187573
0.412925205 -0.387355528
SEC11A 0.694778983 -2.071161615
0.577054344 -0.619275347
SEC11C 0.011038809 -3.112830505
0.583477002 -0.273344486
SEC13 0.00357375 -7.408364075
0.010989543 -7.663872698
SEC16A 0.934900969 -0.236642392
0.650018963 -0.104080509
SEC22C 0.816122712 0.779148446
0.0088025 -1.415479809
SEC23A 0.102752966 0.472365438
0.215244281 -0.872861153
SEC23B 0.911933108 1.064491355 0.166636302 0.692873248
SEC23IP 0.326042066 -0.661987908 0.279526235 0.217880602
SEC24A 0.668124307 0.263883065
0.743583259 -0.293584333
SEC24B 0.316508785 -0.449875988
0.297996465 -0.410415591

SEC24C	0.562726464	0.502099287	0.793138997	0.11562681
SEC24D	0.895836285	−0.687135044	0.982227445	−0.170876795
SEC31A	0.559565964	0.880368035	0.37436769	0.147460455
SEC31B	0.310504457	−1.93946069	0.941118621	−0.349446705
SEC61A1	0.900784901	−3.318144032	0.00126834	−4.921941982
SEC61B	0.19566977	−1.080736878	0.392710765	0.241684808
SEC61G	0.240235599	1.241676619	0.587238872	−0.463318171
SEC63	0.796756634	0.487843058	0.03608759	1.087585894
SELM	0.453741645	0.627132193	0.257554506	−0.316433323
SELV	0.356156548	0.972946767	0.154296139	−0.467242463
SENP2	0.021540106	−2.9164858	0.585625267	−0.188071663
SENP3	0.253751253	0.901874776	0.395692569	−0.222628965
SENP7	0.620065241	−3.953551564	0.462977718	0.275813512
SENP8	0.347665195	0.950352643	0.538463201	0.19176506
15−Sep	0.857022589	0.011868192	0.168803523	−0.263683226
SERBP1	0.06609547	1.190100701	0.199750412	−0.615889267
SERGEF	0.817336961	−0.937015016	0.506193919	0.253749376
SERHL2	0.900166109	−0.366106441	0.368095657	0.059095101
SERINC1	0.171725962	0.946114418	0.895217988	0.258442528
SERINC2	0.993451749	−0.144957362	0.187692421	0.169830871
SERP1	0.323737228	1.239644854	0.57652063	0.006540937
SERP2	0.742401241	0.449006512	0.944228817	−0.133186281
SERPINA3	0.726504455	−0.731189424	0.580261509	−0.72011683
SERPINB10	0.336152625	−1.613033604	0.005207108	−1.418676366
SERPINB13	0.733555629	0.426406691	0.735321966	−0.555780527
SERPINB4	0.742575412	0.364109908	0.866799441	−0.542247104
SERPINB7	0.373036347	−0.476788691	0.060721267	0.581657
SERPINE2	0.787720904	0.201748983	0.099339227	0.901805136
SERPINH1	0.211111736	−1.629664085	0.218841311	0.288115429
SF3A1	0.523088914	−3.639491817	0.497731448	−2.671274625
SF3A3	0.863162948	−0.719437669	0.815515751	0.014241719
SF3B4	0.011421865	1.740364287	0.5082489	0.012587081
SFT2D3	0.280918766	−0.244578098	0.870542274	−0.106373099
SFTPB	0.925892523	−0.711486349	0.131715272	0.387498362

SFXN5	0.838654801	−0.02543726	0.062570401	−0.05897075
SGMS1	0.794344387	1.452777998	0.126006956	−0.317289604
SGSH	0.135562546	0.646719483	0.750095102	0.514400631
SGTA	0.982850946	0.021176714	0.133526983	0.395823925
SGTB	0.379008927	−0.760750037	0.270929317	−0.140945624
SH3GLB1	0.208662823	0.111214806	0.015708206	−0.931237323
SH3PXD2B	0.435327429	−1.575600987	0.59813971	−0.572797462
SH3RF1	0.009621394	−1.458811309	0.05195319	−0.799849562
SH3RF2	0.039400053	0.567863414	0.311624501	0.817750375
SH3RF3	0.542338803	0.37015605	0.543893915	0.06835901
SHANK2	0.012778611	−4.170409972	0.017036288	0.703115408
SHFM1	0.31707557	−3.363674173	0.308643622	−2.764676372
SHKBP1	0.833771101	0.329747785	0.395747589	−0.311524013
SIAE	0.87423621	−0.137791011	0.201124344	−0.842336525
SIAH3	0.390981799	−1.682781642	0.892436447	0.229698042
SIL1	0.059654855	1.283579488	0.112136793	0.907934219
SIRT4	0.033729042	−4.071864054	0.001496459	−0.869362913
SKIL	0.075691271	1.431049881	0.029286837	−1.136156098
SLC10A2	0.41337139	−2.494204147	0.56854299	0.239079093
SLC10A5	0.558514381	−0.564685075	0.461789094	−0.271708803
SLC11A1	0.281616794	1.144267416	0.258870997	−0.340798768
SLC11A2	0.013192009	−3.155778613	0.127990037	0.632530769
SLC13A5	0.614261439	−0.724634807	0.338826167	−0.115862727
SLC15A2	0.712421462	−1.868555375	0.689735678	−0.566188546
SLC15A3	0.611686003	−1.983126173	0.616629005	0.53927869
SLC17A3	0.416054474	−0.690830957	0.026510208	0.930952525
SLC17A5	0.755436276	0.536014454	0.354935554	−1.085783994
SLC17A8	0.982850946	0.04564832	0.903879758	−0.099730819
SLC20A1	0.772725211	−0.662031645	0.068755353	−0.205303407
SLC25A17	0.647208233	−0.480099712	0.863777445	−0.387544576
SLC25A21	0.261850131	0.120300886	0.350481225	−0.056006156

SLC25A25	0.196480971	0.494370526	0.596421267	-0.26567887
SLC25A28	0.866851161	-0.072623175	0.278138405	-0.79517188
SLC25A33	0.158637574	0.576425008	0.051296282	-1.134720778
SLC25A40	0.518774883	-0.515789612	0.879782231	-0.225128387
SLC2A13	0.568013031	0.544145253	0.022353212	-0.465101448
SLC2A6	0.434662206	-0.555645416	0.481035	-0.226567383
SLC2A8	0.583477004	-0.245545613	0.005181991	-1.171992488
SLC2A9	0.127562067	-1.639840441	0.084180556	-1.245678753
SLC30A1	0.865006687	0.28516353	0.010343326	-0.836660377
SLC30A2	0.448109049	0.2034364	0.724159168	-0.402413118
SLC30A4	0.930550952	-0.914039093	0.088025566	-0.618572416
SLC30A5	0.210365496	-2.427748202	0.033794553	-0.497567926
SLC30A7	0.612234359	1.408611301	0.012891457	-0.846285496
SLC30A8	0.031300655	-1.851816472	0.895411173	0.116564383
SLC33A1	0.306050873	-1.671897484	0.681831914	-0.110594957
SLC35A1	0.254609406	0.638399553	0.135972518	-0.518261963
SLC35A3	0.343667329	-1.369613013	0.467996418	-0.896719421
SLC35A4	0.967890697	-0.91077893	0.819159156	-0.332596151
SLC35D1	0.738858838	-0.362973299	0.676678944	0.118713995
SLC35F6	0.136589274	1.512814088	0.897073066	1.022928388
SLC36A4	0.772725211	-0.221943441	0.398798557	0.696137581
SLC37A4	0.290798255	-1.466660994	0.164965198	0.070694588
SLC38A11	0.642262628	-0.657546404	0.018422445	0.679565898
SLC38A7	0.481521682	-0.393509033	0.690453164	0.020457193
SLC39A1	0.078435192	-2.138756077	0.556486259	-0.131663374
SLC39A13	0.16072604	-0.984158772	0.658480952	-0.170156707
SLC39A6	0.142450199	-1.217177383	0.969137025	-0.344330157
SLC39A9	0.251364639	1.266141428	0.009909078	1.045573883
SLC40A1	0.946095347	0.562376649	0.144481995	1.05043204
SLC47A1	0.549614908	0.834375304	0.926203007	-0.132192941
SLC4A2	0.376472971	-0.211025854	0.013410247	-1.305835902

SLC4A5	0.580261511	0.631481855	0.099339227	0.708479495
SLC51A	0.687574883	1.222514965	0.921857266	0.082280194
SLC9A6	0.134949377	0.571808824	0.339698943	0.256964428
SLCO1B3	0.020806561	1.169979085	0.134565104	-0.565445643
SLMAP	0.213513133	-1.110790947	0.203336954	0.067693345
SMARCAD1	0.096799521	-1.311007508	0.113452118	-0.685193307
SMURF2	0.93332949	-5.34023711	0.229804253	-2.960955973
SNAPIN	0.722401901	-0.521185767	0.155657254	-0.742255795
SND1	0.407593308	-0.770642097	0.594254827	-1.405916848
SNF8	0.299450273	1.003626873	0.666418495	-0.218389302
SNN	0.824023098	0.515578859	0.644402522	-0.299968095
SNRNP200	0.025515601	-9.91025412	0.002080824	-5.853681827
SNRNP25	0.230064826	-1.843924497	0.031565375	-2.502544878
SNX1	0.802795707	0.739384911	0.913792888	0.307530413
SNX10	0.884099899	-0.853176625	0.018520249	-1.112856309
SNX12	0.113200113	-0.349187905	0.61497939	-0.137900951
SNX13	0.01559059	-2.982987663	0.959791608	-0.190732999
SNX15	0.055443709	-2.499789325	0.979110112	-0.02516101
SNX17	0.464171071	-0.378478775	0.482008628	0.098990099
SNX18	0.17643035	1.480327674	0.020200024	-0.569783439
SNX19	0.067926308	-2.301798464	0.066062191	-1.586785916
SNX20	0.782781736	-0.637909201	0.087606664	-0.492024421
SNX24	0.335367801	-1.899656552	0.52640026	0.126014039
SNX30	0.846604002	0.483891077	0.203336954	0.088305644
SNX31	0.663579311	0.880787952	0.950451714	0.186249058
SNX32	0.927445058	-0.650258673	0.539751881	-0.91937169
SNX33	0.16026015	0.355899789	0.695645345	0.180562918
SNX5	0.132261876	-1.567585083	0.048109844	-0.577499311
SNX8	0.722987495	-0.655121294	0.899547377	-0.343188567
SOCS3	0.377739638	-0.571172808	0.545451244	-0.358840466
SOCS4	0.331850709	-1.271717087	0.388827099	-0.037472552

SOCS5	0.228642189	−0.569913968	0.11273799	−1.523819084
SOCS6	0.352096865	0.156818343	0.752467495	0.385187507
SOCS7	0.677250756	−0.337209565	0.491803591	−0.163369484
SORBS1	0.135153528	0.301181381	0.202228444	−0.202489752
SORT1	0.784715267	0.821265764	0.871465496	0.340235092
SOX1	0.769736031	−0.385960964	0.555365405	0.250997748
SOX21	0.951074176	1.74000324	0.586162907	−0.370533005
SPACA3	0.521310321	−2.903758019	0.116256019	−0.522632857
SPATA5	0.535111047	−0.738202252	0.987215722	0.220218625
SPCS1	0.612234359	0.408825514	0.138452493	0.72132304
SPCS2	0.781712836	0.482052235	0.327198474	0.293771777
SPCS3	0.568013031	−0.301902847	0.249110536	−0.494516107
SPG20	0.577904295	−0.864701309	0.422354839	−0.109579872
SPG7	0.960414479	−0.068442353	0.284772473	−0.429656523
SPINK5	0.103248171	−0.732406821	0.050186334	−0.009824882
SPNS1	0.173448114	0.750747413	0.677822752	0.019008525
SPOP	0.000957332	−3.253351015	0.196751916	0.633515647
SPOPL	0.710724579	0.323726581	0.005334347	−1.604367058
SPPL2A	0.912552982	−0.253140508	0.246233223	0.593329475
SPPL2B	0.124764814	0.992655772	0.31764302	−0.180544584
SPRED2	0.703750432	−0.385545183	0.421451379	−0.181914942
SPRTN	0.032126197	−1.562120102	0.146535935	−0.265954945
SPSB1	0.061912519	1.269205654	0.135357916	0.690921495
SPSB2	0.55798895	−1.600903306	0.062460354	−0.572640191
SPSB3	0.438810232	0.992515404	0.180465896	−0.645259373
SPSB4	0.472576894	0.468734893	0.405390564	0.494019836
SQSTM1	0.498227149	0.409589907	0.65961256	−0.592529711
SRGAP2	0.694490284	−1.036560656	0.249110536	−0.457678128
SRGN	0.395747592	−2.557670321	0.497731448	1.055749326
SRP19	0.915033006	0.374849204	0.349675172	−2.319341905
SRP54	0.684701121	1.296097832	0.193250897	−1.397816709

SRP9	0.951074176	-0.319090974	0.294742668	0.342783194
SRPR	0.954186927	-0.613358334	0.916893568	-0.399463242
SRPRB	0.814302165	0.562328376	0.946095346	0.15484269
SRSF2	0.324504325	-2.262959972	0.237120634	0.570318593
SSPN	0.021495698	1.634531594	0.256242667	0.222072755
SSR1	0.048026771	-1.349148852	0.040077473	-1.29526088
SSR2	0.086579725	-3.449407085	0.061260369	-1.902037371
SSR3	0.619934041	-0.052118172	0.081055086	-1.233103227
SSR4	0.112490356	0.76474509	0.387537758	0.290547116
ST13	0.383258812	1.6002778	0.764960469	0.27526774
ST3GAL6	0.187169263	1.639220878	0.213800956	-0.540461384
ST5	0.315160071	-4.161076528	0.028211205	1.232245021
STAB1	0.22885313	0.775004988	0.307901358	-0.32985825
STAM	0.525380247	-0.208617318	0.226743606	-0.741826706
STAM2	0.68240539	0.712386033	0.410698577	-0.434854328
STAMBP	0.444376157	1.045447318	0.951074176	-0.237285458
STAMBPL1	0.778114213	0.096524573	0.31802169	-0.398940534
STARD10	0.74624501	0.647989612	0.652834683	0.16344558
STARD3	0.067808547	-2.070200237	0.03807434	-0.559296346
STARD4	0.916893568	-0.240422359	0.908215002	-0.396200743
STARD6	0.77392183	0.457041105	0.631561904	0.413013158
STAU2	0.703170367	-0.903153996	0.026884994	-1.361443805
STEAP1	0.342076436	-1.423753928	0.467996418	-0.423074405
STEAP2	0.384112279	-0.439084603	0.347665193	-0.47744387
STIP1	0.793741631	-0.259702885	0.000918074	-0.805001002
STMN2	0.366434229	-0.122123842	0.453741643	-0.344717012
STMN3	0.198929352	-0.289006172	0.137621992	-0.806391017
STOM	0.538202702	0.945393668	0.408479095	0.382513081
STOML2	0.788322413	-3.460406035	0.117716344	-0.848024679
STOML3	0.148536635	-2.036740731	0.13809218	-0.313681173
STON1-GTF2A1L	0.56325405	-1.967756888	0.027744635	-0.685098691
STRA13	0.211220593	-2.593440333		

0.086292869 -1.543217226
STT3B 0.398798559 0.167365028
0.772127102 -0.308681789
STUB1 0.799170825 0.544399221 0.283717932 0.324449967
STX12 0.45799217 0.797020647
0.016695346 -0.668565561
STX18 0.910073809 -0.925756686
0.009665162 -2.322308191
STX19 0.330606819 -0.228473203 0.05173318 0.885476497
STX2 0.302372058 -0.376625233
0.19892935 -1.311639712
STX3 0.847828364 0.25581164 0.695645345 0.100518872
STX4 0.658952025 -1.033872248
0.033984016 -0.828169248
STX5 0.425072022 -0.125618281 0.668124306 0.03156184
STX6 0.811270154 -0.408933167
0.63267415 -0.187801649
STX7 0.62601322 0.56053438
0.559565962 -1.606201293
STXBP5 0.008524091 2.770283092
0.45327074 -0.397191815
STXBP6 0.03182229 1.892469984 0.036927963 0.560195409
SUGT1 0.792929402 0.711836971
0.531002815 -0.014505956
SULT1A4 0.06121188 -0.856298707 0.151517143 0.428480896
SULT1C2 0.989086465 0.001799681
0.87916571 -0.436974292
SULT1C3 0.043777714 1.367275657
0.467996418 -0.205642248
SUMF2 0.032351361 -1.65125319 0.12943852 0.719565796
SUMO1 0.57598715 0.781511022
0.325657186 -0.461491724
SUMO2 0.429623277 0.470168407
0.000269706 -1.418204807
SUMO3 0.280396016 -0.57187125 0.747132934 0.247555232
SUMO4 0.365605271 0.980744108
0.593172958 -0.367732121
SUN2 0.492879226 -1.475663571
0.016784482 -0.826990429
SUPV3L1 0.058812952 -5.870181395
0.062790976 -3.062424739
SV2C 0.29474267 -1.934972959 0.21093528 0.602788826
SVIP 0.854568844 -0.337721495 0.675535882 0.120204976
SYCN 0.090230772 -1.770921208
0.213225585 -0.314574715
SYMPK 0.80098273 -3.239445047
0.994075379 -1.633715189
SYNCRIP 0.090574211 -3.427538434
0.129488821 -0.402081906
SYNE1 0.035195639 1.407862731

0.142690683 -0.006415618
SYNRG 0.929308465 1.132851895
0.002032226 -0.953658577
SYT10 0.466559871 0.22181821
0.029992157 -0.837338126
SYT11 0.888421012 0.656348113 0.955432226 0.164290139
SYT13 0.101744536 -0.3380728 0.793564816 0.08200301
SYT6 0.275721009 -0.79431727
0.816729781 -0.582807299
SYT7 0.604031979 -1.080698143
0.310877508 -0.961010571
SYT9 0.50319855 -0.840518217 0.148722027 1.268899837
SYTL1 0.136177865 -0.356841271
0.152495411 -0.244044436
SYVN1 0.230064826 0.57273444
0.868696359 -0.403384124
TAB3 0.204231264 0.705523945 0.328357543 0.653560841
TACR2 0.86562143 -0.54227765 0.437425034 0.229253576
TAGAP 0.364777485 0.43027886
0.264518064 -0.486667596
TANGO2 0.227645984 0.294005012
0.342871291 -1.099786371
TAPT1 0.19566977 1.442728788
0.431451674 -0.413149539
TASP1 0.381555367 0.081288313 0.631561904 0.047683902
TBC1D15 0.243379287 -0.754849237
0.071661142 -0.406787599
TBC1D20 0.346062512 -0.509934256
0.234957212 -0.263849434
TBC1D22A 0.508697209 1.297318219
0.034790357 -0.887062707
TBC1D24 0.450451123 -0.601275925 0.327198474 0.226840524
TBCA 0.238363179 -0.770248373
0.119933775 -1.379334981
TBCB 0.399235565 -0.641550981
0.342076434 -0.251204239
TBCC 0.322588799 0.371461487
0.049276821 -0.538506346
TBCCD1 0.67694582 -0.279062595
0.024261803 -0.253504401
TBCD 0.885334157 0.150070808
0.013206343 -1.656805501
TBCE 0.015374215 -3.138716072
0.730614867 -2.112071642
TBL1X 0.655655359 1.639763675
0.901403753 -0.273161928
TBL3 0.861319955 -0.341024416
0.280918764 -1.774627035
TCAP 0.237430842 -0.928732787 0.661878111 0.003869449
TCEB1 0.348066602 -1.020316676

0.046544779 -1.015012998
TCEB2 0.735911059 -1.316506401
0.078833707 -1.150430799
TCF25 0.721231202 -0.078673049
0.677250755 -0.358476012
TCIRG1 0.328357545 -2.983589527
0.952319189 -1.440413655
TCP1 0.024575381 -5.304491876
0.011598814 -4.920836234
TCP11L2 0.793741631 -1.984420113 0.308643622 0.02124902
TDRD3 0.970383429 -0.478410133
0.015573851 -1.402649723
TDRD5 0.333443166 -2.329783545 0.274259995 0.714841842
TECPR1 0.845685973 -0.21211719 0.461313519 0.048059545
TECRL 0.19438802 -1.508943164
0.225200871 -0.906565026
TEX261 0.001251143 -3.049403637 0.828712449 0.294725365
TFCP2 0.573855558 -0.53375453
0.957923147 -0.159745895
TFF1 0.894599756 -0.54273834 0.509700337 0.151779581
TFR2 0.06583217 0.518396741 0.126200533 -1.27408186
TGOLN2 0.451701974 0.536280455 0.142450525 -0.66990649
THAP5 0.226143443 0.549270143 0.088609327 0.477260693
THOC1 0.716554956 -1.229496931
0.568013029 -0.843831127
THOP1 0.898928707 0.204194994
0.738268972 -0.250520116
TIAL1 0.034123746 0.458921022 0.071292505 0.398249127
TIMM10 0.110556605 -0.821997095 0.775118986 -0.71460507
TIMM10B 0.700851836 -0.432433468
0.735911058 -0.169069107
TIMM13 0.539751883 -1.278987423
0.27468938 -0.257466961
TIMM8A 0.895217988 -0.142279569
0.700851834 -0.243605676
TIMM8B 0.688725656 0.523690263 0.086292869 0.231074998
TIMM9 0.003334852 1.455409897 0.014419257 -1.42133597
TINAG 0.503697127 1.826536023
0.183022695 -0.763978384
TINAGL1 0.94858451 0.471494913
0.651707798 -0.234544083
TLL1 0.621037432 -1.181515478
0.120680145 -0.619108392
TLR6 0.756030468 0.606386995
0.006232118 -0.960080243
TM9SF1 0.04369608 -1.960787246 0.264183551 0.025489141
TM9SF2 0.119933777 1.465028793
0.125620495 -1.088553766
TM9SF4 0.72533149 0.231921533 0.562726462 -0.67360912
TMBIM4 0.04945761 -1.563811307

0.166876066 -0.552232503
TMC6 0.028560932 1.468543164
0.094545018 -0.753421743
TMC8 0.188479186 -1.266445168
0.844156391 -0.314544577
TMED10 0.068636413 -1.530777961
0.008121227 -2.508551711
TMED3 0.871157736 -0.537092809 0.198383347 0.770026479
TMED6 0.855182149 0.467442442
0.947962171 -0.191803726
TMED7 0.633787229 -0.486257291 0.591551848 0.38251491
TMEM110 0.065947099 1.630165276
0.715971166 -0.058092522
TMEM115 0.819159157 -0.424567045 0.259200847 0.095178911
TMEM147 0.421000076 0.341670698
0.099018886 -1.060601884
TMEM165 0.306050873 -0.404226087 0.947962171 0.277815025
TMEM167A 0.770931295 -0.468695446 0.416951112 0.115624886
TMEM168 0.836212162 0.576459025
0.038879913 -0.611926525
TMEM169 0.761384644 -0.007607731 0.916273329 -0.41664277
TMEM17 0.68240539 0.819480439
0.570930732 -0.317323046
TMEM176B 0.136527986 -1.837434673
0.005651253 -1.347209279
TMEM178A 0.114809967 1.177289756 0.984721509 0.107861237
TMEM187 0.892745444 0.586272103 0.651707798 0.747717865
TMEM189-UBE2V1 0.633787229 -0.648704885 0.671541068
0.372921699
TMEM204 0.025005212 -2.005107806 0.201124344 0.058669254
TMEM205 0.098391582 2.216993333
0.007510463 -0.898983746
TMEM208 0.500212569 0.666929615 0.399235563 0.581935917
TMEM214 0.626567143 0.426290674
0.281267631 -0.222200342
TMEM230 0.28866179 1.50042163
0.408922419 -0.197290772
TMEM256 0.619382666 0.175097063
0.528954717 -0.162052177
TMEM258 0.204449884 -3.146433798
0.627675621 -1.982471316
TMEM27 0.065717407 2.691208501
0.083605872 -0.720602435
TMEM39A 0.617729826 -0.380446686
0.743583259 -0.085642734
TMEM5 0.155657255 1.529098505
0.441124483 -0.353104724
TMEM50A 0.268554999 0.890951924
0.546490691 -0.688971825
TMEM55A 0.876084211 -0.349656812

			0.653591567	-0.418026363
TMEM55B	0.546490692	-1.320773389	0.293304207	-0.484000412
TMEM63C	0.388397031	0.714717444	0.906976076	0.515171812
TMEM74	0.109512952	0.678935096	0.466081564	-0.278840077
TMEM87A	0.204589311	1.590424288	0.282315996	-0.761965027
TMEM9	0.374498613	-0.148644094	0.788924047	0.383437355
TMEM98	0.529466367	0.430944727	0.022215868	-1.05847517
TMPRSS11B	0.783513907	-0.744323758	0.284772473	-0.231881561
TMPRSS11D	0.185256117	1.422754042	0.693435864	0.229605116
TMPRSS11E	0.143089437	0.409015741	0.274346091	-0.979576787
TMPRSS11F	0.134338353	-0.754876448	0.435121974	0.41314398
TMPRSS12	0.715387545	-0.457483182	0.919375024	0.04688293
TMPRSS13	0.090230772	-1.827101767	0.484447379	0.738656855
TMPRSS3	0.135562546	0.358806443	0.252470692	0.417891817
TMPRSS4	0.450920368	0.279234228	0.097587538	0.354046792
TMPRSS5	0.699404166	-0.719069728	0.425980011	1.028143576
TMPRSS6	0.729439669	0.307323745	0.272634042	0.501320767
TMPRSS7	0.727678062	-0.280295053	0.742992174	0.235660789
TMPRSS9	0.608400447	0.382135694	0.130916208	0.682659911
TMTC2	0.932414999	-0.111587281	0.654526495	-0.052726081
TMTC3	0.197294633	-0.693247663	0.533054929	0.300540208
TMUB1	0.085635154	0.998294967	0.095016334	0.514294017
TMUB2	0.158162075	-0.549327871	0.17328006	0.361299072
TMX1	0.511206972	1.366775475	0.38968811	0.596957065
TNFAIP1	0.02145137	2.137539333	0.728265104	-0.178844208
TNFAIP8	0.110033801	1.946212896	0.059760791	-0.731531658
TNFAIP8L1	0.499219335	0.552075057	0.3838797	-0.095614259
TNNI2	0.244135053	-0.711067903	0.066095469	0.581573793
TNRC6C	0.00104344	-4.97218312	0.039695215	-1.006703221
TNS1	0.150488762	0.02998468	0.023719372	-1.132587825
TOLLIP	0.531002817	0.583685603	0.694490283	-0.231695433
TOM1	0.950451714	0.642280925	0.679539855	0.216814364
TOM1L1	0.292378178	0.424650466	0.374023179	-0.27128208
TOM1L2	0.028885556	0.49509268	0.281616792	0.424937166
TONSL	0.001417804	-8.120181775	2.23E-07	-6.486412689
TOR1A	0.171971197	1.627431172	0.00706244	0.928896474

TOR1AIP2	0.46083822	0.039198535	0.741810458	−0.2260566
TOR1B	0.224946515	2.134660293	0.212078199	0.173542307
TOR2A	0.677822753	−0.525290638	0.217568181	−1.049566975
TOR3A	0.17618038	0.815399104	0.165918563	−0.298472209
TOR4A	0.470876853	0.540723544	0.892127469	0.302965326
TP53I11	0.08379046	−0.996223159	0.007920279	−0.984589133
TPH2	0.55222513	0.143727944	0.49575128	−0.082997098
TPP1	0.770333595	−0.308947103	0.270249477	0.341300404
TPP2	0.680685517	−2.368585048	0.089049192	−2.449229298
TPPP	0.02614099	2.671606681	0.080084791	−0.953668503
TPRA1	0.067393979	1.332754591	0.226658995	0.23689935
TPRKB	0.388827101	0.741185541	0.463694128	−0.514616845
TPSG1	0.939252932	−0.310948447	0.327198474	−0.053219786
TPT1	0.017774088	−1.61019888	0.278831733	−1.47843691
TRAF3	0.153393767	−1.294640585	0.312747208	0.351668676
TRAF4	0.879782232	−1.058787665	0.100305216	−0.849337673
TRAF5	0.38796725	−0.555278344	0.487382686	0.648144644
TRAF6	0.181485416	0.414241605	0.105921385	1.081995919
TRAF7	0.951696668	0.692436349	0.080987035	−0.18872979
TRAIP	0.009599576	−3.660267774	0.000158586	−2.490834923
TRAM1	0.104411028	0.486166819	0.810058138	−0.2911255
TRAP1	0.568542992	−0.593670218	0.116984392	−0.462723518
TRAPPC8	0.479090964	−0.479413844	0.027593172	−0.908078492
TRHDE	0.086867349	1.186547176	0.103248169	0.509425744
TRIM10	0.663012051	0.482171243	0.89150956	−0.069402519
TRIM11	0.591859161	1.563757713	0.464486112	−0.14020477
TRIM14	0.961037373	−0.804375249	0.430536911	−1.487423012
TRIM15	0.235882677	−1.227016857	0.089490815	0.760602057
TRIM17	0.736500305	0.054324648	0.039400052	−0.715094868
TRIM2	0.471561321	−0.708488726	0.627645664	−0.615618691
TRIM21	0.447641465	−0.419661486	0.376472969	0.492141741
TRIM22	0.616078919	−0.149237914	0.215244281	−0.435860178
TRIM23	0.168803525	−1.768717616		

0.792536488 -0.009986484
TRIM25 0.087589784 -0.265422099
0.974123063 -0.155775666
TRIM26 0.568013031 -0.473528735
0.206970157 -0.350498557
TRIM27 0.674964633 0.418369835 0.851503642 0.085349526
TRIM29 0.367264359 -2.579129352 0.805214658 0.103860067
TRIM3 0.82098233 -0.03367725
0.038146983 -0.289096089
TRIM31 0.81855165 -0.19052437
0.513723184 -0.440557521
TRIM32 0.131515154 -1.111077389
0.518774881 -0.326770628
TRIM34 0.621589448 0.541118328 0.471117328 0.169302222
TRIM35 0.474008332 0.921183864 0.429166879 0.194937242
TRIM37 0.428938789 -1.295763408
0.064353066 -0.010146498
TRIM38 0.485888329 -1.881823185
0.245686873 -0.229296212
TRIM39 0.019092722 -3.048674673
0.774520341 -0.347986309
TRIM4 0.539751883 -2.824896391 0.893981587 0.026985694
TRIM40 0.649456418 0.634461486 0.959168763 0.010715675
TRIM41 0.628230177 -0.054003544 0.204171236 0.365103679
TRIM43 0.073527823 1.818335591
0.012066187 -1.002096748
TRIM43B 0.036343567 1.847713684 0.356166248 0.761153617
TRIM45 0.966021352 -0.284579522 0.900784901 0.422672221
TRIM46 0.686712278 -2.929934285
0.186908085 -0.631017576
TRIM47 0.555889633 0.268445294
0.250074843 -0.643855627
TRIM48 0.00243302 1.958952136 0.30605087 0.559691461
TRIM49 0.306420379 0.539396882
0.081259527 -0.878216501
TRIM49B 0.097163558 1.139029405 0.138899516 -2.04901585
TRIM5 0.062350468 0.951380073
0.308272343 -0.584841277
TRIM50 0.180465898 -0.708704609 0.321824656 0.656308307
TRIM51 0.501544926 -0.676208301
0.166117753 -1.213354018
TRIM52 0.34927259 -1.197949557
0.310877508 -0.068902852
TRIM54 0.206970159 0.624600005
0.802795706 -0.258943631
TRIM55 0.226743608 1.473150951 0.076207795 0.701590261
TRIM56 0.334975832 -1.078024561 0.990333661 0.047583204
TRIM58 0.939874792 -0.267359982
0.253958131 -0.010649754
TRIM59 0.119376375 -1.32937347

0.233727264	-1.288943833
TRIM6	0.852116505	0.958755663
0.072155085	-1.230755463
TRIM6-TRIM34	0.719476377	0.251635279
0.784715266	-0.22241431
TRIM60	0.120493214	0.550277493	0.279873926	-0.25472766
TRIM61	0.959168763	0.261512756	0.626013218	0.034540814
TRIM62	0.350884695	-1.227596176
0.820982329	-0.141381535
TRIM63	0.416054474	0.425524987
0.300543725	-0.628234421
TRIM64	0.745357422	0.568651136	0.884099899	0.110911143
TRIM64B	0.165679836	-1.879094039	0.981603955	0.097156344
TRIM64C	0.841710285	0.05042889
0.663863009	-0.301729457
TRIM65	0.642161595	-1.148678417
0.324120627	-0.694001225
TRIM66	0.178439636	0.847915037	0.03608759	0.296829094
TRIM67	0.074539533	-3.126608614
0.111081368	-0.781900595
TRIM68	0.781712836	0.387936904
0.701431207	-0.193815036
TRIM69	0.056044048	2.065365166	0.797963487	0.148572646
TRIM7	0.92558205	0.394512565
0.479090962	-0.413853696
TRIM71	0.676678945	-0.497845616	0.031043441	0.363951494
TRIM72	0.18508739	-1.802924408	0.241489621	0.327684259
TRIM73	0.051711184	1.276790423
0.413926898	-0.013027277
TRIM74	0.386679648	-1.925095046	0.066062191	0.589341627
TRIM77	0.694490284	-0.372268855	0.179259604	0.684230043
TRIM8	0.723573251	-0.868030402	0.499715819	0.286973253
TRIM9	0.124466655	-3.861243078
0.096329156	-0.801677785
TRIML1	0.688150179	-1.467126833	0.810664089	0.097853044
TRIP10	0.245279318	-1.262194865
0.088171214	-1.077680649
TRMT112	0.471839167	-0.08976044
0.198110757	-1.038853051
TRMT6	0.881015492	-0.197306867
0.840487802	-0.183530108
TRNP1	0.581868219	0.085478778
0.662444984	-0.257633949
TROAP	0.00243302	-4.021808122	1.13E-05 -3.20517577
TRPC4AP	0.423712157	-0.266612085	0.431909478	0.296331426
TSC22D1	0.780512771	0.105361465	0.412479308	0.233069125
TSEN2	0.150932936	-4.291262296
0.006027993	-2.440566308
TSEN54	0.125042539	-0.838926094
0.060399697	-0.482687572

TSFM	0.586162908	-1.237863711	0.492296128	-0.008133004
TSKU	0.00688236	2.175949731	0.802191262	-1.395722672
TSNARE1	0.354529147	1.054632915	0.37394751	0.502760731
TSPAN13	0.83255117	-0.29193383	0.090826273	0.986445656
TSPAN17	0.029231149	1.986633184	0.538718846	0.217770497
TSPAN8	0.1414295	-4.581399808	0.213006581	-0.422899736
TSTD1	0.502021125	-0.058980445	0.790729706	-0.26175635
TTC1	0.019866235	-7.618786515	0.034322573	-6.292412372
TTC14	0.59155185	-0.464956547	0.600764902	-0.3673122
TTC16	0.003087814	-2.441957736	0.557463758	-0.82071245
TTC18	0.927445058	1.332998543	0.160959367	-0.22077533
TTC24	0.74121983	-0.280278719	0.669262467	0.759206821
TTC28	0.999688175	-2.62866603	0.577054344	-0.26063102
TTC3	0.17394252	-0.884165106	0.020136464	-0.581173065
TTC30B	0.282315998	0.041557584	0.55484142	0.438341833
TTC32	0.506694059	-1.2015181	0.699114762	0.202182059
TTC36	0.932414999	1.015546652	0.554317678	-0.204109775
TTC39A	0.079904477	0.584706679	0.921857266	0.456301925
TTC4	0.31087751	-0.847878464	0.787119521	-0.70319411
TTC6	0.525380247	-0.915445594	0.587777197	-0.607936195
TTC7B	0.105584263	-0.6861093	0.494762774	0.118828379
TTC9	0.481521682	-1.62241761	0.166756151	-0.31798739
TTC9B	0.774520341	0.537482577	0.919995512	0.27527079
TTC9C	0.847828364	-0.859366526	0.932414998	-0.078334495
TTF1	0.051483257	-1.292189904	0.478120554	-0.908259176
TTLL2	0.086239347	-0.822845089	0.845452505	0.453794161
TUBA3D	0.34406579	0.785644208	0.969760218	-0.0811711
TUBB2A	0.297996467	-0.862519654	0.176931082	0.248420326
TUBB4A	0.247133929	0.753587716	0.043229555	-0.713693815
TUBB4B	0.537686804	-0.565798196	0.303840036	-0.717150132
TUBB6	0.166636304	-1.921522491	0.048916908	-0.635656921
TULP4	0.084587828	-0.189371966	0.478605623	-0.336119011
TVP23B	0.911933108	0.329039689	0.827066508	0.079264553
TXLNA	0.360656138	1.239473183	0.00345915	1.177884284
TXN	0.533568585	-0.706869523	0.088171214	-0.195307627
TXNDC11	0.270300403	0.658845793		

0.688438836 -0.183062182
TXNDC5 0.335760065 0.610840598 0.887803504 0.166237419
TXNL1 0.131515154 0.551741039
0.281267631 -0.806359005
TYSND1 0.852729457 0.456892637
0.02174093 -0.578815994
UBA2 0.197566401 -0.517600501
0.001922854 -1.343786359
UBA5 0.72415917 0.447868396
0.242433158 -0.916222294
UBA52 0.126200535 -2.132880527 0.617179307 0.133476937
UBA6 0.177935725 -0.221632682
0.527421286 -0.240794372
UBA7 0.566424568 -1.103814077 0.617729824 0.188035655
UBAC1 0.700851836 -0.565562613
0.464648284 -0.158391742
UBAC2 0.462741066 -1.298462026 0.592632362 0.241926849
UBAP1 0.618280558 -0.345759367
0.189795856 -0.998070496
UBAP2 0.472320731 0.208877997
0.578656882 -0.320550773
UBAP2L 0.29222846 1.835630065
0.000918074 -1.822924913
UBASH3A 0.086436202 -1.543799276
0.422354839 -0.356736629
UBASH3B 0.452800114 0.668354865 0.486892802 0.466118323
UBD 0.296547384 -1.241326699
0.336545478 -0.273182586
UBE2D1 0.143730873 -1.159450509
0.091125208 -0.919954541
UBE2D3 0.073402153 -1.27066397
0.131964249 -0.716919042
UBE2D4 0.955432226 0.445568989
0.921857266 -0.458950509
UBE2E2 0.002351572 -2.598816403 0.355021227 0.24939416
UBE2E3 0.197217042 0.007353509 0.230368459 -0.30968619
UBE2F 0.051109877 0.641595165
0.840487802 -0.217145569
UBE2G1 0.990333661 1.569666917
0.163779298 -0.365626046
UBE2G2 0.561145137 -0.353764415
0.668124306 -1.115435454
UBE2H 0.696801115 -1.984020158
0.269909996 -1.300959628
UBE2J1 0.334975832 0.690455681
0.375629983 -1.070557959
UBE2J2 0.588315752 -1.929515415
0.101931823 -0.585981903
UBE2K 0.181485416 -1.655658695
0.047150282 -0.803784119

UBE2L3	0.547531119	0.429332006	0.522834637	-0.233303654
UBE2L6	0.671541069	0.351255751	0.943606709	0.224673061
UBE2NL	0.884716993	-0.443854784	0.88595139	-0.474770365
UBE2Q1	0.808240971	0.631708059	0.167837717	-1.448627492
UBE2Q2	0.080579689	-2.277955187	0.610042241	0.696043476
UBE2QL1	0.322843683	-1.53258273	0.17686975	-0.247485631
UBE2S	0.126200535	-1.989365438	0.340886361	-0.425831175
UBE2T	0.971941536	0.780564898	0.21179205	-0.852870113
UBE2U	0.05624536	-1.079547141	0.622141675	0.161556133
UBE2V1	0.799170825	0.094147186	0.116256019	0.145734569
UBE2W	0.444376157	1.154886448	0.447174157	0.256865765
UBE2Z	0.541303294	1.027274131	0.102588317	0.983793445
UBE3A	0.209512907	-2.493343236	0.134635457	0.442611015
UBE3B	0.259737763	0.87859301	0.321866686	-0.319449627
UBE4A	0.171725962	1.668484851	0.090230771	0.547276664
UBE4B	0.334192779	-0.631229471	0.636573544	0.141585743
UBFD1	0.176680585	1.832917736	0.252335023	-0.324513982
UBL3	0.788322413	-0.844338675	0.488363252	-0.177856221
UBL4A	0.498723111	0.806790417	0.37268872	-0.420608263
UBL4B	0.004252342	1.796371493	0.8386548	0.236898946
UBL5	0.985345049	-0.161175033	0.079904476	-0.396474003
UBL7	0.564309938	-0.441896091	0.031073937	0.78947229
UBLCP1	0.457518789	-0.078987137	0.540785908	-0.190791249
UBOX5	0.511709699	-0.361815021	0.136383448	0.096519197
UBQLN1	0.241175686	-1.192651252	0.743583259	0.181264375
UBQLN2	0.855182149	-0.09876557	0.477151221	-0.233543019
UBQLN3	0.788322413	-0.062319726	0.759598633	-0.111620201
UBQLN4	0.891509561	0.087965636	0.753654571	0.4458067
UBQLNL	0.217859935	0.353912519	0.470876851	0.667538676
UBR1	0.924961137	-0.776712137	0.165918563	-0.191751774
UBR2	0.866236255	0.433374144	0.342473715	0.517696599
UBR3	0.679359269	0.482443571	0.088373809	-0.668729055
UBR4	0.538718848	-1.847711652	0.0304389	-2.199498811

UBR7	0.822198317	0.70631801	0.937387575	−0.192104754
UBTD1	0.562199116	−1.103560595	0.004007529	−1.031689475
UBTD2	0.882249041	0.428856624	0.158406794	−0.479095006
UBXN1	0.874852135	−0.010156321	0.053577885	−0.855748551
UBXN10	0.206970159	−1.900602011	0.757813905	−0.254296138
UBXN11	0.449513462	−1.343686703	0.154975561	−0.783701366
UBXN2A	0.195939897	−0.403035561	0.205567235	−0.534712503
UBXN2B	0.741810459	−1.013415685	0.238674529	−0.810435644
UBXN4	0.356564133	−2.742463395	0.717138908	−1.280442398
UBXN6	0.237430842	−1.025747613	0.107619829	−0.874827132
UBXN7	0.951038186	−0.600833696	0.560365188	0.227530828
UBXN8	0.084523965	1.528389532	0.101858399	−0.359038285
UCHL3	0.174934489	−0.56970426	0.868696359	0.231517051
UCHL5	0.116437779	−1.233993802	0.00163071	−2.179385697
UCN2	0.083885485	−1.496903287	0.530490414	−0.240464071
UEVLD	0.150710724	0.346032884	0.662444984	0.012673102
UFC1	0.346062512	−1.416291535	0.460363191	0.096666727
UFD1L	0.935522562	0.34442593	0.949206881	−0.622848092
UFL1	0.777514902	−0.596717656	0.885334157	−0.267160036
UFM1	0.8460978	0.472016826	0.966142811	−0.363568917
UGT2A1	0.366019604	−1.412165687	0.74240124	−0.351509945
UGT3A2	0.965485796	−0.922706854	0.212844333	−0.777237336
UIMC1	0.119391947	−1.00489317	0.242433158	−1.443172769
UNC13A	0.146519889	2.126475654	0.162845068	0.332878307
UNC13B	0.087880112	−0.455229959	0.25168781	−0.986663136
UNC13D	0.131515154	−1.469609154	0.158637572	−0.841830412
UNC45A	0.306790181	−2.8300353	0.015078983	−1.885180819
UNC45B	0.411143333	−0.643901121	0.208662821	0.880435978

UNC50	0.626567143	-0.849112414	0.066062191	-3.12677679
UNK	0.679539857	-0.103452704	0.531002815	-0.790420355
UNKL	0.861934203	-1.877879399	0.312747208	-0.660375298
UPK1A	0.258870999	-0.503462356	0.160260149	0.290102337
URI1	0.44700731	-0.541713822	0.010615997	-0.914740879
URM1	0.550658267	0.971542271	0.118083665	-0.809400721
USE1	0.313122038	-1.461151662	0.000311834	-2.843608012
USO1	0.913792889	0.544531508	0.577588291	0.163026646
USP10	0.68527551	-0.231567194	0.094851726	-0.482220139
USP11	0.80581969	-0.347302095	0.62324677	-0.519626342
USP12	0.098539921	2.270290487	0.193518571	0.088773939
USP13	0.909443931	0.798406336	0.647656409	-0.141900828
USP14	0.492788931	-1.583530404	0.11753302	-2.174159754
USP15	0.224649571	-1.713311549	0.746595708	-0.238006922
USP17L2	0.745357422	-0.655676794	0.384539446	-0.144566157
USP17L24	0.414711649	-1.25985889	0.850890869	0.405284721
USP17L25	0.742992175	-0.470752121	0.608947492	0.405284721
USP17L26	0.009298722	-1.331605083	0.194591988	0.672520783
USP17L27	0.014661356	-1.488963685	0.551702597	0.405284721
USP17L28	0.51624583	0.361307796	0.445307711	0.672520783
USP17L29	0.178691989	-1.25985889	0.834991432	0.644744003
USP17L3	0.361889463	0.130922962	0.896454644	0.255741971
USP17L30	0.093239962	-1.488963685	0.443910795	0.672520783
USP17L4	0.827066509	0.202181422	0.591551848	0.319525773
USP17L5	0.032695537	-1.331605083	0.916893568	0.405284721
USP17L7	0.945473137	0.161635017	0.410698577	-0.182730605
USP17L8	0.566424568	-0.295557602	0.661878111	-0.473093512
USP18	0.367679863	0.223289841	0.394010528	-0.23408231
USP19	0.959168763	-0.815912507	0.732967162	-0.309704056
USP20	0.48200863	-0.876360816	0.041618291	-1.123240928
USP21	0.668124307	0.527798151	0.5107045	-1.810208698
USP22	0.020158035	-1.484438116	0.23067238	0.274726245
USP24	0.187430708	-1.331559756	0.75068798	-0.092941633

USP25	0.425980013	1.795368373	0.141389677	-0.65185398
USP26	0.060292822	-3.536582342	0.072624587	-1.046864687
USP27X	0.152944089	-0.494823388	0.710142463	0.00976406
USP28	0.549093595	-0.105121673	0.016290655	-1.684144276
USP29	0.523343253	-0.522280443	0.152944087	-0.562404118
USP3	0.346462741	0.423521825	0.143089435	1.029960932
USP30	0.866851161	-0.234578138	0.156112978	-0.406118581
USP31	0.120399833	-1.837122829	0.177684166	-1.271761532
USP32	0.289741764	0.071940219	0.261973091	-0.263024469
USP33	0.93857347	0.641337293	0.465493226	0.025387923
USP34	0.443445715	-0.558593501	0.534082488	-0.937630238
USP35	0.434662206	-1.027863194	0.967579127	-0.13148028
USP36	0.596421269	0.121334129	0.764364148	0.250665905
USP37	0.055145555	2.049711562	0.709560518	-0.177389336
USP38	0.853955627	0.02877618	0.655090828	-0.211333365
USP39	0.03314431	-4.216890276	0.023335448	-4.196125987
USP4	0.172954759	0.682549173	0.665282244	0.194933257
USP40	0.471839167	0.253825418	0.412033697	-0.615094064
USP41	0.198656213	-0.68715628	0.167356372	-0.383082441
USP43	0.233179261	0.253823936	0.458968717	0.501397581
USP44	0.081397238	1.376850879	0.364824286	-0.450033737
USP45	0.471357875	-0.753837168	0.7212312	-1.290677886
USP46	0.587238873	-2.341367195	0.012527898	-0.945419659
USP47	0.387038423	0.687328642	0.617740851	-0.195978895
USP48	0.828284604	-2.446699939	0.737679262	0.171447891
USP49	0.035536445	-1.269587053	0.05685291	0.535862225
USP5	0.828284604	-0.496653245	0.59587932	0.631405317
USP50	0.921236634	-0.582104131	0.855182149	0.102761554
USP51	0.831941355	-0.708383339	0.018973025	1.450698736
USP53	0.763209622	0.277493249	0.376719247	0.178942973
USP54	0.622694118	0.326419079	0.519788291	0.12164173
USP8	0.389257462	-0.634387172	0.022676553	-1.663752163
USP9X	0.811876331	0.303650265		

0.66301205 -0.076979696
USP9Y 0.386679648 0.774148636 0.194054735 0.757115669
USPL1 0.201675845 -1.505257036
0.345262937 -2.752572129
UVRAG 0.844768154 -0.489903924
0.001105784 -1.488891739
VAC14 0.180866129 1.625525673 0.247366994 -0.33388958
VAMP4 0.416951114 0.521947829 0.958545942 0.209847559
VAMP5 0.652271143 0.039768328
0.820374497 -0.227024525
VAT1 0.671541069 -0.358760515
0.902022665 -0.644492436
VAV3 0.017811702 -2.278801839 0.482983326 0.08270779
VBP1 0.452800114 -4.68911909
0.023767746 -3.041941821
VCPIP1 0.419197696 0.076746239 0.013587232 0.559972313
VCX 0.372688722 -1.083240298
0.675535882 -1.035764481
VCX3B 0.248789681 -1.417588479 0.103911392 0.096596427
VIMP 0.408922421 -0.739634934
0.275032962 -0.410956104
VPREB3 0.671541069 0.08745768 0.464648284 0.264725038
VPS11 0.538202702 -3.640646114
0.102013701 -0.968902599
VPS13A 0.706653339 0.850075418 0.623799636 -0.28398234
VPS13C 0.663579311 0.29270124 0.160492966 0.445841889
VPS13D 0.716554956 -1.117648286
0.058812951 -1.819446521
VPS16 0.908834549 -0.050249134
0.000611927 -1.314090633
VPS18 0.22375412 1.458935845 0.979110112 0.151825065
VPS25 0.130717032 0.468864818 0.116801964 0.511870322
VPS26A 0.138252924 -1.067284007 0.192975166 0.181674309
VPS26B 0.977239863 0.252234866 0.679539855 0.235764454
VPS29 0.396182579 0.081637886
0.028036924 -1.926975116
VPS33A 0.590472242 -0.654548098
0.467996418 -0.356262891
VPS33B 0.143516816 0.43189679
0.051249627 -0.646694239
VPS35 0.665850275 1.851702215 0.290441439 0.030583998
VPS36 0.84599196 -1.839199884 0.810058138 -2.20518169
VPS37A 0.947962171 0.231008809
0.420098316 -0.160400637
VPS39 0.379008927 -0.83389425 0.21293832 0.091175817
VPS41 0.966021352 -0.40903509
0.382832513 -0.384249256
VPS45 0.333020415 1.061363976
0.300178945 -0.272669058
VPS4A 0.149162221 -3.115857399

			0.011014152	-0.908080924
VPS4B	0.816729781	0.127562312	0.092935464	-0.753230742
VPS53	0.794947265	-0.88384986	0.802795706	0.400779842
VPS54	0.607853619	0.293489543	0.726504454	-0.442154407
VPS8	0.981603955	-1.030860231	0.50569404	-0.58102419
VSX2	0.189795858	0.999275687	0.058812951	-1.213984344
VTI1A	0.018992929	-2.077898258	0.177181845	0.828136974
VTI1B	0.211506183	1.326721534	0.185866074	-0.719911915
WBP11	0.308272345	1.302315112	0.00060318	-1.521394276
WDFY2	0.751281006	-1.59164625	0.157946001	-0.70916347
WDFY3	0.16026015	-1.848058201	0.205008005	0.173857716
WDPCP	0.508196033	-0.70643918	0.918754586	-0.283358471
WDR11	0.180720378	-4.910441937	0.164727502	-0.153875896
WDR16	0.667555512	-0.492220862	0.104077725	-0.661959342
WDR18	0.444376157	-1.556140036	0.206970157	-0.294164222
WDR24	0.280824442	0.173081592	0.00236522	-2.234540787
WDR25	0.916893568	-1.063132817	0.007383475	-0.943310014
WDR3	0.133122725	0.797250608	0.049820854	-1.214353609
WDR31	0.155458316	-2.279329953	0.712253934	0.306428193
WDR45	0.577054346	1.227684432	0.450920366	0.548634704
WDR48	0.629339917	1.51561487	0.476666958	-0.851565698
WDR49	0.271951275	-0.376359431	0.264183551	-1.644179905
WDR59	0.045522002	-1.516634113	0.102423879	-0.997931627
WDR6	0.527421288	0.494675834	0.434202714	0.455165752
WDR7	0.479090964	0.4206149	0.527421286	-0.183221578
WDR78	0.700614914	-3.188601501	0.317228292	-0.170412634
WDTC1	0.518268561	-3.31453122	0.698536087	0.305748626
WFDC2	0.00225649	1.807382838	0.56854299	-0.318128866
WFIKKN1	0.393143732	-0.886225885	0.222862792	-0.417887762
WIPI1	0.917513857	-0.535523797	0.271269676	0.079107338
WIPI2	0.209796825	-0.995666567		

			0.346462739	-0.435826352
WSB1	0.441124485	-0.851788831	0.738858836	0.125654792
WSB2	0.759003582	-1.340470819	0.927445058	0.601104706
WWP1	0.020670525	1.378938496	0.049856842	-1.012742509
WWP2	0.172954759	-0.862372858	0.190324418	-0.299578649
XBP1	0.400986467	0.874258782	0.942362595	0.010280081
XKR9	0.190854066	-1.05545543	0.043533197	0.824386726
XPNPEP1	0.061044255	-3.756847547	0.427799385	0.151322836
XPNPEP2	0.623799638	-0.754531228	0.748317356	-0.040068393
XPNPEP3	0.318400659	-1.411996377	0.790127694	-0.390166231
XPOT	0.335367801	-1.990986935	0.802795706	-0.265449686
XRCC6BP1	0.681258624	0.7897684	0.003591684	-0.902379908
XRRA1	0.895836285	-0.167795473	0.179704054	0.552174822
XXYLT1	0.738858838	-0.837667479	0.134135152	0.034214535
YBX1	0.382530809	0.742123305	0.904545345	-0.180719688
YIF1A	0.143089437	-0.429442591	0.606214451	-0.194384057
YIPF1	0.946717589	-1.057790504	0.241803846	-0.272230573
YIPF2	0.350884695	0.519576465	0.732378852	-0.356535162
YIPF3	0.214666113	1.111135287	0.252011272	-0.475691404
YIPF5	0.882865923	0.559788275	0.839265704	0.476469065
YIPF6	0.486403187	-0.025535504	0.388397029	0.180709264
YKT6	0.011854179	1.485327791	0.085579091	-1.06618209
YME1L1	0.40803606	2.149682565	0.10882152	0.720336896
YOD1	0.520802723	0.155686622	0.447641463	0.143254521
ZBTB40	0.473284669	0.138056432	0.321443029	-0.061066884
ZBTB5	0.280744442	-0.214515502	0.88163223	-0.459553381
ZC3H7A	0.55798895	-0.348097636	0.241803846	0.523878465
ZC3H7B	0.680685517	0.560654992	0.390550278	0.467400386
ZCCHC7	0.863162948	1.148937928	0.007699626	-1.202190561
ZCWPW1	0.839265705	-0.175202033	0.630450492	0.045481395
ZDHHC15	0.392278091	0.019998603	0.483959094	0.241370994
ZDHHC17	0.436502971	0.393941213	0.306420377	-0.652246502
ZDHHC2	0.695067727	0.868373642	0.435582025	-0.592089665
ZDHHC3	0.381555367	-0.607004843	0.943606709	0.33227956

ZDHHC8	0.756624803	0.299430634	0.354935554	0.161633105
ZER1	0.055543392	0.568119276	0.005774404	-1.297634205
ZFAND2A	0.920616049	2.150367883	0.106937882	-0.284287862
ZFAND2B	0.068636413	1.664905367	0.073758645	-0.644118861
ZFAND3	0.498723111	-0.551042527	0.392278089	-0.191386987
ZFAND4	0.066177451	1.482257578	0.21179205	-0.807535836
ZFAND5	0.197294633	-0.691833684	0.115170145	0.922923694
ZFAND6	0.25854144	1.311793071	0.118111571	0.645642842
ZFC3H1	0.657915442	-0.087852955	0.370596579	-0.859839986
ZFP36	0.872388894	0.418355453	0.044352763	0.618102513
ZFP69B	0.602397444	-0.362180189	0.92682401	-0.416084988
ZFPL1	0.710724579	0.00214856	0.190192173	-0.410336676
ZFYVE16	0.064805226	1.471389664	0.724745248	-0.295128113
ZGPAT	0.506193921	-0.060149176	0.1975664	-0.987421713
ZIC3	0.636015869	0.099645881	0.316131295	-0.251636737
ZMPSTE24	0.989398262	0.324030328	0.11020785	0.172862037
ZNF146	0.918134197	-1.812021441	0.035111161	-0.604276121
ZNF330	0.729439669	0.764119346	0.082494963	-0.529499237
ZNF436	0.281442176	0.68177499	0.334584156	-0.254741492
ZNF443	0.257226112	0.55635019	0.2621826	0.324456394
ZNF473	0.2907351	0.992360684	0.138880476	-0.281045343
ZNF598	0.6298951	-1.561504402	0.027538131	-1.20955894
ZNF609	0.400986467	-0.912221089	0.309015198	-0.604575604
ZNF645	0.042407324	1.596506066	0.160959367	-0.263742051
ZNF708	0.860091711	0.832139178	0.439272522	-0.115965025
ZNF85	0.934640223	-1.732581491	0.068846261	-0.79933486
ZNFX1	0.643281654	-0.112849132	0.43673338	0.240429346
ZNRD1	0.76675027	-0.317510311	0.271610328	-0.650556272
ZNRF1	0.450920368	-0.644243897	0.026403959	-0.161929899
ZNRF2	0.678967303	-0.323739198	0.189531981	0.384601596

ZNRF3 0.12671545 0.773477437
0.107989059 -0.477297505
ZNRF4 0.405825169 -0.611416486
0.433743505 -0.167595194
ZRANB1 0.571196336 0.720222981
0.001852971 -0.930287186
ZRANB3 0.045691138 -1.556166273
0.471598485 -0.092741634
ZSCAN18 0.550136466 -1.682231104 0.150267048 0.60033329
ZSWIM2 0.379008927 -0.433723192 0.945162044 0.114418464
ZYX 0.261186063 1.329355863
0.062131173 -0.347992373
pseudo_0 0.483959091 -1.065358287
0.606760617 -0.034973099
pseudo_1 0.414264605 -0.448444754 0.189268372 0.836153948
pseudo_10 0.33733207 -0.379902799 0.238674524
0.633569808
pseudo_100 0.803400267 -0.48760176 0.31312203
0.583606676
pseudo_1000 0.808846577 -0.974022954
0.372688715 -0.167786569
pseudo_1001 0.838654799 0.519086865 0.843544721
0.365622797
pseudo_1002 0.230064821 1.772134962
0.57066519 -0.072795536
pseudo_1003 0.440197941 0.615907768
0.898310097 -0.18530425
pseudo_1004 0.94174059 -0.515552903
0.859477714 -0.237613959
pseudo_1005 0.733555627 0.644498027
0.016360409 -0.763745472
pseudo_1006 0.288661784 -1.488886469 0.307530665
0.386453494
pseudo_1007 0.983474456 -0.239048112
0.0339259 -0.708107772
pseudo_1008 0.035127812 1.283506857
0.365605264 -0.32061411
pseudo_1009 0.585087854 -1.550431867 0.514227193
0.326816406
pseudo_101 0.44764146 0.282355948 0.646085339
0.034728882
pseudo_1010 0.241175681 0.677886986 0.453741638
0.306383502
pseudo_1011 0.969137025 0.300141657
0.02928909 -0.718428968
pseudo_1012 0.101142666 1.832628828 0.519234098
0.295662648
pseudo_1013 0.820050574 -0.592321768
0.890891716 -0.143114863
pseudo_1014 0.086867346 -2.137747894 0.224349744

0.597630869
pseudo_1015 0.968513851 -1.428749391
0.020368744 -0.695505411
pseudo_1016 0.32913173 -0.304454866 0.720061151
0.282908133
pseudo_1017 0.140967169 1.07850485
0.797963485 -0.169723681
pseudo_1018 0.074921841 1.602077447 0.295824606
0.22323475
pseudo_1019 0.067339177 2.277650596
0.721816466 -0.269502546
pseudo_102 0.380280832 -0.639176521 0.748317353
0.051894336
pseudo_1020 0.257226107 -5.272092953
0.735911055 -0.19691162
pseudo_1021 0.717723026 -0.195533015 0.174934482
0.190752128
pseudo_1022 0.65339842 -0.208711396 0.844768152
0.121133544
pseudo_1023 0.577588289 0.340129392
0.96166029 -0.105787902
pseudo_1024 0.514731469 1.470973526
0.512212678 -0.370099628
pseudo_1025 0.027264345 1.585933934
0.884099898 -0.078918035
pseudo_1026 0.486403182 -0.045227569 0.70607241
0.114324777
pseudo_1027 0.4868928 2.266026908
0.037857116 -1.014070775
pseudo_1028 0.401424907 -0.148435825 0.056852908
0.384491441
pseudo_1029 0.970383429 -1.034594933
0.091125204 -0.608770701
pseudo_103 0.499138319 1.71185379 0.924486948
0.153100492
pseudo_1030 0.349675169 0.625948711
0.735911055 -0.005681416
pseudo_1031 0.370179025 1.300357306 0.245279311
1.183017887
pseudo_1032 0.663012048 -0.46919752
0.877316586 -0.409675495
pseudo_1033 0.377739633 2.005548576
0.778114209 -0.041079245
pseudo_1034 0.183794943 -0.06322385 0.453741638
0.285399248
pseudo_1035 0.232196253 -1.688049717
0.727091175 -0.224381177
pseudo_1036 0.94920688 -0.001024207
0.333801689 -0.474647937
pseudo_1037 0.784715264 -0.096757954

0.697957584 -0.151541569
pseudo_1038 0.03829262 -3.571124965
0.14181315 -0.894102163
pseudo_1039 0.478120551 -0.185454817 0.870542272
0.425419732
pseudo_104 0.942984634 0.792433754
0.08374558 -0.673044698
pseudo_1040 0.197294629 -6.462182673 0.358197402
0.116977232
pseudo_1041 0.934312522 0.361966668
0.383204885 -0.401506552
pseudo_1042 0.73826897 -0.834370517 0.515235996
0.477416326
pseudo_1043 0.22494651 -0.458033102 0.496740833
0.22543059
pseudo_1044 0.289017125 -0.840108876 0.711889308
0.02500398
pseudo_1045 0.359425449 -2.771489024
0.550658261 -0.276480712
pseudo_1046 0.606214449 -0.87976046
0.565366772 -0.346443809
pseudo_1047 0.235573898 0.788452555 0.957300376
0.177656331
pseudo_1048 0.686999765 -0.258739097
0.265523348 -0.64489424
pseudo_1049 0.261850126 -0.061005346
0.307160271 -0.139537105
pseudo_105 0.807030097 -1.023176167
0.328744485 -0.207206996
pseudo_1050 0.249753115 0.753498935 0.807030096
0.35841118
pseudo_1051 0.378585534 -0.130898632
0.863777444 -0.280118687
pseudo_1052 0.066177448 1.050876493
0.319539324 -1.167654868
pseudo_1053 0.370387763 0.279434053 0.37900892
0.128167619
pseudo_1054 0.82950311 -0.092422902 0.793138995
0.226209244
pseudo_1055 0.837433284 -0.069084513
0.341679443 -0.289069312
pseudo_1056 0.745357419 0.295484859
0.410698571 -0.16010999
pseudo_1057 0.140967169 -0.783115352 0.206127567
0.072926169
pseudo_1058 0.620356054 -0.250127223 0.386360216
0.251486676
pseudo_1059 0.997817228 -0.851027021 0.486892798
0.374006268
pseudo_106 0.203336951 -1.416457465

0.500709572 -0.238387613
pseudo_1060 0.220353254 0.407754577 0.448291496
0.105992409
pseudo_1061 0.093851354 0.79489483 0.354935549
0.20161984
pseudo_1062 0.432825926 0.544066056 0.401424905
0.25981409
pseudo_1063 0.89150956 0.038624212 0.114630204
0.896711189
pseudo_1064 0.547010779 0.391604993 0.318400651
0.295898714
pseudo_1065 0.274003091 0.411652794
0.096329152 -0.24906704
pseudo_1066 0.717138907 0.637199337
0.941118621 -0.282968263
pseudo_1067 0.140967169 1.078495 0.459888433 0.287543274
pseudo_1068 0.269231908 -0.533590881 0.038585315
0.971863075
pseudo_1069 0.947339864 -0.272674326
0.044023385 -0.457078889
pseudo_107 0.706653336 0.153021435
0.843544721 -0.80594765
pseudo_1070 0.292586743 0.150621079 0.106767927
0.317300353
pseudo_1071 0.535625696 0.89153719 0.697957584
0.436280776
pseudo_1072 0.09446596 1.63663913
0.695645342 -0.373265777
pseudo_1073 0.569073185 -0.33361298
0.351692507 -0.634058652
pseudo_1074 0.296547379 1.147330496
0.749502369 -0.236596871
pseudo_1075 0.983474456 -0.09718155 0.312934579
0.045341965
pseudo_1076 0.446707126 1.485196608 0.520295375
0.877393215
pseudo_1077 0.494268914 -0.284317776 0.854568842
0.540974672
pseudo_1078 0.604577263 0.883770644
0.064127963 -0.260468836
pseudo_1079 0.68642483 0.568665049 0.151824273
0.440864386
pseudo_108 0.278138402 -5.863169791
0.032441437 -0.089962153
pseudo_1080 0.445773903 0.407630284
0.725917889 -0.362202029
pseudo_1081 0.301639834 1.54153139
0.982227445 -0.090463437
pseudo_1082 0.677250753 -0.210877577 0.445307706
0.334315331

pseudo_1083 0.938009322 0.26916437
0.71830731 -0.154794128
pseudo_1084 0.903260667 1.153298078
0.452800108 -0.645220922
pseudo_1085 0.097904176 -1.259732151 0.269909991
0.20570975
pseudo_1086 0.529978261 0.994019258 0.57279116
0.343721982
pseudo_1087 0.697379261 -0.143691903
0.877932887 -0.342089395
pseudo_1088 0.478120551 0.483708467 0.593713776
0.325261304
pseudo_1089 0.055751338 1.55884066 0.549042638
0.4031178
pseudo_109 0.638806305 0.611216135
0.771529128 -0.030368289
pseudo_1090 0.171236272 1.468508459 0.952319189
0.192599806
pseudo_1091 0.273356624 -0.714983689 0.261272655
0.931707474
pseudo_1092 0.415158967 -1.227715104 0.018076956
0.691905312
pseudo_1093 0.560092111 -1.075380722 0.861319953
0.266190241
pseudo_1094 0.411588368 0.818309267 0.515235996
0.479279136
pseudo_1095 0.845380008 0.874102228 0.3334109
0.261090107
pseudo_1096 0.529978261 -2.456760064
0.256242662 -0.596700859
pseudo_1097 0.928687285 -0.131185608
0.006659136 -1.161063116
pseudo_1098 0.957923146 -0.11745524 0.972253169
0.451520528
pseudo_1099 0.470876848 -1.039803129 0.646646683
0.034992594
pseudo_11 0.473767039 0.284691391
0.861319953 -0.041329054
pseudo_110 0.168320097 -2.109301753 0.8404878
0.418856872
pseudo_1100 0.922477946 1.252038533 0.170503704
0.771393859
pseudo_1101 0.94174059 -0.397847452 0.368095652
0.428273657
pseudo_1102 0.454212819 0.252604235
0.135153522 -0.72909912
pseudo_1103 0.763767965 -0.756897818
0.309387063 -0.283725203
pseudo_1104 0.504695059 0.459863623 0.347665187
0.761241061

pseudo_1105	0.268216976	2.088846673	0.358197404	0.127407292
pseudo_1106	0.548051696	-1.964062142	0.587238868	0.300799478
pseudo_1107	0.608400443	0.41021866	0.034790355	0.546287643
pseudo_1108	0.059337974	-1.173543566	0.226443376	0.096624933
pseudo_1109	0.668124304	-1.126226019	0.155884985	0.215187979
pseudo_111	0.94485096	-0.616646546	0.350078046	0.70091782
pseudo_1110	0.264183548	-1.379129155	0.593172954	-0.161627482
pseudo_1111	0.767347144	0.565503532	0.185606238	-0.170704936
pseudo_1112	0.487382683	0.105113799	0.930550951	0.473328398
pseudo_1113	0.484935928	-0.336819621	0.794344384	-0.225195145
pseudo_1114	0.33576006	-0.214670868	0.518268555	-0.291959168
pseudo_1115	0.365915709	-0.59500704	0.000198219	0.686824672
pseudo_1116	0.751281003	1.938797287	0.30310545	-0.704963462
pseudo_1117	0.739448853	0.139587729	0.832551167	0.228776881
pseudo_1118	0.012417874	2.231799654	0.828284601	-0.313203097
pseudo_1119	0.97225317	-0.005528869	0.873620359	0.115113014
pseudo_112	0.755383603	0.435157633	0.37827851	-0.359905326
pseudo_1120	0.304944121	-0.124481636	0.815515749	-0.03686034
pseudo_1121	0.218188718	-1.890662672	0.123053386	0.443420257
pseudo_1122	0.051764786	-1.589288144	0.595879316	0.114656551
pseudo_1123	0.395312888	-0.502476804	0.453741638	-0.732730038
pseudo_1124	0.11698439	0.671683429	0.196210288	0.539523097
pseudo_1125	0.779313227	0.42030091	0.960414478	0.234834394
pseudo_1126	0.73443862	-0.498084158	0.449513455	0.029021686
pseudo_1127	0.803400267	-0.826369477	0.456100288	0.052514907

pseudo_1128	0.148502301	-1.982297647	0.042327852	-0.630923647
pseudo_1129	0.623246768	0.38297392	0.274689375	-0.160159236
pseudo_113	0.214089058	-1.279423733	0.162835209	0.731443851
pseudo_1130	0.408036055	-1.707884072	0.564838234	-0.228275169
pseudo_1131	0.568013027	-0.929216427	0.032000835	-0.219295924
pseudo_1132	0.038146982	1.818407757	0.89027394	0.334640208
pseudo_1133	0.030770133	1.637700388	0.126588378	0.630636018
pseudo_1134	0.631006092	-0.571556506	0.882865921	-0.18507047
pseudo_1135	0.078567845	0.595373177	0.83499143	-0.3469608
pseudo_1136	0.138036759	0.786047427	0.477151216	-0.195549845
pseudo_1137	0.622141673	-1.888556191	0.665282241	0.009075025
pseudo_1138	0.666418493	0.34620764	0.514983699	0.122452146
pseudo_1139	0.808846577	-0.761796788	0.230368454	-0.357796758
pseudo_114	0.799774672	0.067485773	0.013528008	-0.86163813
pseudo_1140	0.527421284	0.706351959	0.728852304	0.12906335
pseudo_1141	0.1772664	0.773334626	0.713790453	-0.28881644
pseudo_1142	0.690453162	0.693825982	0.113022338	-0.300712628
pseudo_1143	0.312747205	1.000833031	0.884099898	0.134202129
pseudo_1144	0.783513905	0.208388425	0.489344875	0.207352555
pseudo_1145	0.431909475	-2.7196649	0.396182572	-0.732235559
pseudo_1146	0.256570188	0.030163668	0.132116215	0.016957538
pseudo_1147	0.686999765	-0.902250389	0.700272633	-0.280230267
pseudo_1148	0.379856571	0.077126756	0.002010963	-1.030806893
pseudo_1149	0.88224904	0.545978341	0.08249496	-0.898825404
pseudo_115	0.152495409	-0.829478188	0.497236007	0.489241393

pseudo_1150	0.411699908	-0.789838259	0.109647988	0.678404794
pseudo_1151	0.999688175	-0.312913691	0.109512947	-0.367160017
pseudo_1152	0.804609743	0.125627594	0.500212562	0.402043415
pseudo_1153	0.501758684	-0.269783003	0.873612848	-0.272802447
pseudo_1154	0.735911056	-0.870300464	0.299086371	0.213113894
pseudo_1155	0.426434428	-1.489698546	0.552747897	-0.303442295
pseudo_1156	0.009931521	2.145602976	0.263849325	-0.307943234
pseudo_1157	0.996054305	0.424715913	0.970413948	-0.238706649
pseudo_1158	0.935527706	0.152784068	0.567882762	-0.274739243
pseudo_1159	0.729439666	0.12834947	0.456572844	0.32010057
pseudo_116	0.991580881	-0.174708253	0.693335926	0.396304853
pseudo_1160	0.0187356	-3.537524944	0.368095652	0.505714437
pseudo_1161	0.628230174	1.218368429	0.64216159	0.355389129
pseudo_1162	0.836822673	0.084929125	0.050461914	0.139591213
pseudo_1163	0.104077723	-0.62552817	0.868696358	-0.17388539
pseudo_1164	0.11789989	0.41030406	0.640483028	-0.146167417
pseudo_1165	0.306050867	-1.190203623	0.233420486	0.337794358
pseudo_1166	0.450920363	1.07402665	0.770333592	-0.225610433
pseudo_1167	0.94858451	0.072210679	0.133932187	0.578907198
pseudo_1168	0.343468203	-0.360993254	0.940496688	0.305088913
pseudo_1169	0.891174941	-1.115792812	0.14827499	-0.153925184
pseudo_117	0.231281072	0.484843448	0.797359998	0.15577309
pseudo_1170	0.115893168	-4.258865393	0.684126909	0.350420404
pseudo_1171	0.106937879	0.519122405	0.449982145	-0.534515364
pseudo_1172	0.075177604	-0.915829565	0.421451374	0.524152394

pseudo_1173	0.716251305	0.399188432	
0.9861907	-0.266135983		
pseudo_1174	0.85824998	0.014030843	
0.365191224	-0.43491477		
pseudo_1175	0.475215783	0.508613006	
0.372269702	-0.244794077		
pseudo_1176	0.300178942	0.35914785	
0.796756631	-0.300881077		
pseudo_1177	0.379856571	-0.729548516	
0.681831911	-0.248782012		
pseudo_1178	0.82950311	0.598196676	
0.049186633	-0.719196116		
pseudo_1179	0.356564128	1.601256965	0.812482619
0.119547363			
pseudo_118	0.36026254	0.218332579	0.016651728
0.749329226			
pseudo_1180	0.32913173	-0.495410839	0.510202285
0.056501846			
pseudo_1181	0.279906383	1.76990698	
0.212651328	-0.348777771		
pseudo_1182	0.67553588	0.52483672	
0.747725068	-0.058137266		
pseudo_1183	0.042566637	-1.683985779	
0.916893567	-0.138205023		
pseudo_1184	0.671541066	-0.55288171	0.921857266
0.31464039			
pseudo_1185	0.636573542	1.225328018	0.436042352
0.735457296			
pseudo_1186	0.998440876	0.167875789	0.79133184
0.221647569			
pseudo_1187	0.11391339	1.270124848	
0.147407399	-0.707925594		
pseudo_1188	0.962283232	-0.398260554	
0.91441292	-0.220410626		
pseudo_1189	0.182509197	1.660403668	
0.938631108	-0.246029631		
pseudo_119	0.730027187	0.097429159	0.100953355
0.880414031			
pseudo_1190	0.190854061	-0.393132044	0.13332473
0.937207384			
pseudo_1191	0.267204675	0.713938601	
0.659612556	-0.109938072		
pseudo_1192	0.971006658	0.61665374	0.748317353
0.121470469			
pseudo_1193	0.747725069	-1.689841911	
0.751874174	-0.087706483		
pseudo_1194	0.476666955	0.135605427	0.594254823
0.288072631			
pseudo_1195	0.146971172	0.696619147	0.586700771
0.185374785			

pseudo_1196 0.994699014 -0.079887622 0.193786512
0.41783102
pseudo_1197 0.967267561 -0.102183034 0.284772468
0.677355166
pseudo_1198 0.839876704 1.00629443
0.043941365 -0.535625041
pseudo_1199 0.899547377 -0.244820693
0.333801689 -0.093776667
pseudo_12 0.800982728 -0.995575599
0.923098673 -0.095682483
pseudo_120 0.366849143 -0.349233481 0.045101467
0.756857023
pseudo_1200 0.902641636 0.564226537
0.389257455 -0.228054121
pseudo_1201 0.251041751 -0.91005886 0.734733028
0.743780228
pseudo_1202 0.669425237 1.063023271
0.939557019 -0.41483973
pseudo_1203 0.296547379 1.257212911
0.903260667 -0.137902339
pseudo_1204 0.107619827 -0.698002473 0.339303722
0.166073314
pseudo_1205 0.718307311 0.58145423 0.03940005
0.820837408
pseudo_1206 0.994699014 0.655513382
0.951696667 -0.276538329
pseudo_1207 0.624906002 -0.92205574 0.241489616
0.282174205
pseudo_1208 0.765556929 -0.204338144 0.790127692
0.742673735
pseudo_1209 0.329907103 -0.258560139
0.713054717 -0.285838858
pseudo_121 0.982903226 -0.330652584 0.815567787
0.261577
pseudo_1210 0.695356511 -0.333875406 0.308272337
0.06148811
pseudo_1211 0.853955625 0.928628029 0.244011471
0.336736654
pseudo_1212 0.682405387 0.736093057
0.88101549 -0.2627683
pseudo_1213 0.463731571 2.143526077
0.27589119 -0.752366688
pseudo_1214 0.084587825 0.697806226
0.866851159 -0.331970756
pseudo_1215 0.455628009 -1.077682914
0.495256891 -0.195048921
pseudo_1216 0.484935928 -0.582520699
0.233113999 -0.616599632
pseudo_1217 0.82950311 0.257267976
0.957300376 -0.089084931

pseudo_1218 0.953992226 1.235978933 0.597281488
0.0667449
pseudo_1219 0.451859684 1.534461198
0.012583231 -0.972833789
pseudo_122 0.246870601 -0.345861878 0.322588792
0.306671395
pseudo_1220 0.786518265 0.667626733 0.198110752
0.233712804
pseudo_1221 0.989086465 -0.726647913 0.544412774
0.535266371
pseudo_1222 0.027428333 1.737364738 0.901403752
0.022692823
pseudo_1223 0.092177711 -1.255591956 0.717138906
0.181044129
pseudo_1224 0.921868678 -0.121558152
0.383204883 -0.23231353
pseudo_1225 0.039984114 2.456967446
0.453984929 -0.299366457
pseudo_1226 0.333410903 0.883404566
0.609494753 -0.288215745
pseudo_1227 0.467996415 0.627671563 0.799774671
0.087239481
pseudo_1228 0.975993098 0.446628183 0.98971006
0.122574517
pseudo_1229 0.33969894 0.125105585 0.030891346
1.289624241
pseudo_123 0.045522 0.470128087 0.70259047 0.15733744
pseudo_1230 0.070681563 -1.258533121
0.66585027 -0.329510482
pseudo_1231 0.902641636 1.890733798
0.855182147 -0.215135347
pseudo_1232 0.196480966 1.160003607
0.102095629 -0.518625679
pseudo_1233 0.704330667 0.059769755 0.503697121
0.231742541
pseudo_1234 0.228248984 0.453553766
0.087880107 -0.555946137
pseudo_1235 0.315377203 -0.129004146 0.720646092
0.211815491
pseudo_1236 0.825544473 -0.576662083
0.548051694 -0.504971215
pseudo_1237 0.819159155 -0.269020388
0.830722027 -0.105446728
pseudo_1238 0.330683657 -0.177482915 0.111608092
0.847183383
pseudo_1239 0.391845699 -0.309293007
0.865621428 -0.11367316
pseudo_124 0.541303289 -0.623403396
0.384112272 -0.848156437
pseudo_1240 0.1086492 -1.312644308

0.682405386 -0.535565027
pseudo_1241 0.811876329 0.23419201 0.078170421
0.191497891
pseudo_1242 0.539751879 -0.265150757 0.980357009
0.080991152
pseudo_1243 0.737089703 -0.280728318 0.813695533
0.165275417
pseudo_1244 0.48885393 0.758168261
0.785917136 -0.143376223
pseudo_1245 0.11826766 1.780798475 0.796153386
0.192057049
pseudo_1246 0.089196202 0.795478877 0.659046655
0.294976482
pseudo_1247 0.154975559 -2.074035929 0.283717927
1.310372625
pseudo_1248 0.526400258 -1.24285494 0.556414095
0.381462268
pseudo_1249 0.108477099 1.33256638
0.679539852 -0.395134863
pseudo_125 0.043941366 0.693027875 0.93303643
0.085098872
pseudo_1250 0.034992496 -1.436082532 0.38155536
0.612931454
pseudo_1251 0.943606708 0.416361162 0.93303643
0.045914264
pseudo_1252 0.507194453 2.929423894
0.563254042 -0.22067314
pseudo_1253 0.020067915 -1.905157653 0.387264644
0.409595083
pseudo_1254 0.450451118 -0.855573481
0.634344074 -0.56264656
pseudo_1255 0.611686 0.726814553 0.526910643 -0.25121641
pseudo_1256 0.01280674 -2.081874498 0.903260667
0.114844637
pseudo_1257 0.668124304 -0.160252271 0.383258805
0.529114657
pseudo_1258 0.859477715 0.528676646
0.986592156 -0.364272467
pseudo_1259 0.252983393 0.64928421
0.175432047 -0.711688881
pseudo_126 0.911933107 -0.187684113
0.957923146 -0.152724345
pseudo_1260 0.845380009 0.523359203 0.29799646
0.405247452
pseudo_1261 0.745949107 0.479695362
0.103082887 -0.424354858
pseudo_1262 0.734733029 0.146380359
0.439735088 -0.64342672
pseudo_1263 0.194591985 0.273023467
0.009999124 -1.004634435

pseudo_1264 0.069833468 -0.260037615
0.849053091 -0.481791266
pseudo_1265 0.638806305 0.30359936
0.225843778 -0.108237462
pseudo_1266 0.407593303 0.364157483
0.819159154 -0.280076773
pseudo_1267 0.966021352 0.034576281 0.754842226
0.250001102
pseudo_1268 0.752467494 -0.077721222
0.800982727 -0.779204401
pseudo_1269 0.557463755 -2.629738246
0.745949106 -0.207752493
pseudo_127 0.465125769 0.537152065 0.260523153
0.229432918
pseudo_1270 0.669262465 0.032856606
0.176680578 -0.390945492
pseudo_1271 0.796153387 0.524796493 0.112844789
0.440170747
pseudo_1272 0.734733029 0.836711226
0.14653593 -0.127083975
pseudo_1273 0.504195961 0.08969869 0.954809562
0.126606932
pseudo_1274 0.286561982 0.41925502
0.548493221 -0.138260788
pseudo_1275 0.443910792 -2.989498303
0.587777193 -0.1977515
pseudo_1276 0.990957268 0.556288524
0.844768152 -0.184229664
pseudo_1277 0.828284602 0.308317872 0.693335926
0.247890705
pseudo_1278 0.071292503 1.948945994 0.376472964
0.242441278
pseudo_1279 0.786518265 -1.571873647
0.440197939 -0.555046956
pseudo_128 0.38283251 0.5383562 0.401424905
0.082475646
pseudo_1280 0.588315748 0.866451395
0.027814284 -0.413691449
pseudo_1281 0.722401898 -2.39351186
0.145668409 -0.451467285
pseudo_1282 0.359829964 0.40139367 0.814288092
0.21046988
pseudo_1283 0.576520627 0.802205369 0.796153386
0.199150825
pseudo_1284 0.432825926 -0.744643831 0.440197939
0.007497241
pseudo_1285 0.468955473 -1.091230794 0.136795332
0.412498639
pseudo_1286 0.715095792 2.180177888 0.838043992
0.268536985

pseudo_1287 0.89027394 0.30874945
0.042566636 -0.758419329
pseudo_1288 0.624352712 0.309203142 0.674108102
0.01126751
pseudo_1289 0.359425449 1.958233434
0.460363186 -0.225160723
pseudo_129 0.670025678 0.069827666
0.904218899 -0.096408195
pseudo_1290 0.708397134 0.687941936
0.719476373 -0.837476412
pseudo_1291 0.709560516 -1.051876324
0.939252931 -0.148632287
pseudo_1292 0.903260667 0.74444659 0.42962327
0.357075117
pseudo_1293 0.510704498 1.805339559
0.981603955 -0.231757985
pseudo_1294 0.674964629 -0.395001458
0.639923916 -0.047069956
pseudo_1295 0.252659062 0.480444916 0.233727259
0.528122187
pseudo_1296 0.610042239 0.129487599 0.245914961
0.430809403
pseudo_1297 0.766153529 0.091088985
0.962906197 -0.315673079
pseudo_1298 0.968513851 -0.192040077
0.685275505 -0.085995197
pseudo_1299 0.308643619 0.428334817 0.501206844
0.615492494
pseudo_13 0.133729466 0.997710982
0.068162331 -0.523977442
pseudo_130 0.131115618 1.827059781 0.489344875
0.114034577
pseudo_1300 0.659612557 -0.248215551
0.126200529 -0.115140112
pseudo_1301 0.754842227 -0.420064089 0.664146758
0.641305067
pseudo_1302 0.487382683 0.680666305 0.157945997
0.244049444
pseudo_1303 0.22494651 1.791333856
0.313497153 -0.72745191
pseudo_1304 0.395312888 0.137546388
0.129922667 -0.460159252
pseudo_1305 0.45610029 -0.757088345
0.808240968 -0.708904682
pseudo_1306 0.494268914 0.511754359 0.185346677
0.169287182
pseudo_1307 0.334474045 -0.994501913
0.519768519 -0.279767646
pseudo_1308 0.294562598 0.163127443 0.869003961
0.183701349

pseudo_1309 0.344863589 −0.064461351
0.742105827 −0.13865962
pseudo_131 0.145236127 1.131659087 0.259530983
0.079106747
pseudo_1310 0.489344877 0.535495933 0.052047604
1.027485562
pseudo_1311 0.297996462 −0.982564603 0.882865921
0.121403257
pseudo_1312 0.97225317 −0.974860203
0.249110531 −0.347173976
pseudo_1313 0.132116217 0.690510219
0.345262932 −0.634535919
pseudo_1314 0.622141673 −0.569105627 0.63100609
0.237398648
pseudo_1315 0.610589941 0.900552724 0.289728689
0.247788654
pseudo_1316 0.185346678 −0.529456634
0.145236125 −0.177138142
pseudo_1317 0.481521677 0.802643242
0.223159613 −0.196957777
pseudo_1318 0.581868215 0.302066069 0.934279415
0.224930842
pseudo_1319 0.542338799 −1.509011438
0.399235558 −0.323850672
pseudo_132 0.232501884 1.236684383
0.192449503 −0.490136674
pseudo_1320 0.870542273 −0.246452537
0.003997611 −0.755912239
pseudo_1321 0.212364623 0.988264798
0.030110733 −0.471700319
pseudo_1322 0.827675502 −1.258143715
0.578122467 −0.37995216
pseudo_1323 0.351673069 −2.008949765 0.094789098
0.453667883
pseudo_1324 0.449982149 1.638267765
0.727091175 −0.355426987
pseudo_1325 0.980980476 −2.568593499
0.854568842 −0.258970123
pseudo_1326 0.276410222 0.364017952 0.688725651
0.266035544
pseudo_1327 0.446707126 0.215319922 0.535625695
0.263059352
pseudo_1328 0.966644446 −0.423567147 0.379856569
0.059649996
pseudo_1329 0.354123026 0.42054034
0.71830731 −0.267297331
pseudo_133 0.163306739 1.72851734
0.060186099 −0.978248786
pseudo_1330 0.080851065 −1.940924797
0.746540944 −0.169859495

pseudo_1331 0.413594582 0.564225346 0.083885481
0.764712017
pseudo_1332 0.230672377 -2.634431949
0.165203147 -0.233521903
pseudo_1333 0.550136461 -0.907809139
0.635458394 -0.158725625
pseudo_1334 0.25007484 0.128648056 0.213513126
0.565146185
pseudo_1335 0.695067724 -3.104122052 0.564838234
0.13522844
pseudo_1336 0.982227445 0.122401633
0.350078046 -0.318101107
pseudo_1337 0.748909788 0.761916779
0.073905886 -0.549223674
pseudo_1338 0.88661632 -0.943631997
0.110197797 -0.65291141
pseudo_1339 0.437425031 -0.42678813
0.662444978 -0.158600353
pseudo_134 0.16401596 -1.891148828 0.683552885
0.398070026
pseudo_1340 0.449045042 1.525701715
0.822806468 -0.029951826
pseudo_1341 0.619382662 -0.137844399 0.806424833
0.208885582
pseudo_1342 0.967267561 -1.232243332 0.359835381
0.7072796
pseudo_1343 0.029638831 1.101521595
0.861319953 -0.24645326
pseudo_1344 0.430994149 -1.093850611 0.827675501
0.174338356
pseudo_1345 0.759772781 0.421773786
0.356381103 -0.30119054
pseudo_1346 0.901403752 0.43044791
0.014788756 -0.6596321
pseudo_1347 0.799608033 0.872801169 0.393174839
0.154066078
pseudo_1348 0.853955625 -1.021867243 0.811876329
0.106074464
pseudo_1349 0.049912013 1.311489714 0.035742275
0.595457061
pseudo_135 0.033015553 2.271850174 0.090230767
0.643935681
pseudo_1350 0.295103018 -1.095139154
0.89027394 -0.328876902
pseudo_1351 0.06652422 1.664166807 0.472802558
0.161256351
pseudo_1352 0.611137862 -0.485166927
0.390550273 -0.245422884
pseudo_1353 0.266867825 -0.626904225 0.079501557
0.263426338

pseudo_1354	0.072031337	1.472601715	0.331461391	0.263212902
pseudo_1355	0.24943168	0.808568335	0.929929686	-0.204931542
pseudo_1356	0.061803427	0.720761846	0.464171065	-0.52032689
pseudo_1357	0.068407651	-1.619028923	0.996054305	-0.457584902
pseudo_1358	0.404228537	0.606484238	0.8091743	0.340787575
pseudo_1359	0.07659707	1.126577087	0.832551167	0.10183466
pseudo_136	0.055145552	1.764646749	0.090528124	0.640263699
pseudo_1360	0.606214449	0.48986221	0.841098993	0.169039565
pseudo_1361	0.340886358	-0.470262556	0.432367558	-0.912200752
pseudo_1362	0.792611745	0.446525431	0.574605308	-0.004612494
pseudo_1363	0.078700683	-1.132987491	0.19405473	0.686715664
pseudo_1364	0.283016373	1.456591086	0.210365489	0.267185641
pseudo_1365	0.011289787	1.557301501	0.723033786	-0.223966717
pseudo_1366	0.620485626	0.949569654	0.386036818	0.171686672
pseudo_1367	0.445828797	1.535873226	0.969756791	0.253716489
pseudo_1368	0.512715923	-1.857182231	0.983474456	-0.268746014
pseudo_1369	0.550658262	0.844595902	0.408036053	-0.030920538
pseudo_137	0.651144652	-1.006892744	0.614979387	-0.242929112
pseudo_1370	0.654526493	-0.94903842	0.053577883	-1.019990254
pseudo_1371	0.71686698	-0.182454562	0.670025676	0.244582801
pseudo_1372	0.805819688	-2.271268512	0.503198543	-0.528814889
pseudo_1373	0.318021686	-1.088555428	0.037140639	0.716144067
pseudo_1374	0.768541312	-1.145809507	0.895217987	0.071384816
pseudo_1375	0.569073185	-0.647124112	0.364364023	0.207537959
pseudo_1376	0.336545475	-0.69634423	0.125042533	0.534128367

pseudo_1377 0.838654799 0.040780828
0.346062505 -0.458136989
pseudo_1378 0.181740957 -0.562992764 0.043047623
0.703562702
pseudo_1379 0.082219112 -1.633938455
0.28512457 -0.088391143
pseudo_138 0.275721004 -0.208139447
0.244327999 -0.484175138
pseudo_1380 0.475215783 0.386719317
0.904498905 -0.190111931
pseudo_1381 0.960558962 -0.653564817 0.516033542
0.587700473
pseudo_1382 0.47763575 0.054955229 0.647769973
0.213774121
pseudo_1383 0.254609401 -1.752077825 0.229761468
0.230179506
pseudo_1384 0.240862031 -0.45255368
0.298359469 -0.280204488
pseudo_1385 0.94858451 -0.503200448
0.30310545 -0.703869201
pseudo_1386 0.383685395 0.466702264
0.819159154 -0.097341963
pseudo_1387 0.033535028 -1.077787729
0.940868561 -0.40879196
pseudo_1388 0.977239862 0.044181091
0.731790697 -0.319120894
pseudo_1389 0.239610306 2.051328353 0.842321667
0.348700044
pseudo_139 0.246870601 1.795732172 0.556414095
0.134443493
pseudo_1390 0.482983324 -1.603853276
0.868696358 -0.333046035
pseudo_1391 0.711889311 -2.847967511 0.019132765
0.928820925
pseudo_1392 0.633787225 -0.235597485 0.095393913
0.864864631
pseudo_1393 0.330295232 0.721753157
0.3150006 -0.55986668
pseudo_1394 0.86009171 -0.367380871 0.256242662
0.271843307
pseudo_1395 0.745357419 -0.260436155
0.981603955 -0.595768111
pseudo_1396 0.951696668 1.604103399 0.624906 0.395841189
pseudo_1397 0.409809923 -0.457014052
0.452329757 -0.365764592
pseudo_1398 0.064918671 -0.384305598
0.757813902 -0.234780729
pseudo_1399 0.502202173 0.21376238
0.326042059 -0.278903483
pseudo_14 0.288661784 0.095819034

0.072651811 -0.271294934
pseudo_140 0.816729779 -0.312902279
0.366434222 -0.186996807
pseudo_1400 0.319919475 1.222586801 0.015275242
0.819823376
pseudo_1401 0.376894897 0.686004688 0.464171065
0.221314471
pseudo_1402 0.681258621 0.372464782 0.838043992
0.240368662
pseudo_1403 0.038695566 -5.284674288
0.350078046 -0.275071971
pseudo_1404 0.042248511 1.363393378 0.80037864
0.186168353
pseudo_1405 0.682979045 -1.207494724
0.900166107 -0.227539571
pseudo_1406 0.337725811 0.385082889 0.926203007
0.275172608
pseudo_1407 0.669262465 -1.97049545
0.486892798 -0.288230355
pseudo_1408 0.356972007 -0.997325652 0.44437615
0.81584367
pseudo_1409 0.688150176 -0.106269525 0.371432551
0.034298125
pseudo_141 0.467038444 0.631216082 0.508697203
0.140951789
pseudo_1410 0.598591295 0.455352136 0.673252 -0.16939537
pseudo_1411 0.553532514 -0.465546765
0.230064819 -0.408092166
pseudo_1412 0.150488758 0.778206685 0.984097978
0.238176532
pseudo_1413 0.401863639 0.886734118
0.093545254 -0.44372757
pseudo_1414 0.995322652 0.415033504
0.957300376 -0.22184545
pseudo_1415 0.861934201 0.74297616
0.844156389 -0.220030638
pseudo_1416 0.866236254 0.9057722
0.105752713 -0.504795485
pseudo_1417 0.176931079 -1.533131964 0.864392023
0.280776742
pseudo_1418 0.906356697 0.551678108
0.01929368 -0.047116479
pseudo_1419 0.597505832 0.491126118
0.730614865 -0.116553489
pseudo_142 0.070803406 1.458629561
0.593172954 -0.230373379
pseudo_1420 0.280918761 1.201964312
0.11771634 -0.729048831
pseudo_1421 0.721816467 -2.040568709
0.27297586 -0.628679755

pseudo_1422	0.599134362	-0.50070498	0.047850383	0.309779827
pseudo_1423	0.509449454	-0.598343851	0.411588366	0.39368539
pseudo_1424	0.939874791	-1.721265078	0.61058994	0.178555445
pseudo_1425	0.400767346	0.636708969	0.939252931	-0.012180467
pseudo_1426	0.064353065	-1.478551363	0.48006244	0.319602237
pseudo_1427	0.515740784	1.598157903	0.321824651	0.117730816
pseudo_1428	0.060936432	2.41434026	0.868388774	-0.264483449
pseudo_1429	0.75721928	-0.326943334	0.736500301	-0.422946266
pseudo_143	0.361889458	0.945199802	0.905737375	0.069812668
pseudo_1430	0.810664088	-0.76434028	0.657350122	0.310205685
pseudo_1431	0.648331921	0.378383115	0.995322652	-0.18249194
pseudo_1432	0.802191261	0.554673375	0.195669763	-0.522959119
pseudo_1433	0.236810712	-0.528935556	0.416054466	0.174781628
pseudo_1434	0.059232659	2.839443168	0.202294555	-0.473508117
pseudo_1435	0.942362594	-0.37710608	0.693624448	0.184582141
pseudo_1436	0.796153387	0.250749776	0.972253169	0.175853413
pseudo_1437	0.851503641	0.954420851	0.95294174	0.235794246
pseudo_1438	0.78711952	0.064631374	0.913792887	-0.145649568
pseudo_1439	0.331461393	1.374193791	0.322206574	-0.163635068
pseudo_144	0.312372674	0.877431399	0.871157734	-0.401574879
pseudo_1440	0.066553846	-3.372256132	0.014598017	1.292007308
pseudo_1441	0.89769155	-0.236336815	0.39271076	-0.299778256
pseudo_1442	0.963529184	-0.203281264	0.70607241	0.281132234
pseudo_1443	0.905737376	0.174198619	0.817944248	0.22977644
pseudo_1444	0.372688717	2.346856932	0.175432047	-0.327331356

pseudo_1445 0.138452491 1.133291221 0.455156002
0.243199875
pseudo_1446 0.744174494 −0.067679956 0.467517292
0.237125792
pseudo_1447 0.807030097 0.697130405 0.460363186
0.236557429
pseudo_1448 0.6056685 0.269561687 0.870542272
0.028645435
pseudo_1449 0.80219126 1.321762248
0.228248981 −0.14338027
pseudo_145 0.27195127 −0.64807593 0.029815043
0.803668304
pseudo_1450 0.346462736 −2.878756461 0.32758453
0.152406258
pseudo_1451 0.059654852 1.941373738
0.072155081 −0.403948126
pseudo_1452 0.693335928 1.667093174
0.572921146 −0.36446483
pseudo_1453 0.329907103 0.619058276
0.4129252 −0.123179253
pseudo_1454 0.531515465 0.583712656 0.563781869
0.228443319
pseudo_1455 0.621037428 −0.47961586
0.015674521 −0.384970886
pseudo_1456 0.938009322 −0.170108667
0.080174003 −0.406639466
pseudo_1457 0.140756281 −6.049218823
0.063345225 −0.408372359
pseudo_1458 0.921857266 0.172381238
0.666986904 −0.321322901
pseudo_1459 0.957923146 0.651446994
0.060506727 −0.656999298
pseudo_146 0.525890123 −3.086592237 0.250074838
0.25098299
pseudo_1460 0.406708661 −1.884974748 0.360656131
0.223327217
pseudo_1461 0.931793607 −1.222966947 0.494268913
0.686889584
pseudo_1462 0.959168762 −0.290994625
0.406708659 −0.524801221
pseudo_1463 0.722401898 1.285332173 0.599134358
0.152730587
pseudo_1464 0.693913016 0.406386859 0.203614767
0.737992957
pseudo_1465 0.810664088 0.411783352 0.091424929
0.694141486
pseudo_1466 0.893781493 −0.187814054
0.771724947 −0.265698697
pseudo_1467 0.986592156 −1.489207749 0.999064525
0.125794118

pseudo_1468	0.854819214	0.325209298	0.661041403	0.487030375
pseudo_1469	0.1411783	0.904291945	0.939874791	-0.000372205
pseudo_147	0.56589555	-1.422351014	0.623246767	-0.38895148
pseudo_1470	0.956054916	-0.484763772	0.244327999	0.601208359
pseudo_1471	0.102423877	1.937834823	0.795550263	0.294676137
pseudo_1472	0.871773275	-0.388286938	0.212364621	0.581109444
pseudo_1473	0.382406503	0.873409127	0.388397024	-0.303277777
pseudo_1474	0.336545475	0.538981423	0.559565958	0.348471439
pseudo_1475	0.246551766	-4.683419317	0.875468133	0.005312551
pseudo_1476	0.541303289	0.588382342	0.436042352	0.791727401
pseudo_1477	0.923098674	-0.872124608	0.568542986	0.274749167
pseudo_1478	0.074794226	-0.412550515	0.881632229	-0.187984114
pseudo_1479	0.210081016	0.29211714	0.490819307	0.154968921
pseudo_148	0.720061152	1.269580897	0.304575794	0.515826893
pseudo_1480	0.764364146	1.114306419	0.246233218	-0.326721733
pseudo_1481	0.246870601	-2.957728568	0.71014246	-0.767163307
pseudo_1482	0.64048303	-0.876715672	0.519281454	0.241469698
pseudo_1483	0.940496688	-2.951908295	0.442980904	0.356540601
pseudo_1484	0.047411803	-1.658718471	0.674964628	-0.154992029
pseudo_1485	0.305312741	-1.311420592	0.22855091	0.370537552
pseudo_1486	0.997193581	0.091108113	0.975993098	0.456263072
pseudo_1487	0.436963858	0.90032154	0.135767408	-0.564051705
pseudo_1488	0.481034998	1.374457945	0.571727705	-0.174405859
pseudo_1489	0.607616823	0.869835428	0.516033542	0.278952694
pseudo_149	0.576520627	-1.825907015	0.801586934	0.228970726

pseudo_1490 0.474249683 1.090876004 0.925582049
0.318641969
pseudo_1491 0.974746393 -2.613507082
0.148722023 -0.625987409
pseudo_1492 0.273660389 0.259173853
0.688725651 -0.215381136
pseudo_1493 0.887186063 -2.279327417
0.705491657 -0.352830042
pseudo_1494 0.443910792 1.446499226
0.292945324 -0.345542335
pseudo_1495 0.632117921 -0.486356614
0.319159471 -0.10211708
pseudo_1496 0.898928706 -0.914498162 0.541303288
0.170999878
pseudo_1497 0.610589941 -1.044968158
0.859477714 -0.644168547
pseudo_1498 0.49921933 0.532249187 0.289017123
0.469044056
pseudo_1499 0.501758684 -0.57077295 0.28992954
0.139443775
pseudo_15 0.013528009 1.816837827
0.229458399 -0.427990464
pseudo_150 0.135562542 0.584139917
0.057158725 -0.298499708
pseudo_1500 0.713637674 -0.426072649
0.445773901 -0.181491605
pseudo_1501 0.658057385 0.523599533 0.505439391
0.131686039
pseudo_1502 0.050094752 -2.280908486 0.036786752
0.856969015
pseudo_1503 0.003776537 2.535506559 0.385421079
0.006727272
pseudo_1504 0.242748243 1.241406537 0.270589245
0.120233489
pseudo_1505 0.099660393 -0.37935083
0.31726464 -0.062236587
pseudo_1506 0.583477 -0.357608574
0.010204399 -0.159865125
pseudo_1507 0.781712834 -0.621273875
0.578656878 -0.415311657
pseudo_1508 0.506193916 1.286317671
0.388397024 -0.18009105
pseudo_1509 0.15071072 -0.290710941
0.549093589 -0.247402619
pseudo_151 0.793138996 0.973208649 0.545451239
0.137650308
pseudo_1510 0.212938317 0.265863392 0.075049632
0.188762174
pseudo_1511 0.975369738 0.304395675
0.425525869 -0.439416933

pseudo_1512	0.117075687	0.364977525	0.752467493	-0.293129116
pseudo_1513	0.392710762	0.219888165	0.453741638	0.125103237
pseudo_1514	0.17026004	-1.432860736	0.091274968	0.731984097
pseudo_1515	0.006565479	-0.967518431	0.181230128	0.732225008
pseudo_1516	0.397925407	-0.36599039	0.596963435	0.215609774
pseudo_1517	0.411143328	-1.417087506	0.472320724	0.098285335
pseudo_1518	0.49921933	0.363993885	0.204728801	0.658698504
pseudo_1519	0.178691984	-0.558557979	0.759598631	-0.182129975
pseudo_152	0.703170363	1.734006835	0.888421011	0.163624176
pseudo_1520	0.398361837	0.67960428	0.902022664	-0.111687423
pseudo_1521	0.207533265	0.243926471	0.71014246	0.20038673
pseudo_1522	0.595879318	0.662621773	0.74476588	-0.073379975
pseudo_1523	0.480062442	1.151722364	0.348468296	-0.481296841
pseudo_1524	0.820982328	0.812795261	0.505194416	0.087833265
pseudo_1525	0.634344075	0.384429193	0.934279415	0.189900897
pseudo_1526	0.275032959	-1.863099124	0.778713652	-0.321596149
pseudo_1527	0.04543763	1.771971633	0.497731443	0.142389999
pseudo_1528	0.954809562	-0.32610385	0.7721271	-0.362121344
pseudo_1529	0.604577263	1.156704031	0.994081487	0.096124675
pseudo_153	0.836751338	0.478117945	0.784998445	0.379616635
pseudo_1530	0.676644368	-0.50504322	0.208786575	0.417373243
pseudo_1531	0.549093591	-2.755867685	0.69449028	0.012002778
pseudo_1532	0.666986905	0.677759816	0.051670799	0.520386006
pseudo_1533	0.652552885	-0.388389918	0.932414998	0.002883804
pseudo_1534	0.817944249	-0.340091089	0.939874791	-0.254171689

pseudo_1535 0.187954403 0.910899486 0.063123041
0.30380796
pseudo_1536 0.623799634 −0.217602324 0.396182572
0.075194864
pseudo_1537 0.498723106 −1.684543453
0.219322903 −0.542351754
pseudo_1538 0.584550673 0.015545386 0.821894277
0.108155482
pseudo_1539 0.040305463 1.386941266 0.698536083
0.129334614
pseudo_154 0.721231199 −0.207064692 0.977863265
0.233270239
pseudo_1540 0.624906002 0.162255266
0.462264937 −0.143337165
pseudo_1541 0.921236633 −2.75044549
0.487382682 −0.865406784
pseudo_1542 0.936144194 1.005200085
0.016360409 −0.876270265
pseudo_1543 0.929308464 0.266086262 0.484447374
0.124552306
pseudo_1544 0.4868928 0.15543692
0.448576902 −0.493409151
pseudo_1545 0.65339842 −2.165601819
0.855182147 −0.21420597
pseudo_1546 0.394546452 −0.709195097 0.290493379
0.026133401
pseudo_1547 0.572360281 0.520130863 0.760400441
0.277369354
pseudo_1548 0.113734739 1.412768196
0.414994227 −0.332937906
pseudo_1549 0.26797915 0.873654424
0.976329205 −0.223024229
pseudo_155 0.551243027 −2.293041958 0.572921146
0.020929377
pseudo_1550 0.214955053 2.014156355
0.708978739 −0.097001931
pseudo_1551 0.535111043 −0.336275217 0.540785903
0.136831277
pseudo_1552 0.698536085 −0.222651386 0.450920361
0.252105316
pseudo_1553 0.121242302 0.560824219 0.673252 0.185231348
pseudo_1554 0.009130608 1.662205876 0.465603527
0.343831896
pseudo_1555 0.293663377 −2.343753042 0.838043992
0.414157942
pseudo_1556 0.784715264 0.186495152 0.451389883
0.498248784
pseudo_1557 0.877316587 0.698864786 0.960414478
0.2715715
pseudo_1558 0.216345597 0.456531401 0.83932743

0.287398534
pseudo_1559	0.376051327	-0.658155843
0.59913436	-0.206647918
pseudo_156	0.036018299	2.531484916	0.059549069
0.392610064
pseudo_1560	0.060079538	-1.355559382
0.121806506	-0.580313723
pseudo_1561	0.978486682	0.032530902
0.863162946	-0.220681664
pseudo_1562	0.875468134	-1.574760458
0.288306736	-0.327068336
pseudo_1563	0.054553253	0.644144648	0.587238868
0.341257384
pseudo_1564	0.081944011	1.752318575	0.573323239
0.581524494
pseudo_1565	0.263849327	-4.508437038
0.889038586	-0.440532876
pseudo_1566	0.833771099	0.747167426
0.326042059	-0.494876583
pseudo_1567	0.944228817	0.192783622
0.844156389	-0.245739251
pseudo_1568	0.733555627	-0.825350624	0.919995511
0.23865154
pseudo_1569	0.656220082	0.317225952	0.015607343
0.349703538
pseudo_157	0.24496192	-1.148690187
0.802795704	-0.074113177
pseudo_1570	0.270589247	1.086394621
0.651144651	-0.179946451
pseudo_1571	0.7775149	-1.113596992
0.439272517	-0.142015495
pseudo_1572	0.036575802	2.119996358
0.200299155	-0.148237593
pseudo_1573	0.471839163	0.454230031
0.383258805	-0.394467223
pseudo_1574	0.527932175	-0.913097591	0.463217455
0.07122642
pseudo_1575	0.354529142	2.216530064	0.643281649
0.100887583
pseudo_1576	0.587238869	-0.05128864
0.660744943	-0.041342464
pseudo_1577	0.402373449	-1.013882488
0.723573247	-0.25667924
pseudo_1578	0.547531115	-0.600092093
0.02759317	-0.891726566
pseudo_1579	0.459888434	-0.549421048	0.137414964
0.581884418
pseudo_158	0.318779915	-2.553073982	0.37900892
0.646020631
pseudo_1580	0.465125769	0.700854931	0.621037427

0.00460447
pseudo_1581	0.557988946	-0.25032363
0.850278185	-0.070094145
pseudo_1582	0.713054719	0.384225237	0.13720818
0.243135961
pseudo_1583	0.208662818	2.674395011
0.634901131	-0.327861831
pseudo_1584	0.099178951	-0.411983155	0.248789674
0.096042769
pseudo_1585	0.47280256	0.00324179
0.161660882	-0.784190922
pseudo_1586	0.753654569	-2.412051926	0.064465858
0.905582332
pseudo_1587	0.047586828	-2.316499942
0.723573247	-0.699278101
pseudo_1588	0.73414425	1.870630283	0.218736869
0.480552099
pseudo_1589	0.876210929	-2.853191975	0.104244266
0.699034482
pseudo_159	0.160492963	1.417692749
0.699114758	-0.351989984
pseudo_1590	0.195669765	-0.735246789
0.559040047	-0.433796614
pseudo_1591	0.704911077	0.761184635	0.213800951
0.186301296
pseudo_1592	0.37478816	-2.977391024	0.784715262
0.271533734
pseudo_1593	0.69911476	-0.251381301	0.839876703
0.259805538
pseudo_1594	0.607853615	-2.447851292
0.070195908	-0.307864442
pseudo_1595	0.693913016	0.261119222	0.6188315
0.101623165
pseudo_1596	0.065488372	1.207577553
0.677250751	-0.254217005
pseudo_1597	0.436963858	-0.14643499	0.238674524
0.21656085
pseudo_1598	0.792536487	-0.408393312	0.031503581
0.814311591
pseudo_1599	0.788322411	0.254040535	0.782913417
0.238961952
pseudo_16	0.685850078	0.981752426
0.742992172	-1.03253521
pseudo_160	0.691605732	-0.771383111
0.419197688	-0.343060495
pseudo_1600	0.18643574	-1.474114861	0.645584356
0.268627584
pseudo_1601	0.983474456	-2.825835942	0.495256891
0.068264168
pseudo_1602	0.341679445	-0.406112842

0.668693288 -0.319894749
pseudo_1603 0.170991822 1.903817618
0.723573247 -0.194310011
pseudo_1604 0.895836283 -0.173966736 0.538202696
0.751676004
pseudo_1605 0.480062442 1.110919234 0.203059408
0.771719927
pseudo_1606 0.820982328 0.289115721
0.387967243 -0.045499896
pseudo_1607 0.129329362 1.220337817 0.52538024
0.065166995
pseudo_1608 0.881632229 0.69744929 0.142025254
0.668613037
pseudo_1609 0.251687807 0.255046427 0.536140597
0.006157459
pseudo_161 0.841098994 0.975970756
0.659046655 -0.753669398
pseudo_1610 0.095704849 -1.921640124 0.874236208
0.139766199
pseudo_1611 0.07352782 -3.747785079
0.698536083 -0.284753447
pseudo_1612 0.392718279 1.265114194 0.755383602
0.148718048
pseudo_1613 0.628230174 1.918013266
0.913792887 -0.452809222
pseudo_1614 0.073151336 -0.669080794 0.819159154
0.044692253
pseudo_1615 0.129132061 0.14114622 0.164015958
0.553054697
pseudo_1616 0.161192948 -0.924515012
0.05367476 -0.573656756
pseudo_1617 0.053577884 -1.690658187 0.374788157
0.105386532
pseudo_1618 0.048856215 -1.195817916
0.38230627 -0.235372099
pseudo_1619 0.748502299 0.814493528
0.568441533 -0.177195921
pseudo_162 0.106937879 -3.673111518
0.410254103 -0.916087714
pseudo_1620 0.460838215 -1.049540656 0.492788924
0.28997674
pseudo_1621 0.714220796 -0.294341507 0.15748622
0.644289319
pseudo_1622 0.794344384 -0.501854127 0.885334155
0.528418512
pseudo_1623 0.506193916 1.204382711
0.959168762 -1.321104736
pseudo_1624 0.310877505 -1.440876155 0.232196251
1.033598269
pseudo_1625 0.940496688 -0.183827239 0.643841983

0.190700719
pseudo_1626 0.896454643 0.86775309 0.486892798
0.055843458
pseudo_1627 0.1772664 1.227350073
0.374720305 -0.180191786
pseudo_1628 0.997193581 -0.724113031
0.30237205 -0.526438407
pseudo_1629 0.546490688 -0.922433049
0.717138906 -0.16895749
pseudo_163 0.191916605 0.627228539
0.031073935 -0.977057527
pseudo_1630 0.115893168 2.156561512 0.458939749
0.130788778
pseudo_1631 0.696223139 -0.90643221 0.683552885
0.201500223
pseudo_1632 0.659612557 -1.230655878 0.898310097
0.272599129
pseudo_1633 0.855182148 -0.216355347 0.645524197
0.082008383
pseudo_1634 0.10796209 -1.709069205
0.360656131 -0.348418103
pseudo_1635 0.247509137 -3.686901749 0.314624295
0.282203135
pseudo_1636 0.627121274 0.233199556
0.751281002 -0.121058433
pseudo_1637 0.270589246 1.551585422
0.09885902 -0.673093148
pseudo_1638 0.161660884 1.261862922
0.259861415 -0.586810263
pseudo_1639 0.825240145 1.133656789
0.79977467 -0.020850489
pseudo_164 0.803400267 0.033073052
0.511709693 -0.049691341
pseudo_1640 0.156112975 -1.55752198
0.596421264 -0.367599021
pseudo_1641 0.993451748 -0.066507355
0.304575794 -0.374633297
pseudo_1642 0.803400267 0.696219534
0.864392023 -0.036420153
pseudo_1643 0.079100296 1.697413737
0.413371382 -0.107678663
pseudo_1644 0.038184412 -2.199989943 0.543014013
0.005914186
pseudo_1645 0.524870614 1.073272745
0.655090824 -0.242726061
pseudo_1646 0.250719158 -1.593413324 0.162835209
0.666350744
pseudo_1647 0.237430837 -1.168651204 0.227044109
0.419947111
pseudo_1648 0.208945901 -0.80744681 0.874236208

0.152498612			
pseudo_1649	0.43973509	−0.642138878	0.203892861
0.488588107			
pseudo_165	0.040229344	−1.91890494	0.807635474
0.213762898			
pseudo_1650	0.250581347	−0.916780444	
0.570679246	−0.134224243		
pseudo_1651	0.133729466	−1.993345133	
0.217568176	−0.302473578		
pseudo_1652	0.085579089	1.885353857	
0.614979387	−0.220463487		
pseudo_1653	0.6261848	0.581990553	0.068874462
0.849849413			
pseudo_1654	0.331461393	0.219677073	0.978486682
0.188076521			
pseudo_1655	0.78411452	−0.359190887	0.336938623
0.205953433			
pseudo_1656	0.961037373	0.263061582	
0.438348213	−0.537449525		
pseudo_1657	0.153618979	1.067573393	
0.231585845	−0.794339393		
pseudo_1658	0.428710763	−1.212910012	0.199202758
0.309708132			
pseudo_1659	0.527932175	−0.373283061	
0.995946292	−0.039396966		
pseudo_166	0.418298205	1.05813046	0.496740833
0.170618446			
pseudo_1660	0.807635475	0.699568123	
0.57066519	−0.132788215		
pseudo_1661	0.815567788	0.168447042	
0.156058622	−0.339999926		
pseudo_1662	0.287951986	0.346481163	
0.943606708	−0.203320342		
pseudo_1663	0.551243027	0.351306137	
0.698478428	−0.04240766		
pseudo_1664	0.447174154	−1.062352518	
0.52946636	−0.348555627		
pseudo_1665	0.385822692	0.341437118	0.398798552
1.052297751			
pseudo_1666	0.484447376	−0.676489156	0.833771099
0.182612466			
pseudo_1667	0.101768221	1.097898237	0.741810456
0.37018603			
pseudo_1668	0.741810457	0.07845907	
0.093392506	−0.575579804		
pseudo_1669	0.688150176	0.454517477	0.215823568
0.519396462			
pseudo_167	0.637689513	0.251131244	0.289728689
0.611814563			
pseudo_1670	0.211220588	−2.604027222	0.142663028

0.167738814			
pseudo_1671	0.961660291	0.041185294	0.705491657
0.10905646			
pseudo_1672	0.223754115	0.981624076	
0.954186926	-0.188730044		
pseudo_1673	0.791331841	-0.189865623	0.310131692
0.311503961			
pseudo_1674	0.373108022	-0.750862298	
0.135357912	-0.718381978		
pseudo_1675	0.827675502	0.489724206	0.260192139
0.202797202			
pseudo_1676	0.905118112	-0.209898303	0.394878476
0.689888041			
pseudo_1677	0.847828362	0.367844184	0.326042059
0.668983967			
pseudo_1678	0.044601157	-1.700366093	
0.19405473	-0.317863482		
pseudo_1679	0.216403983	1.282068757	
0.557988944	-0.301408683		
pseudo_168	0.972564808	-0.276512839	
0.751874174	-0.403206462		
pseudo_1680	0.408922416	-1.072394708	0.650018959
0.299210796			
pseudo_1681	0.503198545	-0.079108366	
0.255588485	-0.827602344		
pseudo_1682	0.810664088	-1.473071	0.310877503
0.055931302			
pseudo_1683	0.589932775	0.754322683	0.355342252
0.199532884			
pseudo_1684	0.752467494	0.492944807	
0.783513904	-0.453852012		
pseudo_1685	0.778669671	0.618244286	
0.332414327	-0.156137268		
pseudo_1686	0.259179134	0.861151453	
0.351247026	-0.215840627		
pseudo_1687	0.68207833	0.941934252	0.434115664
0.334350136			
pseudo_1688	0.411143328	-0.917138943	0.767347143
0.163285666			
pseudo_1689	0.103082888	-0.763084488	
0.44904504	-0.170189536		
pseudo_169	0.123702027	-1.08400886	0.08963841
0.838051643			
pseudo_1690	0.613880725	-0.452767484	
0.031689269	-0.596673067		
pseudo_1691	0.702010751	0.72051176	0.303840031
0.379338593			
pseudo_1692	0.834176996	-0.239719242	
0.146433476	-0.703693417		
pseudo_1693	0.308272339	1.02356424	

0.294742663 -0.21359082
pseudo_1694 0.214209487 -2.948502494 0.661638856
0.17204797
pseudo_1695 0.146101679 2.199513497 0.649456413
0.101350153
pseudo_1696 0.363125422 -0.516790305 0.961037372
0.146581758
pseudo_1697 0.091876004 1.94543223 0.480548584
0.204263
pseudo_1698 0.906976075 -2.47310313
0.140756279 -0.349842993
pseudo_1699 0.393576982 -3.864155379 0.619934036
0.260205631
pseudo_17 0.401863639 0.753337272 0.642721518
0.346301799
pseudo_170 0.181485411 -1.441071579
0.484935926 -0.037238008
pseudo_1700 0.048856215 1.595872919
0.927760934 -0.200320403
pseudo_1701 0.92645119 -0.366438877 0.297890426
0.299021148
pseudo_1702 0.330683656 0.34557769
0.364364022 -0.373989665
pseudo_1703 0.66131143 -0.779462486
0.247509135 -0.242051336
pseudo_1704 0.243379282 1.692714833
0.963529184 -0.128477009
pseudo_1705 0.361996109 -1.263176934 0.007349076
0.915797728
pseudo_1706 0.518268556 -1.238954931
0.588315746 -0.318106132
pseudo_1707 0.51221268 -0.255516935 0.090975638
0.605849609
pseudo_1708 0.361478056 2.057874623 0.541303288
0.17922597
pseudo_1709 0.98971006 0.306124677
0.039699916 -0.519371661
pseudo_171 0.609494755 0.966477161
0.299086371 -0.04245387
pseudo_1710 0.246233219 1.465263102
0.233113998 -0.487363316
pseudo_1711 0.770931293 -1.036115023
0.933657902 -0.302214904
pseudo_1712 0.88965623 -0.596040728 0.140545633
0.212912145
pseudo_1713 0.326042061 -2.055528849 0.012555537
0.514703629
pseudo_1714 0.536140599 -1.00085509 0.895836283
0.201572382
pseudo_1715 0.474732598 0.779581715

0.260192139 -0.700724602
pseudo_1716 0.700272634 -0.577485304 0.670401385
0.828370736
pseudo_1717 0.271269673 -0.271654273 0.320680657
0.265702434
pseudo_1718 0.427344115 0.823530399
0.896454643 -0.537197226
pseudo_1719 0.814927879 -0.491177563 0.418304078
0.433481316
pseudo_172 0.485114883 -0.599198491
0.16215881 -0.343222903
pseudo_1720 0.256570188 2.020054143
0.365191224 -0.821768177
pseudo_1721 0.433743502 0.372040846 0.627121272
0.215782526
pseudo_1722 0.066408464 -2.23081994 0.473284662
0.111857935
pseudo_1723 0.507194453 0.327600089 0.652271138
0.304093433
pseudo_1724 0.894599755 -0.999328985
0.731202702 -0.093127295
pseudo_1725 0.086579722 1.318584855
0.803400266 -0.159396091
pseudo_1726 0.942362594 -0.284769498
0.604031974 -0.177412382
pseudo_1727 0.81612271 -1.816274486 0.307160271
0.306421062
pseudo_1728 0.788924046 -0.402161461 0.796756631
0.394046703
pseudo_1729 0.561672003 0.557574892 0.297271328
0.303340042
pseudo_173 0.434600135 0.980364552
0.921868677 -0.168673084
pseudo_1730 0.670401386 -0.353244987 0.873004587
0.169687018
pseudo_1731 0.178327377 0.911815502
0.716866978 -0.166878124
pseudo_1732 0.578122468 -0.853906422 0.360656131
0.836392309
pseudo_1733 0.634901132 0.733041292 0.203614767
0.402143583
pseudo_1734 0.285476969 2.095049491 0.819159154
0.186591011
pseudo_1735 0.175681227 -0.424279604 0.607853614
0.169465173
pseudo_1736 0.601853038 0.477064188 0.83499143
0.18376207
pseudo_1737 0.81612271 0.311810936
0.730027186 -0.204658581
pseudo_1738 0.230672377 0.642696795 0.047674543

0.097648655
pseudo_1739 0.689877146 0.254471499 0.259861415
0.66747243
pseudo_174 0.622694114 0.477500577 0.08291014
0.794103926
pseudo_1740 0.417399857 0.476125336 0.763767964
0.720287121
pseudo_1741 0.584759155 -2.462116523 0.407020742
1.056448901
pseudo_1742 0.528954715 1.016206486 0.368095652
0.73065468
pseudo_1743 0.013616929 -1.989213805
0.489836087 -0.391163196
pseudo_1744 0.495751277 0.540810985 0.53049041
0.180021784
pseudo_1745 0.023430919 1.623622241
0.087589778 -1.097174859
pseudo_1746 0.811876329 0.409412502
0.847216135 -0.50538964
pseudo_1747 0.510704498 -0.596762111
0.352906442 -0.885783077
pseudo_1748 0.215533784 0.434450104
0.52946636 -0.126614282
pseudo_1749 0.220498353 1.074611339 0.854568842
0.042100797
pseudo_175 0.692182286 0.425297459 0.994075379
0.3440018
pseudo_1750 0.452800109 -0.830514251 0.014130628
1.211660668
pseudo_1751 0.887186063 0.023262913 0.889656229
0.328944091
pseudo_1752 0.494762772 0.519688269
0.286889496 -0.228638332
pseudo_1753 0.22494651 -0.756716996
0.849665593 -0.103943683
pseudo_1754 0.695645343 -0.249231417 0.92682401
0.41263853
pseudo_1755 0.415606575 -0.052166554
0.623246767 -0.126634838
pseudo_1756 0.957300377 0.420227505 0.557988944
0.127168375
pseudo_1757 0.502202173 -0.510174439 0.882249039
0.32845009
pseudo_1758 0.661878109 -3.511578271 0.014223036
0.680187636
pseudo_1759 0.855795541 1.190972243
0.576520626 -0.51792087
pseudo_176 0.992204499 0.189665992 0.688725651
0.564733335
pseudo_1760 0.750377059 -0.015546442 0.649731189

0.240571611
pseudo_1761 0.168320097 -1.879030697
0.025208295 -0.791571544
pseudo_1762 0.506694055 1.620909326 0.821590267
0.588032789
pseudo_1763 0.292945326 0.360005262
0.290441433 -0.516207092
pseudo_1764 0.830722028 0.537696601 0.747132931
0.333731741
pseudo_1765 0.529466362 0.856461884 0.238986164
0.284154873
pseudo_1766 0.858863804 0.012500647 0.623799633
0.407593045
pseudo_1767 0.484447376 0.853082697
0.363537995 -0.283258406
pseudo_1768 0.515235998 0.594330754
0.22855091 -0.600801524
pseudo_1769 0.684126911 -1.98726393 0.17197119
0.277431973
pseudo_177 0.356156543 -2.006901219 0.924961136
0.170722051
pseudo_1770 0.691605732 -2.524168087 0.067222253
0.628066884
pseudo_1771 0.545970842 0.666883779 0.131715268
0.309455194
pseudo_1772 0.784715264 0.219776625 0.133526979
0.675216943
pseudo_1773 0.616078915 -1.003276046
0.37310802 -0.460083193
pseudo_1774 0.654526493 -0.80685363
0.553270914 -0.625192152
pseudo_1775 0.862548532 -1.324738368 0.981603955
0.049197104
pseudo_1776 0.670401386 0.110129826
0.14033523 -0.658823679
pseudo_1777 0.719476374 0.906476145
0.344863587 -0.187587476
pseudo_1778 0.682979045 -4.152496165 0.27641022
0.248061375
pseudo_1779 0.162599832 1.085521425 0.924961136
0.093961874
pseudo_178 0.298722775 -0.346961655
0.586162903 -0.643918795
pseudo_1780 0.215533784 1.526812819 0.648331919
0.430777942
pseudo_1781 0.747132932 -0.416229451
0.170747631 -0.375487887
pseudo_1782 0.058188016 -3.198728538
0.785316136 -0.129334377
pseudo_1783 0.990957268 -0.48461712 0.11937637

0.736869156
pseudo_1784 0.156112975 −1.039120898 0.76854131
0.13176965
pseudo_1785 0.976986542 −0.711049641 0.121856405
0.519675433
pseudo_1786 0.241803843 0.932748007 0.416502645
0.624176347
pseudo_1787 0.81551575 0.175231146 0.663012046
0.061233152
pseudo_1788 0.092328863 −0.519381869 0.598591294
0.261436071
pseudo_1789 0.101441649 −1.138021416
0.060936431 −0.373943992
pseudo_179 0.975369738 −0.326615649
0.799170822 −0.106381062
pseudo_1790 0.059022494 −1.436922793
0.056447269 −0.230757265
pseudo_1791 0.731202703 −0.648586926
0.582940507 −0.296963059
pseudo_1792 0.201124341 0.765438213
0.333020408 −0.166779314
pseudo_1793 0.288661784 1.618492141
0.627121272 −0.098377555
pseudo_1794 0.612234355 0.668811115 0.445773901
0.316271949
pseudo_1795 0.938631108 −0.313544609
0.735321964 −0.262246743
pseudo_1796 0.847828362 −0.578773476
0.453741638 −0.379557415
pseudo_1797 0.594995649 0.390626833 0.134243391
0.73590864
pseudo_1798 0.206127569 0.653495465
0.006100192 −0.758308642
pseudo_1799 0.407150838 0.460791583
0.011262938 −0.626788784
pseudo_18 0.379856571 −0.789919641
0.676678941 −0.133831132
pseudo_180 0.421153426 −0.310364582 0.99473909
0.165242699
pseudo_1800 0.095860621 1.704806303 0.436042352
0.354410461
pseudo_1801 0.934900969 −2.394769663 0.256242662
0.188819749
pseudo_1802 0.516751124 −0.177894523 0.825848827
0.15282329
pseudo_1803 0.821973578 −0.86803863
0.489765517 −0.109650201
pseudo_1804 0.778713653 −0.129678277 0.379856569
0.251454869
pseudo_1805 0.786217684 −1.075729714

0.553794172 -0.179622733
pseudo_1806 0.230672377 -1.312571213 0.205008 0.093999099
pseudo_1807 0.957923146 -0.866532902 0.567483299
0.084975291
pseudo_1808 0.384966902 -4.633623106
0.595337594 -0.396269515
pseudo_1809 0.923719448 0.194125904 0.219322903
0.50036463
pseudo_181 0.943606708 -0.565962893 0.357788644
0.262093424
pseudo_1810 0.214666108 -1.069913006 0.630450488
0.460256476
pseudo_1811 0.113378099 1.363878648 0.118083661
0.098330256
pseudo_1812 0.801586935 -0.836401896 0.326234608
0.299737609
pseudo_1813 0.356156543 -0.493411012 0.687574879
0.197864815
pseudo_1814 0.361066948 -1.569038845 0.834381215
0.180427507
pseudo_1815 0.890891717 -0.329711661
0.074158813 -0.750922831
pseudo_1816 0.952319189 1.101868423 0.205008 0.350616655
pseudo_1817 0.493775321 -0.544437333
0.402302658 -0.179167489
pseudo_1818 0.294742665 0.964478088 0.198656206
0.422754161
pseudo_1819 0.893981586 1.474956462 0.629339911
0.182435998
pseudo_182 0.576520627 1.344770014
0.28442066 -0.235870983
pseudo_1820 0.358197406 -2.123392159 0.649456413
0.265358914
pseudo_1821 0.985345049 -0.407960156
0.588854528 -0.394440811
pseudo_1822 0.48885393 0.534979084 0.905118111
0.381624949
pseudo_1823 0.631006092 -1.264040861 0.878549261
0.000892158
pseudo_1824 0.414711644 0.543470741
0.596421264 -0.189759221
pseudo_1825 0.512715923 0.510655187 0.292586741
0.381300629
pseudo_1826 0.513219423 -0.222103835 0.103745266
0.395071319
pseudo_1827 0.56230955 -1.813707988
0.056068643 -0.367089269
pseudo_1828 0.696223139 -0.057974981
0.787720901 -0.144941969
pseudo_1829 0.973499749 0.804026063 0.710724575

0.531599135
pseudo_183 0.753654569 0.876866945 0.170503703
0.054046393
pseudo_1830 0.273717727 -0.392553725 0.433147663
0.01131623
pseudo_1831 0.562199112 0.439630058 0.145452144
0.286248192
pseudo_1832 0.117533018 1.532128432 0.771529128
0.405826619
pseudo_1833 0.421902964 -0.400121127
0.15748622 -0.396630074
pseudo_1834 0.552747899 -0.517760178 0.957300376
0.204418675
pseudo_1835 0.201399952 0.826916
0.394878475 -0.036812209
pseudo_1836 0.936765865 0.475914549 0.229761468
0.458801799
pseudo_1837 0.048648423 -1.922371209
0.192716361 -0.446401848
pseudo_1838 0.581332415 -0.42718347 0.989086465
0.023877832
pseudo_1839 0.451752225 -0.4536186 0.611079579
0.137705185
pseudo_184 0.927445058 -0.337357791 0.013148589
0.792293117
pseudo_1840 0.793138996 -0.884337482
0.135357912 -0.181313809
pseudo_1841 0.082633167 1.680918722
0.16904562 -0.326324148
pseudo_1842 0.850890868 -0.456165022
0.216403981 -0.602238869
pseudo_1843 0.594796098 -0.414389089 0.52946636
0.274669323
pseudo_1844 0.372688717 0.742120679 0.226143433
0.652465736
pseudo_1845 0.710724576 -0.729140384
0.143945168 -0.493618802
pseudo_1846 0.073905887 -0.762337813
0.462741059 -0.520153864
pseudo_1847 0.474357035 -0.752427786
0.493919474 -0.152235172
pseudo_1848 0.497308397 -0.778526704 0.65626958
0.335084477
pseudo_1849 0.130717028 1.279644926
0.203336949 -0.247862091
pseudo_185 0.935522562 1.166213584 0.309387063
0.397238606
pseudo_1850 0.282666036 -0.732964097
0.544931884 -0.833296966
pseudo_1851 0.319026209 0.032397679 0.488729974

0.656579368
pseudo_1852 0.714220796 1.452554559
0.439966477 -0.10823511
pseudo_1853 0.118083663 -1.457815575 0.868081211
0.235316466
pseudo_1854 0.295463666 -0.258760362
0.454684271 -0.43452844
pseudo_1855 0.860705789 0.200543993
0.156798457 -0.600567531
pseudo_1856 0.236589264 -0.897979075 0.134135148
0.153387382
pseudo_1857 0.931172258 -2.237807422 0.817944248
0.314077952
pseudo_1858 0.563781871 0.545689345 0.741219826
0.191468176
pseudo_1859 0.148063598 2.42083717 0.247198616
0.802523378
pseudo_186 0.201124341 0.579167722
0.295463661 -0.407050127
pseudo_1860 0.810058137 -0.074033303 0.017699067
0.677010158
pseudo_1861 0.074412447 0.641399021
0.024653219 -1.124784966
pseudo_1862 0.096016595 -1.288844471
0.153618977 -0.38188967
pseudo_1863 0.550136461 1.170191351
0.126394338 -0.745273515
pseudo_1864 0.995322652 -0.483216697
0.186908081 -0.320129133
pseudo_1865 0.239610306 1.058097327 0.997193581
0.394440947
pseudo_1866 0.040611161 1.737838616 0.488853928
0.316234451
pseudo_1867 0.095115328 1.264618934 0.920559857
0.073386132
pseudo_1868 0.475215783 -0.605038633 0.88101549
0.007414573
pseudo_1869 0.367264354 0.806568143
0.17730732 -0.729145902
pseudo_187 0.388827096 -0.849012509
0.930550951 -0.441494688
pseudo_1870 0.586162904 -0.495693622
0.223159613 -0.786211272
pseudo_1871 0.70897874 -0.852912486 0.764960467
0.050417999
pseudo_1872 0.591551846 1.817737872 0.674964628
0.160824027
pseudo_1873 0.88224904 0.332960457
0.389257455 -0.681567533
pseudo_1874 0.969760218 -1.271507653

0.095238749 -0.49406412
pseudo_1875 0.778669671 -0.540591084 0.325472722
0.374849757
pseudo_1876 0.510192544 -1.931512936 0.807258714
0.281202261
pseudo_1877 0.030289354 0.624775303
0.080039146 -0.681209673
pseudo_1878 0.921236633 1.912153848 0.49328199
0.814018933
pseudo_1879 0.247828839 -0.358910747 0.478605619
0.292150243
pseudo_188 0.744174494 0.0840607
0.454212817 -0.403051147
pseudo_1880 0.483471074 0.415025868
0.818551647 -0.594027656
pseudo_1881 0.158406791 -1.104169841
0.814302163 -0.066517688
pseudo_1882 0.302005796 -0.315165939
0.919375024 -0.828180686
pseudo_1883 0.135357913 -0.727548137 0.660178652
0.214521429
pseudo_1884 0.165441361 -0.515474669
0.586700771 -0.262074092
pseudo_1885 0.911313286 -0.203897831
0.651144651 -0.139498103
pseudo_1886 0.700851832 -0.512857984 0.278138399
0.128735428
pseudo_1887 0.413817852 2.243457465 0.299814455
0.257117731
pseudo_1888 0.111608094 -1.635286972
0.313497153 -0.254858663
pseudo_1889 0.881411066 0.396456949 0.391845697
0.120014825
pseudo_189 0.893363482 0.533156694 0.23588267
0.248875952
pseudo_1890 0.245914963 -1.444097672
0.825240145 -0.254855799
pseudo_1891 0.518774878 0.2152026
0.17197119 -0.684677534
pseudo_1892 0.098699367 1.190795159
0.374367685 -0.354120354
pseudo_1893 0.825240145 0.62634526
0.634901131 -0.093142627
pseudo_1894 0.334886973 -3.566975197 0.455725773
0.305466782
pseudo_1895 0.209229262 -0.848859134 0.77332345
0.088790018
pseudo_1896 0.217276708 -2.469719891
0.719476373 -0.297540238
pseudo_1897 0.154859754 2.555963401

0.861291038	-0.144485513
pseudo_1898	0.294382607	-0.519278374	0.722401897
0.433834636
pseudo_1899	0.736500302	-1.201746535
0.521310315	-0.013315327
pseudo_19	0.379432601	-2.172027742
0.839265702	-0.402060242
pseudo_190	0.889038587	0.32263767	0.697379259
0.252788979
pseudo_1900	0.794344384	1.148998782	0.132317043
0.518859249
pseudo_1901	0.408479092	-0.710374848
0.181996768	-0.243465535
pseudo_1902	0.959791608	-0.74690582	0.123702026
0.623076589
pseudo_1903	0.110731305	1.54721317
0.953875619	-0.212703883
pseudo_1904	0.991580881	-0.346332099	0.438810225
0.225470046
pseudo_1905	0.877932887	0.523769393
0.207815234	-0.378945142
pseudo_1906	0.228853125	-0.801461218
0.270589245	-0.276110353
pseudo_1907	0.034057687	-3.469468636
0.074666788	-0.935391531
pseudo_1908	0.808240969	-1.00827495
0.916273328	-0.315422326
pseudo_1909	0.535111043	-0.613528878
0.356156541	-0.176193636
pseudo_191	0.178439631	-0.329440406
0.608400441	-0.279756957
pseudo_1910	0.417848889	-0.639588331	0.617179303
0.275401324
pseudo_1911	0.055330591	1.855746358
0.588162212	-0.216369901
pseudo_1912	0.802795705	0.445479199
0.15748622	-0.205200913
pseudo_1913	0.512715923	-0.71113211	0.516751123
0.20623861
pseudo_1914	0.501206846	0.742209847
0.289372759	-0.584865608
pseudo_1915	0.353514403	0.322120654	0.591011927
0.126237988
pseudo_1916	0.921236633	0.809103522
0.799774671	-0.253804399
pseudo_1917	0.256242664	-1.360314439	0.74476588
0.414179617
pseudo_1918	0.099821287	-0.45996152	0.426889127
0.907257929
pseudo_1919	0.74950237	-0.067708514	0.259200842

0.753898181			
pseudo_192	0.367679857	0.438475994	
0.193250892	-0.53378977		
pseudo_1920	0.294022845	-0.956068013	0.684126909
0.086344123			
pseudo_1921	0.703750429	0.555788973	0.808846576
0.178852378			
pseudo_1922	0.081300677	-3.423669382	0.441904548
1.014788866			
pseudo_1923	0.770333593	0.500186866	
0.624906001	-0.089621766		
pseudo_1924	0.760789163	0.527061688	
0.919375024	-0.180270129		
pseudo_1925	0.943606708	-0.263085208	
0.726504451	-0.079354695		
pseudo_1926	0.55956596	-0.904652833	0.556414095
0.566784915			
pseudo_1927	0.301639834	-1.183667991	0.501704377
0.536293583			
pseudo_1928	0.007917079	-0.987537496	0.590472236
0.34411464			
pseudo_1929	0.269909993	-0.63734008	
0.449982147	-0.056504874		
pseudo_193	0.254683112	-1.468124953	0.576573119
0.155848422			
pseudo_1930	0.051953189	-3.186146421	0.42779938
0.287243504			
pseudo_1931	0.394010525	0.508709485	0.021943381
0.796782153			
pseudo_1932	0.845380009	-0.620411668	0.263849325
0.549841157			
pseudo_1933	0.947339864	-0.197649273	
0.505194416	-0.344042807		
pseudo_1934	0.816122708	-1.479469787	
0.407150837	-0.312965099		
pseudo_1935	0.20375378	0.882015263	0.552225124
0.345521279			
pseudo_1936	0.18560624	-1.920567107	
0.589393537	-0.130979815		
pseudo_1937	0.074666789	-1.004947482	
0.073151335	-0.521478286		
pseudo_1938	0.460363188	0.258511295	0.146101677
0.255977719			
pseudo_1939	0.221087769	1.436775636	0.592632358
0.117641971			
pseudo_194	0.898310097	1.023576667	0.910693519
0.35248785			
pseudo_1940	0.908215001	1.04857028	
0.947962171	-0.654678191		
pseudo_1941	0.069592695	-2.224704857	

0.353311675 -0.154673061
pseudo_1942 0.434662201 0.306157521 0.206970152
0.363237847
pseudo_1943 0.093851353 1.915994547 0.482495838
0.059567544
pseudo_1944 0.51776249 0.243474076 0.490327565
0.12456225
pseudo_1945 0.294382607 1.836449839
0.592632358 -0.082041913
pseudo_1946 0.137829254 1.508265046 0.813695536
0.049838529
pseudo_1947 0.796153387 1.365648999
0.829503109 -0.224117498
pseudo_1948 0.123702027 1.879445386 0.323354116
0.405102683
pseudo_1949 0.557988946 -1.161133305
0.772725207 -0.025772199
pseudo_195 0.055543389 -1.375369814
0.094465959 -0.60526776
pseudo_1950 0.898310097 -0.028195838
0.586700771 -0.386986454
pseudo_1951 0.503860207 -0.575756044 0.081123184
0.618449009
pseudo_1952 0.946095346 0.376205613
0.997817228 -0.028444735
pseudo_1953 0.671541066 -0.485399843 0.688725651
0.124766417
pseudo_1954 0.729439666 0.303430138 0.263181758
0.641066235
pseudo_1955 0.344863589 -0.762325396 0.547010777
0.336293293
pseudo_1956 0.170747633 -1.081734283 0.939874791
0.278014834
pseudo_1957 0.1282471 -0.924170992 0.674964628
0.024374698
pseudo_1958 0.522834635 0.282740853 0.260854459
0.137093427
pseudo_1959 0.8094523 0.765335511 0.458465819
0.432495933
pseudo_196 0.911933107 0.333004879 0.926203007
0.179246703
pseudo_1960 0.73296716 -0.561807324 0.073151335
0.386498157
pseudo_1961 0.407372034 0.529434886 0.142237602
0.294529053
pseudo_1962 0.253958128 -0.289896762 0.073527819
0.526422429
pseudo_1963 0.89769155 -0.549265373
0.47618296 -0.091406587
pseudo_1964 0.772127101 -0.769728073

0.520295375 -0.283895279
pseudo_1965 0.515235998 0.361853829 0.586700771
0.243511079
pseudo_1966 0.595879318 0.02838686 0.432367558
0.616441714
pseudo_1967 0.35689578 0.471601047 0.181610083
0.313108392
pseudo_1968 0.011619622 2.872448347
0.308457938 -0.957172963
pseudo_1969 0.589932775 0.638277832 0.321443024
0.316664937
pseudo_197 0.222566246 1.538968531 0.251687804
0.270416305
pseudo_1970 0.24496192 0.353320121
0.997817228 -0.176301569
pseudo_1971 0.011542357 1.530183938 0.53254152
0.284326764
pseudo_1972 0.346062507 -0.562749232 0.271610323
0.183281946
pseudo_1973 0.756624801 0.120188436
0.414711642 -0.663456355
pseudo_1974 0.091575894 -1.017660211
0.875561282 -0.12313559
pseudo_1975 0.862548533 -0.07239193
0.76138464 -0.403318862
pseudo_1976 0.042646484 1.439409306 0.121995029
0.533660823
pseudo_1977 0.657915439 -0.301191244 0.496245922
0.24617424
pseudo_1978 0.761384641 0.247605374 0.693335926
0.169534227
pseudo_1979 0.062901502 -1.615967715 0.044435429
0.864308689
pseudo_198 0.876700361 -0.920118223
0.955432225 -0.262554733
pseudo_1980 0.352501505 0.043378639 0.838043992
0.205781378
pseudo_1981 0.190059999 1.364534553
0.63211792 -0.477196128
pseudo_1982 0.885951389 0.427602945 0.023192873
0.766440122
pseudo_1983 0.273146883 -1.453917058 0.749502369
0.182406131
pseudo_1984 0.627121274 -0.428199353 0.434202709
0.881240051
pseudo_1985 0.83621216 -1.056344925
0.051764785 -1.021636405
pseudo_1986 0.193786514 -1.070665594 0.633230582
0.114519537
pseudo_1987 0.735321965 -0.153507484 0.676678941

0.277993723
pseudo_1988 0.562199112 0.546413053
0.275376833 -0.27713602
pseudo_1989 0.843544721 1.163538851
0.146971171 -0.846585743
pseudo_199 0.865621429 0.907283568 0.858249979
0.291141827
pseudo_1990 0.943606708 1.390715695 0.332240309
0.086610626
pseudo_1991 0.631561902 0.613589557
0.65622008 -0.346221327
pseudo_1992 0.852729455 0.656272062
0.074285541 -0.80159805
pseudo_1993 0.585625265 0.69050667 0.771529128
0.335152246
pseudo_1994 0.465125769 -2.34835252
0.565895549 -0.311098931
pseudo_1995 0.109465202 -1.833893941
0.687528827 -0.013446842
pseudo_1996 0.137218378 -3.094304397
0.532077799 -0.456953885
pseudo_1997 0.895836283 0.102689647
0.719476373 -0.411068779
pseudo_1998 0.986848224 -0.622689567
0.426886164 -0.765781958
pseudo_1999 0.736500302 0.521720296 0.657350122
0.326646098
pseudo_2 0.538460738 -0.38906025 0.099419437 -0.24871872
pseudo_20 0.782313061 0.597510828 0.409809921
0.171808336
pseudo_200 0.080583648 1.604925419
0.869718291 -0.297530454
pseudo_2000 0.013947403 -1.318644551
0.602942062 -0.217170869
pseudo_2001 0.872388893 -0.540366927
0.281966242 -0.292475308
pseudo_2002 0.571196331 -0.298363844
0.685850076 -0.150485467
pseudo_2003 0.523852117 0.462083151
0.768541311 -0.099457212
pseudo_2004 0.261622711 0.40677964 0.225358499
0.546003112
pseudo_2005 0.350481222 0.035234571 0.046976607
0.584089103
pseudo_2006 0.180975119 -2.421472601
0.653398419 -0.256605054
pseudo_2007 0.401863639 -0.799003848 0.49328199
0.507258759
pseudo_2008 0.576520627 0.478724658
0.869311584 -0.355244738

pseudo_2009 0.286182644 -0.632780301
0.553270914 -0.300192974
pseudo_201 0.228248984 -0.585949379
0.787119519 -0.487718166
pseudo_2010 0.4868928 0.898908044
0.991580881 -0.111059534
pseudo_2011 0.164965194 -0.709109426 0.468955471
0.065385368
pseudo_2012 0.422806991 -0.282388066 0.143302998
0.757612968
pseudo_2013 0.633230583 0.670355686
0.288306736 -0.106304793
pseudo_2014 0.264852865 0.529245646 0.722987491
0.111011063
pseudo_2015 0.69908861 1.758580151
0.906177812 -0.243815526
pseudo_2016 0.074158814 1.793934132 0.917513856
0.301022208
pseudo_2017 0.106937879 1.276444877
0.657915438 -0.374525321
pseudo_2018 0.511206967 -0.386838327 0.034589214
0.613969005
pseudo_2019 0.346462736 -0.130305224 0.119191021
0.402284981
pseudo_202 0.192716363 -0.376085143
0.237430835 -0.298873558
pseudo_2020 0.689877146 0.864491215
0.297633747 -0.565445884
pseudo_2021 0.748317354 -0.304776925
0.451389883 -0.34368172
pseudo_2022 0.074158814 1.103155016 0.510704496
0.535953267
pseudo_2023 0.780512769 0.035895311
0.384112272 -0.131532616
pseudo_2024 0.218736871 0.566173739
0.052617119 -0.719275509
pseudo_2025 0.982227445 -2.040192265 0.788924044
0.104851621
pseudo_2026 0.779301896 -0.2033858 0.839971723
0.26411992
pseudo_2027 0.217568177 -3.369860671 0.725331486
0.095186582
pseudo_2028 0.737089704 -0.459218373
0.628230172 -0.066605012
pseudo_2029 0.104077723 1.04264966
0.351692507 -0.253063104
pseudo_203 0.895217987 -1.567055281 0.905118111
0.111166512
pseudo_2030 0.885951389 -0.196267317
0.135972514 -0.947364755

pseudo_2031 0.129922669 -0.760189848 0.538202696
0.25929338
pseudo_2032 0.449513457 -4.193342766 0.455628007
0.312259673
pseudo_2033 0.275376835 0.176556789
0.256570186 -0.605285404
pseudo_2034 0.18643574 1.349637648 0.574605308
0.232196655
pseudo_2035 0.551702595 0.536616463 0.232807798
0.189451437
pseudo_2036 0.299450268 -0.649351532
0.401424905 -0.849542012
pseudo_2037 0.190324415 0.46129948
0.096172772 -0.184420919
pseudo_2038 0.376472966 0.246826341
0.282666034 -0.435932731
pseudo_2039 0.669831831 0.461628056 0.169773489
0.535218546
pseudo_204 0.619934036 0.329761788
0.250074837 -0.746470443
pseudo_2040 0.470396098 0.418894186
0.506694053 -0.073332568
pseudo_2041 0.470396098 0.643025807 0.395747584
0.079320885
pseudo_2042 0.553270915 0.757173947
0.564309933 -0.824988989
pseudo_2043 0.569603618 -0.059095646
0.762576021 -0.187330992
pseudo_2044 0.543893912 0.584964271
0.784114519 -0.10659857
pseudo_2045 0.838654799 0.583333394 0.581332413
0.279825583
pseudo_2046 0.050278053 1.300760969 0.248148827
0.245926739
pseudo_2047 0.633230583 -0.271043932 0.226443376
0.739878785
pseudo_2048 0.599677652 0.805457224
0.145452144 -0.583325843
pseudo_2049 0.564309934 -1.412427668 0.35574925
0.255807642
pseudo_205 0.64833192 -0.204847787
0.081944009 -0.498633795
pseudo_2050 0.873004588 0.172558374 0.279526229
0.179881993
pseudo_2051 0.495256892 -0.184698343 0.028543484
0.851323338
pseudo_2052 0.860705789 -0.269708592
0.146101677 -0.571022131
pseudo_2053 0.67553588 0.425015523
0.830112517 -0.273801841

pseudo_2054   0.265187962   0.215746283   0.754248324
0.62458599
pseudo_2055   0.596421265   0.609830487   0.995322652
0.394359807
pseudo_2056   0.040305463   -1.725793148
0.15748622   -0.282898989
pseudo_2057   0.016465536   -1.812886597   0.320680657
0.459621347
pseudo_2058   0.757263722   0.727298459
0.102349245   -0.568570027
pseudo_2059   0.350481222   -0.36587448   0.321061693
0.269267161
pseudo_206   0.33302041   0.268632182   0.395747584
0.244730834
pseudo_2060   0.657915439   -0.614863457   0.343269157
0.779976643
pseudo_2061   0.69166963   0.097374844   0.359641094
0.019524209
pseudo_2062   0.984721509   -0.425169633   0.597505831
0.164500162
pseudo_2063   0.655655355   -2.246643786
0.370596573   -0.577160741
pseudo_2064   0.275032959   -0.301946198   0.728852304
0.190823563
pseudo_2065   0.644963258   -0.683058044   0.962906197
0.26279484
pseudo_2066   0.129329362   -3.008052571   0.201952001
1.087531198
pseudo_2067   0.119561946   2.253922103
0.644963257   -0.087230367
pseudo_2068   0.248789676   0.9378366
0.633230582   -0.08572358
pseudo_2069   0.377739633   -1.449788416   0.248789674
0.346501787
pseudo_207   0.064127964   -0.84157529
0.734144249   -0.369274413
pseudo_2070   0.207533265   1.410519014
0.316319995   -0.06866444
pseudo_2071   0.026884993   -1.463701127   0.046030995
0.779641268
pseudo_2072   0.767944159   -0.349003283   0.217568176
0.81151821
pseudo_2073   0.632117921   -3.799818257
0.013119796   -1.348222889
pseudo_2074   0.711889311   -0.74857114
0.921236633   -0.42544999
pseudo_2075   0.211220588   0.134397109
0.098859021   -0.632535568
pseudo_2076   0.496245924   -0.236029628   0.371432554
0.645989227

pseudo_2077 0.283590857 -0.762172765
0.038338124 -0.765323184
pseudo_2078 0.903260667 -0.257959001
0.034189909 -0.643862771
pseudo_2079 0.569073185 -1.077325991
0.747725066 -0.166001387
pseudo_208 0.859477715 -1.688782711 0.763767964
0.806266364
pseudo_2080 0.811270152 0.437049633 0.812482619
0.363501561
pseudo_2081 0.2328078 -2.230026567
0.634344074 -0.412137979
pseudo_2082 0.244328001 -0.314023282 0.242748241
0.204501789
pseudo_2083 0.928066149 -0.834915852
0.505694035 -0.128734544
pseudo_2084 0.972876451 -0.002701507
0.66244498 -0.279845743
pseudo_2085 0.723573249 -0.636853965
0.776915721 -0.125459294
pseudo_2086 0.427799382 0.607674054 0.360245609
0.801871795
pseudo_2087 0.344065784 0.013555302 0.262182595
0.491370099
pseudo_2088 0.167116087 1.634949984 0.281966242
0.766360793
pseudo_2089 0.529978261 -0.380746914
0.946095346 -0.176188693
pseudo_209 0.123892839 1.257531499
0.078435187 -0.813008757
pseudo_2090 0.240862031 -0.079758872
0.081669656 -0.305368422
pseudo_2091 0.501704379 -0.48506048 0.372688715
0.200359632
pseudo_2092 0.069833468 -2.784171449
0.736500301 -0.07301858
pseudo_2093 0.356972007 0.034418946
0.022491291 -0.614224594
pseudo_2094 0.89769155 -0.116672599 0.807030096
0.027669048
pseudo_2095 0.623246768 0.880433895 0.506193914
0.32463797
pseudo_2096 0.480062442 -0.733375485 0.118821001
0.12830042
pseudo_2097 0.183279842 -0.071886537
0.305312739 -0.032113063
pseudo_2098 0.206970154 1.239426651
0.336152618 -0.564201015
pseudo_2099 0.560092111 -2.2783161
0.414711642 -0.942931118

pseudo_21 0.89769155 −2.01930284
0.579726398 −0.223370365
pseudo_210 0.379008922 −1.747285796 0.094004703
0.674170644
pseudo_2100 0.805819688 0.490531871
0.234341661 −0.640391759
pseudo_2101 0.885965391 −0.41272917 0.605888732
0.205666522
pseudo_2102 0.864392024 −0.37011942
0.371014418 −0.30014002
pseudo_2103 0.642161592 0.385551939
0.394878476 −0.214339195
pseudo_2104 0.166157547 −2.469611537
0.646085339 −0.488913756
pseudo_2105 0.551180307 −1.088416933
0.848440681 −0.092658232
pseudo_2106 0.373947507 −0.476455346 0.389688104
0.632025544
pseudo_2107 0.934900969 −0.999759702
0.17970405 −0.257522484
pseudo_2108 0.755436274 0.487282729 0.245596991
0.672222196
pseudo_2109 0.992204499 0.865218469 0.414711642
0.259962262
pseudo_211 0.853955625 −2.211104534 0.315754097
0.138389465
pseudo_2110 0.6007649 0.688411384
0.613880724 −0.085067143
pseudo_2111 0.87608421 0.242420348
0.456100288 −0.316414724
pseudo_2112 0.895217987 0.305334588
0.352906442 −0.123346886
pseudo_2113 0.996054305 0.574513751 0.977239862
0.338017342
pseudo_2114 0.582404247 0.002393046 0.56009211
0.220299678
pseudo_2115 0.39923556 −0.306755043
0.741219826 −0.035699442
pseudo_2116 0.49921933 0.430632622
0.473767037 −0.289171738
pseudo_2117 0.20346385 0.95373035 0.148738121
0.871823592
pseudo_2118 0.12049321 1.82030646 0.691605731
0.031265955
pseudo_2119 0.090082385 −0.923803836
0.651707795 −0.091270711
pseudo_212 0.607853615 −0.836263722 0.021274851
0.870992771
pseudo_2120 0.819159155 −0.266263765 0.448576902
0.26149121

pseudo_2121 0.395747586 0.215704879 0.85579554
0.19965031
pseudo_2122 0.317264642 1.650399744
0.500709572 -0.322955813
pseudo_2123 0.097745753 -1.79956204
0.930550951 -0.256450639
pseudo_2124 0.265494703 0.841745737 0.89117494
0.064825268
pseudo_2125 0.992828121 -5.048914626
0.130120905 -0.120172169
pseudo_2126 0.88224904 0.765448109 0.078567843
0.764144523
pseudo_2127 0.225245317 -1.783816879 0.551180305
0.270521152
pseudo_2128 0.703750429 0.3817294
0.542338797 -0.695796386
pseudo_2129 0.207251571 -1.544861054
0.902641636 -0.010712784
pseudo_213 0.187954403 1.214655626 0.978486682
0.308828887
pseudo_2130 0.439272519 -0.581739474
0.105584257 -0.14566327
pseudo_2131 0.811876329 0.44331399 0.079100295
0.886433867
pseudo_2132 0.911933107 -0.610513821 0.254609399
0.122672206
pseudo_2133 0.158868602 -1.372972394 0.271269671
0.509516238
pseudo_2134 0.046717107 -1.458148228 0.688725651
0.44476673
pseudo_2135 0.305312741 0.842300328
0.238986164 -1.289015213
pseudo_2136 0.663579307 0.101184532 0.112844789
0.928870739
pseudo_2137 0.06652422 0.665956082
0.424618444 -0.703536251
pseudo_2138 0.173329596 -0.437334529
0.695430454 -0.071691851
pseudo_2139 0.663012048 -1.950517739
0.443445708 -0.411364661
pseudo_214 0.272292508 0.730021767 0.047674543
0.826880905
pseudo_2140 0.647769975 0.380615409 0.591011927
0.232671626
pseudo_2141 0.428254931 1.033214577
0.516751123 -0.509325755
pseudo_2142 0.767347144 -0.162411242
0.495256891 -0.107849381
pseudo_2143 0.784715264 0.245609787
0.921857266 -0.079561176

pseudo_2144 0.651144652 1.900859446
0.584550672 -0.044116262
pseudo_2145 0.84415639 0.258951682 0.589932773
0.447924264
pseudo_2146 0.144589544 -1.378136981 0.954809562
0.7896218
pseudo_2147 0.672681501 -0.51053084
0.806424833 -0.036537962
pseudo_2148 0.876700361 1.093660985
0.538202696 -0.208173101
pseudo_2149 0.663012048 0.844990662
0.274346085 -0.139258063
pseudo_215 0.837395199 0.100988784
0.781199445 -0.269589025
pseudo_2150 0.838043993 -0.59644191 0.477151216
0.168311755
pseudo_2151 0.462264939 -1.878208331
0.208097484 -0.393642779
pseudo_2152 0.837433284 0.707782982
0.410254103 -0.436000854
pseudo_2153 0.871773275 -0.083597898 0.544412774
0.314000563
pseudo_2154 0.712868401 -0.670337058
0.863234424 -0.201810501
pseudo_2155 0.985345049 0.687824571
0.816729779 -0.35935943
pseudo_2156 0.133932189 -2.098273164 0.151155391
0.303455501
pseudo_2157 0.291434753 1.299557944 0.292567101
0.286290169
pseudo_2158 0.735911056 0.615539472
0.893981586 -0.266888457
pseudo_2159 0.908215001 -0.026127582
0.93303643 -0.137996673
pseudo_216 0.461789091 -0.519853851 0.867466145
0.702075507
pseudo_2160 0.194591985 -2.907798703 0.223456721
0.268830385
pseudo_2161 0.218151962 -2.024949366
0.735911055 -0.300247608
pseudo_2162 0.595879318 -0.2953252
0.781712833 -0.14571504
pseudo_2163 0.816729779 -0.533830562 0.67667894
0.129324138
pseudo_2164 0.139021756 -1.386319324 0.331182439
0.560381319
pseudo_2165 0.324888312 -1.130264352 0.050738763
0.694990944
pseudo_2166 0.518268556 1.325637541
0.65895202 -0.298014912

pseudo_2167 0.18506575 -0.603272233 0.262674816
0.090530292
pseudo_2168 0.12639434 -0.654831589 0.019334093
0.887723374
pseudo_2169 0.242118355 0.23495364
0.107790849 -0.517916124
pseudo_217 0.706072412 -0.06769
0.19593989 -0.369836989
pseudo_2170 0.41964786 0.491103457 0.7721271
0.310915105
pseudo_2171 0.589393538 -1.019168672 0.623799633
0.280406635
pseudo_2172 0.622694114 0.362993875 0.632952337
0.352063365
pseudo_2173 0.311250854 1.309175897 0.11498994
0.724350563
pseudo_2174 0.976616473 0.007953182 0.121806506
0.303551231
pseudo_2175 0.903879757 -0.474877211 0.630450488
0.070811618
pseudo_2176 0.171514941 -0.644455143 0.114675035
0.569351873
pseudo_2177 0.228853125 2.261845382
0.919375024 -0.041512793
pseudo_2178 0.319919475 -0.309850579 0.473284662
0.111745614
pseudo_2179 0.769736029 -0.301310759
0.193786512 -0.21541687
pseudo_218 0.489344877 0.219184665
0.944228817 -0.9147844
pseudo_2180 0.345262934 -1.170422969 0.421902962
0.934777311
pseudo_2181 0.727091176 0.088616774 0.635458394
0.283094819
pseudo_2182 0.060828769 -3.907258034
0.450451115 -0.079873488
pseudo_2183 0.898345704 0.298059884 0.09237087
0.739651478
pseudo_2184 0.754842227 0.592308679 0.033533156
0.34683979
pseudo_2185 0.283367004 -1.523424367
0.426889128 -0.425617196
pseudo_2186 0.766153529 0.868736501 0.387108552
0.346300528
pseudo_2187 0.530490411 0.226457175
0.217276706 -0.482219431
pseudo_2188 0.430994149 -1.353094443 0.279526229
0.215536318
pseudo_2189 0.871016131 0.786799713 0.4758858
0.297172107

pseudo_219 0.154522358 1.994284727 0.643281647
0.044316037
pseudo_2190 0.537171151 -1.263016417 0.522834633
0.361404092
pseudo_2191 0.331072377 1.66355294 0.279873921
0.060018774
pseudo_2192 0.912243037 -2.219573537
0.253958126 -0.309533049
pseudo_2193 0.104620404 -0.328415513 0.097924718
0.658477067
pseudo_2194 0.249753115 0.71882424
0.331072375 -0.224949936
pseudo_2195 0.344895697 -0.463524467
0.412639554 -0.276197792
pseudo_2196 0.202505155 1.920655056 0.737089703
0.097857677
pseudo_2197 0.823256176 -0.16617508
0.730614865 -0.434306266
pseudo_2198 0.498723106 -3.524818037
0.334975824 -0.313834612
pseudo_2199 0.071784365 0.021729882 0.721816466
0.178213456
pseudo_22 0.890891717 0.540305203
0.509198638 -0.400281929
pseudo_220 0.315377203 0.878488652 0.819766771
0.101621988
pseudo_2200 0.908834548 -0.696963044
0.685850076 -0.188554611
pseudo_2201 0.230064821 1.486329041 0.960414478
0.361973978
pseudo_2202 0.877316587 -1.275161745 0.661311428
0.210544872
pseudo_2203 0.388827095 -0.081489667
0.024753347 -1.256076222
pseudo_2204 0.264852865 -1.558909804
0.600221163 -0.014150169
pseudo_2205 0.090082385 -1.54367431 0.382832508
0.227204412
pseudo_2206 0.787720902 0.309850047
0.464648279 -0.215256014
pseudo_2207 0.028373762 -0.080052069 0.517256677
0.65087016
pseudo_2208 0.150488758 -0.894638244 0.501704377
0.431785736
pseudo_2209 0.719476374 -0.21929164 0.51372318
0.373798936
pseudo_221 0.744174494 -0.543179195
0.457045676 -0.20840362
pseudo_2210 0.547010779 -0.591964382 0.421451374
0.294241875

pseudo_2211 0.222269986 -1.29131522
0.29799646 -0.348277347
pseudo_2212 0.421902964 1.28614 0.94485096 0.276794381
pseudo_2213 0.996569936 1.33204412 0.961037372
0.28657529
pseudo_2214 0.698536085 -0.725166028
0.932414998 -0.145155388
pseudo_2215 0.554317675 -1.782670736
0.270589245 -0.48383341
pseudo_2216 0.299996662 1.330942445 0.778114209
0.038725623
pseudo_2217 0.260854461 -1.571141092 0.471357868
0.218964238
pseudo_2218 0.274003091 0.448897203
0.606214448 -0.477766075
pseudo_2219 0.288306738 -0.195812912
0.870542272 -0.004915218
pseudo_222 0.137414964 -2.609967503
0.068044233 -0.218620938
pseudo_2220 0.748909788 -0.096173727 0.039926085
0.405877056
pseudo_2221 0.14266303 -1.78513041
0.611685998 -0.191322806
pseudo_2222 0.692182286 0.723323725 0.30054372
0.813933028
pseudo_2223 0.689877146 -2.508015702
0.397053412 -0.736436342
pseudo_2224 0.905737376 0.462616327 0.30017894
0.347261962
pseudo_2225 0.975993098 1.140957856
0.49476277 -0.30702589
pseudo_2226 0.954186927 0.507059501
0.127562062 -1.028938338
pseudo_2227 0.767347144 -0.299215534 0.563781867
0.597084105
pseudo_2228 0.520802719 -2.090456364 0.502202171
0.173282131
pseudo_2229 0.957300377 0.238804688 0.059337973
0.708199651
pseudo_223 0.775717764 -0.403906576 0.66585027
0.208939783
pseudo_2230 0.446707126 -0.5447037 0.600221163
0.484276744
pseudo_2231 0.098221636 -0.284176679 0.061694496
0.66342251
pseudo_2232 0.584013722 0.084445005 0.629339911
0.344678121
pseudo_2233 0.326427234 -2.715078198
0.982850945 -0.851670911
pseudo_2234 0.070559892 0.529061541

0.906356696 -0.177876046
pseudo_2235 0.634901132 -0.504786306
0.439272517 -0.406355619
pseudo_2236 0.228853125 -3.262479588
0.356156541 -0.026073122
pseudo_2237 0.743583258 -0.258227808
0.171725955 -0.833240739
pseudo_2238 0.9881633 0.003067413
0.133815371 -0.834180055
pseudo_2239 0.986592156 -0.436775538
0.668124302 -0.418360784
pseudo_224 0.819409812 0.555692635 0.359397712
0.22340381
pseudo_2240 0.871157735 -0.954901018
0.863162946 -0.625371271
pseudo_2241 0.811270152 0.180845886 0.470636434
0.756193644
pseudo_2242 0.73107755 1.547488791 0.430251162
0.135250341
pseudo_2243 0.932414998 0.754469192
0.072403098 -0.25951106
pseudo_2244 0.160726036 1.721665346 0.072155082
0.752475086
pseudo_2245 0.781199446 -0.951149946
0.148043838 -0.55265399
pseudo_2246 0.632674148 -0.541306854 0.113022336
0.55774467
pseudo_2247 0.813089022 0.465063775
0.624906001 -0.782659818
pseudo_2248 0.415606575 -0.640886455
0.70259047 -0.153671101
pseudo_2249 0.213800953 0.339307865
0.225544405 -0.206061579
pseudo_225 0.441415413 -3.086838197 0.572921146
0.466668438
pseudo_2250 0.6056685 0.200615385
0.152719619 -0.2405121
pseudo_2251 0.274346087 -1.943166099 0.713637673
0.543976416
pseudo_2252 0.181485411 0.561480251 0.838960238
0.336873368
pseudo_2253 0.542856924 0.542338466 0.514437109
0.319645506
pseudo_2254 0.312747205 -2.499842343 0.129724664
0.773802412
pseudo_2255 0.012168342 1.508566369 0.874262242
0.005388397
pseudo_2256 0.436963858 -0.826620854
0.714220795 -0.008051334
pseudo_2257 0.584550673 -0.221148234 0.578122467

0.510274725
pseudo_2258 0.869926889 -0.43526796 0.562726458
0.361684737
pseudo_2259 0.25591543 -0.80782545
0.876084209 -0.548610718
pseudo_226 0.281267628 0.801491742 0.374788157
0.198066691
pseudo_2260 0.983474456 -1.881320553
0.664714403 -0.118286089
pseudo_2261 0.557988945 -0.221084863 0.522834632
0.416280297
pseudo_2262 0.11789989 1.405411846
0.983560699 -0.431830971
pseudo_2263 0.497236009 0.380568521
0.492296123 -0.229740925
pseudo_2264 0.684701118 -1.745479912 0.413371383
0.054961305
pseudo_2265 0.631561902 -0.64563108
0.455156002 -0.365027463
pseudo_2266 0.224051789 -1.011595471 0.1588686
0.231342983
pseudo_2267 0.344065783 0.135175074
0.069954108 -1.199955407
pseudo_2268 0.738858835 0.177975915 0.346462734
0.268052521
pseudo_2269 0.227573639 -0.252914418 0.220974511
0.075713931
pseudo_227 0.715971164 0.967005241
0.705491657 -0.366288524
pseudo_2270 0.613392933 0.408542077
0.463228923 -0.096020935
pseudo_2271 0.024954661 1.778179152
0.196480964 -0.251491263
pseudo_2272 0.802191261 -0.343258429 0.175930668
0.477851042
pseudo_2273 0.437886483 0.974132461
0.567483301 -0.154984497
pseudo_2274 0.504912708 -1.168916121
0.880760764 -0.178350232
pseudo_2275 0.394878478 -0.860553614 0.58401372
0.325270669
pseudo_2276 0.258212167 -0.941960294
0.180465892 -1.01022766
pseudo_2277 0.306790176 1.230427462 0.296547377
0.313164448
pseudo_2278 0.359835383 0.791189861
0.328744485 -0.651027924
pseudo_2279 0.827066507 0.168964093 0.295824606
0.611533548
pseudo_228 0.504695059 -0.499343529

0.180720371 -0.497136948
pseudo_2280 0.404502072 0.477620122 0.108305212
0.841245722
pseudo_2281 0.990957268 0.028477319
0.790729703 -0.250482231
pseudo_2282 0.346863259 0.31329625
0.618280553 -1.01572445
pseudo_2283 0.978486682 -2.865978045
0.096329152 -0.622618826
pseudo_2284 0.024010899 -1.645802197 0.037712888
0.486304404
pseudo_2285 0.887918407 0.932761041 0.704330665
0.200644389
pseudo_2286 0.052358666 -1.349790771
0.686316189 -0.342442387
pseudo_2287 0.11863633 0.579071243 0.703170362
0.363163227
pseudo_2288 0.781712834 -0.566847424 0.935522561
0.259563819
pseudo_2289 0.608400443 0.538274207
0.668124302 -0.258851551
pseudo_229 0.647769975 -2.479521608 0.692182283
0.322740934
pseudo_2290 0.021274852 1.363908581 0.064578816
0.274639539
pseudo_2291 0.728852305 0.596579559 0.872388892
0.010081529
pseudo_2292 0.567748134 -0.795879444
0.970383429 -0.103414933
pseudo_2293 0.509700334 0.557851217
0.218444274 -0.218174799
pseudo_2294 0.712471931 1.117285481 0.186647174
0.247691598
pseudo_2295 0.301639834 -1.325863026 0.297271328
0.56598461
pseudo_2296 0.172809665 -1.478474668
0.945460201 -0.020793663
pseudo_2297 0.224051789 0.895209928 0.193518566
0.692137246
pseudo_2298 0.634344075 0.696631672
0.594254823 -1.409466317
pseudo_2299 0.175183135 0.536534223
0.843544721 -0.163688167
pseudo_23 0.834381215 0.836565546
0.974746392 -0.314660042
pseudo_230 0.065461561 0.377599519
0.611079579 -0.088455862
pseudo_2300 0.834381215 0.841165492
0.840793384 -0.086338097
pseudo_2301 0.293304204 -1.407426901 0.363537995

0.546383134
pseudo_2302 0.518268556 0.345705649 0.28442066
0.723102502
pseudo_2303 0.019213076 1.784564033
0.61058994 -0.687957138
pseudo_2304 0.521310316 -0.405655534 0.133526979
0.647648628
pseudo_2305 0.385394652 0.331163234
0.398361835 -0.88843556
pseudo_2306 0.761980261 -3.426877855 0.844156389
0.061347676
pseudo_2307 0.009319929 1.543339481 0.846604 0.066411415
pseudo_2308 0.744174494 -1.499416052 0.506193914
0.295172637
pseudo_2309 0.084814818 1.374870226
0.443373819 -0.071532802
pseudo_231 0.717138907 -2.723738276
0.271610323 -0.017138684
pseudo_2310 0.296185846 -0.858586671
0.854568842 -0.381805413
pseudo_2311 0.903260667 0.414460486 0.794947262
0.480830899
pseudo_2312 0.373108022 0.611469958 0.270249469
0.600469319
pseudo_2313 0.572259317 -0.38332685 0.758408669
0.205412172
pseudo_2314 0.331850704 0.354487855
0.048292369 -0.428925574
pseudo_2315 0.145020354 0.422340403
0.059232658 -0.675490368
pseudo_2316 0.57332324 -0.018119573
0.253632924 -0.707320534
pseudo_2317 0.072031337 0.341091517
0.438810225 -0.322336882
pseudo_2318 0.215823569 1.462556653
0.335760058 -0.089237022
pseudo_2319 0.125042534 -3.994872895 0.120306503
0.219966825
pseudo_232 0.061585727 1.23565526
0.436042352 -0.22669703
pseudo_2320 0.279526231 0.849679622
0.163306737 -0.390877182
pseudo_2321 0.201952002 1.032700551
0.771529128 -0.389129188
pseudo_2322 0.467996415 -0.357525628 0.416951107
0.597429083
pseudo_2323 0.395312888 0.16001648
0.856715792 -0.273068312
pseudo_2324 0.823414729 -0.787787045 0.361066946
0.187203275

pseudo_2325 0.231585847 1.39624979
0.159563225 -0.698135376
pseudo_2326 0.95858864 0.428524758
0.874262242 -0.068329717
pseudo_2327 0.028942908 2.210658569
0.629617476 -0.381835857
pseudo_2328 0.989086465 0.508859435 0.868696358
0.315471557
pseudo_2329 0.564309934 0.511097327
0.866851159 -0.276538329
pseudo_233 0.784715264 0.904054757
0.025618752 -1.291815041
pseudo_2330 0.577054342 -0.730360412 0.324120621
0.265920482
pseudo_2331 0.733261373 0.115246309 0.120680141
1.11085198
pseudo_2332 0.161660884 -0.478850167
0.445773901 -0.56757747
pseudo_2333 0.489344877 0.928570721 0.578656878
0.185301288
pseudo_2334 0.622141673 1.058376539 0.064465858
0.716497423
pseudo_2335 0.202782144 1.084579714 0.032695534
0.270785779
pseudo_2336 0.805214657 -0.142783921 0.134338345
0.805355679
pseudo_2337 0.471839163 -0.489929155
0.980980476 -0.208962271
pseudo_2338 0.293702403 0.67732389 0.634384367
0.453301917
pseudo_2339 0.954809562 1.19374854 0.509700332
0.085995753
pseudo_234 0.500212564 -0.933782467
0.431909473 -0.803440327
pseudo_2340 0.677250753 0.368673837
0.261517944 -0.226419517
pseudo_2341 0.91441292 -0.717675454 0.422354834
0.587595945
pseudo_2342 0.934900969 0.607994518 0.701431204
0.161847849
pseudo_2343 0.386251022 -0.186326354 0.673251999
0.330437122
pseudo_2344 0.026723814 2.130678553
0.13639939 -0.208644407
pseudo_2345 0.680112591 -0.13250876
0.839876703 -0.068320889
pseudo_2346 0.092935462 1.564317132
0.145452144 -0.689380052
pseudo_2347 0.036998739 -1.65028942 0.107790849
0.170604021

pseudo_2348	0.718307311	-1.570434463	
0.19165056	-0.319697416		
pseudo_2349	0.606760618	-0.227848466	
0.037712888	-0.309927794		
pseudo_235	0.773323451	-1.042185716	
0.12199503	-0.440200176		
pseudo_2350	0.457518784	1.461902369	
0.116437773	-1.176442152		
pseudo_2351	0.95294174	-0.265998742	
0.739448852	-0.097057101		
pseudo_2352	0.424165158	0.306810228	0.23097658
0.635126419			
pseudo_2353	0.905118112	0.294682331	
0.475215781	-0.243183711		
pseudo_2354	0.483471074	0.75911571	0.474249682
0.094143855			
pseudo_2355	0.40230266	1.410182941	
0.473284662	-0.675325664		
pseudo_2356	0.367264354	0.883330111	0.844768152
0.205847359			
pseudo_2357	0.81248262	0.203488374	0.681831911
0.421576826			
pseudo_2358	0.64328165	1.054245038	0.903260667
0.087339607			
pseudo_2359	0.827675502	-0.149532665	
0.221087767	-0.207889147		
pseudo_236	0.489836089	-0.063313993	0.091125202
0.777558684			
pseudo_2360	0.5376868	0.01341696	0.681545242
0.123459459			
pseudo_2361	0.151378103	1.670348559	0.303840031
0.244687831			
pseudo_2362	0.376894897	-0.691855384	0.741810456
0.051169144			
pseudo_2363	0.376051327	-0.284349704	
0.225245315	-0.385673616		
pseudo_2364	0.379008922	-0.450445371	0.374788157
0.187341659			
pseudo_2365	0.678394932	0.755287459	
0.42371215	-0.150858042		
pseudo_2366	0.337725811	-0.066887714	0.008465465
0.667793755			
pseudo_2367	0.450920363	1.528829684	0.937387574
0.138782581			
pseudo_2368	0.753060958	-0.704828226	
0.558514375	-0.136171585		
pseudo_2369	0.21208863	1.030239824	0.048667543
0.538316235			
pseudo_237	0.64048303	1.017195243	
0.83621216	-0.251417675		

pseudo_2370	0.962906197	0.325755364	0.733555626
0.323519466			
pseudo_2371	0.908834548	0.883877149	0.226143436
0.454142687			
pseudo_2372	0.932414998	0.955049737	0.311998436
0.340838803			
pseudo_2373	0.726504453	0.328197679	
0.287951984	-0.452360702		
pseudo_2374	0.078700683	1.39298731	
0.200849002	-0.276564902		
pseudo_2375	0.283016373	0.607825676	0.630450488
0.393500566			
pseudo_2376	0.700272634	-0.240817369	0.714804085
0.029467421			
pseudo_2377	0.350682918	0.793830454	0.086149727
0.646669812			
pseudo_2378	0.136833781	0.650983323	
0.898345704	-0.118316099		
pseudo_2379	0.91069352	0.736343353	0.96166029
0.95440812			
pseudo_238	0.921857266	-0.490101733	0.032504802
0.730861014			
pseudo_2380	0.792536487	0.626500473	0.721816466
0.32268016			
pseudo_2381	0.532541522	-0.340962393	0.760193825
0.341534695			
pseudo_2382	0.766750267	-0.580555107	0.176680578
0.578494875			
pseudo_2383	0.921236633	0.6854713	
0.294742663	-0.446034399		
pseudo_2384	0.928066149	-0.24708825	0.996569936
0.286177454			
pseudo_2385	0.739773229	0.652550789	0.329135861
0.064521612			
pseudo_2386	0.440661071	-0.178852687	0.123511444
0.130167347			
pseudo_2387	0.319539326	0.583802564	0.659046655
0.345152908			
pseudo_2388	0.456486214	0.214554645	
0.427846024	-0.039153502		
pseudo_2389	0.886568692	-0.575870476	
0.312747203	-0.028299025		
pseudo_239	0.635458396	0.102606376	0.501704377
0.29584858			
pseudo_2390	0.682979045	0.581587243	
0.248148827	-0.034887607		
pseudo_2391	0.192983493	0.779261565	
0.290084914	-0.170529653		
pseudo_2392	0.171848541	-3.571505278	
0.270589245	-1.300035971		

pseudo_2393	0.45610029	0.389622148	0.580261505
0.307309439			
pseudo_2394	0.141389674	1.823787981	
0.805819687	-0.503316981		
pseudo_2395	0.345462716	-1.164018816	0.521818166
0.204817588			
pseudo_2396	0.278831729	-0.284763036	0.390550273
0.32816841			
pseudo_2397	0.673632737	-0.346388419	0.224099641
0.816673308			
pseudo_2398	0.619193145	1.268305241	0.93824564
0.682921546			
pseudo_2399	0.269909993	0.667864099	0.48006244
0.211774229			
pseudo_24	0.365191226	0.477573657	
0.797359998	-0.15382477		
pseudo_240	0.084728863	-0.850021132	
0.449513455	-0.087284724		
pseudo_2400	0.539235237	0.248622453	
0.634901131	-0.185999402		
pseudo_2401	0.236191732	1.214320051	0.115350564
0.255403958			
pseudo_2402	0.965398278	-0.37478552	0.487382682
0.466386347			
pseudo_2403	0.986592156	0.464249874	0.600221163
0.030496002			
pseudo_2404	0.826457615	-3.531667395	0.905737375
0.13489923			
pseudo_2405	0.727361307	0.289013549	
0.399600406	-0.1511106		
pseudo_2406	0.421451376	-0.125219831	
0.059232658	-0.481320935		
pseudo_2407	0.129176557	0.74199212	0.40144782
0.112963973			
pseudo_2408	0.228248984	0.739260123	0.966021351
0.237333784			
pseudo_2409	0.736500302	0.546014073	0.412033692
0.496693717			
pseudo_241	0.713637674	-0.610866466	0.886568691
0.17470341			
pseudo_2410	0.959791608	0.17693372	
0.747132931	-0.197206572		
pseudo_2411	0.047150281	2.776672239	
0.834381215	-0.224931961		
pseudo_2412	0.696223139	-1.592518442	0.939252931
0.247812967			
pseudo_2413	0.753060958	-0.959609861	0.565895549
0.233967211			
pseudo_2414	0.666418493	2.01239254	
0.493775319	-0.063421164		

pseudo_2415 0.230368455 -1.569758122 0.169045619
0.60654711
pseudo_2416 0.258541435 -2.301948212
0.940496688 -0.620914657
pseudo_2417 0.415606575 0.733869352 0.460838213
0.267361668
pseudo_2418 0.923098674 -0.328426786
0.967890696 -0.123033931
pseudo_2419 0.788924046 -0.63358605 0.753654568
0.294350177
pseudo_242 0.789525806 -0.222811482 0.830722027
0.266309918
pseudo_2420 0.232196253 -1.604815669 0.564309933
0.774250726
pseudo_2421 0.372948989 0.476469585 0.187813207
0.296690345
pseudo_2422 0.519281456 0.280885784 0.07936762
0.575158926
pseudo_2423 0.411143328 -1.233545002
0.022583756 -0.631933352
pseudo_2424 0.240548669 -1.416771714
0.390981792 -0.131856529
pseudo_2425 0.555889629 0.547359085
0.726504451 -0.188988165
pseudo_2426 0.434662201 -0.211763624
0.967890696 -0.287551187
pseudo_2427 0.079501558 1.117530805
0.164965193 -1.026203277
pseudo_2428 0.375208924 -0.053622713 0.166876062
0.554921779
pseudo_2429 0.418747805 -0.935033702
0.981603955 -0.195486844
pseudo_243 0.818551648 -0.381449901 0.288306736
0.134984376
pseudo_2430 0.527421284 0.817877883
0.7721271 -0.02476137
pseudo_2431 0.359015808 -0.930471976 0.655090824
0.03090611
pseudo_2432 0.612782928 0.359149163 0.759598631
0.466674173
pseudo_2433 0.497731445 0.469917505 0.077118626
0.732181715
pseudo_2434 0.650581707 1.32636447 0.638806303
0.355692302
pseudo_2435 0.270589247 -0.144902731 0.750687977
0.793577931
pseudo_2436 0.430079949 -0.095637689 0.349675167
0.266949867
pseudo_2437 0.763767965 0.099100784 0.976616473
0.192853112

pseudo_2438	0.331072377	1.673990456	0.564838234
0.260274082			
pseudo_2439	0.311998438	−1.576446949	
0.504695057	−0.451438834		
pseudo_244	0.120867302	−1.67168949	
0.842933146	−0.255191995		
pseudo_2440	0.743583258	−0.805366456	
0.48395909	−0.164977802		
pseudo_2441	0.761384641	0.303112354	
0.401863637	−0.098741624		
pseudo_2442	0.253632926	−1.623420213	0.766750266
0.295015246			
pseudo_2443	0.191710061	−0.060982574	0.16515006
0.237787158			
pseudo_2444	0.311250854	0.535532714	0.579726398
0.121159535			
pseudo_2445	0.529466362	−0.282218168	0.87978223
0.192420233			
pseudo_2446	0.738858835	−0.280539709	
0.608400441	−0.299788953		
pseudo_2447	0.633230583	−0.596998024	
0.311998436	−0.646839349		
pseudo_2448	0.343269159	−2.389475681	0.924340269
0.66549815			
pseudo_2449	0.637131424	−0.870137377	0.725917889
0.186315797			
pseudo_245	0.894599755	0.385322053	
0.0249042	−0.641707435		
pseudo_2450	0.371850984	0.026591483	0.239610304
0.34691901			
pseudo_2451	0.094774471	0.628219262	0.592632358
0.37591194			
pseudo_2452	0.511709694	−0.399644509	
0.077643073	−0.733713159		
pseudo_2453	0.027264345	−1.471728646	0.613331717
0.073278064			
pseudo_2454	0.977239862	−0.009443216	0.104912564
0.766237666			
pseudo_2455	0.064353064	1.265100742	0.341679442
0.301610324			
pseudo_2456	0.007229808	2.691390196	
0.12629025	−0.718366075		
pseudo_2457	0.188216657	1.215509803	
0.499219328	−0.162132094		
pseudo_2458	0.384555235	−1.086498481	
0.749127053	−0.040589608		
pseudo_2459	0.874236208	−0.859779658	
0.004200248	−0.848039616		
pseudo_246	0.68642483	−0.203422551	0.769736028
0.375628363			

pseudo_2460	0.114989942	1.607407003	
0.302005794	-0.960725666		
pseudo_2461	0.810058137	0.26349828	0.930550951
0.471853425			
pseudo_2462	0.328744488	2.838465915	0.538718841
0.209625945			
pseudo_2463	0.546490688	-0.285636177	0.013736292
0.679267543			
pseudo_2464	0.164252882	0.592082567	
0.699114758	-0.247093478		
pseudo_2465	0.172290902	1.664721102	0.786265922
0.063620894			
pseudo_2466	0.545451241	0.392097634	0.454684271
0.310814579			
pseudo_2467	0.838654799	0.066271789	
0.269570802	-0.021261422		
pseudo_2468	0.990957268	-0.403642446	
0.946717588	-0.161980252		
pseudo_2469	0.117349918	1.691480878	0.367264352
0.029032672			
pseudo_247	0.922477946	0.496845812	
0.775717763	-0.326138725		
pseudo_2470	0.736663742	0.337128984	
0.791976535	-0.003903597		
pseudo_2471	0.41964786	1.037958527	
0.4337435	-0.382315124		
pseudo_2472	0.046803473	-0.539938546	
0.169287982	-0.636438738		
pseudo_2473	0.416054468	-2.597935824	0.474866322
0.232756645			
pseudo_2474	0.940496688	-0.376129349	
0.261850124	-0.387658379		
pseudo_2475	0.16977349	-1.028700719	0.379432599
0.313606484			
pseudo_2476	0.51221268	-0.67218538	0.803400266
0.314841044			
pseudo_2477	0.322588794	-1.664282005	
0.770333592	-0.130647879		
pseudo_2478	0.622141673	-0.931486912	
0.04715028	-0.587927025		
pseudo_2479	0.952319189	0.515918863	0.601308856
0.294784326			
pseudo_248	0.599677652	0.807868529	0.174190111
0.768844274			
pseudo_2480	0.613331718	-2.303923155	
0.933657902	-0.238296485		
pseudo_2481	0.339303724	0.352579135	0.465603527
0.085227045			
pseudo_2482	0.055344173	-1.400182099	
0.902022664	-0.126618381		

pseudo_2483	0.915033005	-2.883343948	0.461789089
0.201893005			
pseudo_2484	0.6056685	0.450828375	0.746540944
0.314101852			
pseudo_2485	0.822806469	-0.229981677	0.575987144
0.493938029			
pseudo_2486	0.020158033	-1.703604671	
0.596963435	-0.309985254		
pseudo_2487	0.328744488	-0.495431029	
0.220204066	-0.281931892		
pseudo_2488	0.66131143	-0.758222785	0.418747804
0.447465035			
pseudo_2489	0.250921388	1.119492801	
0.847065934	-0.453641774		
pseudo_249	0.145884921	-0.628510351	0.957923146
0.177685992			
pseudo_2490	0.030468884	-1.042279257	
0.607853614	-0.191311296		
pseudo_2491	0.893981586	-0.309491373	0.977239862
0.325651103			
pseudo_2492	0.305312741	-1.425326234	0.199202758
0.519894522			
pseudo_2493	0.271610325	1.701546637	
0.575453896	-0.135215245		
pseudo_2494	0.47135787	0.599890389	0.71830731
0.314939954			
pseudo_2495	0.935522562	0.923817163	0.134541783
0.675976501			
pseudo_2496	0.519788289	0.698158764	0.723573247
0.622237168			
pseudo_2497	0.483959091	-0.43460605	
0.495256891	-0.250207819		
pseudo_2498	0.994699014	0.301178964	
0.387108552	-0.045446769		
pseudo_2499	0.143089432	-0.189018499	0.790127692
0.429247686			
pseudo_25	0.702880395	-0.062627443	0.363537995
0.348871925			
pseudo_250	0.713054719	0.253259865	0.627675617
0.283497762			
pseudo_2500	0.503198545	0.041560125	
0.868696358	-0.536841663		
pseudo_2501	0.747725069	-1.023904465	0.283717927
0.352557497			
pseudo_2502	0.97225317	-2.243429159	0.844768152
0.005424556			
pseudo_2503	0.621037428	1.586585737	0.906976075
0.10575781			
pseudo_2504	0.349473839	0.725378399	0.934279415
0.196070801			

pseudo_2505 0.058499789 2.184882238
0.084587823 -0.669222776
pseudo_2506 0.139496041 1.894643985
0.845991957 -0.751974188
pseudo_2507 0.926203007 0.92239917
0.589393537 -0.564549696
pseudo_2508 0.077774639 1.603345078 0.310504449
0.059575057
pseudo_2509 0.686999765 0.305600481
0.449982147 -0.279446719
pseudo_251 0.68642483 -0.594357311
0.075177603 -0.006809721
pseudo_2510 0.430994149 -0.221713698 0.159795276
0.606553318
pseudo_2511 0.992828121 0.216539476 0.387537753
0.125183003
pseudo_2512 0.627675619 0.934114045
0.436963856 -0.288235007
pseudo_2513 0.738858835 -0.732304884
0.871157734 -0.181121648
pseudo_2514 0.999342382 0.470071085
0.223158802 -0.138380425
pseudo_2515 0.475215783 0.558264883 0.260523153
0.166857238
pseudo_2516 0.273317979 -1.014851133 0.228248982
0.07290314
pseudo_2517 0.140756281 0.591190245 0.977863265
0.719876489
pseudo_2518 0.751281003 0.874337478
0.8149089 -0.04155373
pseudo_2519 0.17270847 -0.917050258 0.240235592
0.178274204
pseudo_252 0.282666036 0.957320164
0.598591294 -0.318304232
pseudo_2520 0.02011612 2.09044175 0.686424829
0.286508444
pseudo_2521 0.483959091 -0.317106424
0.329907101 -0.082515102
pseudo_2522 0.250719158 0.660637315 0.663579306
0.022156122
pseudo_2523 0.11445067 0.33486994
0.690453161 -0.330723728
pseudo_2524 0.175930669 -1.09066064
0.537686798 -0.498626559
pseudo_2525 0.911313286 0.539913492 0.942984634
0.208715269
pseudo_2526 0.561145133 0.149213697 0.402741965
0.297444622
pseudo_2527 0.84415639 -0.103055765
0.592632358 -0.217983854

pseudo_2528	0.269909993	1.831713705	0.221087767	0.681265112
pseudo_2529	0.662444982	0.298270703	0.594254823	0.080362447
pseudo_253	0.324120624	0.742337865	0.8149089	0.147407874
pseudo_2530	0.593713777	-0.597426609	0.447174153	-0.776202986
pseudo_2531	0.460363188	-1.920567107	0.158176266	-0.153059342
pseudo_2532	0.133122721	-2.341479189	0.216694608	-0.797529178
pseudo_2533	0.67239632	-0.792253733	0.260854458	-0.060901326
pseudo_2534	0.11863633	-3.878331991	0.254935471	0.315120515
pseudo_2535	0.757813903	0.31681688	0.980980476	0.03644466
pseudo_2536	0.574920883	-0.64824788	0.736500301	-0.307295294
pseudo_2537	0.170503706	0.528577279	0.38582269	0.36602708
pseudo_2538	0.560092111	-0.820346145	0.271951268	-0.195334526
pseudo_2539	0.719331514	0.617724969	0.929308464	-0.267024286
pseudo_254	0.910073808	2.56539488	0.955432225	-0.196178415
pseudo_2540	0.987215722	0.901154796	0.294022842	-0.402482342
pseudo_2541	0.992828121	-0.498847758	0.42371215	-0.119483378
pseudo_2542	0.141601292	0.873375668	0.748909787	0.269465646
pseudo_2543	0.887267325	-0.173787819	0.882865921	0.217329312
pseudo_2544	0.672681501	0.723214182	0.94920688	-0.120887081
pseudo_2545	0.445307708	-2.272288093	0.481521676	-1.481049894
pseudo_2546	0.214666108	1.749993514	0.824631567	-0.274817345
pseudo_2547	0.911933107	-0.67769415	0.762576021	-0.449922178
pseudo_2548	0.285124572	1.51525304	0.743583257	0.345015969
pseudo_2549	0.807938207	0.73020991	0.447641458	-0.404407048
pseudo_255	0.293663377	0.321915526	0.253958126	-0.219987845

pseudo_2550 0.681258621 -2.007804915
0.072527367 -1.046050793
pseudo_2551 0.741810457 -0.026570667 0.40450207
0.162801767
pseudo_2552 0.801586935 -0.025705241
0.90759551 -0.262773249
pseudo_2553 0.276410222 0.443254505 0.315377201
0.549140095
pseudo_2554 0.719476374 -0.431409591
0.019415141 -0.629564068
pseudo_2555 0.936765865 -1.123754469
0.953564319 -0.640722363
pseudo_2556 0.582136201 -0.184352223 0.261186056
0.176408405
pseudo_2557 0.229155622 -2.983607822 0.822198314
0.336351318
pseudo_2558 0.742992173 0.538258323 0.501704377
0.804524619
pseudo_2559 0.010916011 -1.677189019
0.208662816 -0.51205076
pseudo_256 0.02217025 -1.800726963 0.295463663
0.280426128
pseudo_2560 0.413817852 -0.40404238
0.2095129 -0.234435499
pseudo_2561 0.051203006 2.165796922 0.116984388
0.738996489
pseudo_2562 0.540785905 -0.556355571 0.338908801
0.321982727
pseudo_2563 0.39923556 0.25542964 0.846604 0.120901261
pseudo_2564 0.237430837 -0.802647793 0.177432868
0.578093233
pseudo_2565 0.174686097 0.819126761
0.528954713 -0.30879133
pseudo_2566 0.346863259 1.187574287
0.600221163 -0.149913065
pseudo_2567 0.755383603 0.729686421 0.455725773
0.401512777
pseudo_2568 0.62656714 -0.575476928
0.558514375 -0.133729593
pseudo_2569 0.706072412 0.11149837
0.165441359 -0.399351288
pseudo_257 0.641601868 0.31831328
0.145452144 -0.457471306
pseudo_2570 0.769736029 -0.07706479 0.42962327
0.173093344
pseudo_2571 0.962283232 0.019657284 0.580796843
0.258676412
pseudo_2572 0.030289354 1.809210345
0.079233866 -0.529040351
pseudo_2573 0.646646684 -0.699808214

0.045101467	-1.083273991		
pseudo_2574	0.092177711	-1.632420693	0.630450488
0.035066291			
pseudo_2575	0.377739633	-0.006682013	0.677250751
0.243932748			
pseudo_2576	0.628230174	1.048320741	
0.721816466	-0.483220272		
pseudo_2577	0.001652661	-1.997570474	0.386679641
0.194106037			
pseudo_2578	0.80037864	-0.282245033	0.32565718
0.084218741			
pseudo_2579	0.310131694	1.076699699	
0.620485625	-0.378414386		
pseudo_258	0.723573249	0.132919741	
0.224647984	-0.277045658		
pseudo_2580	0.861934201	-0.345811871	
0.969760218	-0.165458465		
pseudo_2581	0.853955625	-0.321369183	0.216403981
0.467634376			
pseudo_2582	0.59209199	1.286457498	
0.208945899	-0.718460114		
pseudo_2583	0.780512769	-0.379375555	
0.092783512	-1.142963072		
pseudo_2584	0.371850984	-0.794101369	0.074539529
0.608335482			
pseudo_2585	0.2213829	-0.107274248	0.221087767
0.302162823			
pseudo_2586	0.563254046	0.171764098	
0.198383342	-0.670230149		
pseudo_2587	0.30444095	0.605655006	
0.702753762	-0.686082366		
pseudo_2588	0.59263236	0.626963197	
0.997817228	-0.543795632		
pseudo_2589	0.722401898	0.725084323	0.491803587
0.192997941			
pseudo_259	0.715971164	-2.018980412	0.951696667
0.257417317			
pseudo_2590	0.145236126	-1.319862881	0.748317353
0.17409749			
pseudo_2591	0.637689513	-2.552580073	0.146971171
0.468036781			
pseudo_2592	0.83133164	0.815718049	0.022816366
0.603602764			
pseudo_2593	0.956054916	0.674759017	0.830112517
0.196520828			
pseudo_2594	0.199750409	1.317230996	0.91441292
0.12760172			
pseudo_2595	0.440661071	1.058675165	0.119191021
0.471583514			
pseudo_2596	0.540723212	0.46133198	

0.76988633 -0.307960751
pseudo_2597 0.17270847 0.488318238
0.254609399 -0.298386382
pseudo_2598 0.067690951 -4.081250757 0.430994147
0.746697231
pseudo_2599 0.44344571 0.052561552
0.462026978 -0.010307724
pseudo_26 0.417848889 -0.5564185
0.399235558 -0.327957331
pseudo_260 0.570665192 -0.023591469
0.754842226 -0.214048611
pseudo_2600 0.893363482 0.12772652 0.290798248
0.904190372
pseudo_2601 0.319919475 -1.929527268
0.172954752 -0.273715433
pseudo_2602 0.766153529 -0.535258737
0.729439665 -0.445599272
pseudo_2603 0.765556929 -0.469591166
0.20922926 -0.990792318
pseudo_2604 0.270589247 -0.669087379 0.991580881
0.05121517
pseudo_2605 0.400548304 -0.649391163
0.514731467 -0.197126512
pseudo_2606 0.545451241 -0.199361009
0.936144194 -0.223693985
pseudo_2607 0.324888312 -1.542134738 0.460363186
0.726224053
pseudo_2608 0.224051789 -1.15596788 0.217276706
0.718730187
pseudo_2609 0.412033694 0.680409518 0.08402557
0.78223567
pseudo_261 0.199750409 -1.74848686 0.300908793
0.303047671
pseudo_2610 0.16401596 -1.214542572 0.634901131
0.016287913
pseudo_2611 0.318779915 0.625915808
0.716554951 -0.24662364
pseudo_2612 0.884099898 -0.572651647 0.989086465
0.328019936
pseudo_2613 0.090082385 1.292732145
0.637131423 -0.187930762
pseudo_2614 0.514731469 -0.883964936
0.955432225 -0.1668348
pseudo_2615 0.978486682 -0.063613924
0.242433153 -0.248591563
pseudo_2616 0.999688175 -0.490911307 0.589932773
0.661497888
pseudo_2617 0.452329758 0.226718817
0.457518782 -0.265890725
pseudo_2618 0.029521854 2.637412452

0.721231197	-0.414848961
pseudo_2619	0.975014579	0.511178502
0.047456036	-0.61165356
pseudo_262	0.710104763	-1.054160561	0.010617919
0.707971237
pseudo_2620	0.898928706	-0.977094557	0.594254823
0.472294079
pseudo_2621	0.70897874	-1.05252967
0.865621428	-0.184494269
pseudo_2622	0.617450568	1.080003626	0.976329205
0.114167618
pseudo_2623	0.545451241	-0.641286903	0.126394338
0.483057482
pseudo_2624	0.606214449	0.257369041
0.933657902	-0.172520312
pseudo_2625	0.253308015	1.045568092
0.657350122	-0.126887284
pseudo_2626	0.087444907	-1.500141514	0.115170141
0.651072193
pseudo_2627	0.67333186	0.868475619	0.374720305
0.157820996
pseudo_2628	0.710142461	0.387032353
0.717723024	-0.069529348
pseudo_2629	0.33733207	-0.645765602	0.282666034
0.468354464
pseudo_263	0.970383429	-0.481919732	0.921857266
0.472999383
pseudo_2630	0.768541312	-0.567357188	0.608947488
0.19973705
pseudo_2631	0.93303643	0.692340403	0.53254152
0.044840328
pseudo_2632	0.443910792	-0.460098917	0.820982328
0.347259356
pseudo_2633	0.549614904	0.900288274	0.731202702
0.371827567
pseudo_2634	0.183279842	-1.518099992
0.564838234	-0.194422639
pseudo_2635	0.925141645	-1.406342618
0.864530475	-0.245872105
pseudo_2636	0.03829262	-4.498937379	0.445307706
0.292184783
pseudo_2637	0.764364146	-0.326911737
0.83621216	-0.244834974
pseudo_2638	0.485630437	-2.1717747	0.220353252
0.192424377
pseudo_2639	0.916273329	0.026595522
0.143302998	-0.35548905
pseudo_264	0.216403983	-0.39195034
0.473284662	-0.178073808
pseudo_2640	0.836751338	-1.461288251

0.76291266 -0.897253578
pseudo_2641 0.588315748 0.000510061 0.417848887
0.134498227
pseudo_2642 0.093698205 1.682867397 0.768541311
0.335414723
pseudo_2643 0.079769985 -2.171716832 0.227645977
1.159138648
pseudo_2644 0.94485096 -0.041775119
0.639923916 -0.281660593
pseudo_2645 0.106767929 1.851198596
0.209796818 -1.069281309
pseudo_2646 0.330683657 -0.612004953
0.740039024 -0.136880526
pseudo_2647 0.246870601 0.417352408
0.567483301 -0.073420815
pseudo_2648 0.677850473 -0.628929925 0.477928351
0.390966074
pseudo_2649 0.434662201 -0.201077197
0.892745442 -0.137106789
pseudo_265 0.219322905 -1.039483919
0.896454643 -0.156464905
pseudo_2650 0.999064525 0.564534218 0.758408669
0.161485041
pseudo_2651 0.109512949 0.221819388
0.219616342 -0.645701413
pseudo_2652 0.811091031 -0.530914893 0.62969332
0.25851777
pseudo_2653 0.950451713 0.326860669
0.018423079 -1.014429229
pseudo_2654 0.084446977 -2.617038061 0.17718184
0.197016746
pseudo_2655 0.416054468 -0.592795539 0.735911055
0.181857308
pseudo_2656 0.271610325 0.676692653 0.053288127
0.873455445
pseudo_2657 0.343667324 1.369994095 0.973499749
0.149892395
pseudo_2658 0.045353392 -2.201762463 0.215533782
0.809157097
pseudo_2659 0.369761766 -0.116820518 0.770333592
0.204343186
pseudo_266 0.924961136 0.199259968 0.827675501
0.232669642
pseudo_2660 0.463694125 -0.876097137 0.68011259
0.187521049
pseudo_2661 0.244644816 -0.746680374
0.152047734 -0.497773578
pseudo_2662 0.603486909 0.076621909
0.241489616 -0.31144451
pseudo_2663 0.202782144 1.41722979

0.047063376 -0.584219586
pseudo_2664 0.778713653 0.649948936 0.103413655
0.324524219
pseudo_2665 0.764364146 0.534838816 0.382406501
0.635498129
pseudo_2666 0.604031976 -1.334551904 0.259530983
0.610196526
pseudo_2667 0.243063619 -1.254248623 0.26518796
0.857877401
pseudo_2668 0.764960468 -0.878072826 0.537686798
0.327403173
pseudo_2669 0.523088909 -0.017379705 0.160492961
0.834948643
pseudo_267 0.77811421 0.492110336
0.708397133 -0.48962687
pseudo_2670 0.378162438 0.245714486
0.334192772 -0.143141183
pseudo_2671 0.773921828 0.014380409
0.37310802 -0.411831579
pseudo_2672 0.81551575 -1.343457652 0.113022338
0.776172534
pseudo_2673 0.368928125 -0.522934303 0.965398277
0.201025988
pseudo_2674 0.289728691 -0.535161214 0.34247371
0.483149526
pseudo_2675 0.241803843 -1.209362376
0.456572844 -0.213454749
pseudo_2676 0.991580881 -0.242164007
0.548572519 -0.068521976
pseudo_2677 0.538718843 -1.082638605 0.663012046
0.322405554
pseudo_2678 0.208662818 -1.008212271 0.496740833
0.459287564
pseudo_2679 0.680685514 -2.027579189
0.276065464 -0.850769825
pseudo_268 0.874852133 -0.229241966
0.498723104 -0.054310434
pseudo_2680 0.552747899 -1.526952245
0.113200108 -0.656078431
pseudo_2681 0.075691268 -3.2123389
0.087880107 -0.362253302
pseudo_2682 0.880398824 -2.42898813
0.282315991 -0.261331104
pseudo_2683 0.866851159 0.714940393
0.060186099 -0.306499225
pseudo_2684 0.37478816 -1.137832232
0.338119844 -0.370305882
pseudo_2685 0.534596639 1.217867383 0.369344797
0.139286205
pseudo_2686 0.302738604 -1.019012317

0.771529128 -0.280035248
pseudo_2687 0.178944602 -3.194286258 0.184311113
0.480236712
pseudo_2688 0.570665192 0.674646493
0.303840031 -0.713612381
pseudo_2689 0.974123063 0.203139796 0.808240968
0.095467976
pseudo_269 0.485424746 -0.716958946
0.94858451 -0.256051166
pseudo_2690 0.627121274 -0.306526388
0.451389883 -0.43604269
pseudo_2691 0.666418493 -0.622955214
0.019866233 -0.498408938
pseudo_2692 0.463228925 0.823536264 0.816847974
0.102949704
pseudo_2693 0.126200531 1.415695089 0.650581705
0.284236067
pseudo_2694 0.474732598 0.820003344
0.764364145 -0.044523666
pseudo_2695 0.442980906 0.623101484 0.139496039
0.387888961
pseudo_2696 0.876700361 -1.190033856 0.375208922
0.561243465
pseudo_2697 0.772127101 0.390827213 0.749502369
0.325451558
pseudo_2698 0.697379261 -0.641869993
0.447174153 -0.353948567
pseudo_2699 0.677250753 0.277212874
0.581332413 -0.436882385
pseudo_27 0.779912933 0.704107353
0.700851831 -0.370317024
pseudo_270 0.132116217 -1.37737895
0.753654568 -0.239098496
pseudo_2700 0.919375024 0.555625946 0.338119842
0.178195159
pseudo_2701 0.967267561 0.656330914
0.458939749 -0.464779552
pseudo_2702 0.07166114 0.768119056 0.41381785
0.193835632
pseudo_2703 0.560618503 0.462099456
0.929929686 -0.241117774
pseudo_2704 0.665850271 -2.271266748
0.858863804 -0.39028321
pseudo_2705 0.669831831 -0.323603297 0.207251569
0.240658267
pseudo_2706 0.432825926 -0.564018303 0.785316136
0.16085613
pseudo_2707 0.94174059 -2.403594991
0.942984634 -0.145438704
pseudo_2708 0.982850945 0.515207437

0.108305212 -0.535246017
pseudo_2709 0.104796753 -1.19586362 0.046269989
0.6848724
pseudo_271 0.436042354 -0.158710925 0.350078046
0.320166672
pseudo_2710 0.726742553 -0.34018984 0.688742269
0.010852841
pseudo_2711 0.655078804 0.588867827
0.005908133 -0.64233157
pseudo_2712 0.402741967 -0.130705119 0.629339911
0.294175251
pseudo_2713 0.614979388 0.393097728 0.052426704
0.690917837
pseudo_2714 0.984721509 0.369225917
0.753654568 -0.264050035
pseudo_2715 0.079233867 -1.134576693 0.838043992
0.133785882
pseudo_2716 0.523343249 -0.975506292
0.697957584 -0.091523189
pseudo_2717 0.155202537 -0.326044638 0.892745442
0.344690087
pseudo_2718 0.163779296 -1.474445586
0.524361237 -0.353767874
pseudo_2719 0.68642483 0.015788317
0.956677633 -0.179165601
pseudo_272 0.900166108 -0.594669798 0.703170362
0.424739883
pseudo_2720 0.793882574 -1.54470665 0.955961939
0.447403961
pseudo_2721 0.09523875 -2.843394754 0.20922926
0.823110868
pseudo_2722 0.436502965 -0.588838601
0.157256711 -0.031163462
pseudo_2723 0.465125769 0.443434286
0.353717202 -0.33711745
pseudo_2724 0.199202759 1.155887795 0.94920688
0.231208907
pseudo_2725 0.265187962 -0.532229901
0.65622008 -0.402136458
pseudo_2726 0.69911476 -0.321216403
0.053868953 -1.080029282
pseudo_2727 0.326812705 0.65850591
0.735321964 -0.106057328
pseudo_2728 0.506694055 0.470538476 0.189005037
0.634804759
pseudo_2729 0.37101442 -0.629304391 0.097904174
0.352807838
pseudo_273 0.12408388 -0.33247322
0.689877145 -0.470857176
pseudo_2730 0.775118985 -0.478911976

0.8149089	-0.034547182
pseudo_2731	0.154522358	-2.796189471	0.172708468
0.679045549
pseudo_2732	0.995946292	1.174021184	0.30310545
0.126377235
pseudo_2733	0.000579304	2.352779582	0.589393536
0.351044776
pseudo_2734	0.302738604	-0.198674725
0.331072375	-0.907364816
pseudo_2735	0.597853573	0.57203683	0.620937864
0.266571809
pseudo_2736	0.084025572	1.243419058
0.316131289	-0.61702873
pseudo_2737	0.157486221	-1.254904725	0.603486907
0.247433629
pseudo_2738	0.820982328	-0.17782325
0.527421282	-0.328026788
pseudo_2739	0.285476969	-0.718744259
0.717138906	-0.282606973
pseudo_274	0.186710635	-1.104676996	0.27771138
0.197399639
pseudo_2740	0.690453162	-1.030962164	0.80219126
0.137265586
pseudo_2741	0.919375024	-0.953267218	0.747132931
0.149817126
pseudo_2742	0.043370871	1.227693679
0.494440041	-0.238716501
pseudo_2743	0.367264354	-0.485734816
0.327198469	-0.81549364
pseudo_2744	0.795550264	0.509189621
0.675535879	-0.116580168
pseudo_2745	0.94858451	0.656436987
0.714220795	-0.259831329
pseudo_2746	0.314624297	-1.168527166
0.124083878	-0.324260173
pseudo_2747	0.115350566	-0.2446233	0.011880497
0.698660789
pseudo_2748	0.262848414	-0.870957226
0.882249039	-0.503383695
pseudo_2749	0.826457615	-2.775541773
0.21322558	-0.311100371
pseudo_275	0.807030097	-0.440493476	0.700272633
0.320175464
pseudo_2750	0.300178942	-1.003033223
0.477635748	-0.264323153
pseudo_2751	0.825848828	0.256620438
0.043859473	-0.735816862
pseudo_2752	0.002175539	2.712475867	0.808240968
0.186960882
pseudo_2753	0.071292503	1.238025147	0.405383844

0.519133097
pseudo_2754 0.600221164 0.052046598
0.827675502 -0.151344674
pseudo_2755 0.1772664 1.294607442
0.361562225 -1.167557207
pseudo_2756 0.371182866 0.037275127 0.273356622
0.18580139
pseudo_2757 0.60103685 0.60841261 0.366434222
0.60822634
pseudo_2758 0.527932175 0.864197763 0.959168762
0.141886898
pseudo_2759 0.648894067 0.421090286 0.409366024
0.558798317
pseudo_276 0.234649293 0.804780756
0.113200106 -0.477797473
pseudo_2760 0.275165399 1.077786925
0.859348492 -0.052592116
pseudo_2761 0.714804086 0.267392893
0.078038312 -0.526629839
pseudo_2762 0.841098994 0.037274553 0.482983321
0.08267843
pseudo_2763 0.476666955 -0.803437088
0.78231306 -0.079597224
pseudo_2764 0.616078915 0.357946642 0.942984634
0.121394445
pseudo_2765 0.390550275 -1.28020365 0.252983391
0.477262413
pseudo_2766 0.659046656 -0.349550939
0.698536083 -0.004759563
pseudo_2767 0.739448853 -0.532440649 0.807030096
0.225513579
pseudo_2768 0.860705789 -1.554431067
0.108305212 -0.433792699
pseudo_2769 0.873004588 0.043265388
0.878549261 -0.152824017
pseudo_277 0.399672853 0.813683203 0.806424833
0.067652942
pseudo_2770 0.668693289 1.106868298 0.464171065
0.54323956
pseudo_2771 0.672681501 0.949744707 0.346462734
0.0124453
pseudo_2772 0.114630206 -3.32413832 0.58401372
0.064945425
pseudo_2773 0.226743603 -0.656039484 0.216985517
0.292323933
pseudo_2774 0.253632926 -1.62443999
0.659612556 -0.673290693
pseudo_2775 0.161170868 1.575994517 0.311086407
0.443788358
pseudo_2776 0.201124341 0.407747102

0.235265409 -0.603646089
pseudo_2777 0.768541312 0.345743224
0.776915721 -0.042324217
pseudo_2778 0.796756632 0.258937937 0.110906225
0.290927781
pseudo_2779 0.431909475 0.938468601
0.626567138 -0.632326722
pseudo_278 0.053917593 0.100653872 0.699114758
0.001968943
pseudo_2780 0.703750429 -0.319547088
0.122941073 -0.430381117
pseudo_2781 0.101768221 0.334716269
0.916273328 -0.310549885
pseudo_2782 0.9861907 -0.416509784
0.042776047 -0.870888672
pseudo_2783 0.192983493 0.572738279
0.264518059 -0.301175637
pseudo_2784 0.057056635 0.599454249
0.365191224 -0.065959646
pseudo_2785 0.825848828 0.220053733 0.097666617
0.63504961
pseudo_2786 0.404693107 -0.78372085 0.829033665
0.12444734
pseudo_2787 0.004972882 1.503701744
0.748317353 -0.311051189
pseudo_2788 0.864392024 -0.262876047
0.811270151 -0.770563669
pseudo_2789 0.688135449 -0.861624973
0.891826469 -0.084963128
pseudo_279 0.051953189 2.101985124 0.879165709
0.142829674
pseudo_2790 0.607307007 0.358250486
0.767944158 -0.109185311
pseudo_2791 0.744174494 -0.890083822 0.40450207
0.2439182
pseudo_2792 0.145236127 -0.527295291
0.052617117 -0.203845452
pseudo_2793 0.957923146 0.357780567 0.646085339
0.365140021
pseudo_2794 0.212938317 1.386939153 0.155884985
0.64552953
pseudo_2795 0.212364623 -1.132953484 0.226143436
0.377017089
pseudo_2796 0.689877146 0.636886941
0.784114519 -0.221297521
pseudo_2797 0.563143845 -0.738700809
0.719948104 -0.056650794
pseudo_2798 0.51284325 0.348250847
0.225358499 -0.221801582
pseudo_2799 0.611657556 -0.340801781 0.846420484

0.129899102
pseudo_28 0.939252931 -0.091128785
0.157945997 -0.775647912
pseudo_280 0.11196034 1.035258576
0.66585027 -0.515241174
pseudo_2800 0.410698573 -1.750223736
0.384539441 -1.203568155
pseudo_2801 0.929929686 0.232070451
0.940496688 -0.479837322
pseudo_2802 0.768541312 -2.296872559 0.597505831
0.770734923
pseudo_2803 0.292586743 0.846676131 0.318779913
0.302510007
pseudo_2804 0.306050867 0.494000587 0.263515395
0.53557798
pseudo_2805 0.833771099 -0.298555183 0.474249682
0.180229845
pseudo_2806 0.647769975 0.107906083 0.014917132
0.629879628
pseudo_2807 0.094312007 -2.29553073
0.471839161 -0.266172751
pseudo_2808 0.409366026 0.638441068 0.194457567
0.492809515
pseudo_2809 0.306420374 -2.853655145
0.020411117 -0.992489847
pseudo_281 0.200573943 1.507274233 0.653962357
0.250208387
pseudo_2810 0.547010779 -1.131342192 0.978486682
0.33297562
pseudo_2811 0.766685668 -1.109753495 0.616870185
0.156130199
pseudo_2812 0.001282254 1.740984146
0.869926888 -0.018609909
pseudo_2813 0.655674083 -0.175126452
0.633230582 -0.34699639
pseudo_2814 0.703170363 -0.670345583 0.987215722
0.282498373
pseudo_2815 0.320299919 0.077377329
0.720646092 -0.176556107
pseudo_2816 0.962283232 0.170255876 0.765556928
0.05669821
pseudo_2817 0.048470122 1.649859633 0.751874174
0.172844011
pseudo_2818 0.11789989 1.965287584
0.184828358 -0.836503106
pseudo_2819 0.85763624 -2.324278362
0.361478054 -0.856409864
pseudo_282 0.164965195 -0.472658114
0.663012046 -0.136810416
pseudo_2820 0.91441292 -0.685437567 0.563254045

0.058378859
pseudo_2821 0.46943541 1.93563554
0.659612556 -0.192602165
pseudo_2822 0.944228817 0.31707751 0.118821001
0.521344633
pseudo_2823 0.076727189 -1.609588532 0.980980476
0.286791503
pseudo_2824 0.110749783 1.521758287 0.477912001
0.01523659
pseudo_2825 0.315754099 -0.63953801 0.227947337
0.327981005
pseudo_2826 0.540268768 0.385306933
0.747725068 -0.751490622
pseudo_2827 0.276065466 -1.278751701 0.217859928
1.119037073
pseudo_2828 0.905118112 0.481801084
0.48640318 -1.299353297
pseudo_2829 0.852729455 0.313860133
0.673822688 -0.006109189
pseudo_283 0.174437973 1.533547594
0.892127468 -0.329239463
pseudo_2830 0.135767409 1.200621983
0.928066149 -0.251635016
pseudo_2831 0.062460352 0.977508057
0.017849384 -0.599512761
pseudo_2832 0.055443706 -0.577232488
0.959168762 -0.171322781
pseudo_2833 0.160027584 2.195205738
0.791934101 -0.301172411
pseudo_2834 0.096172773 1.755800463
0.137829252 -0.174298555
pseudo_2835 0.585087854 -0.696238879
0.639365007 -0.239176611
pseudo_2836 0.983474456 0.118188282
0.993451748 -0.428494984
pseudo_2837 0.685275507 -0.355189366 0.618280551
0.213206787
pseudo_2838 0.294742665 -2.78520399 0.983474456
0.00355661
pseudo_2839 0.571196331 0.642838264
0.40098646 -0.213429576
pseudo_284 0.831941353 -0.304965098 0.748909787
0.178117763
pseudo_2840 0.64272152 -0.288797235 0.367679855
0.158227462
pseudo_2841 0.639365009 0.775437442 0.329907101
0.038337267
pseudo_2842 0.096956712 1.656962023 0.674393564
0.308762929
pseudo_2843 0.974123063 1.191602519 0.613331717

0.39867709
pseudo_2844 0.190059999 0.413081425
0.204728801 -0.503648494
pseudo_2845 0.249224397 -1.880367691
0.088914023 -0.421353604
pseudo_2846 0.095083788 1.854559104 0.285829657
0.819373968
pseudo_2847 0.073162357 -1.212688767 0.578544105
0.317909402
pseudo_2848 0.283367004 -0.815721402
0.230368454 -0.92873432
pseudo_2849 0.5376868 0.231324375 0.123321091
0.143704447
pseudo_285 0.084870091 1.391760115 0.911933106
0.246099115
pseudo_2850 0.573855554 -0.203838684 0.839265702
0.281806212
pseudo_2851 0.497236009 -2.474054978 0.263849325
0.593476343
pseudo_2852 0.866851159 0.544791009
0.618280553 -0.090540082
pseudo_2853 0.587026844 0.710334714
0.604162846 -0.398855875
pseudo_2854 0.761980261 -0.244950703
0.052521839 -0.623392131
pseudo_2855 0.853342496 -1.859027112 0.250074838
0.049787238
pseudo_2856 0.318779915 0.669627611 0.638247805
0.081890306
pseudo_2857 0.536140599 -0.565604117 0.38539465
0.575502651
pseudo_2858 0.589393538 0.668027328
0.08875575 -1.092712401
pseudo_2859 0.516751124 0.685232411 0.796756631
0.018839991
pseudo_286 0.952022793 0.394597991 0.198816073
0.280315891
pseudo_2860 0.852729455 0.472585741
0.450451116 -0.015301719
pseudo_2861 0.45610029 -0.181359333 0.267204672
0.080922026
pseudo_2862 0.166876063 2.005268798 0.455156002
0.248630338
pseudo_2863 0.505194418 0.546612013
0.455156002 -0.488800345
pseudo_2864 0.426434428 1.329613614
0.309015192 -0.947369795
pseudo_2865 0.775118985 0.449844721 0.605668499
0.040088579
pseudo_2866 0.919995512 0.48257654 0.625459502

0.192673501
pseudo_2867 0.085294917 -0.701218182 0.25265906
0.557146716
pseudo_2868 0.157945998 1.212109002
0.719476373 -0.023081232
pseudo_2869 0.507285064 -0.248959535 0.236262988
0.225340685
pseudo_287 0.252011269 1.585588283
0.488363247 -1.137471507
pseudo_2870 0.294022845 -1.449966505
0.647769973 -0.403606169
pseudo_2871 0.591551846 -0.181701246
0.629339911 -0.594622216
pseudo_2872 0.890891717 -0.982801406 0.417848887
0.231936593
pseudo_2873 0.718307311 0.586346963
0.157945997 -0.256853167
pseudo_2874 0.257554503 -0.724117361
0.759598631 -0.013849397
pseudo_2875 0.230672377 2.170990946
0.115893166 -1.20496348
pseudo_2876 0.258212167 -2.472013948 0.559565958
0.287806782
pseudo_2877 0.148282826 -1.045433572
0.442052133 -0.930098631
pseudo_2878 0.668124304 0.26352232 0.585625263
0.968191422
pseudo_2879 0.953564319 -0.058944213
0.775717763 -0.303116907
pseudo_288 0.072635641 1.689446805 0.402836739
0.24109626
pseudo_2880 0.617179305 0.482649274 0.457992163
0.371613357
pseudo_2881 0.486403182 2.570926271
0.908215001 -0.227923105
pseudo_2882 0.624906002 -0.568657133
0.905737375 -0.724605165
pseudo_2883 0.689301309 -2.643139279 0.70607241
0.111018246
pseudo_2884 0.566953815 0.404296925 0.716554951
0.102823684
pseudo_2885 0.479576566 0.061399845 0.720061151
0.373937379
pseudo_2886 0.923098674 -0.297233452
0.811876329 -0.703442138
pseudo_2887 0.56589555 -2.024513451 0.134745458
0.080296053
pseudo_2888 0.822806469 -0.752805817 0.332240309
0.829351679
pseudo_2889 0.222566246 -1.771586447

0.85150364 -0.247413864
pseudo_289 0.74920606 0.039549556
0.805214656 -0.223118427
pseudo_2890 0.261850126 -1.505829225 0.76198026
0.3005873
pseudo_2891 0.256570188 1.353059487 0.831941353
0.056288278
pseudo_2892 0.830722028 0.100204577
0.158176266 -0.476390345
pseudo_2893 0.48885393 -0.62112286
0.124850341 -0.636459717
pseudo_2894 0.056245357 -2.17291661 0.250719156
0.28178442
pseudo_2895 0.446707126 1.028309705 0.334975824
0.817584716
pseudo_2896 0.623799634 0.854083356
0.504195959 -0.529886425
pseudo_2897 0.579726399 -0.40745397
0.131315265 -1.105834831
pseudo_2898 0.245914963 0.778429483
0.414264603 -0.119566168
pseudo_2899 0.550658262 -0.501333852
0.734144249 -0.094908175
pseudo_29 0.494762772 -0.481634733
0.247189723 -0.249947927
pseudo_290 0.529466362 0.44030599
0.748909787 -0.333783418
pseudo_2900 0.42401413 -0.544208891 0.849002897
0.247588177
pseudo_2901 0.820982328 -1.007033709 0.633787224
0.353624359
pseudo_2902 0.755436274 -0.276114481
0.180211678 -0.904339181
pseudo_2903 0.317643017 -2.263171226
0.765556928 -0.246484589
pseudo_2904 0.936144194 0.736752221
0.23128107 -0.633660798
pseudo_2905 0.846604 0.428128685 0.927445057 0.295495032
pseudo_2906 0.871465495 -0.372277353 0.792536486
0.325468929
pseudo_2907 0.290441436 1.131205004
0.400110433 -0.468237398
pseudo_2908 0.955432225 -1.980363721 0.797359998
0.235648598
pseudo_2909 0.691605732 0.379854072 0.982227445
0.246882935
pseudo_291 0.770931293 1.930824817
0.182765809 -0.281963868
pseudo_2910 0.591551846 -1.44281841 0.596963435
0.32655152

pseudo_2911	0.833161084	−0.410438932	0.364364023	0.21921781
pseudo_2912	0.104411024	1.184766275	0.162835209	0.65890462
pseudo_2913	0.887186063	−0.161352358	0.358197404	−0.24755008
pseudo_2914	0.60239744	−0.876290751	0.735911055	0.257864458
pseudo_2915	0.177684163	−1.295497564	0.916273328	−0.668739271
pseudo_2916	0.645524199	−0.562062122	0.551702593	0.287421457
pseudo_2917	0.112667461	2.008195303	0.995322652	0.324819119
pseudo_2918	0.754842227	0.428072727	0.92682401	−0.100232741
pseudo_2919	0.312747205	2.105903547	0.191710059	−0.601691881
pseudo_292	0.195399911	−2.79038208	0.392278084	0.208216983
pseudo_2920	0.898310097	1.007111068	0.326812703	−0.049907839
pseudo_2921	0.017849384	2.225372778	0.587238868	0.105703938
pseudo_2922	0.374367687	1.542805074	0.863162946	0.117323303
pseudo_2923	0.313850381	−0.279527133	0.822198314	−0.604566737
pseudo_2924	0.641042347	−0.72840697	0.754248324	0.204335524
pseudo_2925	0.375208924	−0.424666697	0.16759691	0.213547604
pseudo_2926	0.706072412	0.709461063	0.237741327	0.8095556
pseudo_2927	0.239922806	0.877923859	0.295103016	0.621801379
pseudo_2928	0.818551647	0.350289511	0.733555625	0.09588041
pseudo_2929	0.819766772	0.043095384	0.255261833	−0.565806758
pseudo_293	0.462264939	−0.249197227	0.57066519	−0.532161085
pseudo_2930	0.036998739	1.221987721	0.269909991	−0.274695725
pseudo_2931	0.007162438	−2.289639064	0.301274165	0.23250551
pseudo_2932	0.474732598	−0.418830939	0.23619173	−0.509881426
pseudo_2933	0.172462449	−0.97595324	0.551702593	0.399902414

pseudo_2934 0.135357913 0.999736212
0.848440681 -0.069332042
pseudo_2935 0.883482875 -2.207534549 0.706653335
0.14662114
pseudo_2936 0.174934484 -1.642692981
0.383258805 -0.253158751
pseudo_2937 0.10209563 0.921058113
0.554841416 -0.207491037
pseudo_2938 0.80037864 0.121032845 0.131315264
0.793788171
pseudo_2939 0.87362036 -0.236224582 0.985968598
0.131040297
pseudo_294 0.307901355 0.847616191 0.517256677
0.283570849
pseudo_2940 0.112136791 1.506362135 0.813695536
0.367277837
pseudo_2941 0.900166108 -0.787556757
0.052426704 -0.768942668
pseudo_2942 0.860705789 -0.453679463
0.394444355 -0.17366659
pseudo_2943 0.572791161 -0.720958304 0.032631851
0.597049476
pseudo_2944 0.537833623 0.683008673 0.714378192
0.015098529
pseudo_2945 0.854568842 0.925551135 0.700851831
0.259727858
pseudo_2946 0.956054916 0.542759414 0.226743601
0.30112022
pseudo_2947 0.082771561 0.952419056
0.023003951 -1.250744439
pseudo_2948 0.830961568 0.83953926
0.298656151 -0.512616162
pseudo_2949 0.116256016 0.345775356 0.663012046
0.125223829
pseudo_295 0.288661784 1.434359425
0.375629977 -0.352244349
pseudo_2950 0.483959091 0.43803589 0.707815696
0.148847779
pseudo_2951 0.418298205 0.884703786 0.201952001
0.99111818
pseudo_2952 0.291512763 2.272220766 0.144374506
0.598961461
pseudo_2953 0.543375295 0.315847793
0.222269984 -0.790799731
pseudo_2954 0.100466938 -1.536099448 0.857022587
0.097083378
pseudo_2955 0.268691917 1.596349857
0.118708937 -0.533816959
pseudo_2956 0.508196028 -0.138121142
0.316508777 -0.572354021

pseudo_2957 0.65339842 0.594985019 0.518268555
0.621886952
pseudo_2958 0.408036055 1.773876613 0.996569936
0.143711655
pseudo_2959 0.359835383 0.759410999 0.338119844
0.171580995
pseudo_296 0.607307007 -0.185732926
0.031689269 -0.418980712
pseudo_2960 0.904498905 0.490752645 0.150488756
0.704923431
pseudo_2961 0.446240376 -1.007192559 0.261186056
0.594077405
pseudo_2962 0.449045042 0.834662836
0.988462877 -0.285744302
pseudo_2963 0.153905704 0.925634165
0.34070243 -0.254267098
pseudo_2964 0.339303724 0.495607151
0.270589245 -1.006020039
pseudo_2965 0.185087385 -0.376957846 0.650018959
0.054167536
pseudo_2966 0.047586828 -1.478186465 0.338908801
0.593119437
pseudo_2967 0.18225285 2.026473776 0.448576902
0.368494401
pseudo_2968 0.167116087 1.422718489 0.70723443
0.157242359
pseudo_2969 0.033208847 -1.158070063 0.021718534
0.750149704
pseudo_297 0.972876451 -0.053088298 0.593172953
0.008470114
pseudo_2970 0.313122032 0.688719884
0.394878476 -0.397591614
pseudo_2971 0.032600049 -1.890887137 0.784114519
0.373168069
pseudo_2972 0.983474456 0.398352638 0.330683655
0.087511843
pseudo_2973 0.991580881 -0.467103432 0.607853614
0.175308389
pseudo_2974 0.664714405 -2.21719311 0.481521676
0.049917085
pseudo_2975 0.761980261 0.564993314 0.568542986
0.107267968
pseudo_2976 0.375447351 0.543424495 0.228005709
0.63156681
pseudo_2977 0.113200109 -0.950502845
0.388397024 -0.374617431
pseudo_2978 0.334192774 -0.91239816 0.458465819
0.320812375
pseudo_2979 0.594254825 1.227383343
0.636573541 -0.1616074

pseudo_298 0.676644368 0.399106322 0.0540842
0.697113938
pseudo_2980 0.618831501 0.646510237 0.09293546
0.493474985
pseudo_2981 0.401424907 0.901021357
0.310504449 -0.298726716
pseudo_2982 0.693913016 1.175182436
0.732967159 -0.049840964
pseudo_2983 0.019824851 1.665394168 0.128541561
0.511006666
pseudo_2984 0.701431205 -1.423227547 0.777514899
0.601021385
pseudo_2985 0.821590268 0.689987948
0.816122709 -0.72553409
pseudo_2986 0.51776249 -0.278577544 0.438810225
0.237271912
pseudo_2987 0.370179025 0.41925485
0.032189032 -0.66358334
pseudo_2988 0.388397026 0.848856161
0.005119678 -0.637942088
pseudo_2989 0.504195961 0.496012379
0.544412772 -0.393905347
pseudo_299 0.390981794 0.564695383 0.259530983
0.312655954
pseudo_2990 0.009426613 -2.068964004 0.333410901
0.729482963
pseudo_2991 0.604304591 -0.526135948
0.623246767 -0.107561555
pseudo_2992 0.893981586 -0.062882156
0.27641022 -0.204630012
pseudo_2993 0.647208229 0.915525163 0.674964628
0.121681089
pseudo_2994 0.362713141 -0.711376273 0.432825924
0.238705197
pseudo_2995 0.883482875 0.389323509
0.041306175 -0.620148369
pseudo_2996 0.666418493 -0.312348014 0.122183782
0.573201153
pseudo_2997 0.638247806 1.392514749
0.203892861 -0.447794806
pseudo_2998 0.472320726 -1.137181229 0.709560515
0.202778772
pseudo_2999 0.56589555 0.419447831
0.90945415 -0.065809719
pseudo_3 0.568013027 0.086559008
0.417848887 -0.659188885
pseudo_30 0.80037864 -0.679866543
0.093087609 -0.530341939
pseudo_300 0.629339913 0.082569461
0.254283617 -0.661994134

pseudo_3000 0.391413602 -0.605320621 0.503697121
0.118760322
pseudo_3001 0.799774672 0.483754197 0.816122709
0.268267689
pseudo_3002 0.582940508 0.745904931
0.713054717 -0.329242607
pseudo_3003 0.878160367 -0.635060968 0.2839611
0.417982228
pseudo_3004 0.66131143 0.563498072
0.931172258 -0.683763845
pseudo_3005 0.947339864 -0.314013524 0.721816466
0.247051088
pseudo_3006 0.505694037 0.558727607 0.081259523
0.197758336
pseudo_3007 0.287243366 0.639103596 0.857022587
0.316663212
pseudo_3008 0.803400267 -2.268467433 0.38028083
0.112100349
pseudo_3009 0.098859022 -1.438137872 0.5743881
0.277672655
pseudo_301 0.064015658 -3.432256154 0.799774671
0.244620286
pseudo_3010 0.56589555 -0.64541449 0.538718841
0.773606085
pseudo_3011 0.296547379 -1.350136615
0.672111188 -0.15700091
pseudo_3012 0.099499706 -2.61891924
0.195669763 -1.374250573
pseudo_3013 0.466081562 -0.555691886 0.531002811
0.434234827
pseudo_3014 0.943606708 -1.487357372
0.296547377 -0.276614638
pseudo_3015 0.505194418 0.01706922
0.114630204 -0.592303023
pseudo_3016 0.282666036 -0.40986884
0.291155357 -0.852686214
pseudo_3017 0.20979682 0.428488624
0.219616342 -0.190945457
pseudo_3018 0.623246768 -0.707122845
0.466559865 -0.127808961
pseudo_3019 0.216985519 1.103612706
0.936144194 -0.020422435
pseudo_302 0.338119846 -2.491201447
0.752467493 -0.473713428
pseudo_3020 0.694490281 0.892107289
0.039027923 -0.867386923
pseudo_3021 0.87978223 -0.509953506 0.688150175
0.032024825
pseudo_3022 0.963529184 -0.40404091
0.496245922 -0.398870341

pseudo_3023 0.331072377 0.632663236 0.529978259
0.180091318
pseudo_3024 0.22494651 -1.447577448 0.107449017
0.801295272
pseudo_3025 0.269231908 -0.64405338
0.449982147 -0.509608653
pseudo_3026 0.173942515 1.246476848 0.695645342
0.203839883
pseudo_3027 0.17369518 -2.960103953 0.871773274
0.753072589
pseudo_3028 0.659612557 0.995644871 0.22855091
0.785294828
pseudo_3029 0.058708408 -2.836429448
0.657915438 -0.117535937
pseudo_303 0.074412447 2.193339651 0.770333592
0.187934688
pseudo_3030 0.078302714 1.826436668
0.382406501 -0.391402998
pseudo_3031 0.588315748 0.98764773 0.059337973
0.731886494
pseudo_3032 0.999688175 -0.501694516 0.971006658
0.310948874
pseudo_3033 0.680112591 -0.254196723
0.510704496 -0.106987628
pseudo_3034 0.326812705 -0.927824725
0.696223138 -0.0417348
pseudo_3035 0.137208182 -0.337739162
0.220498351 -0.713709869
pseudo_3036 0.883482875 1.44071182
0.046030995 -0.225239623
pseudo_3037 0.330295232 1.139230619 0.248789674
0.415004561
pseudo_3038 0.604577263 0.020338316 0.852729455
0.023726436
pseudo_3039 0.173448109 2.441193419 0.748909787
0.161005582
pseudo_304 0.159099888 1.880278487
0.659612556 -0.703851375
pseudo_3040 0.227044111 2.148649244 0.150710718
0.846144346
pseudo_3041 0.052236867 -1.134593217
0.644963256 -0.24334706
pseudo_3042 0.957300377 -0.544193907 0.913172907
0.145035583
pseudo_3043 0.425980008 0.606479037 0.132719407
0.431399052
pseudo_3044 0.673632737 1.781322519
0.855465964 -0.320287676
pseudo_3045 0.89027394 0.411054072
0.489344875 -0.712871251

pseudo_3046 0.605122771 0.367266911 0.522326273
0.258479665
pseudo_3047 0.56272646 0.122383885
0.842321667 -0.315068281
pseudo_3048 0.798567094 0.340475422
0.90945415 -0.199773897
pseudo_3049 0.098062803 1.774479253
0.542856922 -0.179958894
pseudo_305 0.927445058 0.506361148 0.417399855
0.182238469
pseudo_3050 0.005985043 1.815526601 0.476666953
0.210648342
pseudo_3051 0.012227392 -2.226377781
0.143516808 -0.337910745
pseudo_3052 0.049820852 1.193910927
0.258212165 -0.066476301
pseudo_3053 0.865621429 0.681014437
0.87978223 -0.108036662
pseudo_3054 0.355749252 0.304702811
0.622694113 -0.257933524
pseudo_3055 0.91069352 0.229443423 0.807635474
0.22470543
pseudo_3056 0.904498905 -0.617349285
0.691029357 -0.238717586
pseudo_3057 0.244961919 -0.482361895 0.839876703
0.219931164
pseudo_3058 0.403181563 0.056539674
0.223456721 -0.853032542
pseudo_3059 0.847216135 -0.048122243
0.114271354 -0.481588259
pseudo_306 0.524361239 -0.055203667
0.556414095 -0.517294804
pseudo_3060 0.082910142 -0.615903275 0.154748831
0.612901417
pseudo_3061 0.033338247 1.33323718 0.366434222
0.175315927
pseudo_3062 0.724745246 0.009007425 0.987839296
0.242574799
pseudo_3063 0.375629979 0.148259749 0.758408669
0.173248558
pseudo_3064 0.036087588 1.97203566
0.532028367 -0.050002211
pseudo_3065 0.510202286 0.049124478
0.507695108 -0.149708518
pseudo_3066 0.638247806 -2.416585145
0.281616787 -0.93014004
pseudo_3067 0.804004946 -0.835331465 0.431909473
0.31366987
pseudo_3068 0.770931293 0.853224288
0.089049189 -0.649536523

pseudo_3069 0.46943541 −1.243549658
0.124083878 −0.775905882
pseudo_307 0.215533784 1.173349435
0.887803503 −0.538923778
pseudo_3070 0.140967169 −0.711449624
0.542856922 −0.149832217
pseudo_3071 0.838654799 0.520743873 0.009976544
0.617898818
pseudo_3072 0.364364026 −0.897530014
0.176430344 −0.323325932
pseudo_3073 0.292586743 0.425393377
0.783513904 −0.211450932
pseudo_3074 0.877932887 −0.623479838
0.292945324 −0.474524952
pseudo_3075 0.377317119 1.460902792
0.496245922 −0.1289434
pseudo_3076 0.772725208 0.828209738 0.010746135
0.69322609
pseudo_3077 0.227044111 −0.266620473
0.2781384 −0.033625934
pseudo_3078 0.83621216 0.838278717
0.227645977 −0.461901283
pseudo_3079 0.206689015 −0.693667673 0.585625263
0.693748502
pseudo_308 0.394444357 0.19475512 0.160959362
0.243954578
pseudo_3080 0.72591789 −0.722026795
0.983474456 −0.223063516
pseudo_3081 0.92682401 0.001804271
0.425072015 −0.172271604
pseudo_3082 0.946717589 1.258324436
0.947962171 −0.036201465
pseudo_3083 0.277792179 −4.404521336 0.429166874
0.372321141
pseudo_3084 0.214377443 0.538179187
0.332240309 −0.209683688
pseudo_3085 0.568542987 −0.231446408 0.413817849
0.187553735
pseudo_3086 0.580236038 0.416255607
0.955305337 −0.060217791
pseudo_3087 0.759598632 −0.452825574 0.853342495
0.127122271
pseudo_3088 0.318779915 0.649608902 0.966644446
0.047918434
pseudo_3089 0.265859031 0.780345334 0.879165709
0.105922417
pseudo_309 0.796153387 −0.235335904 0.942984634
0.314965426
pseudo_3090 0.097271715 0.995227989 0.833771099
0.276625611

pseudo_3091 0.279873923 -1.028345928 0.065660083
0.711316467
pseudo_3092 0.10291782 -1.962885468 0.979110112
0.243646372
pseudo_3093 0.221087769 -1.706153225 0.173942513
0.213076617
pseudo_3094 0.46943541 -0.517645399
0.484935926 -0.150920801
pseudo_3095 0.959791608 -0.386718583
0.503198543 -0.223608482
pseudo_3096 0.999064525 0.154807384
0.564309933 -0.083223825
pseudo_3097 0.371850983 -0.048925764 0.063456558
0.733860073
pseudo_3098 0.275376835 -1.767631514
0.668124302 -0.422557501
pseudo_3099 0.544931886 0.151950549
0.522834633 -0.04273009
pseudo_31 0.772127101 0.479130095 0.491803587
0.293177457
pseudo_310 0.974746393 0.12783285 0.210650243
0.261093504
pseudo_3100 0.88224904 0.415173697
0.581868214 -0.164875788
pseudo_3101 0.837433284 -0.926233402
0.700851831 -0.132261037
pseudo_3102 0.684126911 -0.876356425 0.254609399
0.636819326
pseudo_3103 0.606760618 0.404820844
0.888421011 -0.417548226
pseudo_3104 0.977239862 0.414361597
0.988462877 -0.061200354
pseudo_3105 0.688725652 -1.360667852
0.652834679 -0.097324191
pseudo_3106 0.981603955 0.642890406
0.041618289 -0.901275001
pseudo_3107 0.380280832 -1.30005288
0.810058136 -0.190661538
pseudo_3108 0.096799517 0.649485211
0.497236007 -0.487188085
pseudo_3109 0.908215001 -0.364071156 0.447641458
0.264139036
pseudo_311 0.690453162 0.352352291 0.897073065
0.011397776
pseudo_3110 0.939252931 -2.782427589
0.120680141 -0.28914458
pseudo_3111 0.906356697 1.003218273 0.816122709
0.160188302
pseudo_3112 0.02525929 1.3100549
0.128345195 -0.298632946

pseudo_3113	0.5376868	-0.284415409	
0.301639832	-0.09862053		
pseudo_3114	0.034790356	1.346997083	0.09711411
0.50811694			
pseudo_3115	0.433284573	-1.081300683	
0.237430835	-0.777529732		
pseudo_3116	0.459218705	0.657028091	0.876210929
0.136165093			
pseudo_3117	0.168561679	1.112777433	0.647208228
0.133645011			
pseudo_3118	0.83133164	0.927678231	0.525890122
0.086080841			
pseudo_3119	0.962283232	-0.649123362	
0.465603527	-0.192969225		
pseudo_312	0.596421265	0.492718667	0.502202171
0.304072373			
pseudo_3120	0.125620493	2.09377041	
0.412479303	-0.034117093		
pseudo_3121	0.769138602	0.227596135	
0.850278185	-0.084431035		
pseudo_3122	0.794344384	0.669806809	0.003899658
0.72708098			
pseudo_3123	0.820374496	-1.931080364	
0.059127498	-0.873521906		
pseudo_3124	0.199970677	-1.340858201	
0.365042404	-0.40774959		
pseudo_3125	0.123892839	1.54468642	0.632674147
0.114078941			
pseudo_3126	0.884716992	-0.064578314	0.437886481
0.258450876			
pseudo_3127	0.692960051	-0.245406185	0.667325383
0.433121666			
pseudo_3128	0.561672003	1.35146184	0.521909034
0.177844186			
pseudo_3129	0.427365939	0.707500499	0.472322881
0.232843252			
pseudo_313	0.937387575	0.004889386	0.436042352
0.904805572			
pseudo_3130	0.929308464	0.236266192	
0.449982147	-0.74824247		
pseudo_3131	0.354935551	-0.324276583	
0.038365616	-0.871212937		
pseudo_3132	0.931793607	-1.177122551	
0.067456266	-0.761181493		
pseudo_3133	0.079730001	1.518078027	0.118320027
0.26763077			
pseudo_3134	0.387537755	-2.536765954	0.370179023
0.142842566			
pseudo_3135	0.475215783	0.387473288	
0.200849002	-0.55358079		

pseudo_3136 0.365191226 -0.792798486 0.208662816
0.244988512
pseudo_3137 0.152271447 1.32543718 0.214377441
0.189050212
pseudo_3138 0.828893804 1.090543175 0.983474456
0.14952124
pseudo_3139 0.126006954 -1.577310465
0.921236633 -0.248546133
pseudo_314 0.375208924 -2.644714152
0.411143326 -0.406055222
pseudo_3140 0.046372983 -1.709433591
0.780512767 -0.31529882
pseudo_3141 0.524361239 0.560127544
0.765556928 -0.336198836
pseudo_3142 0.855795541 0.359340924
0.979110112 -0.184331764
pseudo_3143 0.612234355 -0.474564505
0.708397133 -0.13501757
pseudo_3144 0.473767039 -0.70142806
0.482495839 -0.160718802
pseudo_3145 0.95294174 0.683754991 0.886568691
0.198018013
pseudo_3146 0.356972007 2.418235961
0.1588686 -0.234761909
pseudo_3147 0.187169258 1.468255993
0.201952001 -0.317289939
pseudo_3148 0.825848828 0.88113752
0.478120549 -0.968279583
pseudo_3149 0.034589214 2.260654003
0.030891346 -0.807740567
pseudo_315 0.942984634 0.373281836
0.859477714 -0.042574473
pseudo_3150 0.481521677 -1.63122834 0.804609742
0.089000757
pseudo_3151 0.05129628 0.905978829
0.831331638 -0.102191246
pseudo_3152 0.749127054 -0.549179411 0.486662431
0.238661272
pseudo_3153 0.853955625 -1.104853635 0.415606573
0.53356563
pseudo_3154 0.817336959 -2.825058778 0.515740782
0.145813843
pseudo_3155 0.001289264 -1.645946117 0.00048661
0.841737232
pseudo_3156 0.361996109 -3.55199267 0.739150988
0.675592138
pseudo_3157 0.490802243 -1.182974066
0.380512918 -0.307675083
pseudo_3158 0.250396854 -0.833228224 0.532541518
0.038424596

pseudo_3159 0.091725448 1.128492476 0.05367476
0.333178159
pseudo_316 0.367679857 -2.19754085 0.562726458
0.138416728
pseudo_3160 0.134949372 -0.39747584 0.916893567
0.225957635
pseudo_3161 0.33733207 0.547571059 0.825848827
0.670762742
pseudo_3162 0.754842227 -0.358524706 0.964775225
0.270093548
pseudo_3163 0.211786893 -0.145019912
0.850294713 -0.051229007
pseudo_3164 0.087589781 -1.296118042 0.854568842
0.128887442
pseudo_3165 0.142450195 -0.636877988 0.887803503
0.362387794
pseudo_3166 0.910073808 -0.022130144
0.213800951 -0.452573939
pseudo_3167 0.065488372 1.879496762
0.811876329 -0.274755944
pseudo_3168 0.999064525 0.715758869 0.94920688
0.038501096
pseudo_3169 0.391413602 1.181281476
0.158637567 -0.877022252
pseudo_317 0.690453162 -0.219733672 0.000611926
0.80556763
pseudo_3170 0.066292873 -1.788362024
0.778114209 -0.097166794
pseudo_3171 0.528954715 -1.860471159
0.08600678 -0.237408339
pseudo_3172 0.384112274 0.708140326 0.738268969
0.243789823
pseudo_3173 0.455628009 -0.969942642
0.643841983 -0.472314264
pseudo_3174 0.460363188 -0.733550839
0.482008623 -0.1460007
pseudo_3175 0.982227445 -0.195045284
0.228853123 -0.412066303
pseudo_3176 0.957300377 0.493055041
0.239540063 -0.85260137
pseudo_3177 0.025564448 -0.368696464
0.486146285 -0.451019238
pseudo_3178 0.554841418 -0.121383731 0.677822748
0.099997177
pseudo_3179 0.114809963 -2.38738336 0.810664087
0.25917826
pseudo_318 0.755733351 -0.666884299 0.964152193
0.04847194
pseudo_3180 0.305312741 -0.624147068
0.452329757 -0.344053954

pseudo_3181 0.02715549 -0.747602335
0.781112735 -0.09987784
pseudo_3182 0.293304204 -0.384641165
0.900784899 -0.209461455
pseudo_3183 0.518774878 1.609875598
0.75977278 -0.497885714
pseudo_3184 0.311250853 2.487840212 0.790127691
0.639494041
pseudo_3185 0.905737376 0.320643885 0.495256891
0.330274134
pseudo_3186 0.691029358 -0.403491638
0.972876451 -0.182747949
pseudo_3187 0.744174494 0.250403758 0.915033004
0.046148546
pseudo_3188 0.955432225 -1.776595869
0.620485625 -0.278062627
pseudo_3189 0.308643619 -1.240027036 0.918134196
0.129621116
pseudo_319 0.803097971 0.219554948
0.6188315 -0.058942286
pseudo_3190 0.302738604 -0.302043514 0.369761763
0.151364464
pseudo_3191 0.450451118 1.484872603 0.934900968
0.262719821
pseudo_3192 0.442393997 0.977612464
0.36112867 -0.419007149
pseudo_3193 0.460363188 -0.569941785 0.531515463
0.136529578
pseudo_3194 0.09097564 -1.780412859 0.34247371
0.014834587
pseudo_3195 0.351692509 -1.797745897
0.509700332 -0.884438127
pseudo_3196 0.877932887 0.034511452
0.843238921 -0.256801161
pseudo_3197 0.280570188 -0.599872057
0.919995511 -0.58810108
pseudo_3198 0.021230921 -1.773781062
0.859477714 -0.078431764
pseudo_3199 0.257554503 -0.550123664
0.552747897 -0.203866778
pseudo_32 0.467517293 0.286496041
0.094004703 -0.252046723
pseudo_320 0.743583258 0.672834043
0.745949106 -0.201835265
pseudo_3200 0.550136461 0.961687053
0.260854459 -0.494976954
pseudo_3201 0.417399857 -0.872853104 0.128345195
0.339005062
pseudo_3202 0.700851833 0.075635629 0.764960467
0.175759433

pseudo_3203	0.502700229	-0.755574487	0.641042345	0.211942541
pseudo_3204	0.371432556	-0.38252851	0.485913829	-0.371416585
pseudo_3205	0.847828362	-0.713094228	0.068280597	0.566074977
pseudo_3206	0.42054905	1.793214876	0.459413954	0.210853556
pseudo_3207	0.375208924	0.048093438	0.059127498	-0.261018864
pseudo_3208	0.807030097	1.338042103	0.874236208	0.061618123
pseudo_3209	0.148063598	0.655162924	0.515235996	-0.2070402
pseudo_321	0.349546136	0.456304098	0.741810456	-0.251266211
pseudo_3210	0.540284674	-0.153032274	0.296362914	0.5610346
pseudo_3211	0.429623272	-0.252255095	0.644963257	-0.080265531
pseudo_3212	0.951696668	-0.117494318	0.506694053	0.204595011
pseudo_3213	0.747132932	0.643847956	0.588315746	0.33113547
pseudo_3214	0.496740835	-5.570399829	0.654526492	0.230124504
pseudo_3215	0.128149064	-1.190097731	0.871157734	0.169642279
pseudo_3216	0.866851159	-0.587741285	0.075562583	0.788090388
pseudo_3217	0.401424907	-0.378817914	0.766750266	0.146114932
pseudo_3218	0.178565773	0.230548132	0.539235235	0.185545721
pseudo_3219	0.547010779	0.754767944	0.664714403	0.101173258
pseudo_322	0.145020354	-0.590103478	0.220792918	0.33752226
pseudo_3220	0.890891717	0.995229358	0.102095629	-1.118852516
pseudo_3221	0.668693289	-0.306751877	0.538202696	-0.208277686
pseudo_3222	0.659612557	0.39430867	0.114630204	0.820361157
pseudo_3223	0.112844791	1.157313107	0.825848827	-0.066897555
pseudo_3224	0.813695537	0.403599801	0.426434426	-0.352138005
pseudo_3225	0.924961136	0.089294659	0.348066594	0.115625281

pseudo_3226 0.981603955 1.210571674
0.626013215 -0.436024614
pseudo_3227 0.224728443 -1.526972691
0.467763657 -0.082308708
pseudo_3228 0.846604 0.225511131 0.185606238 0.094584383
pseudo_3229 0.60239744 0.351775251
0.808846576 -0.626533864
pseudo_323 0.137001638 0.466904567
0.784715263 -0.114744001
pseudo_3230 0.609494755 0.1991461 0.239610304
0.238844393
pseudo_3231 0.251364634 -3.534359686 0.758408669
0.398819827
pseudo_3232 0.709491137 -0.675460912 0.195666057
0.641541721
pseudo_3233 0.609494755 -0.307163679 0.608947488
0.384334432
pseudo_3234 0.280918761 -1.509020759
0.866851159 -0.263984512
pseudo_3235 0.587777195 -1.834312504
0.461789089 -0.447110144
pseudo_3236 0.698536085 0.36836601 0.737679259
0.281817482
pseudo_3237 0.174437973 1.495810071
0.326042059 -0.530744608
pseudo_3238 0.566424563 0.828648568
0.053288127 -0.827748578
pseudo_3239 0.868081212 -0.086715926 0.397489264
0.354244514
pseudo_324 0.522326274 -0.428069438
0.717138906 -0.323815038
pseudo_3240 0.168320097 -1.079820336
0.356564126 -0.664358029
pseudo_3241 0.828893804 0.420752816 0.997193581
0.073774351
pseudo_3242 0.756030465 0.335116181 0.506694053
0.241198463
pseudo_3243 0.656220082 -1.26750836
0.076143068 -0.809612474
pseudo_3244 0.089196202 0.859519473
0.748317353 -0.302664594
pseudo_3245 0.294840655 0.939735971 0.76794457
0.245502107
pseudo_3246 0.434662201 1.874762936 0.732378849
0.044081389
pseudo_3247 0.772127101 -0.427105795
0.531515463 -0.186184814
pseudo_3248 0.647208229 0.288500716 0.199476445
0.660642074
pseudo_3249 0.267204675 -0.726424985

0.601308856 -1.067719943
pseudo_325 0.672681501 -0.305802079 0.612782927
0.473160448
pseudo_3250 0.077118627 -1.605032933
0.4129252 -0.596821076
pseudo_3251 0.73826897 -0.590469654
0.830722027 -0.334233173
pseudo_3252 0.030051395 -0.464920169 0.365605264
0.63302782
pseudo_3253 0.577588289 0.453997494
0.19405473 -0.274380515
pseudo_3254 0.926203007 -0.330975733
0.289372759 -0.0777045
pseudo_3255 0.645524199 0.445701116
0.384539441 -0.67411497
pseudo_3256 0.256898002 0.095760679 0.102259647
0.457537158
pseudo_3257 0.055643221 -1.420469634
0.065488371 -0.370720006
pseudo_3258 0.47280256 0.746728941 0.63100609
0.172723308
pseudo_3259 0.916893567 0.163621684
0.188216655 -0.613775024
pseudo_326 0.333215618 0.242411059
0.643841983 -0.518558985
pseudo_3260 0.438810227 0.283836723
0.124275149 -0.233415854
pseudo_3261 0.596421265 -0.711051335 0.999064525
0.078764993
pseudo_3262 0.062021764 -1.545058681
0.797963485 -0.29902476
pseudo_3263 0.081944011 -1.16437896
0.269570801 -1.035904725
pseudo_3264 0.429166876 -0.561848816 0.762576021
0.288428839
pseudo_3265 0.074412447 -2.076467408
0.577588287 -0.168062497
pseudo_3266 0.966956001 0.302032663 0.607307005
0.188344115
pseudo_3267 0.842933147 0.858670278 0.768541311
0.328835988
pseudo_3268 0.762284371 0.453329233 0.395920953
0.259068091
pseudo_3269 0.194054732 -1.811193013
0.21815196 -0.595711087
pseudo_327 0.923098674 0.999891738 0.537686798
0.18580377
pseudo_3270 0.471839163 -0.358816491 0.030349096
0.60876187
pseudo_3271 0.593172956 0.316722543 0.173448107

0.595291482
pseudo_3272 0.431451671 -0.87952936
0.816729779 -0.396856164
pseudo_3273 0.643219664 -1.005491654 0.284702573
0.109632923
pseudo_3274 0.347264077 0.174156232 0.501206844
0.377325552
pseudo_3275 0.896454643 0.503467115 0.393143725
0.193189365
pseudo_3276 0.654526493 -0.305613104
0.554841416 -0.422831807
pseudo_3277 0.905118112 0.107989292 0.458939749
0.299730468
pseudo_3278 0.804609743 0.651049299 0.316886561
0.236516522
pseudo_3279 0.692759018 -0.772303118 0.691029357
0.286597549
pseudo_328 0.791331841 -0.573332034 0.039549742
0.895059267
pseudo_3280 0.172290902 1.483319952
0.898345704 -0.141606802
pseudo_3281 0.284420662 -0.531860022
0.76198026 -0.049419318
pseudo_3282 0.070945211 1.736106892
0.984218184 -0.252571911
pseudo_3283 0.586700773 1.201859144
0.486892798 -0.198156562
pseudo_3284 0.015275243 0.691879077
0.708978739 -0.145830822
pseudo_3285 0.893781493 -0.184192259 0.169970872
0.288225663
pseudo_3286 0.824631568 -0.891641417 0.697379259
0.069459091
pseudo_3287 0.158176268 -2.031239985
0.26518796 -0.094106042
pseudo_3288 0.057363364 1.678824602
0.383258805 -0.238650044
pseudo_3289 0.266195004 -1.175229422 0.908834548
0.332381085
pseudo_329 0.633787225 -0.091726605 0.134135148
0.164100399
pseudo_3290 0.471839163 1.172086676 0.471357868
0.096485199
pseudo_3291 0.012200393 1.841963351
0.066524219 -0.400243436
pseudo_3292 0.268554994 0.619016464
0.711306858 -0.377096244
pseudo_3293 0.6261848 -2.675792727
0.766056445 -0.168424584
pseudo_3294 0.69969361 -0.60305666 0.339106223

0.036411341			
pseudo_3295	0.032315024	2.317003916	
0.777514899	-0.015569224		
pseudo_3296	0.45374164	-0.375310042	
0.383685393	-0.612839941		
pseudo_3297	0.044518228	-0.842116094	
0.676107316	-0.1676243		
pseudo_3298	0.168320097	-0.65878667	
0.77332345	-0.39116612		
pseudo_3299	0.22584378	0.985483537	0.439272517
0.172901159			
pseudo_33	0.785917137	-0.431312668	
0.838043992	-0.031187967		
pseudo_330	0.484447376	1.715597906	
0.655090824	-0.03457647		
pseudo_3300	0.762576022	0.683518912	0.70723443
0.1648264			
pseudo_3301	0.01877499	2.066916067	
0.033533156	-0.902017133		
pseudo_3302	0.685710173	-0.544334746	0.21726575
0.531079858			
pseudo_3303	0.600221164	0.450713866	
0.041696628	-0.382297301		
pseudo_3304	0.00381337	1.814789929	0.324504318
0.508463825			
pseudo_3305	0.021057987	2.021671654	0.856759755
0.150676778			
pseudo_3306	0.990333661	-0.353737896	0.480305477
0.23059958			
pseudo_3307	0.739448853	1.294892019	
0.19623605	-0.081747273		
pseudo_3308	0.487872833	-1.500027039	0.165441359
0.295117731			
pseudo_3309	0.560618503	-1.274995517	0.165203147
0.114129784			
pseudo_331	0.706653336	0.488972512	0.799774671
0.471994495			
pseudo_3310	0.958545942	-0.558483686	
0.674964628	-0.164639769		
pseudo_3311	0.376051327	0.709360991	0.878549261
0.062891356			
pseudo_3312	0.190324415	0.590438893	
0.701431204	-0.638452039		
pseudo_3313	0.739448853	0.775402304	
0.254283617	-0.726294904		
pseudo_3314	0.413371385	-0.890208107	0.593713776
0.497174852			
pseudo_3315	0.980980476	-1.028818005	0.895527127
0.006269987			
pseudo_3316	0.106767929	1.911188198	

0.58401372 -0.51875647
pseudo_3317 0.987839296 -0.542081203 0.766153526
0.174613742
pseudo_3318 0.238363174 0.8835445
0.938631108 -0.433110065
pseudo_3319 0.099821287 -1.720346465 0.211792046
0.238817177
pseudo_332 0.449045042 -0.795079442
0.091424929 -0.565499985
pseudo_3320 0.222269986 -0.180868652 0.874852133
0.139705442
pseudo_3321 0.013259256 2.732108318
0.555104104 -0.119946636
pseudo_3322 0.031751374 -1.372450596 0.265859029
0.270575122
pseudo_3323 0.93303643 0.709728264 0.022630113
0.825434329
pseudo_3324 0.374157559 0.551350185
0.135972514 -0.546433652
pseudo_3325 0.224099643 2.229961034 0.603013483
0.075329327
pseudo_3326 0.450451118 0.709265659 0.795550263
0.494458764
pseudo_3327 0.127171893 1.287720473 0.406708659
0.924532952
pseudo_3328 0.954809562 0.57643716
0.447174153 -0.056647165
pseudo_3329 0.931172258 0.980508465 0.753654568
0.289785648
pseudo_333 0.422806991 -2.718520922
0.685850076 -0.063969412
pseudo_3330 0.159099888 1.361688417 0.90759551
0.263176182
pseudo_3331 0.653294282 -0.479464145 0.318626094
0.104991062
pseudo_3332 0.870542273 -0.760692175 0.941118621
0.177323423
pseudo_3333 0.923719448 -0.192690738 0.689877145
0.001254142
pseudo_3334 0.839265703 -2.052930301
0.111256722 -0.299006522
pseudo_3335 0.222862789 -0.373884381
0.758408669 -0.53142757
pseudo_3336 0.638247806 -0.637746417 0.511206965
0.152667928
pseudo_3337 0.995322652 -0.50932201 0.482008623
0.116736052
pseudo_3338 0.44764146 0.396290797
0.697957584 -0.425203077
pseudo_3339 0.764960468 1.289013652 0.875468133

0.242123666
pseudo_334 0.121618211 −0.776836827 0.915033004
0.278838368
pseudo_3340 0.938009322 −2.344839005
0.670401385 −0.152002838
pseudo_3341 0.786518265 0.281017821 0.016784481
0.229393768
pseudo_3342 0.539235237 −0.546243579
0.322971306 −0.155855138
pseudo_3343 0.622141673 −1.251192546 0.76198026
0.105899525
pseudo_3344 0.476666955 −1.02630646
0.789525805 −0.492927123
pseudo_3345 0.564838235 −0.586895213 0.574920881
0.393958964
pseudo_3346 0.334975826 0.3932758
0.811876329 −0.184421773
pseudo_3347 0.682979045 0.39297375
0.360656128 −0.203006357
pseudo_3348 0.057465911 −1.462386076 0.073527819
0.903955076
pseudo_3349 0.563254045 0.990272651
0.295824605 −0.90470473
pseudo_335 0.523343249 −0.937458431
0.262848412 −0.753249174
pseudo_3350 0.545970842 0.444702537 0.210365489
0.295666679
pseudo_3351 0.37101442 1.02520479 0.393143725
0.21452832
pseudo_3352 0.209512902 −0.878183747 0.049729831
0.748194301
pseudo_3353 0.716554953 0.005026269
0.086723436 −0.668750253
pseudo_3354 0.595879318 0.591706418 0.96166029
0.344070375
pseudo_3355 0.008272644 −1.774137184
0.653962357 −0.160878929
pseudo_3356 0.579191524 0.46249667 0.715387541
0.268291767
pseudo_3357 0.915653141 −2.397996409
0.915653141 −0.278567872
pseudo_3358 0.953564319 0.065455236 0.490327565
0.378618015
pseudo_3359 0.951696668 −0.535191037
0.266867823 −0.035475992
pseudo_336 0.070074924 −3.2544709 0.764364145
0.155601082
pseudo_3360 0.32450432 2.150690177
0.668977851 −0.236047382
pseudo_3361 0.997817228 −0.53781603 0.934900968

0.352455312
pseudo_3362 0.385394652 −0.159411986 0.289017123
0.380590949
pseudo_3363 0.73107755 −1.093542145 0.537562209
0.050618477
pseudo_3364 0.395747586 −0.328730858 0.796756631
0.209080983
pseudo_3365 0.295463666 −0.774532425 0.639365007
0.122467063
pseudo_3366 0.817336959 0.98447759 0.117716339
0.69825187
pseudo_3367 0.16735637 −0.682714012 0.17718184
0.277212947
pseudo_3368 0.51221268 0.262391639
0.448109039 −0.747861576
pseudo_3369 0.421000071 −1.069539431 0.866236253
0.191941413
pseudo_337 0.75840867 −0.358691368
0.533568579 −0.454783824
pseudo_3370 0.072155083 0.526872427
0.27126967 −0.55537906
pseudo_3371 0.742992173 0.989091331
0.666418491 −0.45328954
pseudo_3372 0.111608094 −3.971585218 0.531002811
0.241228772
pseudo_3373 0.35738018 −0.667720434
0.814302163 −0.340667284
pseudo_3374 0.747132932 −0.189605518 0.522834633
0.236235285
pseudo_3375 0.473284664 −0.665085773 0.35048122
0.339968764
pseudo_3376 0.036646004 1.447002165
0.929308464 −0.151328371
pseudo_3377 0.892745443 −0.752347334 0.28442066
1.075536838
pseudo_3378 0.632117921 −2.044334656
0.985345049 −0.289226612
pseudo_3379 0.770333593 0.684232589 0.812482619
0.208120784
pseudo_338 0.279873923 −0.338208549
0.8149089 −0.31847174
pseudo_3380 0.685275507 −0.252421064 0.259530983
0.638425906
pseudo_3381 0.730457735 1.138826758 0.048761802
0.282419072
pseudo_3382 0.581868215 −2.276398085
0.038732375 −0.59884895
pseudo_3383 0.822198315 0.723351586 0.414264602
0.279215862
pseudo_3384 0.597505832 −0.324382099

0.177684161 -0.406148029
pseudo_3385 0.140791453 -2.921349157
0.039114602 -0.686240102
pseudo_3386 0.189795852 0.655158309 0.672681499
0.18336732
pseudo_3387 0.429623272 -2.615507152
0.942362594 -0.432144235
pseudo_3388 0.219616344 -0.638605004
0.997817228 -0.026412991
pseudo_3389 0.655090826 0.421396245 0.470396096
0.230111538
pseudo_339 0.319919475 -0.875906991
0.889038586 -0.300491423
pseudo_3390 0.325272598 0.570858437 0.358197403
0.254970891
pseudo_3391 0.331072377 -0.808061953 0.352096858
0.08910599
pseudo_3392 0.913172908 -0.808325837 0.899547376
0.290652198
pseudo_3393 0.313872573 -0.858117916
0.252011267 -0.298967538
pseudo_3394 0.71889176 -2.73920249
0.957923146 -0.145283098
pseudo_3395 0.460838215 1.135401548
0.496740833 -0.764010033
pseudo_3396 0.322971308 -0.582441701
0.380705383 -0.061767271
pseudo_3397 0.146318682 -1.977208696
0.164015958 -0.613356881
pseudo_3398 0.431451671 -1.259399278 0.892127468
0.266312467
pseudo_3399 0.756010149 -0.929001493
0.875561282 -0.180666026
pseudo_34 0.334975826 0.604912939
0.418298203 -0.06355589
pseudo_340 0.875468134 -1.061165509 0.709560515
0.211327725
pseudo_3400 0.500709573 -0.520352603 0.986592156
0.163626035
pseudo_3401 0.88224904 0.979757568 0.793741628
0.181580503
pseudo_3402 0.782913418 0.855800479
0.770931292 -0.155756731
pseudo_3403 0.87608421 -0.367216628
0.23588267 -0.856839175
pseudo_3404 0.022671803 -0.602530482 0.048479484
0.463014352
pseudo_3405 0.171971192 -1.451514237
0.263849325 -0.19375304
pseudo_3406 0.462264939 -3.601753044

0.483471072 -0.182611823
pseudo_3407 0.034861907 1.783422093
0.516033542 -0.187133107
pseudo_3408 0.599134362 -0.276683597
0.469915616 -0.287337553
pseudo_3409 0.810451994 1.269186165 0.627938008
0.163821931
pseudo_341 0.243695234 -0.374946818
0.667555508 -0.942328828
pseudo_3410 0.598048452 -0.488871696 0.148282824
0.314978233
pseudo_3411 0.507194453 -1.666996329
0.152495407 -0.444267296
pseudo_3412 0.851503641 1.188404433
0.143704083 -0.361960574
pseudo_3413 0.379856571 -1.12293967 0.917513856
0.295487135
pseudo_3414 0.308643619 2.057807174
0.626567138 -0.6423421
pseudo_3415 0.487872833 0.587903452 0.421000069
0.011374341
pseudo_3416 0.346062507 -2.82905154
0.919375024 -0.437108592
pseudo_3417 0.769204089 -0.566841918
0.727361306 -0.257618765
pseudo_3418 0.042566637 0.728033116 0.455628007
0.260810233
pseudo_3419 0.567483303 0.355242417 0.647208228
0.371069152
pseudo_342 0.450920363 0.717841428
0.081532759 -0.663115265
pseudo_3420 0.024354937 -3.366005296
0.753060957 -0.166053862
pseudo_3421 0.260523154 0.537580066
0.32182465 -0.236257822
pseudo_3422 0.0906771 -1.403947373
0.951696667 -0.451762204
pseudo_3423 0.102752962 -3.773742963
0.221974006 -0.167575112
pseudo_3424 0.957923146 0.569676281 0.435121969
0.169779816
pseudo_3425 0.242118355 -0.382978467 0.550658261
0.175974787
pseudo_3426 0.897693465 0.133850679
0.543014015 -0.852722471
pseudo_3427 0.526400258 0.605895846 0.897073064
0.054573771
pseudo_3428 0.911313286 -0.056134443
0.874236208 -0.175381708
pseudo_3429 0.4868928 0.675118952 0.911933106

0.024333022
pseudo_343 0.560618503 -1.233205666 0.727678058
0.327236623
pseudo_3430 0.410254105 1.070933727 0.144159715
0.30753731
pseudo_3431 0.567483303 0.656081059 0.735321964
0.040123533
pseudo_3432 0.945473136 -0.276347016 0.294022842
0.137971381
pseudo_3433 0.37478816 -0.439425801 0.589393537
0.16120771
pseudo_3434 0.659612557 -0.40346152
0.94174059 -0.281441601
pseudo_3435 0.882865922 -0.438431042 0.427344113
0.121590489
pseudo_3436 0.100953356 1.032676003
0.392278084 -0.058813676
pseudo_3437 0.467517293 0.768406524 0.450451116
0.134077493
pseudo_3438 0.83133164 -0.296463345 0.350884688
0.158099283
pseudo_3439 0.647489076 0.190197789
0.931793607 -0.484471917
pseudo_344 0.847828362 -2.055136198
0.082425927 -1.232188187
pseudo_3440 0.913172908 0.126979589 0.505194416
0.121916864
pseudo_3441 0.81551575 1.040749598
0.953564319 -0.31877002
pseudo_3442 0.224647986 0.382066316 0.509700332
0.86886655
pseudo_3443 0.57865688 -0.51792785
0.894599754 -0.387233631
pseudo_3444 0.547531115 2.513677898 0.95294174
0.297939758
pseudo_3445 0.045285998 0.790331415 0.249224395
0.629686533
pseudo_3446 0.979110112 -0.808626394 0.255915428
0.199482083
pseudo_3447 0.881015491 -0.30516411
0.317643015 -0.00639391
pseudo_3448 0.163542889 -0.477693977 0.670401385
0.167074612
pseudo_3449 0.202505155 -1.048791296 0.179450634
0.154868054
pseudo_345 0.625459503 0.947172802 0.226443375
0.14595232
pseudo_3450 0.400548304 -0.58573793
0.743583257 -0.401248125
pseudo_3451 0.361066948 0.79684203

0.188216655 -0.230429237
pseudo_3452 0.961660291 -0.733362574
0.310131692 -0.246227803
pseudo_3453 0.804609743 0.214710952
0.050278052 -0.16289031
pseudo_3454 0.775118985 -0.277501228 0.358606458
0.068317658
pseudo_3455 0.249902227 -0.311865485 0.96166029
0.169608
pseudo_3456 0.065832167 1.652268494 0.23681071
0.909951522
pseudo_3457 0.228853125 0.89818345
0.130916204 -0.17014195
pseudo_3458 0.336938625 -0.751731126 0.111960338
0.550312825
pseudo_3459 0.747258065 0.569139161 0.540144672
0.217165782
pseudo_346 0.680685514 1.793933155
0.333020408 -1.361175013
pseudo_3460 0.843544721 -0.447517335
0.70607241 -0.078123809
pseudo_3461 0.153844447 1.256222446
0.721231197 -0.292115502
pseudo_3462 0.6056685 0.12339451
0.601853036 -0.436094751
pseudo_3463 0.740039025 0.360773453 0.897073065
0.183386557
pseudo_3464 0.062460352 1.652754486
0.745357418 -0.533430428
pseudo_3465 0.527421284 0.165610828 0.240862029
0.655251952
pseudo_3466 0.268554994 1.051217679 0.234341661
0.32821833
pseudo_3467 0.791331841 -0.398357264 0.683552885
0.156120953
pseudo_3468 0.584550673 0.525496979 0.906976075
0.258070982
pseudo_3469 0.060107229 -1.646132872
0.658057384 -0.224859717
pseudo_347 0.307530667 1.386499569 0.105080168
0.667791681
pseudo_3470 0.399672853 1.079677771 0.917513856
0.27720861
pseudo_3471 0.899547377 1.254322034
0.524870612 -0.604227522
pseudo_3472 0.401863639 -1.683128959 0.642721518
0.047053024
pseudo_3473 0.695645342 0.243410705 0.8149089
0.834652425
pseudo_3474 0.916634703 0.760861483

0.754130993 -0.182281605
pseudo_3475 0.513219423 0.287698544 0.177935719
0.471437403
pseudo_3476 0.766750267 0.232322576 0.644402519
0.090037578
pseudo_3477 0.303840033 1.324981928
0.136177859 -0.072149049
pseudo_3478 0.377832602 1.078956906
0.151070357 -0.280710348
pseudo_3479 0.24943168 -1.792412192 0.077511688
0.374160714
pseudo_348 0.599134362 -0.478977298 0.869311584
0.12259929
pseudo_3480 0.643841984 -0.291475167
0.501704377 -0.096583068
pseudo_3481 0.59263236 0.20097231
0.168078774 -0.043354255
pseudo_3482 0.404061615 -0.293518249 0.78832241
0.104164017
pseudo_3483 0.670971131 0.775866603 0.638247805
0.204432958
pseudo_3484 0.647769975 1.632799882 0.931793607
0.056090167
pseudo_3485 0.790729704 0.567023575
0.562726458 -0.182997448
pseudo_3486 0.483959091 -0.803660884 0.859477714
0.408573784
pseudo_3487 0.849665593 1.001240498 0.275721002
0.461055885
pseudo_3488 0.674393566 -0.062904363
0.206408152 -0.609778627
pseudo_3489 0.555365403 0.117387885
0.456100288 -0.42036661
pseudo_349 0.789525806 0.20072225 0.506193914
0.20212126
pseudo_3490 0.29446091 1.554178291
0.00527201 -0.965963739
pseudo_3491 0.292567103 -1.484622342 0.045999858
0.992638093
pseudo_3492 0.240548669 0.262815987
0.435121969 -0.264835901
pseudo_3493 0.301091445 -0.679420404
0.820982328 -0.109493969
pseudo_3494 0.005070317 -1.921298026
0.820982328 -0.065345225
pseudo_3495 0.394444357 -0.277544342
0.32565718 -0.280333955
pseudo_3496 0.122183783 -1.346032822
0.414711642 -0.75123095
pseudo_3497 0.91441292 0.827003304 0.019578128

0.525885893
pseudo_3498 0.112490351 -3.529539235
0.70607241 -0.131198629
pseudo_3499 0.753654569 -0.067649383 0.228853123
0.084468897
pseudo_35 0.51221268 0.165064915 0.527421282
0.20512661
pseudo_350 0.64048303 -0.63760042 0.283717927
0.705872912
pseudo_3500 0.351288452 1.445830427 0.593713776
0.182290655
pseudo_3501 0.257226107 1.122079632
0.915033004 -0.240237635
pseudo_3502 0.847828362 -4.23299932 0.510704496
0.088798245
pseudo_3503 0.168561679 -0.407303128 0.502202171
0.236406887
pseudo_3504 0.234312031 -1.676054735
0.75977278 -0.510543226
pseudo_3505 0.359015808 -1.114955543
0.627121272 -0.360406503
pseudo_3506 0.237120631 0.491571038
0.09082627 -0.279589011
pseudo_3507 0.797963486 0.367796093
0.972253169 -0.113612027
pseudo_3508 0.956054916 0.188916474 0.05839571
0.289034401
pseudo_3509 0.602942064 -0.05957149 0.09477447
0.871533368
pseudo_351 0.457045678 -0.172214042
0.340886356 -0.588879936
pseudo_3510 0.793741629 0.36629944
0.426889128 -0.509693405
pseudo_3511 0.321443026 0.939683269 0.374788157
0.042179379
pseudo_3512 0.43236756 -1.182720519 0.792536486
0.183727379
pseudo_3513 0.462741061 -1.956579546
0.521310315 -0.365817245
pseudo_3514 0.773921828 -0.931173979 0.730614865
0.20761798
pseudo_3515 0.500709574 -0.930708123
0.312372672 -0.872589297
pseudo_3516 0.021988593 2.242366178 0.937387574
0.075904447
pseudo_3517 0.772725208 -0.711461917
0.572259315 -0.312627664
pseudo_3518 0.88224904 -0.56319938 0.051953188
0.76798825
pseudo_3519 0.242433155 -0.55584223 0.810058136

0.491438068
pseudo_352 0.508196028 -0.218829161 0.214089056
0.717149735
pseudo_3520 0.152944084 -0.743558973
0.107449017 -0.635324667
pseudo_3521 0.538202697 -0.276692435 0.11498994
0.691491301
pseudo_3522 0.028543484 -1.486718166 0.660178652
0.636960276
pseudo_3523 0.16544136 -0.913411249
0.865006685 -0.016767605
pseudo_3524 0.432825926 -1.479448033 0.966644446
0.155375695
pseudo_3525 0.852116503 0.903167716
0.633787224 -0.220210392
pseudo_3526 0.445773903 -1.473125433
0.910073807 -0.097171869
pseudo_3527 0.724745246 0.403842879
0.803400266 -0.309147801
pseudo_3528 0.59263236 0.581135057 0.900166107
0.291462089
pseudo_3529 0.243514699 -1.134046381 0.414051406
0.270993013
pseudo_353 0.289728691 0.883391951
0.843544721 -0.141560825
pseudo_3530 0.113200109 -1.038352263 0.869926889
0.082155312
pseudo_3531 0.775717764 -0.156564293 0.037069631
0.18572919
pseudo_3532 0.21669461 0.503586961
0.509198638 -0.28657988
pseudo_3533 0.458939751 -2.590610446
0.859477714 -0.112178092
pseudo_3534 0.877316587 -0.274533739 0.496245922
0.568189534
pseudo_3535 0.284069149 1.811941834
0.245914961 -0.014129753
pseudo_3536 0.357788646 -1.919210726 0.032189032
0.600426601
pseudo_3537 0.722987492 -0.882412486 0.614429947
0.283619241
pseudo_3538 0.483959091 -2.436609674 0.76198026
0.285180223
pseudo_3539 0.64973119 0.57565591
0.778037592 -0.603103613
pseudo_354 0.149603401 0.452242008
0.908834548 -0.257083366
pseudo_3540 0.692182286 -0.431970821
0.132518107 -0.637362887
pseudo_3541 0.171236272 0.34246451 0.640483028

0.310491237
pseudo_3542	0.663012048	2.25784368
0.978486682	-0.099213973
pseudo_3543	0.172954754	0.488639004	0.865006685
0.304486323
pseudo_3544	0.344895697	-0.676576836	0.921214241
0.200759308
pseudo_3545	0.439272519	-0.634508751
0.816122709	-0.171420456
pseudo_3546	0.280918761	0.601034731	0.622141671
0.24351148
pseudo_3547	0.301639834	0.611471983	0.595337594
0.152541399
pseudo_3548	0.466081562	0.711491722
0.274346085	-0.237649091
pseudo_3549	0.120120025	-0.168689345	0.742992172
0.363216313
pseudo_355	0.701431205	0.251954115	0.536140597
0.267216884
pseudo_3550	0.340490257	-0.159340701	0.764960467
0.23458473
pseudo_3551	0.75900358	-0.040491314
0.207533263	-0.360841633
pseudo_3552	0.072403099	-2.196881716	0.273660387
0.648720699
pseudo_3553	0.5376868	-0.249335573
0.959791608	-0.156912899
pseudo_3554	0.966021352	0.679813063	0.387108552
0.601458251
pseudo_3555	0.533054926	-0.628531141	0.52946636
0.193849525
pseudo_3556	0.544931886	-0.968699808
0.411588366	-0.443259748
pseudo_3557	0.561145133	-0.007528401	0.955432225
0.075490749
pseudo_3558	0.773323451	0.654739944
0.031441893	-0.846334508
pseudo_3559	0.906976075	-0.652087461
0.216985517	-0.283464445
pseudo_356	0.341282754	-2.381749925	0.456336531
0.013425362
pseudo_3560	0.295103018	-1.046937643	0.51372318
0.256890378
pseudo_3561	0.957300377	0.26947066
0.721231197	-0.217990748
pseudo_3562	0.277100613	1.579558162
0.855182147	-0.356060359
pseudo_3563	0.112136791	-2.051699643	0.180720371
0.497664404
pseudo_3564	0.076857488	-1.539913149

0.252983391 -0.258238275
pseudo_3565 0.255588487 -0.658079402
0.043208969 -0.406171076
pseudo_3566 0.867466146 -0.121241639
0.982227445 -0.120154733
pseudo_3567 0.493775321 0.500211507 0.80219126
0.501922224
pseudo_3568 0.762912661 0.859072182
0.368982523 -0.277915002
pseudo_3569 0.391413602 -0.331471914 0.721231197
0.073803868
pseudo_357 0.475215783 1.917246965 0.577588287
0.300496492
pseudo_3570 0.176180375 0.369704543 0.875468133
0.159872948
pseudo_3571 0.783513905 -0.7614153 0.047411802
0.111606244
pseudo_3572 0.617179305 -1.153151812
0.343468201 -0.822726758
pseudo_3573 0.538650369 -0.940206055
0.944804138 -0.296658669
pseudo_3574 0.877932887 -0.189646544
0.203059408 -0.554833007
pseudo_3575 0.614429948 -0.404698532 0.902641636
0.242270948
pseudo_3576 0.692759018 0.457742618 0.626567138
0.675588565
pseudo_3577 0.302005796 -2.746571306
0.422806989 -0.007375462
pseudo_3578 0.651707796 -1.186721435 0.346863257
0.145620445
pseudo_3579 0.582940508 -0.662276152 0.115712077
0.788905043
pseudo_358 0.367264354 2.085788755 0.650018959
0.282961325
pseudo_3580 0.035949123 1.607705342 0.977863265
0.040376633
pseudo_3581 0.623246768 1.457593388 0.646085339
0.540617638
pseudo_3582 0.02753813 0.97287909 0.776915721
0.152422652
pseudo_3583 0.662834409 0.266691667
0.028952181 -0.872743039
pseudo_3584 0.410254105 -1.494176763
0.120493208 -0.988447372
pseudo_3585 0.506694055 0.295387024
0.460838213 -0.16814341
pseudo_3586 0.877510471 -0.550238054
0.529436381 -0.025340371
pseudo_3587 0.585625265 0.478779487 0.234034317

0.444546572
pseudo_3588 0.572791161 -0.350527195 0.346863257
0.323145809
pseudo_3589 0.347264077 0.473374317 0.80219126
0.170336912
pseudo_359 0.089846299 2.068819009
0.90944393 -0.632779613
pseudo_3590 0.896454643 -1.224678944 0.244961918
0.325639099
pseudo_3591 0.672111189 0.437056102
0.32488831 -0.268306364
pseudo_3592 0.132920946 0.857911074 0.32488831
0.135605367
pseudo_3593 0.639923917 -0.872762483
0.288306736 -0.176513229
pseudo_3594 0.232196253 -0.713168521
0.871157734 -0.500950422
pseudo_3595 0.498723106 0.154121739
0.123321091 -0.253516679
pseudo_3596 0.153844446 0.665863815 0.01261098
0.695735928
pseudo_3597 0.056649788 1.723845512
0.448576902 -0.078160843
pseudo_3598 0.923719448 0.337437519 0.368511741
0.256261103
pseudo_3599 0.070303695 -2.249414974 0.397758115
0.195074767
pseudo_36 0.455156003 -0.860032008 0.966644446
0.252784888
pseudo_360 0.134847385 1.721021645
0.775717763 -0.283034461
pseudo_3600 0.379856571 2.78667701
0.055543388 -0.693411032
pseudo_3601 0.884099898 0.291105127
0.895836283 -0.211236319
pseudo_3602 0.036646004 2.403069996
0.508697203 -0.18519954
pseudo_3603 0.201952002 -0.998842326
0.524361237 -0.379097913
pseudo_3604 0.468236076 0.776297499 0.648331919
0.315550693
pseudo_3605 0.451389885 0.637361815
0.117533016 -0.51363534
pseudo_3606 0.876210929 0.956198935 0.547944075
0.307963587
pseudo_3607 0.859348493 0.585775756 0.212693033
0.622766124
pseudo_3608 0.446240376 -1.045475496
0.101441648 -0.556190359
pseudo_3609 0.622694114 0.219444967 0.804609742

0.268216917
pseudo_361	0.673252001	0.145328705	0.899547376
0.034212563
pseudo_3610	0.744174494	-0.743146301
0.523852115	-0.179087409
pseudo_3611	0.181485411	0.742284884	0.892127468
0.261323397
pseudo_3612	0.619774481	0.606964712	0.252284774
0.48878749
pseudo_3613	0.247828839	-0.965943076	0.010674053
0.607596934
pseudo_3614	0.341679445	0.463494538	0.2095129
0.467673045
pseudo_3615	0.357788646	0.822670747	0.934279415
0.274102561
pseudo_3616	0.017218172	-0.624171243	0.053868953
0.511938183
pseudo_3617	0.265859031	0.522291474
0.674393564	-0.002974343
pseudo_3618	0.457518784	-1.376443391
0.214666106	-0.279308285
pseudo_3619	0.010842917	1.871991298	0.531515463
0.070037342
pseudo_362	0.962906197	-0.540375361	0.612782927
0.238440097
pseudo_3620	0.237120631	-1.367264347	0.977863265
0.092990114
pseudo_3621	0.280918761	-0.51847864
0.983474456	-0.17915514
pseudo_3622	0.248789676	-0.811534494	0.329907101
0.522094452
pseudo_3623	0.057774467	-0.174149268	0.270929312
0.417756395
pseudo_3624	0.506694055	0.467855264	0.384539441
0.181860561
pseudo_3625	0.365605266	-1.037012759	0.001444889
0.884315498
pseudo_3626	0.376472966	-0.381836866
0.643281649	-0.503977662
pseudo_3627	0.465603529	-0.392768322
0.247189723	-0.466201178
pseudo_3628	0.545970842	-0.041191537
0.377739631	-0.335496069
pseudo_3629	0.790729704	0.491366348	0.372688715
0.001286427
pseudo_363	0.039102109	-1.643739199	0.072155082
0.173404567
pseudo_3630	0.85824998	0.540654977
0.553270914	-0.40424075
pseudo_3631	0.306420374	0.44137517	0.658480948

0.471084823
pseudo_3632 0.882711908 0.896014973
0.09413933 -0.586275211
pseudo_3633 0.322206576 -0.827135721
0.462264937 -0.468386422
pseudo_3634 0.577588289 -2.171166742
0.683552885 -0.157742616
pseudo_3635 0.997193581 0.413439207
0.407593301 -1.243191429
pseudo_3636 0.365915709 -1.49568164
0.820050573 -0.263644986
pseudo_3637 0.113542526 -1.709110931
0.722416287 -0.24570127
pseudo_3638 0.31481241 -0.695841676 0.302005793
0.852914576
pseudo_3639 0.269909993 0.688996621
0.411143326 -0.208727302
pseudo_364 0.108477099 1.513657099 0.467038442
0.252129373
pseudo_3640 0.850890868 0.834054403
0.885334155 -0.308907672
pseudo_3641 0.417399857 -0.491170604
0.617179303 -0.197722629
pseudo_3642 0.820982328 -0.625417242
0.018814451 -0.642749631
pseudo_3643 0.690453162 -0.318508888 0.30975923
0.054245238
pseudo_3644 0.481034998 1.381323378
0.216113634 -0.029328586
pseudo_3645 0.636573542 0.921349336
0.327198469 -0.276866123
pseudo_3646 0.18482836 -1.378612877
0.969137025 -0.172479548
pseudo_3647 0.444376152 0.53537407 0.329519267
0.460167054
pseudo_3648 0.936144194 -0.161853816 0.469915616
0.379881306
pseudo_3649 0.288661784 0.796533495
0.490327565 -0.307255833
pseudo_365 0.568542988 -2.270790721
0.911313286 -0.134040109
pseudo_3650 0.900302834 0.700879928 0.495482055
0.101216361
pseudo_3651 0.69969361 0.167861593 0.073026191
0.744150748
pseudo_3652 0.577588289 -1.378968893 0.715971163
0.188461357
pseudo_3653 0.440661071 -0.467929198
0.440197939 -0.608275602
pseudo_3654 0.774520339 0.432171074

0.663579306	−0.301866051
pseudo_3655	0.274346087	2.578675403
0.747253283	−0.256531733
pseudo_3656	0.830112518	−1.239831674	0.626013215
0.554859581
pseudo_3657	0.995322652	−0.630138658	0.408036053
0.260000683
pseudo_3658	0.684701118	0.836713373
0.751874174	−0.221886321
pseudo_3659	0.317427729	−0.781146862	0.314246547
0.135759712
pseudo_366	0.201399953	−0.998995778	0.307160271
0.243816555
pseudo_3660	0.0255156	1.501537185	0.661311428
0.333934342
pseudo_3661	0.783731538	0.92412715
0.439735088	−0.188197779
pseudo_3662	0.014253957	−2.361634223	0.779313226
0.230388038
pseudo_3663	0.397053414	−1.448639749
0.962283232	−0.260018171
pseudo_3664	0.153618979	−2.832310787	0.740039024
1.258644017
pseudo_3665	0.828284602	−0.363816921	0.073905886
0.596245653
pseudo_3666	0.267204675	−0.292016176
0.624906001	−0.121249174
pseudo_3667	0.541303289	0.257068626
0.839265702	−0.146591579
pseudo_3668	0.873004588	0.790858164
0.997817228	−0.150577278
pseudo_3669	0.005678413	2.459033685	0.097271714
0.538791483
pseudo_367	0.005802098	−1.651261179
0.667555508	−0.335844022
pseudo_3670	0.289017125	−0.496594804	0.501704377
0.326726784
pseudo_3671	0.202228441	0.451645396	0.464648279
0.093032753
pseudo_3672	0.801586935	−0.276729217	0.03588006
0.250078273
pseudo_3673	0.156798458	1.038873986	0.460838213
0.43100404
pseudo_3674	0.707234431	−0.460534052
0.663012046	−0.180522679
pseudo_3675	0.309759232	1.47842473
0.308643617	−1.102405719
pseudo_3676	0.805214657	0.83544207
0.966021351	−0.158813652
pseudo_3677	0.000588358	2.391907406	0.716554951

0.223148228
pseudo_3678 0.705491658 0.286439231 0.587777193
0.233302796
pseudo_3679 0.626013216 -0.653857676 0.224349744
0.537807952
pseudo_368 0.412479305 1.260901731 0.134135148
0.9749896
pseudo_3680 0.835601746 0.357661806 0.74476588
0.399851364
pseudo_3681 0.808846577 0.431814304
0.481521676 -0.037992562
pseudo_3682 0.568542988 0.556552867
0.136383444 -0.375144272
pseudo_3683 0.385394652 -0.716286788 0.232196251
0.459328573
pseudo_3684 0.692182286 -0.375273544
0.80037864 -0.061115638
pseudo_3685 0.419727332 -0.978129076 0.49809221
0.060701676
pseudo_3686 0.624352712 1.494636571 0.918754585
0.059067219
pseudo_3687 0.752467494 -0.250916253 0.947962171
0.18677567
pseudo_3688 0.988462877 0.692099568
0.318779913 -0.832304105
pseudo_3689 0.218496998 -2.874253056
0.103043846 -0.423711461
pseudo_369 0.154382169 0.252233068 0.583061064
0.707971601
pseudo_3690 0.525380242 -0.110074878
0.199750407 -0.864358588
pseudo_3691 0.762576022 0.947818826 0.67097113
0.159154795
pseudo_3692 0.827675502 -2.244513966 0.777514898
0.208414349
pseudo_3693 0.881632229 -0.795403218
0.252011267 -0.110325608
pseudo_3694 0.94485096 0.798487318 0.12736686
0.557306203
pseudo_3695 0.742401239 -0.101452494
0.987215722 -0.38577313
pseudo_3696 0.569603618 -0.514230525 0.225245315
0.744262845
pseudo_3697 0.641042347 -2.24418282 0.790127692
0.109624646
pseudo_3698 0.356156543 1.578043934
0.099982387 -0.270415447
pseudo_3699 0.277100613 1.6985411 0.368928123
0.203235271
pseudo_37 0.197294629 1.744516856 0.983474456

0.197411934
pseudo_370 0.534596639 0.553317603 0.864392023
0.16041831
pseudo_3700 0.794344384 -0.053255103
0.346062505 -0.449379333
pseudo_3701 0.373947507 0.944623324
0.950451713 -0.28458251
pseudo_3702 0.555365403 1.040915769
0.163306737 -0.309171574
pseudo_3703 0.804004946 -0.18748821 0.286182642
0.087993783
pseudo_3704 0.994766853 -0.052610234
0.838198714 -0.172574852
pseudo_3705 0.850278186 -0.292673914 0.457045676
0.319497972
pseudo_3706 0.184569604 -1.598396581
0.498227143 -0.668361652
pseudo_3707 0.754248325 0.614031429
0.659046655 -0.431540334
pseudo_3708 0.163779296 1.209616173
0.060399694 -0.121753141
pseudo_3709 0.162835211 -0.669746264 0.664146758
0.251677713
pseudo_371 0.832551168 0.597834583
0.39271076 -0.288330641
pseudo_3710 0.438348215 0.062346631
0.040996046 -0.793818329
pseudo_3711 0.703750429 -3.000306676
0.369761761 -0.178195331
pseudo_3712 0.160492963 -1.39506333 0.844156389
0.282753306
pseudo_3713 0.633797178 0.34912441
0.593172954 -0.310408398
pseudo_3714 0.682979045 0.661163041
0.987839296 -0.364106051
pseudo_3715 0.242748243 -0.617010356 0.804912184
0.26311299
pseudo_3716 0.804004946 0.67453388
0.65622008 -0.046223772
pseudo_3717 0.661041404 -1.978476984
0.061125242 -0.79145976
pseudo_3718 0.690453162 -0.879351545 0.684701116
0.274298545
pseudo_3719 0.69911476 0.123557158
0.958545942 -0.152615654
pseudo_372 0.011063514 -1.957191139
0.430079946 -0.142418746
pseudo_3720 0.436963858 0.807109952 0.22855091
0.598524919
pseudo_3721 0.838043993 -0.272655016

0.701431204 -0.669384172
pseudo_3722 0.244328001 -0.420554857
0.831941353 -0.635505112
pseudo_3723 0.715387542 0.511206307 0.156112973
0.24795655
pseudo_3724 0.808846577 0.658101066 0.789525805
0.262780095
pseudo_3725 0.919375024 0.079195085 0.437886481
0.060030555
pseudo_3726 0.73414425 1.255601609 0.595879316
0.115965865
pseudo_3727 0.266531268 -3.12094593
0.955432225 -0.352941214
pseudo_3728 0.026510207 1.37709564
0.312372672 -0.176614866
pseudo_3729 0.944228817 -0.356895267 0.775118984
0.12326168
pseudo_373 0.126782652 1.326704938 0.03714064
0.939820916
pseudo_3730 0.084587825 0.211225778
0.925582048 -0.046629099
pseudo_3731 0.100305214 -0.286155194 0.316886561
0.296298943
pseudo_3732 0.87362036 0.373747397 0.146971171
0.465983021
pseudo_3733 0.315377203 1.720748257
0.516751123 -0.878856998
pseudo_3734 0.929725917 0.14258291
0.854819213 -0.373100055
pseudo_3735 0.524615894 0.763802497
0.903260667 -0.376536086
pseudo_3736 0.214955053 -0.57792453
0.708978739 -0.112673904
pseudo_3737 0.580261507 -0.665945827
0.707525041 -0.005553204
pseudo_3738 0.212938317 -0.980875672 0.677250751
0.346958897
pseudo_3739 0.73296716 -0.515303508 0.440197939
0.660453419
pseudo_374 0.417848889 -0.230447715 0.337332068
0.318349659
pseudo_3740 0.131115618 -0.515811008
0.237741326 -0.099043511
pseudo_3741 0.913172908 0.401952681 0.80037864
0.317818844
pseudo_3742 0.327970888 -0.071740983 0.096172772
0.257003445
pseudo_3743 0.413817852 0.607903779
0.006882359 -0.925635615
pseudo_3744 0.058499789 -3.264399664

0.908215001 -0.277840853
pseudo_3745 0.931172258 -1.58681276
0.656785003 -0.066958218
pseudo_3746 0.069666946 -2.567068583 0.900955345
0.282559412
pseudo_3747 0.294742665 0.439849876 0.425980006
0.270807731
pseudo_3748 0.315754099 0.294416957 0.511206965
0.163981777
pseudo_3749 0.325472724 -1.744248762
0.684498746 -0.260269022
pseudo_375 0.21265133 -1.405216749 0.852116503
0.160636252
pseudo_3750 0.690453162 -0.918250537
0.346062505 -0.511938061
pseudo_3751 0.997193581 -0.5296045 0.435121969
0.411537513
pseudo_3752 0.470876848 -1.689971809 0.270249472
0.175548844
pseudo_3753 0.877316587 0.347666003 0.479576564
0.641612465
pseudo_3754 0.322588794 -0.687103315 0.604031974
0.484446381
pseudo_3755 0.783213645 0.607684043
0.273317977 -0.63218264
pseudo_3756 0.287243366 -0.346359608
0.985345049 -0.485853838
pseudo_3757 0.971629905 -1.237845669 0.263849325
0.306525529
pseudo_3758 0.629895097 -0.052976283 0.417848887
0.481512121
pseudo_3759 0.984721509 0.33558028 0.622694113
0.317424846
pseudo_376 0.264183548 -0.149039161 0.764960467
0.126339441
pseudo_3760 0.760193826 -0.394584029 0.706653335
0.137013279
pseudo_3761 0.040534553 1.486140308 0.527932174
0.279447789
pseudo_3762 0.539235237 0.495276772 0.931793607
0.081574588
pseudo_3763 0.767347144 0.050748443 0.847216135
0.314465839
pseudo_3764 0.765556929 0.669404089 0.696223138
0.163076088
pseudo_3765 0.862548533 -0.566027569 0.225544405
0.520069435
pseudo_3766 0.513723181 0.419052545
0.763171922 -0.513533568
pseudo_3767 0.929929686 -0.287713985 0.298722772

0.573075554
pseudo_3768 0.999688175 0.454859446 0.345662571
0.161200035
pseudo_3769 0.847828362 -0.015705084
0.02831738 -0.797307597
pseudo_377 0.234961078 1.479952227 0.667625183
0.293074918
pseudo_3770 0.003889984 -1.984132843 0.967267561
0.094730186
pseudo_3771 0.624906002 -0.781074049
0.048559202 -0.547767723
pseudo_3772 0.632623491 -0.467383447 0.832890488
0.360210032
pseudo_3773 0.872388893 0.16591203 0.387967243
0.24754493
pseudo_3774 0.39923556 0.524964626
0.072527367 -0.363057631
pseudo_3775 0.969756792 -1.991986498
0.131691088 -0.32618982
pseudo_3776 0.187169258 -1.810424209
0.954809562 -0.427280023
pseudo_3777 0.692759018 -0.598690207 0.945473136
0.244513291
pseudo_3778 0.202003 1.605791159 0.990957268 0.231404159
pseudo_3779 0.239922806 -0.102286874
0.667555508 -0.163001973
pseudo_378 0.333410903 0.379961503
0.15840679 -0.755749103
pseudo_3780 0.701431205 -3.811875227
0.173942513 -0.605917723
pseudo_3781 0.631561902 0.698696719
0.473284662 -0.261006445
pseudo_3782 0.796153387 0.00462064
0.370179023 -0.166528662
pseudo_3783 0.097271715 2.042256835 0.88101549
0.16538024
pseudo_3784 0.180463495 2.008805215
0.144609512 -0.054149819
pseudo_3785 0.782913418 0.144438117 0.106259359
0.663380805
pseudo_3786 0.657915439 0.494256605 0.940496688
0.1818245
pseudo_3787 0.378162438 0.192878211
0.881632229 -0.342777303
pseudo_3788 0.485913831 -2.003352348
0.198656206 -0.522614591
pseudo_3789 0.295824608 -0.281484099 0.379856569
0.64974003
pseudo_379 0.344863589 -3.283122618 0.897073065
0.282373195

pseudo_3790 0.414711644 1.054331413
0.010089899 -0.656784684
pseudo_3791 0.109512949 -1.441800689 0.689877145
0.273787171
pseudo_3792 0.054750094 0.51565362
0.025361544 -1.162833507
pseudo_3793 0.7775149 -3.001321444
0.923719448 -0.202104637
pseudo_3794 0.329519269 -0.589347108 0.58401372
0.199822605
pseudo_3795 0.505194418 0.829430662
0.435121969 -0.099754343
pseudo_3796 0.172216689 0.503221372
0.195399909 -0.23567004
pseudo_3797 0.903260667 -1.879572295
0.594254823 -0.314244274
pseudo_3798 0.713346175 0.396907507 0.062790973
0.882250006
pseudo_3799 0.062131171 1.911576292
0.472802558 -0.398757492
pseudo_38 0.961215791 0.229984792
0.865178638 -0.17047611
pseudo_380 0.671828263 0.38065571
0.152483015 -0.377623496
pseudo_3800 0.713054719 0.62903439
0.511206965 -0.34655548
pseudo_3801 0.885314538 -1.803016126 0.369421941
0.117559914
pseudo_3802 0.501704379 0.936559637
0.099339224 -0.414531402
pseudo_3803 0.121806507 -1.524963535 0.871773274
0.122234112
pseudo_3804 0.43236756 0.928514919 0.169287982
0.705849197
pseudo_3805 0.480548586 -0.928516668 0.407593301
0.64393655
pseudo_3806 0.20305941 -1.218580461
0.170260038 -0.475767275
pseudo_3807 0.842933147 -0.819402432
0.263849325 -0.34114163
pseudo_3808 0.507695111 0.520523022
0.859477714 -0.053599254
pseudo_3809 0.211792048 1.096775763 0.160492961
0.800809294
pseudo_381 0.623799634 0.950924195
0.898310097 -0.105162722
pseudo_3810 0.564309934 -0.462756854 0.883482874
0.317706267
pseudo_3811 0.546490688 0.454594683
0.444841789 -0.101467977

pseudo_3812	0.711306859	-3.276971707	0.502202171	0.914151314
pseudo_3813	0.506193916	-0.578428871	0.317643015	-0.271339
pseudo_3814	0.811270152	1.021315758	0.52946636	0.386630412
pseudo_3815	0.215244278	0.375292462	0.966644446	0.266860891
pseudo_3816	0.105584259	1.114080309	0.850890868	-0.682041719
pseudo_3817	0.943606708	0.411075034	0.412479303	0.313596662
pseudo_3818	0.684701118	-0.982914574	0.804004946	-0.266590928
pseudo_3819	0.852729455	0.197717918	0.641601866	0.116783487
pseudo_382	0.691605732	-0.64186548	0.585087853	-0.427488313
pseudo_3820	0.872388893	0.238432697	0.615529042	0.658362894
pseudo_3821	0.960414478	-0.726703143	0.319539324	-0.298482829
pseudo_3822	0.946717589	-0.06737367	0.058499788	0.919095987
pseudo_3823	0.449982149	-0.571837283	0.593713776	0.328903484
pseudo_3824	0.873004588	-0.801349061	0.634344073	0.019993491
pseudo_3825	0.175183135	0.132400164	0.858249979	0.173856477
pseudo_3826	0.052808112	0.101946451	0.915653141	0.058842927
pseudo_3827	0.923719448	-0.019972853	0.455628007	-0.082963026
pseudo_3828	0.273317979	-2.10274455	0.372688715	0.095252555
pseudo_3829	0.55956596	-0.442864879	0.063123041	-0.521583019
pseudo_383	0.353717205	-0.645953456	0.474732596	0.223435071
pseudo_3830	0.1086492	-5.723973595	0.377317117	0.210606546
pseudo_3831	0.100305213	-1.106494064	0.773921826	-1.442908299
pseudo_3832	0.736500302	-1.127794804	0.552747897	-0.230892491
pseudo_3833	0.042691639	-1.239153971	0.756010148	0.010462033
pseudo_3834	0.637131424	0.134434694	0.883482874	0.148959244

pseudo_3835 0.939874791 -2.887977876 0.341282752
0.34840235
pseudo_3836 0.474249683 -0.940786416 0.00762834
0.573934254
pseudo_3837 0.149202357 -0.970103076
0.966142811 -0.077796577
pseudo_3838 0.922523167 -2.333727866 0.513905536
0.052619249
pseudo_3839 0.82950311 -0.692147677
0.135153522 -1.002677107
pseudo_384 0.476909052 0.393887422
0.538718841 -0.256353252
pseudo_3840 0.962283232 1.150036069
0.524870612 -0.332853938
pseudo_3841 0.294742665 -0.024494205
0.576520626 -0.114104951
pseudo_3842 0.577588289 -0.943571479 0.30017894
0.116833937
pseudo_3843 0.988462877 0.25351057 0.810058136
0.18725978
pseudo_3844 0.189795853 0.077655927
0.16904562 -0.182436829
pseudo_3845 0.848440681 -0.243295192 0.819766771
0.106029641
pseudo_3846 0.227645979 1.018560793
0.936765865 -0.400635583
pseudo_3847 0.077249467 -1.083435336 0.986592156
0.680777925
pseudo_3848 0.804609743 -0.751332724
0.29799646 -0.502737427
pseudo_3849 0.355749252 2.066993098 0.639923916
0.284370586
pseudo_385 0.137414965 -0.13448075
0.786518264 -0.046692733
pseudo_3850 0.888421011 0.963570521 0.430994146
0.518304331
pseudo_3851 0.586969792 -0.997333292 0.279178832
0.10634586
pseudo_3852 0.346160068 -1.747152387 0.110565544
0.669637639
pseudo_3853 0.754248325 0.502830977 0.993451748
0.256344456
pseudo_3854 0.288306738 0.350158111
0.474249682 -0.20828868
pseudo_3855 0.932414998 -0.453479005
0.024059787 -0.769848577
pseudo_3856 0.617729822 -0.179549158 0.450451116
0.285622488
pseudo_3857 0.980273453 0.038177522
0.684801526 -0.383474993

pseudo_3858	0.968513851	-0.356666466	0.434662199
0.298423719			
pseudo_3859	0.700272634	0.88544573	
0.186126168	-0.940669998		
pseudo_386	0.427365939	-1.25143249	
0.284702573	-0.327375149		
pseudo_3860	0.145020354	2.138477174	
0.290084914	-0.31198304		
pseudo_3861	0.331461393	-1.482816171	
0.488212645	-0.469203047		
pseudo_3862	0.919375024	-0.146150113	0.083466346
0.667780559			
pseudo_3863	0.237120631	-2.711641851	
0.944228817	-0.380098664		
pseudo_3864	0.449045042	-0.745735301	0.951074175
0.051292673			
pseudo_3865	0.197566397	-0.507642233	0.034455672
0.800906622			
pseudo_3866	0.447174154	0.272653982	0.049367143
0.596043407			
pseudo_3867	0.400548304	0.679491139	0.822198314
0.242618238			
pseudo_3868	0.94485096	-0.421947842	0.564309933
0.201906626			
pseudo_3869	0.828893804	-0.261882364	0.062240737
0.605758696			
pseudo_387	0.807635475	-0.679623539	0.042967141
0.215026681			
pseudo_3870	0.395747586	1.026372008	0.186647174
0.839916532			
pseudo_3871	0.577054342	0.172349507	
0.844768152	-0.255225822		
pseudo_3872	0.097271715	-1.480342851	
0.17197119	-0.493545398		
pseudo_3873	0.227344903	-1.99242125	0.415606573
0.764600223			
pseudo_3874	0.260854461	-0.297061463	0.929929686
0.181726775			
pseudo_3875	0.319539326	-0.14860291	
0.833161083	-0.172184223		
pseudo_3876	0.031196175	1.575378265	0.779313226
0.188705834			
pseudo_3877	0.400548303	0.74912172	0.471357867
0.171101387			
pseudo_3878	0.151824275	-0.375016711	0.896454643
0.313056985			
pseudo_3879	0.993451748	-1.477764743	0.551702593
0.861497307			
pseudo_388	0.007557646	-1.430091318	0.383685393
0.285050774			

pseudo_3880 0.788802574 0.877847665
0.613971873 -0.154518135
pseudo_3881 0.860705789 0.874408089
0.728852304 -0.547375283
pseudo_3882 0.06995411 0.777201314
0.434202709 -0.343196334
pseudo_3883 0.986592156 0.463170897 0.120306501
1.163792477
pseudo_3884 0.670401386 -0.178519366 0.600221163
0.119271425
pseudo_3885 0.593713777 0.29278053 0.90759551
0.124123175
pseudo_3886 0.83031882 0.53563579
0.790071741 -0.040119147
pseudo_3887 0.430994149 -2.893877293
0.975369738 -0.299018344
pseudo_3888 0.840487801 -0.299648204 0.436502964
0.246357309
pseudo_3889 0.261517946 -1.15992427
0.588854528 -1.347350101
pseudo_389 0.123702027 -1.646879879 0.966644446
0.425754728
pseudo_3890 0.329907103 0.619455205
0.569073183 -0.593293277
pseudo_3891 0.593172956 0.510782573
0.40011043 -0.085534742
pseudo_3892 0.702590471 0.517088774
0.48006244 -0.413745547
pseudo_3893 0.402741967 -0.599445039 0.401863637
0.237187111
pseudo_3894 0.300543722 -1.195707842 0.119191021
0.244363333
pseudo_3895 0.310131694 0.758859843
0.335760058 -0.15599002
pseudo_3896 0.596421265 -0.460414494 0.546490686
0.002213532
pseudo_3897 0.74950237 -1.121819036 0.624906001
0.250814662
pseudo_3898 0.667555509 1.581409214
0.307530665 -0.439735258
pseudo_3899 0.484935928 -0.678403358 0.041306175
0.926604548
pseudo_39 0.689301309 0.165151758
0.947962171 -0.278866568
pseudo_390 0.387537755 -1.661401729 0.167356368
0.537015373
pseudo_3900 0.928066149 -1.05069049
0.509700332 -1.17331157
pseudo_3901 0.669262465 -0.694536529
0.245596991 -0.164677406

pseudo_3902 0.628230174 0.887769314 0.623246767
0.082074965
pseudo_3903 0.581365279 -1.021779741
0.485114882 -0.382195259
pseudo_3904 0.583477 0.491598415 0.793138994 0.320114073
pseudo_3905 0.111432299 1.18566538
0.492788924 -0.185677227
pseudo_3906 0.711889311 -0.364860657
0.718891759 -0.138579699
pseudo_3907 0.479576566 0.229067145
0.883482874 -0.152337817
pseudo_3908 0.232501884 -1.241702483 0.461313514
0.358934107
pseudo_3909 0.7775149 0.657615801
0.886568691 -0.076001939
pseudo_391 0.375629979 1.364589949 0.309015192
0.267919261
pseudo_3910 0.929929686 0.578614693
0.143730867 -0.699941963
pseudo_3911 0.305681655 0.688929941 0.942984634
0.328241656
pseudo_3912 0.195399911 1.600359365
0.495256891 -0.003835556
pseudo_3913 0.921236633 0.715554131
0.708978739 -0.369322662
pseudo_3914 0.367264354 0.521999273 0.592632358
0.016339803
pseudo_3915 0.275032959 -1.388930539 0.433513999
0.057445439
pseudo_3916 0.11863633 -0.743804362 0.57119633
0.161317775
pseudo_3917 0.720646093 0.459113664 0.522834633
0.05808236
pseudo_3918 0.168078776 -0.480649194
0.388397024 -0.266057672
pseudo_3919 0.308272339 0.509470265 0.296547377
0.637086871
pseudo_392 0.464234521 0.565519328 0.097924718
0.266666287
pseudo_3920 0.5192341 0.01023325 0.09462632
0.241024015
pseudo_3921 0.219322905 -0.513977028 0.390550273
0.358576657
pseudo_3922 0.014884947 -0.976379293 0.807030096
0.077123254
pseudo_3923 0.118636329 2.077891262
0.434662198 -0.166522522
pseudo_3924 0.361478056 -1.538038063
0.303472593 -0.626756845
pseudo_3925 0.836822673 -3.44833327 0.988462877

0.365963595
pseudo_3926 0.195399911 1.529909911
0.227344901 -0.083865024
pseudo_3927 0.460363188 1.11592759
0.971629905 -0.263322617
pseudo_3928 0.02656347 1.467195743 0.074539529
0.541037503
pseudo_3929 0.496245924 0.283728261
0.100791007 -0.819314159
pseudo_393 0.738858835 -0.303384593
0.058499788 -0.44598682
pseudo_3930 0.692759018 -0.238151605 0.662728488
0.242052981
pseudo_3931 0.158868602 -3.202515972 0.040996046
0.411265311
pseudo_3932 0.330295232 0.954987523 0.570665188
0.270903832
pseudo_3933 0.568542988 -0.725829991 0.015507052
0.723514052
pseudo_3934 0.468774706 0.38798819 0.771094504
0.506493915
pseudo_3935 0.711332579 -2.040163202
0.717482837 -0.206615064
pseudo_3936 0.838654799 -0.185051047
0.685275505 -0.188482603
pseudo_3937 0.366434224 0.996021403 0.447641458
0.298051085
pseudo_3938 0.443910792 -0.668433752
0.838043992 -0.087209503
pseudo_3939 0.584819234 -0.556394397 0.695067722
0.063520964
pseudo_394 0.421000071 -0.810288357 0.589932771
0.369085808
pseudo_3940 0.433284573 -0.450902813
0.283717927 -0.686928689
pseudo_3941 0.78411452 0.423199749 0.335367794
0.766748769
pseudo_3942 0.551180307 0.093207623 0.465125767
0.278705155
pseudo_3943 0.903260667 0.245166554 0.422806989
0.451004832
pseudo_3944 0.873004588 -0.91795379
0.331850702 -0.385200401
pseudo_3945 0.305681656 -2.464312292
0.3849669 -0.654533642
pseudo_3946 0.831941353 -2.41501127
0.651707795 -0.513283542
pseudo_3947 0.456572846 0.256576584 0.354529139
0.058366859
pseudo_3948 0.313872573 -1.014344766

0.263849325	-0.694696009
pseudo_3949	0.057363364	1.381442324
0.543893911	-0.301129119
pseudo_395	0.613331718	0.577345797	0.037784943
0.801166111
pseudo_3950	0.446707126	-0.081893157	0.684701116
0.744632413
pseudo_3951	0.210282863	1.863685161	0.117159288
0.05124619
pseudo_3952	0.660744944	1.18655384	0.015741952
0.443778618
pseudo_3953	0.111256723	-0.793595247	0.223159613
0.252840935
pseudo_3954	0.043859474	2.1277467	0.485424744
0.059528053
pseudo_3955	0.178439631	0.393474549	0.422354834
0.173032257
pseudo_3956	0.875468134	-0.159107494	0.83621216
0.313788072
pseudo_3957	0.289372761	1.65363219	0.484935926
0.192520168
pseudo_3958	0.539235237	0.98520493
0.741810456	-0.297474588
pseudo_3959	0.376051326	-1.142314723	0.028430238
0.570907573
pseudo_396	0.291155359	0.396954791
0.563781869	-0.249724542
pseudo_3960	0.363299709	-0.762136031	0.70214242
0.472665924
pseudo_3961	0.464234521	0.551555709	0.931036144
0.184054257
pseudo_3962	0.1091668	1.049189163	0.12199503
0.222467213
pseudo_3963	0.380280832	0.50134489
0.093239957	-0.748797696
pseudo_3964	0.497236009	0.617989352
0.561145131	-0.253188305
pseudo_3965	0.406708661	-0.581722329	0.456100288
0.953222071
pseudo_3966	0.741219827	0.451595867
0.540785903	-0.175719736
pseudo_3967	0.88965623	-2.60776205	0.120493208
0.259069695
pseudo_3968	0.137829254	-1.322837954
0.478605619	-0.392863988
pseudo_3969	0.147844618	-1.023893504	0.207251569
0.08602822
pseudo_397	0.064127964	1.889009258
0.224946508	-0.281549278
pseudo_3970	0.170991822	1.086908003	0.247828836

0.400848887
pseudo_3971 0.361889458 -1.373068257
0.249431677 -0.26110176
pseudo_3972 0.045691136 1.208999644
0.657350122 -0.324114223
pseudo_3973 0.406266769 -0.754931411
0.832551167 -0.155463919
pseudo_3974 0.701431205 0.936122086 0.936765865
0.021136153
pseudo_3975 0.074032262 1.87576887
0.718891759 -0.318982278
pseudo_3976 0.869311584 0.537821823 0.177935719
0.262256799
pseudo_3977 0.566424564 -1.241664092
0.421000069 -0.188652645
pseudo_3978 0.798567094 -0.48937639
0.29799646 -0.135321159
pseudo_3979 0.796153387 1.310489533 0.44904504
0.404503782
pseudo_398 0.511709694 -0.525334783 0.344863587
0.786887812
pseudo_3980 0.803400267 -0.410230044 0.668693288
0.19064927
pseudo_3981 0.849665593 -0.642884402
0.244961918 -0.099230043
pseudo_3982 0.034455673 2.307465726
0.72550558 -0.010153541
pseudo_3983 0.69969361 1.353198165
0.807030096 -0.402059014
pseudo_3984 0.707234431 -0.395317328
0.313497153 -0.228231363
pseudo_3985 0.87978223 1.248492852
0.85763624 -0.267460775
pseudo_3986 0.402373449 0.649407595 0.553447739
0.496101404
pseudo_3987 0.114092262 -1.554603095 0.767347143
0.078371424
pseudo_3988 0.265187962 0.711496335
0.521818166 -0.46664363
pseudo_3989 0.833771099 -0.602074237 0.072031336
0.960068251
pseudo_399 0.159099888 -1.399484524 0.240862029
0.570382231
pseudo_3990 0.676678942 -0.49657478
0.733555626 -0.142093864
pseudo_3991 0.541921457 0.446972035 0.131480105
0.486915639
pseudo_3992 0.35860646 1.371916817
0.346462734 -0.447730086
pseudo_3993 0.715387542 0.258351716 0.29474266

0.207068937
pseudo_3994 0.847828362 0.319400371 0.448109042
0.221838183
pseudo_3995 0.425525871 -1.424744069
0.516751123 -0.420966005
pseudo_3996 0.961660291 0.449928007 0.682979044
0.001022691
pseudo_3997 0.966644446 -0.645097772 0.582404245
0.140461671
pseudo_3998 0.218151962 1.913314801
0.87978223 -0.230312039
pseudo_3999 0.608400443 0.715694591 0.760789162
0.154530772
pseudo_4 0.401424907 -1.331806373 0.674964628 0.337269536
pseudo_40 0.449982149 1.703798541
0.054161341 -0.797154713
pseudo_400 0.674393566 -1.067771159 0.937387574
0.214574809
pseudo_401 0.05954907 1.526151842
0.511206965 -0.021661373
pseudo_402 0.779313227 -0.706693518
0.511709693 -0.324239718
pseudo_403 0.484447376 -0.679908083
0.868081211 -0.073372357
pseudo_404 0.811876329 0.561267584 0.956677633
0.335750078
pseudo_405 0.363537997 0.356078652 0.119561944
0.495980074
pseudo_406 0.759598632 -0.519522071
0.115170141 -0.414695907
pseudo_407 0.781712834 -0.884833731 0.386251021
0.434929887
pseudo_408 0.322206576 0.881114697
0.132920945 -0.18347746
pseudo_409 0.267204675 1.575643469
0.408036053 -0.237162692
pseudo_41 0.971071124 0.091322432 0.04328547
0.98577467
pseudo_410 0.462741061 1.767043134 0.522326273
0.144927551
pseudo_411 0.780512769 0.930910609
0.124275149 -0.423787194
pseudo_412 0.262182597 1.099164263
0.346863257 -0.174643134
pseudo_413 0.264852865 0.914765238
0.274689375 -0.38914975
pseudo_414 0.578122467 -0.081618304 0.85579554
0.803968625
pseudo_415 0.442393997 -0.046928412 0.24686211
0.116507511

pseudo_416	0.489836089	-0.634639114	
0.160492961	-0.188145918		
pseudo_417	0.137414965	0.81680321	
0.796153386	-0.128576514		
pseudo_418	0.608400443	0.476202911	0.399235558
0.064944921			
pseudo_419	0.00035262	2.030605018	0.300543718
0.720741176			
pseudo_42	0.186908083	0.48045818	
0.225245315	-0.550095329		
pseudo_420	0.805982299	-0.002555461	
0.339034308	-0.708676146		
pseudo_421	0.504695059	-0.230435043	0.410254103
0.481400549			
pseudo_422	0.070303695	2.658499957	
0.790017194	-0.112399011		
pseudo_423	0.081944011	-2.879554243	0.093545254
0.213090916			
pseudo_424	0.458465821	-1.363567089	0.266867823
0.10666204			
pseudo_425	0.219616344	1.564094469	
0.741219826	-0.556103138		
pseudo_426	0.94485096	-0.336601281	
0.036296138	-0.749998969		
pseudo_427	0.590472238	-0.588865087	0.559040047
0.538872414			
pseudo_428	0.655090826	-0.594584135	0.052236866
0.574787683			
pseudo_429	0.184052895	-1.073341205	0.412033692
0.30956401			
pseudo_43	0.048827274	1.834961806	
0.07936762	-0.828513682		
pseudo_430	0.885951389	0.607007894	
0.708397133	-0.240908212		
pseudo_431	0.219029747	0.634416832	
0.734144249	-0.475668621		
pseudo_432	0.111432299	-0.717673533	0.245914961
0.438497554			
pseudo_433	0.652271139	-0.397236315	0.250074838
0.076374232			
pseudo_434	0.118821002	-0.334939346	0.17970405
0.722554954			
pseudo_435	0.803400267	-0.701379832	0.35048122
0.827581248			
pseudo_436	0.993451748	-0.213292453	0.836822672
0.263746233			
pseudo_437	0.923098674	-0.24629155	0.295463663
0.239716932			
pseudo_438	0.160726036	1.49231047	
0.126782651	-0.532729252		

pseudo_439 0.14566841 -1.162700345
0.414264603 -0.713051525
pseudo_44 0.07277643 -0.840188218
0.698536083 -0.443905009
pseudo_440 0.501206846 0.699932473 0.382832508
0.000315054
pseudo_441 0.642161592 0.113135553 0.730614865
0.148838307
pseudo_442 0.440661071 -0.303976709 0.600221163
0.511557273
pseudo_443 0.355342254 -1.37537124 0.504695057
0.803887
pseudo_444 0.021629159 2.627969293
0.028600251 -0.685768014
pseudo_445 0.990333661 0.234316861
0.165441359 -0.309468061
pseudo_446 0.947339864 -0.704954667
0.654526492 -0.067625797
pseudo_447 0.391413602 0.473720708
0.474249682 -0.542210473
pseudo_448 0.143730869 -0.288101427
0.905737375 -0.363943156
pseudo_449 0.506193916 -2.943057883
0.269909991 -0.673521004
pseudo_45 0.248469108 2.047127844
0.655090824 -0.330916352
pseudo_450 0.306420374 0.46702814 0.969137025
0.279790102
pseudo_451 0.376941754 -1.191096881
0.337786661 -0.179260731
pseudo_452 0.172585426 0.925587411
0.873004587 -0.490221391
pseudo_453 0.025157391 -0.85364008
0.361478054 -0.758835467
pseudo_454 0.233340842 -1.306869232
0.940868561 -0.094861261
pseudo_455 0.263026168 -0.698391778
0.408888589 -0.626201823
pseudo_456 0.851503641 -0.299127037
0.959168762 -0.103968543
pseudo_457 0.171725957 -0.612807484
0.953564319 -0.168629711
pseudo_458 0.753654569 1.0388485
0.685850076 -0.207187541
pseudo_459 0.473767039 -0.941979845 0.597505831
0.250275703
pseudo_46 0.296547379 0.780061039
0.278484917 -0.354177524
pseudo_460 0.409587938 0.304193111
0.294022842 -0.616062294

pseudo_461 0.839265703 0.514379767 0.368928123
0.305715458
pseudo_462 0.411588368 0.619889528
0.971629905 -0.149202662
pseudo_463 0.704911077 -0.18784425
0.670401385 -0.247439005
pseudo_464 0.195939892 0.619677114
0.820982328 -0.545290166
pseudo_465 0.587238869 0.825192398 0.307530665
0.03594784
pseudo_466 0.407487228 0.171814909
0.560642751 -0.375483287
pseudo_467 0.126977156 0.278075728
0.073905885 -0.798028196
pseudo_468 0.566953814 1.411974086 0.178593355
0.442302585
pseudo_469 0.920616048 0.944957688 0.819409811
0.244733453
pseudo_47 0.971629905 0.488820637
0.794645807 -0.144681025
pseudo_470 0.924340269 -1.503139381
0.905118111 -0.195202865
pseudo_471 0.421902964 -3.300399345
0.225843778 -1.252336255
pseudo_472 0.892745443 -0.40489229
0.918134196 -0.261618572
pseudo_473 0.622694113 -0.843759574 0.605668498
0.622251852
pseudo_474 0.493281992 -1.497278622
0.239922804 -0.746698651
pseudo_475 0.154070166 -0.869179505
0.4129252 -0.104645767
pseudo_476 0.885334156 0.805615883 0.924340269
0.224031765
pseudo_477 0.278484919 -0.429593542
0.741810456 -0.255025077
pseudo_478 0.390981794 -0.632833937 0.347264075
0.263846332
pseudo_479 0.65848095 -0.478555243
0.304207765 -0.521725582
pseudo_48 0.727091176 0.337547906
0.125813606 -0.478525245
pseudo_480 0.529978261 -1.109541675
0.388827094 -0.370111272
pseudo_481 0.297633749 -2.421634627
0.563254045 -0.598092311
pseudo_482 0.385822692 -0.289645145
0.674393564 -0.053379342
pseudo_483 0.805214657 -0.759791797
0.572525205 -0.429604766

pseudo_484	0.752467494	0.613832556	0.748909787	0.218125218
pseudo_485	0.042407322	-4.323793666	0.386679641	0.260351608
pseudo_486	0.649456414	0.489303638	0.894599754	-0.211217916
pseudo_487	0.632117921	0.61534041	0.085294916	0.269979425
pseudo_488	0.307530667	0.255819436	0.076525539	0.660790148
pseudo_489	0.311624497	-0.470493126	0.399235557	0.71539233
pseudo_49	0.190854061	1.402583313	0.784715263	0.120694824
pseudo_490	0.545451241	0.855274524	0.190059997	-0.962935246
pseudo_491	0.105584259	0.470321603	0.607853614	0.184289967
pseudo_492	0.053868954	-2.502954087	0.512212678	0.042363435
pseudo_493	0.407150839	-0.631657763	0.947651013	0.416255062
pseudo_494	0.535111043	0.640987574	0.409809921	0.654719674
pseudo_495	0.21785993	-0.418354592	0.029756205	0.448552447
pseudo_496	0.325657183	0.179992634	0.107278422	-0.56577839
pseudo_497	0.394878477	-0.479843157	0.844768151	-0.078704881
pseudo_498	0.830112518	-1.094755093	0.384966898	0.148468483
pseudo_499	0.115531211	-1.156198463	0.303472593	-0.58626305
pseudo_5	0.393576982	1.597290635	0.915033004	-0.014714037
pseudo_50	0.174686097	-1.792227824	0.968513851	-0.186522516
pseudo_500	0.703170363	-1.897009644	0.38155536	0.167080487
pseudo_501	0.823897653	0.29558691	0.853955624	0.246000644
pseudo_502	0.130916206	-2.2178472	0.802795704	0.337445048
pseudo_503	0.791934102	0.364074512	0.588315746	0.576573785
pseudo_504	0.442052135	0.11971407	0.153393761	0.456683359
pseudo_505	0.513219423	-0.810659093	0.351692507	0.270212918

pseudo_506 0.984721509 1.88572707
0.919995511 -0.520860402
pseudo_507 0.238363174 1.547546761 0.139496039
0.825543335
pseudo_508 0.568013027 -0.571512083
0.985968598 -0.169839022
pseudo_509 0.058812949 -3.879181614 0.697379259
0.495144771
pseudo_51 0.582495547 0.719178603 0.776142239
0.484770242
pseudo_510 0.29727133 0.790512866 0.172954752
0.767919889
pseudo_511 0.238986166 0.59834475
0.046889972 -0.824485308
pseudo_512 0.600221164 -0.47830923
0.180720371 -0.398625432
pseudo_513 0.509700334 -2.781301026 0.92682401
0.341261029
pseudo_514 0.56272646 1.009721333
0.180211678 -0.590106631
pseudo_515 0.393143726 -0.481020726
0.624906 -0.193392023
pseudo_516 0.277100613 -1.319615749
0.219616342 -0.429046483
pseudo_517 0.13171527 1.208399288
0.58401372 -0.045835884
pseudo_518 0.71686698 0.275319096
0.68328813 -0.24629661
pseudo_519 0.985968598 0.662426452
0.70607241 -0.271058317
pseudo_52 0.107449018 0.388684428
0.643281649 -0.712559894
pseudo_520 0.297508058 -0.9215693 0.137269236
0.759764998
pseudo_521 0.133324732 0.652354071 0.963529184
0.297321656
pseudo_522 0.480062442 -2.888530447 0.994699014
0.485990785
pseudo_523 0.885334156 -0.187460648 0.946095346
0.208918961
pseudo_524 0.840487801 -0.383357378
0.704330665 -0.283761396
pseudo_525 0.88965623 -0.159879422
0.939252931 -0.380431343
pseudo_526 0.238363174 0.897972884
0.574920881 -0.240186876
pseudo_527 0.240859829 1.812832487
0.85417256 -0.248051077
pseudo_528 0.103248167 0.39345259
0.165441359 -0.69648353

pseudo_529 0.322971308 -3.510423231
0.384539441 -0.208961904
pseudo_53 0.018857003 2.644112015
0.116576935 -0.308655108
pseudo_530 0.813695537 0.720017246 0.170016633
0.57486526
pseudo_531 0.059760789 2.705757448 0.836212159
0.261332478
pseudo_532 0.032251976 -1.997935965
0.340490255 -0.351643222
pseudo_533 0.002048308 -1.476488392 0.026670272
0.713996649
pseudo_534 0.507695111 -2.008727449
0.436042352 -0.019606321
pseudo_535 0.204171233 -0.621886244
0.519281454 -0.514167906
pseudo_536 0.039926086 -2.412948705 0.440197939
0.276855177
pseudo_537 0.142237604 -2.870186395
0.985345049 -0.728652167
pseudo_538 0.29079825 0.836773195 0.750687977
0.126340387
pseudo_539 0.345662573 0.389110374 0.991580881
0.048157229
pseudo_54 0.31069287 1.115745448
0.943492125 -0.29300601
pseudo_540 0.636015866 0.934495284
0.819159154 -0.243168687
pseudo_541 0.906976075 0.113573217
0.042011237 -0.659323834
pseudo_542 0.234341663 1.415869758 0.204171231
0.697875231
pseudo_543 0.226443378 -1.631264154
0.248789674 -0.037466958
pseudo_544 0.982850945 0.183743958
0.727091175 -0.063571662
pseudo_545 0.977863265 1.017217358
0.782913417 -0.013204447
pseudo_546 0.564838235 -0.233020652
0.659612556 -0.326125754
pseudo_547 0.698536085 -3.953032051 0.167356368
0.207576847
pseudo_548 0.223754115 -1.245243601
0.790127692 -0.320091273
pseudo_549 0.867466146 -0.434295739
0.167837712 -0.726559855
pseudo_55 0.689877146 0.591241529
0.022376172 -0.562347259
pseudo_550 0.715971164 -0.518884808
0.376051325 -0.609621946

pseudo_551	0.711889311	0.585381399	
0.215533782	-0.460134469		
pseudo_552	0.669262465	0.015598513	0.931172258
0.200998753			
pseudo_553	0.91441292	0.345866085	0.050554056
0.991849383			
pseudo_554	0.91441292	-0.144882634	0.774520338
0.256296649			
pseudo_555	0.023574762	1.769749161	0.556414095
0.129006199			
pseudo_556	0.709560516	-1.035787493	
0.593713776	-0.342722936		
pseudo_557	0.083327014	-1.175199307	
0.847216135	-0.278960382		
pseudo_558	0.796756632	0.057062653	
0.92682401	-0.27679704		
pseudo_559	0.491803588	-2.428097919	
0.307901353	-0.140560288		
pseudo_56	0.460838215	0.338915282	0.689589203
0.08787384			
pseudo_560	0.33576006	-1.761354694	
0.724745245	-0.252253735		
pseudo_561	0.799170823	-0.316840541	
0.430079947	-0.218801722		
pseudo_562	0.428710763	-0.623318439	
0.496245922	-0.56787725		
pseudo_563	0.858863804	-0.546443675	
0.94485096	-0.151764779		
pseudo_564	0.593713777	-1.017854324	
0.773921827	-0.03241347		
pseudo_565	0.449513457	1.271581857	
0.772725207	-0.013754325		
pseudo_566	0.481521677	-1.546543944	0.452329757
0.218807958			
pseudo_567	0.942984634	0.509589378	0.827675501
0.382695843			
pseudo_568	0.770333593	-0.673271377	0.284948481
0.333175738			
pseudo_569	0.144804826	0.032833103	
0.436963856	-0.053254488		
pseudo_57	0.177684163	1.515557923	
0.169287982	-0.602776626		
pseudo_570	0.606760618	-0.462927868	
0.811876329	-0.071012768		
pseudo_571	0.920616048	0.230812236	0.799774671
0.367474437			
pseudo_572	0.542856924	0.556253617	
0.7566248	-0.224285157		
pseudo_573	0.414711644	-0.68844033	
0.105921382	-0.015916662		

pseudo_574	0.350078049	-2.031649932	
0.350884688	-0.16283574		
pseudo_575	0.90945415	0.631389079	0.240548667
0.446205286			
pseudo_576	0.246525928	-1.263472584	
0.269048786	-0.442595577		
pseudo_577	0.724745246	-0.881609012	0.17123627
0.129450881			
pseudo_578	0.90945415	-0.93990327	0.717723025
0.188811274			
pseudo_579	0.030830689	1.275858277	
0.979110112	-0.283883102		
pseudo_58	0.902641636	0.096934468	
0.959791608	-0.276273166		
pseudo_580	0.274079156	0.44968423	
0.112979583	-0.590003268		
pseudo_581	0.923098674	0.906805615	
0.961037372	-0.132926769		
pseudo_582	0.255261835	-1.638983971	0.732084753
0.143838239			
pseudo_583	0.000880312	1.315999494	
0.790729703	-0.348832406		
pseudo_584	0.941118621	-0.251104002	0.320680657
0.853274198			
pseudo_585	0.913172908	-0.208151895	
0.725917889	-0.173015246		
pseudo_586	0.68026516	1.148159138	0.04260737
0.864363639			
pseudo_587	0.191650562	-1.039253468	0.628230172
0.03700057			
pseudo_588	0.033468079	1.73975679	0.860091709
0.173184557			
pseudo_589	0.11038155	-2.380822821	
0.834820415	-0.051220157		
pseudo_59	0.206408154	1.838164672	0.063123041
0.409202171			
pseudo_590	0.707815697	-0.111555139	0.065947094
0.996315854			
pseudo_591	0.878549261	-0.549236828	
0.397489264	-0.848695483		
pseudo_592	0.873004588	-0.045667465	
0.23681071	-0.135289716		
pseudo_593	0.822198315	0.457388393	
0.094620113	-0.431985497		
pseudo_594	0.392278086	0.030536913	
0.30237205	-0.23969674		
pseudo_595	0.143516812	-1.284083257	
0.763767964	-0.714584147		
pseudo_596	0.225544407	2.045493843	0.527932174
0.234424444			

pseudo_597	0.635458396	0.362256282	0.225544405
0.369012211
pseudo_598	0.457518784	0.863908022
0.507695109	-0.073077021
pseudo_599	0.277100613	-0.311218486	0.676678941
0.226757381
pseudo_6 0.804609743	-0.397284375	0.302005794	0.406632255
pseudo_60	0.145020354	1.572535831
0.091876002	-0.815688216
pseudo_600	0.311250854	-0.670101741
0.33029523	-1.093487313
pseudo_601	0.256898002	1.393202474
0.488363247	-0.414022869
pseudo_602	0.222220795	0.403128308
0.893129746	-0.074057359
pseudo_603	0.958545942	-0.149413344	0.38028083
0.302901416
pseudo_604	0.682405387	-0.330339484	0.042967141
0.657209607
pseudo_605	0.97225317	0.067515465	0.673822688
0.023742485
pseudo_606	0.0503878	-1.177676207	0.246190062
0.55166525
pseudo_607	0.516245826	1.491532563	0.600221163
0.251505681
pseudo_608	0.373527617	-1.155364065
0.460838212	-0.655313404
pseudo_609	0.296185846	0.797407165	0.354123024
0.333030025
pseudo_61	0.680869345	-0.969076893
0.206112732	-0.41789354
pseudo_610	0.533568581	0.954709245	0.822198314
0.255714934
pseudo_611	0.612782928	-2.533920617
0.211506176	-0.486517364
pseudo_612	0.348870294	-1.693221284
0.727678058	-0.311084473
pseudo_613	0.734733029	0.189700735
0.841098993	-0.228122116
pseudo_614	0.847828362	0.211834126
0.604577262	-0.085924557
pseudo_615	0.41964786	0.22071248	0.288306736
0.185343825
pseudo_616	0.981603955	0.70800337
0.046976607	-1.098297617
pseudo_617	0.326042061	0.824504741
0.104912564	-0.835778555
pseudo_618	0.169530607	-0.064589928
0.176680578	-0.668392731
pseudo_619	0.781112736	0.047890703	0.332240309

0.074978156
pseudo_62 0.145884921 −1.503787891
0.110731303 −0.276151606
pseudo_620 0.373947507 −1.136045223 0.666986904
0.081113548
pseudo_621 0.719476374 0.198432056 0.052808111
0.795673653
pseudo_622 0.502700229 0.652118402 0.931793607
0.193631441
pseudo_623 0.904498905 −0.071554731 0.860091709
0.238954198
pseudo_624 0.813648431 −0.675212405 0.587026842
0.110941095
pseudo_625 0.500709574 1.014930096 0.024256206
0.930697864
pseudo_626 0.90759551 −0.713803777 0.283016371
0.339982394
pseudo_627 0.990135973 0.322962305 0.286189444
0.729759015
pseudo_628 0.32604206 −0.742772496 0.249753112
0.244403319
pseudo_629 0.647952638 −0.971832292 0.752253293
0.306946641
pseudo_63 0.791934102 0.674836179
0.27641022 −0.715993183
pseudo_630 0.192182919 1.132630031 0.428710761
0.165540804
pseudo_631 0.43489205 0.382664339 0.942984634
0.102876449
pseudo_632 0.713637674 0.926961781
0.747132931 −0.434892026
pseudo_633 0.252983393 −1.058754065
0.827066505 −0.135528961
pseudo_634 0.68757488 0.276678498
0.641042345 −0.004702271
pseudo_635 0.252983393 1.886324224 0.213800951
0.054357437
pseudo_636 0.046544777 0.540281661
0.561145131 −0.330591033
pseudo_637 0.44764146 −0.381031714 0.406708659
0.326570291
pseudo_638 0.706653336 −0.941246499
0.911933106 −0.214267984
pseudo_639 0.496740835 0.07744788
0.891509559 −0.465589063
pseudo_64 0.850278186 0.102432435 0.087734847
0.264845948
pseudo_640 0.898928706 −0.482694016
0.929929686 −0.258871888
pseudo_641 0.848440681 0.351982066 0.087734847

0.593574891			
pseudo_642	0.901403752	0.431616804	0.142450193
0.679824717			
pseudo_643	0.615529044	-0.554255919	0.923719448
0.46648788			
pseudo_644	0.337725811	0.457779346	0.916893567
0.193189495			
pseudo_645	0.9007849	-0.016008237	0.54597084
0.230428984			
pseudo_646	0.845380009	0.464992888	
0.566953813	-0.026795739		
pseudo_647	0.254609401	1.017762523	0.08963841
0.590506261			
pseudo_648	0.64440252	-0.630114844	0.373947505
0.278075122			
pseudo_649	0.437886483	-1.280724105	0.607853614
0.316168548			
pseudo_65	0.704588941	0.265658324	
0.079588442	-0.338223112		
pseudo_650	0.794344384	0.799684002	
0.525890122	-0.49613855		
pseudo_651	0.983474456	-2.029618717	
0.269231905	-0.546324667		
pseudo_652	0.282315993	-2.968411403	
0.792536486	-0.225862229		
pseudo_653	0.337371437	-0.707553484	0.265848649
0.27327384			
pseudo_654	0.745357419	-0.527437571	0.783513904
0.03557453			
pseudo_655	0.763171923	0.875285155	
0.63211792	-0.275195547		
pseudo_656	0.197566397	-0.475658948	
0.793741628	-0.068204177		
pseudo_657	0.593172956	1.000189604	
0.263181758	-0.563872214		
pseudo_658	0.820374496	1.2330078	
0.972253169	-0.143602185		
pseudo_659	0.829033665	0.438503178	
0.636147316	-0.282850601		
pseudo_66	0.126977157	0.642052513	
0.434662199	-0.330993098		
pseudo_660	0.976616473	0.173376576	
0.644963257	-0.208448035		
pseudo_661	0.300961206	0.045963432	0.243514697
0.134755294			
pseudo_662	0.979110112	-0.271233534	0.333020408
0.301778748			
pseudo_663	0.171971192	-0.194481324	0.351692507
0.462946672			
pseudo_664	0.237430837	-1.779485503	

0.474249682	-0.096506793
pseudo_665	0.955432225	-0.366435266	0.679539852
0.322247305
pseudo_666	0.334584153	1.610597628	0.982850945
0.211112184
pseudo_667	0.164252882	-1.040888783	0.950451713
0.313707897
pseudo_668	0.733555627	-0.876049217
0.839265702	-0.193897992
pseudo_669	0.690453162	0.697240476
0.744174493	-0.101788324
pseudo_67	0.942984634	0.539190774	0.211792046
0.501137773
pseudo_670	0.617729822	-0.704126969	0.941118621
0.212025997
pseudo_671	0.896454643	-0.607866113	0.841098993
0.041166929
pseudo_672	0.249753114	-2.352896631	0.123511443
0.141356574
pseudo_673	0.736500302	-0.162818383
0.90945415	-0.846905737
pseudo_674	0.07963568	1.816825891	0.030952106
0.532904881
pseudo_675	0.814288093	-0.155590837	0.142130053
0.339895336
pseudo_676	0.64440252	0.181877494
0.893981586	-0.231908973
pseudo_677	0.884716992	-0.144988384
0.177935719	-0.622568972
pseudo_678	0.030589079	2.411655704
0.470396096	-0.237690556
pseudo_679	0.181485411	-2.708862664	0.729439665
0.176719974
pseudo_68	0.648331921	-0.342288148	0.133526979
0.282848361
pseudo_680	0.888421011	-0.749213998
0.451389883	-0.380616623
pseudo_681	0.593283894	-0.149007834	0.083482882
0.739113467
pseudo_682	0.590472238	0.252184434	0.44437615
0.293254274
pseudo_683	0.946717589	-0.619731102
0.209796818	-0.324086883
pseudo_684	0.770333593	0.184188365
0.173942513	-0.216555114
pseudo_685	0.176931079	1.713817957	0.274346085
0.6222125
pseudo_686	0.066408464	-1.243800438	0.080715281
0.335042875
pseudo_687	0.613880725	0.816414365

0.676107316	-0.030670989
pseudo_688	0.396617849	0.380156778
0.403621443	-0.221750528
pseudo_689	0.102423877	1.083321008	0.544153311
0.268313063
pseudo_69	0.169045622	1.937194271	0.408036053
0.207006039
pseudo_690	0.007663909	0.780388154
0.808846576	-0.254747978
pseudo_691	0.093392507	1.46250592
0.138660716	-0.47332413
pseudo_692	0.695645343	-0.711020874
0.722401897	-0.566087653
pseudo_693	0.852729455	-0.398863053	0.285829657
0.242178931
pseudo_694	0.960414478	-0.181125494
0.580796843	-0.27462097
pseudo_695	0.88224904	-0.707908893
0.163542887	-0.506269881
pseudo_696	0.564309934	0.83091847
0.046116292	-0.783553459
pseudo_697	0.297508058	0.097815509
0.432664131	-0.200774883
pseudo_698	0.87978223	0.783827454
0.138244502	-0.64413501
pseudo_699	0.036365883	-0.515526217	0.055344172
0.801468293
pseudo_7 0.253632926	-1.707883456
0.479576564	-0.677012428
pseudo_70	0.717138907	-0.417201734
0.674964628	-0.200595485
pseudo_700	0.986592156	-1.211870308
0.203892861	-0.791297132
pseudo_701	0.802191261	-1.107846896
0.846604 -0.267655323
pseudo_702	0.575453898	-0.760208351	0.880398824
0.255771484
pseudo_703	0.594796098	0.645292696	0.795550263
0.075469659
pseudo_704	0.104411024	-0.570059923
0.603486907	-0.235112968
pseudo_705	0.023670463	1.56218749	0.982245764
0.055804236
pseudo_706	0.624352712	0.510447606	0.555889627
0.736818396
pseudo_707	0.567483303	0.102226242
0.496740833	-0.351300768
pseudo_708	0.602942064	0.50009841	0.684701116
0.192684403
pseudo_709	0.412033694	-0.345808104	0.200299155

0.545925378
pseudo_71 0.235573898 0.428219 0.696801111 0.212177079
pseudo_710 0.381555362 1.644224661 0.277446247
0.48079914
pseudo_711 0.087589781 0.781165063
0.936144194 -0.326163316
pseudo_712 0.44764146 0.628604953 0.034589214
0.871340187
pseudo_713 0.964152193 0.731212697
0.707815696 -0.313249806
pseudo_714 0.798567094 0.22109769
0.762576021 -0.109784477
pseudo_715 0.397925407 -0.353717975 0.313497153
0.744378665
pseudo_716 0.875468134 -0.12751455 0.760789162
0.092315169
pseudo_717 0.761384641 0.36553679 0.526400256
0.13139879
pseudo_718 0.493775321 0.597492899
0.950451713 -0.421869869
pseudo_719 0.511709694 1.678452145
0.639365007 -0.380278771
pseudo_72 0.093545256 -3.797869892 0.648894066
0.141276079
pseudo_720 0.772127101 -0.459935924
0.274689375 -0.277238985
pseudo_721 0.196210293 0.638127746
0.452800108 -0.109930747
pseudo_722 0.142025256 0.920541208
0.739448852 -0.311173483
pseudo_723 0.086723437 1.955096131
0.546490686 -0.318863496
pseudo_724 0.060079538 1.031087785 0.752467493
0.173918175
pseudo_725 0.38283251 0.016120339 0.589932773
0.234960801
pseudo_726 0.820374496 0.019913231
0.439735088 -0.333923898
pseudo_727 0.788322411 -0.105923626
0.804609742 -0.596930506
pseudo_728 0.072527368 -1.386869231 0.486892798
0.099361595
pseudo_729 0.479090959 -0.479203298
0.272634036 -0.206303853
pseudo_73 0.17743287 -0.078655049 0.016048562
0.72748998
pseudo_730 0.501206846 0.537040646 0.454684271
0.156657808
pseudo_731 0.760789163 -0.538265678 0.040841722
0.688355392

pseudo_732 0.670401386 0.781781672
0.112136789 -0.191751236
pseudo_733 0.64440252 -0.32767952 0.202228439
0.439311555
pseudo_734 0.748317354 0.100654879
0.855182147 -0.278517385
pseudo_735 0.625017165 -0.324038171 0.941524393
0.081191958
pseudo_736 0.741219827 0.473775205 0.245279311
0.524926231
pseudo_737 0.090826271 -1.356493925
0.624906001 -0.0588372
pseudo_738 0.931793607 0.529730979
0.657350122 -0.184157987
pseudo_739 0.147844618 -0.977434408 0.71188931
0.261247358
pseudo_74 0.53322065 -1.017781477 0.119685599
0.819056214
pseudo_740 0.366434224 -4.184323296
0.421902959 -0.224843558
pseudo_741 0.899547377 -0.831502922 0.404061613
0.725574065
pseudo_742 0.274689377 -0.56524314
0.547010777 -0.725233532
pseudo_743 0.850890868 0.829600546
0.031441892 -0.853148314
pseudo_744 0.021274852 -1.161602346 0.646646683
0.211518924
pseudo_745 0.917513857 -0.237948426 0.613880724
0.096283642
pseudo_746 0.137621989 -3.573721315
0.498723104 -0.39668526
pseudo_747 0.334584153 -1.68809523 0.351692507
0.71582355
pseudo_748 0.908834548 -0.206607877 0.984721509
0.080103063
pseudo_749 0.252011269 0.237884962 0.128149062
0.459116896
pseudo_75 0.510704498 0.43108314 0.361478054
0.76084842
pseudo_750 0.016891995 1.971087958 0.436963856
0.556781636
pseudo_751 0.781712834 -0.564611215
0.09082627 -0.348064535
pseudo_752 0.84415639 -0.356342067 0.954186926
0.001397332
pseudo_753 0.766153529 1.375486857
0.685850076 -0.257665242
pseudo_754 0.409809923 -0.351969438
0.822198314 -0.113428503

pseudo_755	0.568013027	-2.373776244	
0.619934036	-0.309910203		
pseudo_756	0.48885393	-0.465990625	
0.378585532	-0.215715657		
pseudo_757	0.894599755	-0.97566699	0.591011927
0.202348461			
pseudo_758	0.43973509	-0.832559012	
0.261850124	-0.7270599		
pseudo_759	0.386251023	1.06521685	0.01594578
0.744735486			
pseudo_76	0.24496192	0.989333613	0.534596637
0.381251202			
pseudo_760	0.897073065	-1.663301211	0.758408669
0.159175778			
pseudo_761	0.881015491	-0.10750246	0.699693608
0.196940987			
pseudo_762	0.75721928	-0.839086728	0.655655354
0.036275201			
pseudo_763	0.148274991	1.207612087	
0.648331919	-0.109101991		
pseudo_764	0.634344075	-1.328553987	0.166876062
0.700311549			
pseudo_765	0.593172956	-1.723905043	
0.822806468	-0.75509482		
pseudo_766	0.426434428	0.516774314	0.595337594
0.234766309			
pseudo_767	0.329907103	0.800017382	0.113734738
0.292762365			
pseudo_768	0.378162438	-0.981356882	0.308643617
0.276404822			
pseudo_769	0.696223139	-0.643035232	0.073026191
0.295195532			
pseudo_77	0.448109044	-0.555071184	
0.878549261	-0.178259723		
pseudo_770	0.676107318	-0.379245571	0.16259983
0.407524075			
pseudo_771	0.309387065	1.546556875	
0.68011259	-0.070810454		
pseudo_772	0.538202697	-1.001392401	0.138869185
0.542418631			
pseudo_773	0.523852116	0.196138492	
0.999064525	-0.106395234		
pseudo_774	0.072651812	-1.57287316	0.184052893
0.644587993			
pseudo_775	0.289728691	0.498946909	
0.553270914	-0.436958904		
pseudo_776	0.555889629	0.452291831	
0.545451239	-0.17639425		
pseudo_777	0.52620363	-0.14450498	0.65269988
0.238556351			

pseudo_778 0.764960468 −0.47260408 0.974123063
0.012050888
pseudo_779 0.296547379 0.282388872
0.522834633 −0.181493794
pseudo_78 0.926203007 −0.019536485 0.394444355
0.241000385
pseudo_780 0.793882574 −0.466944884 0.211184349
0.599149729
pseudo_781 0.868081212 −0.196998737 0.990333661
0.008281907
pseudo_782 0.393576982 −0.463468075 0.095238749
0.706589504
pseudo_783 0.841710283 0.424789164
0.551180305 −0.180774202
pseudo_784 0.695067724 0.182026592 0.518774877
0.323559455
pseudo_785 0.997193581 −0.571426194 0.40318156
0.253048191
pseudo_786 0.807030097 0.968172897
0.664146758 −0.470310879
pseudo_787 0.467996415 0.065809416 0.999064525
0.122268694
pseudo_788 0.234341663 1.217597134
0.776915721 −0.271224116
pseudo_789 0.004326258 −4.279940789
0.57066519 −0.145761261
pseudo_79 0.25233502 1.880316532
0.798567094 −0.219670175
pseudo_790 0.059022494 2.110652933 0.197023132
0.177346404
pseudo_791 0.932414998 −0.001487685 0.613331717
0.267698502
pseudo_792 0.770931293 0.582564859
0.663579306 −0.003008073
pseudo_793 0.439272519 0.438268432
0.060936431 −0.75329494
pseudo_794 0.244328001 0.776995499
0.343667322 −0.500533621
pseudo_795 0.037140641 1.670923665
0.827066506 −0.159692628
pseudo_796 0.296547379 1.129619996
0.051296279 −0.615169357
pseudo_797 0.464171067 0.250350089
0.415606573 −0.444409488
pseudo_798 0.372688717 1.499929084
0.76198026 −0.34803403
pseudo_799 0.669262465 0.482186509
0.762576021 −0.305380455
pseudo_8 0.393576982 −0.609179142 0.594254823 0.2280195
pseudo_80 0.302372052 −1.159895316

0.868696358	-0.392682104		
pseudo_800	0.895085201	-1.875193115	
0.0242878	-0.655468983		
pseudo_801	0.74950237	2.229988467	
0.387967243	-1.208387552		
pseudo_802	0.708397134	0.089607523	0.930240313
0.12054041			
pseudo_803	0.59209199	-0.979655757	0.265523348
0.271405023			
pseudo_804	0.928687285	0.232388105	0.53254152
0.036066471			
pseudo_805	0.515740784	0.859655785	
0.708397133	-0.275859086		
pseudo_806	0.755436274	-0.371781513	
0.326042059	-0.193062774		
pseudo_807	0.337371437	0.368996821	0.649731189
0.387251437			
pseudo_808	0.024553442	-3.323518996	
0.105752713	-0.721077308		
pseudo_809	0.108821518	1.416120714	
0.260854459	-0.236299456		
pseudo_81	0.079904474	0.763698933	
0.219616342	-0.324715557		
pseudo_810	0.12658838	1.929879769	0.844156389
0.062391795			
pseudo_811	0.175930669	1.185931348	0.1496034
0.332948589			
pseudo_812	0.00762834	1.888753927	
0.369761763	-0.691400141		
pseudo_813	0.773617176	-0.743130227	
0.344054419	-0.014072625		
pseudo_814	0.069352604	-4.134591478	0.371432554
0.872810443			
pseudo_815	0.838043993	-0.884847109	
0.165918558	-0.660263409		
pseudo_816	0.532541522	-0.658184857	
0.801586934	-0.358938719		
pseudo_817	0.946095346	-0.767288323	
0.339501292	-0.272065231		
pseudo_818	0.200024645	1.723597735	0.66244498
0.702355758			
pseudo_819	0.358197406	0.412673012	0.868081211
0.277976474			
pseudo_82	0.868081212	-0.076302644	0.729439665
0.478245135			
pseudo_820	0.063123042	-3.497093184	0.555365401
0.766800755			
pseudo_821	0.813695537	-0.656352785	0.200024643
0.263436888			
pseudo_822	0.343269159	0.009558683	

0.947962171	-0.212371218
pseudo_823	0.607307007	0.796570357	0.317643015
0.178234773
pseudo_824	0.878549261	-1.198183135	0.677250751
0.190686047
pseudo_825	0.510202287	1.569910722	0.600764898
0.679643815
pseudo_826	0.201124341	-1.773108561
0.644963257	-0.070677083
pseudo_827	0.026087415	0.680972233
0.492788924	-0.046971164
pseudo_828	0.639644437	-1.068179806
0.938631108	-0.227575695
pseudo_829	0.47135787	-0.465640363	0.40098646
0.227242273
pseudo_83	0.73414425	0.842498324	0.899547376
0.277054514
pseudo_830	0.801586935	-1.214939371	0.593713776
0.261382788
pseudo_831	0.886568692	0.610582533	0.596421264
0.30432729
pseudo_832	0.088317055	-3.270218423	0.87670036
0.282827778
pseudo_833	0.681258621	-1.028090601	0.554579514
0.160137451
pseudo_834	0.310131694	1.415646715	0.4337435
0.203319195
pseudo_835	0.322206576	0.901969429
0.066292872	-0.459490143
pseudo_836	0.361478056	0.05184304	0.990957268
0.136772694
pseudo_837	0.457518784	-0.896317238
0.017512732	-0.950663726
pseudo_838	0.410698573	-0.436421996	0.91441292
0.117646953
pseudo_839	0.995322652	-0.33713232	0.565366772
0.099027559
pseudo_84	0.147407401	0.667372097	0.913792887
0.555822067
pseudo_840	0.404061615	0.786675532
0.491311314	-0.371213539
pseudo_841	0.637131424	-0.979656733	0.549093589
0.672879645
pseudo_842	0.855795541	-0.420745133
0.977239862	-0.281262566
pseudo_843	0.025157391	1.822243643
0.753060956	-0.158671017
pseudo_844	0.827066507	-0.526142497	0.600221163
0.09316367
pseudo_845	0.046976608	2.097607453

0.362301151	-0.330856024
pseudo_846	0.693335928	0.006590379
0.884716992	-0.438285217
pseudo_847	0.033533157	-2.275548338	0.533568579
0.442832276
pseudo_848	0.553999593	1.198582108
0.358534191	-0.35692664
pseudo_849	0.727091176	-1.025645581
0.874852133	-0.441416764
pseudo_85	0.489836089	0.53254014	0.004200248
0.656182644
pseudo_850	0.951696668	-0.367173936	0.598591294
0.165110122
pseudo_851	0.739448853	-2.320886495
0.283016371	-0.386785176
pseudo_852	0.194529717	-2.609023097
0.335713813	-0.340600125
pseudo_853	0.947962171	0.125372595
0.983474456	-0.215028608
pseudo_854	0.224647986	2.009842497	0.465125767
0.060857945
pseudo_855	0.096594502	-0.510245794	0.247535442
0.250783743
pseudo_856	0.858863804	0.547153224	0.78231306
0.12186786
pseudo_857	0.167116087	2.597703193
0.121430141	-0.744930286
pseudo_858	0.433147665	0.019131014	0.828893803
0.221981005
pseudo_859	0.326427236	0.23723124	0.039102109
0.805577794
pseudo_86	0.188216657	-2.116274489	0.713637673
0.142519671
pseudo_860	0.139705475	-1.706303966	0.001984671
0.871946311
pseudo_861	0.836822673	-0.81547891	0.91255298
0.393677778
pseudo_862	0.190311557	0.714129764
0.84319479	-0.228772935
pseudo_863	0.549093591	-1.635751578
0.113200108	-0.884318482
pseudo_864	0.345738283	-0.711243899	0.732317705
0.04825857
pseudo_865	0.934279416	-0.007969342	0.151378102
0.408521482
pseudo_866	0.908834548	-1.890658318
0.657915438	-0.361441896
pseudo_867	0.668693289	-0.03898256	0.545451239
0.166646126
pseudo_868	0.201675839	0.397460861

0.326427232	-0.33372039		
pseudo_869	0.953992226	0.616422126	
0.102003345	-0.744917679		
pseudo_87	0.352906444	0.867864778	
0.241175679	-0.541742384		
pseudo_870	0.836822673	0.576810632	
0.863777444	-0.248501611		
pseudo_871	0.281616789	-3.710322495	0.89027394
0.099166008			
pseudo_872	0.579726399	-0.496732787	
0.77332345	-0.13357594		
pseudo_873	0.905737376	0.414508714	
0.871773274	-0.037081516		
pseudo_874	0.19138479	-3.397871097	
0.459888433	-0.232160668		
pseudo_875	0.017967568	1.64020089	0.511251964
0.170791262			
pseudo_876	0.122183783	-0.249756759	
0.36312542	-0.039273403		
pseudo_877	0.173201299	-2.619381978	
0.400110432	-0.390825534		
pseudo_878	0.382406503	-0.371583616	
0.936144194	-0.26418992		
pseudo_879	0.799774672	-0.941292726	
0.120306503	-0.229691383		
pseudo_88	0.215823569	0.329295402	0.653398419
0.246485891			
pseudo_880	0.538202697	-0.879029971	0.715971163
0.151807658			
pseudo_881	0.607307007	-0.285771437	0.943606708
0.079018371			
pseudo_882	0.840487801	0.283216373	0.518268555
0.383915598			
pseudo_883	0.85763624	0.126519907	
0.692759016	-0.334546273		
pseudo_884	0.953564319	0.242490258	0.445307706
0.167159818			
pseudo_885	0.007297747	2.325468737	0.206689013
0.381559596			
pseudo_886	0.553794174	0.855719738	
0.906356696	-0.441967971		
pseudo_887	0.440197941	0.574825333	0.170260038
0.469213878			
pseudo_888	0.924961136	0.57097356	0.906356696
0.430072295			
pseudo_889	0.379008922	-0.372891201	0.268216974
0.898310365			
pseudo_89	0.543375295	0.52999058	0.287243364
0.889622794			
pseudo_890	0.773323451	0.576375274	0.386251021

0.224535537
pseudo_891 0.9007849 0.389442742
0.978486682 -0.178810734
pseudo_892 0.201130169 -1.300594369 0.31663046
0.467718028
pseudo_893 0.063345226 1.877660525 0.825848827
0.274485301
pseudo_894 0.344863589 0.048196561
0.933657902 -0.731731079
pseudo_895 0.920616048 -0.977545293 0.215533782
0.500832762
pseudo_896 0.318400653 1.396389163 0.190854059
0.410050078
pseudo_897 0.390981794 -0.803935135
0.103082887 -0.587242646
pseudo_898 0.828893804 1.433983825 0.30975923
0.163435453
pseudo_899 0.523852117 -0.820033866
0.246551764 -0.211522298
pseudo_9 0.363734894 0.869805178 0.318400651 0.227206275
pseudo_90 0.825240145 0.07904407
0.827066506 -0.39917763
pseudo_900 0.476666955 -0.900039924
0.916273328 -0.415481256
pseudo_901 0.071907764 1.881299487 0.859477714
0.227280228
pseudo_902 0.265859031 -0.565073418
0.442980904 -0.517836851
pseudo_903 0.191916605 1.075904206 0.301639832
0.104819024
pseudo_904 0.735321965 0.897347821
0.697379259 -0.25316343
pseudo_905 0.249110533 -1.586313725 0.267541814
0.191673866
pseudo_906 0.13413515 2.367001059
0.503198543 -0.181671215
pseudo_907 0.554841418 2.150049538 0.588854528
0.152600937
pseudo_908 0.455156003 -1.048053399 0.769138601
0.534981913
pseudo_909 0.863234425 0.455709595 0.021318862
0.73246211
pseudo_91 0.956677633 0.585323841
0.604031974 -0.194573163
pseudo_910 0.992204499 1.196201808 0.486146285
0.195554556
pseudo_911 0.767347144 -0.630112503 0.507695109
0.082683651
pseudo_912 0.257554503 -0.321337432 0.616078914
0.129240223

pseudo_913	0.319539326	-0.052535669	0.877316586	0.554256316
pseudo_914	0.514227197	0.094643013	0.934900968	-0.291637975
pseudo_915	0.677250753	0.381498157	0.714804085	0.122218636
pseudo_916	0.805819687	-2.839221292	0.291512759	1.075554775
pseudo_917	0.070559892	1.780506888	0.329131728	0.115243362
pseudo_918	0.876700361	0.442179762	0.913172907	-0.065899405
pseudo_919	0.433284573	-0.15204927	0.484935926	-0.601556091
pseudo_92	0.285476969	1.096244054	0.360656131	-0.122642203
pseudo_920	0.635559438	0.851354722	0.145291448	0.457601503
pseudo_921	0.839265703	0.282248451	0.537686798	0.149000771
pseudo_922	0.176931079	0.614447793	0.352906442	-0.341203599
pseudo_923	0.780512769	0.805595871	0.390981792	0.036543984
pseudo_924	0.186647176	1.128731587	0.360656131	-0.407234001
pseudo_925	0.882865922	-0.318780327	0.455628007	-0.207666585
pseudo_926	0.017000113	1.614360615	0.21236462	-0.796628332
pseudo_927	0.47763575	1.117800109	0.457992163	0.067379465
pseudo_928	0.018973025	0.797769979	0.28512457	0.69081038
pseudo_929	0.097924719	-0.97774908	0.94742861	0.097907582
pseudo_93	0.016927967	-2.246818833	0.056852908	0.353196544
pseudo_930	0.463217456	-0.632452914	0.929308464	0.158262295
pseudo_931	0.11890377	0.663249799	0.764169708	0.256739923
pseudo_932	0.397489266	-0.127520765	0.781712833	0.170105157
pseudo_933	0.791934102	0.55398891	0.899547376	-0.327470962
pseudo_934	0.040996046	0.864308616	0.560618501	-0.222982997
pseudo_935	0.865006686	0.389784742	0.087300226	0.621576847

pseudo_936	0.176931079	0.297551996	0.329519264	−0.117574312
pseudo_937	0.783513905	0.488018717	0.537686798	−0.062163223
pseudo_938	0.825240145	−2.49836132	0.556938804	−0.153783841
pseudo_939	0.408036055	−0.713840439	0.487382682	0.45901162
pseudo_94	0.849053092	0.923482123	0.563254045	−0.077247333
pseudo_940	0.998027146	−0.656032328	0.344895695	0.281927274
pseudo_941	0.659046656	−0.539275109	0.226443376	−0.639549279
pseudo_942	0.949829282	0.773965197	0.900166107	−0.176378388
pseudo_943	0.982850945	0.236987073	0.376051325	0.039864126
pseudo_944	0.111303949	1.885709491	0.332825614	0.347717898
pseudo_945	0.918754585	−0.298221845	0.394444355	−0.134512851
pseudo_946	0.646646684	0.10937011	0.402302658	−0.698109433
pseudo_947	0.814908901	0.753869185	0.590472236	−0.52021583
pseudo_948	0.328744488	−0.519062029	0.954809562	−0.616244897
pseudo_949	0.895217987	0.561884946	0.377317117	0.314233198
pseudo_95	0.727091176	−0.76403631	0.230064819	−0.2491488
pseudo_950	0.319159473	1.074923437	0.377739631	0.108307158
pseudo_951	0.257883189	−0.97954602	0.199476445	0.57077261
pseudo_952	0.56589555	−0.222990012	0.362301151	−0.0384422
pseudo_953	0.155429768	−2.568821705	0.862548532	−0.196412217
pseudo_954	0.997193581	0.264380676	0.589932773	−0.187315245
pseudo_955	0.984409742	−2.09494	0.067573524	−0.859315829
pseudo_956	0.68642483	0.41474716	0.013736291	−0.709287185
pseudo_957	0.978486682	−0.379400815	0.365605264	0.306449152
pseudo_958	0.712471931	−0.147964151	0.200024643	−0.637275182

pseudo_959	0.021318862	1.601753311	0.060506727	1.192446546
pseudo_96	0.166408604	−1.19918009	0.838039167	0.086696398
pseudo_960	0.536140599	−0.401508508	0.641601866	0.412805033
pseudo_961	0.187692418	−0.941560335	0.68011259	−0.015077815
pseudo_962	0.047499248	1.255075099	0.956677633	0.084109019
pseudo_963	0.327584532	1.289030132	0.045437629	−1.066363263
pseudo_964	0.561145133	−0.931584289	0.162835209	−1.089599924
pseudo_965	0.18225285	1.134663043	0.919995511	−0.303224645
pseudo_966	0.318779915	−0.672242197	0.007029386	−1.029727226
pseudo_967	0.532028368	−2.397620057	0.110207846	−0.5462164
pseudo_968	0.999064525	0.426431326	0.519788287	−0.185026428
pseudo_969	0.782313061	0.310143324	0.938009322	0.30698809
pseudo_97	0.628230174	−0.125249946	0.511206965	0.199988092
pseudo_970	0.563781871	0.205383639	0.571727705	0.051937081
pseudo_971	0.308643619	0.624223783	0.506193914	0.197815186
pseudo_972	0.480488268	−2.33306435	0.154143795	−0.913202736
pseudo_973	0.334192774	0.166064471	0.485913829	0.032882197
pseudo_974	0.274346087	0.349050635	0.98971006	−0.260899834
pseudo_975	0.42054905	−0.499883648	0.177684161	−0.565387955
pseudo_976	0.89027394	0.488637877	0.939874791	0.244048043
pseudo_977	0.638247806	0.406225737	0.849665593	0.322980977
pseudo_978	0.918754585	0.428230524	0.347264075	−0.259881858
pseudo_979	0.651707796	1.5603038	0.778114209	−0.330535389
pseudo_98	0.805214657	−2.329860426	0.910693519	−0.170322962
pseudo_980	0.791934102	−0.175921126	0.296547377	0.342847591

pseudo_981	0.449982149	0.724016828	0.8298078
0.075010384			
pseudo_982	0.036365883	1.77772823	0.237120629
0.944157098			
pseudo_983	0.160959364	1.134528462	
0.969760218	-0.247030611		
pseudo_984	0.670626329	0.569520686	0.970413948
0.193591998			
pseudo_985	0.978486682	-2.293423321	
0.573855552	-0.529475686		
pseudo_986	0.195399911	-0.250154742	0.19432322
0.765079669			
pseudo_987	0.476666955	0.748091185	
0.8149089	-0.16103525		
pseudo_988	0.238052108	-0.902989715	
0.482983322	-0.12586542		
pseudo_989	0.149667696	1.850861561	
0.504912707	-0.421835607		
pseudo_99	0.081669658	-1.669305738	0.243695232
0.302724348			
pseudo_990	0.533568581	0.617081041	
0.193518566	-0.634386156		
pseudo_991	0.027318913	1.089181324	0.987215722
0.108724559			
pseudo_992	0.961215791	-0.873272749	
0.145063863	-0.331050196		
pseudo_993	0.652834681	-0.065391954	
0.024454014	-0.479750867		
pseudo_994	0.580796845	0.487221985	
0.984097978	-0.208737058		
pseudo_995	0.938631108	0.42320261	0.471357868
0.029670772			
pseudo_996	0.784715264	-1.169674002	
0.166636297	-1.132301		
pseudo_997	0.317075563	0.567354417	
0.022676552	-0.933377499		
pseudo_998	0.097757646	-1.20775338	
0.587026842	-0.318571226		
pseudo_999	0.056548454	1.581529412	
0.212938315	-0.551530809		
